# Communicative Efficiency or Iconic Learning: Do Acquisition and Communicative Pressures Interact to Shape Colour- Naming Systems?

**DOI:** 10.3390/e24111542

**Published:** 2022-10-26

**Authors:** Balint Gyevnar, Gautier Dagan, Coleman Haley, Shangmin Guo, Frank Mollica

**Affiliations:** School of Informatics, University of Edinburgh, Edinburgh EH8 9AB, UK

**Keywords:** colour-naming systems, communicative efficiency, language evolution, information bottleneck

## Abstract

Language evolution is driven by pressures for simplicity and informativity; however, the timescale on which these pressures operate is debated. Over several generations, learners’ biases for simple and informative systems can guide language evolution. Over repeated instances of dyadic communication, the principle of least effort dictates that speakers should bias systems towards simplicity and listeners towards informativity, similarly guiding language evolution. At the same time, it has been argued that learners only provide a bias for simplicity and, thus, language users must provide a bias for informativity. To what extent do languages evolve during acquisition versus use? We address this question by formally defining and investigating the communicative efficiency of acquisition trajectories. We illustrate our approach using colour-naming systems, replicating a communicative efficiency model based on the information bottleneck problem, and an acquisition model based on self-organising maps. We find that to the extent that language is iconic, learning alone is sufficient to shape language evolution. Regarding colour-naming systems specifically, we find that incorporating learning biases into communicative efficiency accounts might explain how speakers and listeners trade off communicative effort.

## 1. Introduction

Language is a flexible cognitive technology that culturally evolves over multiple timescales [1]. For example, learners might be exposed to slightly different input than the past generation and, therefore, change the language they use to better reflect their environment [2]. Interactions between strangers might inspire new linguistic conventions, which may last for the interaction or permeate into common use [3,4]. At a larger timescale, environments and the goals we want to achieve change, inspiring language change [5]. Regardless of the timescale, the evolution of language requires two pressures [6]: a bias towards *informativity* to ground the communicative task and a way to *lose information* to both permit innovation and adapt to novel communicative scenarios. At what timescales are these criteria minimally established?

Although there are many approaches to studying the complex dynamics of language evolution [7,8], cultural evolutionary approaches typically focus on two timescales: language acquisition and dyadic coordination. Language acquisition provides an opportunity for the next generation of a language to lose information about the environment both by forgetting environmental statistics that are no longer relevant and by simplifying, or regularising, patterns that are now prominent in the environment [2,9]. In contrast, dyadic coordination pressures a system towards informative (non-ambiguous) conventions that are easy to use but readily discarded across conversation partners [3,10,11]. Taken together, it is reasonable to think that both timescales interact to shape language (as argued extensively by [12,13,14]); however, it has also been argued that the communicative timescale and, more recently, the acquisition timescale have independent pressures for information loss and informativity, independently satisfying the criteria for language evolution. In this case, acquisition models would be minimally sufficient to explain language evolution. That said, there have been some concerns that the pressure for informativity in acquisition might better reflect other learning biases [14,15]. We briefly review the arguments that dyadic coordination and acquisition independently satisfy the criteria for language evolution and the concerns about an informativity bias in acquisition. We then provide a formal analysis of acquisition and communication to address these concerns, predicting when acquisition biases for simplicity and iconicity will provide a pressure for informativity. We illustrate our account with the domain of colour-naming systems, demonstrating that a learning bias for iconicity provides an informativity pressure. Therefore, acquisition is a sufficient timescale for explaining the evolution of colour-naming systems.

### 1.1. How Might Languages Evolve Due to Communication?

As early as Zipf [16], languages have been described as efficient trade-offs between speaker and listener processing considerations. Speakers want languages to be as simple as possible; in the degenerate case, there is one word to rule them all (i.e., the word is the *only* word with which to refer to anything), resulting in maximum information loss. On the other hand, listeners want languages to be as informative as possible (e.g., a word for every possible state of the world in every imaginable universe). Therefore, both requirements for language evolution arise without any consideration for the acquisition of the system. Several researchers have argued that speaker–listener interaction is vital for *informative* signs to emerge [10,17,18]. Information is lost through shifting conversation partners and generational turnover, which allows alignment on a generalisable language [19]. Thus, language evolution can occur through dyadic coordination alone (for a review of the empirical literature see [4]).

Recently, the speaker–listener trade-off has been formalised at Marr [20]’s computational level in semantic typology to demonstrate that, at least for some domains, these communicative efficiency constraints can explain both the diversity of attested language systems [21,22,23,24,25] and arguably their evolution [26,27]. Under this account, languages evolve by traversing the Pareto frontier of the communicative efficiency trade-off by increasing speaker effort. While this account has been wildly successful, it is not without its criticisms. Levinson [28] pointed out that although diversity is well explained, the model does not account for the *process* by which these systems emerge.

### 1.2. How Might Languages Evolve Due to Acquisition?

There are several ways in which learners might lose information about the language held by a prior generation. To start, a learner’s input reflects the current environment, which may differ drastically from the environment in which previous generations acquired language [5]. More relevant to cultural evolution accounts, in the face of noisy inconsistent grammatical input, learners prefer to adopt a simple, mostly correct rule rather than the set of rules and exceptions often required to capture the nuance of a language [2,9,29]. This process of simplification, or regularisation, acts as a natural pressure to lose information.

The real challenge of an acquisition account of language emergence is to provide a pressure for informativity. It has been argued that without dyadic communication, cross-generational learning simplifies an initial language resulting in less informative systems [13,30,31]. Recently, it has been demonstrated that learners might repurpose optional variations in their input (e.g., using *don’t* and *do not* equally in statements, commands, and questions) into a more informative language (e.g., only using *do not* as a command). This form of regularisation would suggest that learners have an inductive bias towards informativity. In an important artificial language learning experiment, Fedzechkina et al. [32] demonstrated that learners could regularise the use of optional case markers in a way that increases the informativity of the initial input language. Similarly, Kurumada and Grimm [33] demonstrated that learners will acquire complex yet informative patterns of nominal number marking not present in their input language. In another iterated-learning experiment, Carstensen et al. [34] generalised this finding from grammatical features to semantic categories, illustrating an informativity bias with colour and spatial relations. Crucially, none of these studies included dyadic communication, which suggests that there may be an inductive bias that guides learners to regularise for more informative languages.

Recently, this informativity bias in acquisition has been challenged. In a series of experiments, Smith and Culbertson [15] failed to replicate Fedzechkina et al. [32]. They argued that instead of an informativity bias, the original results may have been driven by an early learning bias to use longer linguistic codes (e.g., an additional case marker) to communicate unusual events. This bias has been referred to as *iconicity of markedness matching* [35] because the addition to the linguistic code is as unusual as the event the code signifies. While this form of iconicity also contributes to communicatively efficient coding, it suggests that an iconicity bias might provide the same functional pressure as an informativity bias. Similarly, Carr et al. [14] demonstrated that including an inductive bias for informativity, compared to a bias for simplicity, provides a poor fit to data in an artificial language learning experiment with semantic categories. Specifically, they argued that a bias for simplicity can provide the same functional pressure as informativity and give rise to categories similar to those seen in Carstensen et al. [34]. In light of their experiments, Carr et al. [14] argued that language evolution is shaped over both timescales, i.e., language acquisition and dyadic communication (see also [12,13]).

Taken together, it appears that acquisition biases for iconicity and simplicity may moonlight as an informativity bias, but only sometimes. When they act as an informativity bias, the requirements for language evolution are met and acquisition is a sufficient timescale for language evolution (as in [32,34]). When they do not provide the same pressure as an informativity bias, languages move towards simple degeneracy [13,30,31]. Under what conditions do simplicity and iconicity result in a pressure for informativity?

We propose a formal analysis to investigate the timescales of acquisition and explain the conditions under which well-established learning biases (simplicity and iconicity) provide the same functional pressure as an informativity bias. In the next section, we outline our proposed explanation. We then illustrate our analysis using the domain of colour terms, as there exist well-motivated models of both colour-term communicative efficiency and colour acquisition. Specifically, we reimplement Zaslavsky et al. [22]’s communicative efficiency model and Beekhuizen and Stevenson [36]’s colour acquisition model. We use the models to weigh in on whether both acquisition and communicative timescales are required to explain language evolution by investigating if and when the minimal conditions for language evolution (information loss and informativity) are met by acquisition models, i.e., are learners’ languages communicatively efficient over the course of acquisition? Along the way, we investigate whether the acquisition trajectory, i.e., the ordered set of languages that a learner infers as they see more data, includes languages that are more informative than adult-like language, as suggested by Fedzechkina et al. [32] among others.

Lest we leave you in suspense, we demonstrate that when a learner’s conceptual hypotheses about the world align with the meanings speakers want to convey, learning biases for simplicity and iconicity can function as a pressure for informativity. We demonstrate this alignment with colour naming, suggesting that acquisition is a sufficient timescale for explaining the evolution of colour naming. We do not find evidence of an additional bias for informativity in learning. We take this illustration as a promising direction for future work investigating how communicative efficiency and acquisition dynamics interact.

## 2. Our Approach

Formally, we see acquisition and communication as two distinct lossy compressions, i.e., Rate-Distortion Theory (RDT) problems [37]. An RDT problem describes the *optimal* efficiency trade-off between the amount of information compressed from a source domain into a representation and the amount of distortion (error) caused by using this representation instead of the source for some downstream task (e.g., communication). By definition, lossy compression problems satisfy both requirements for language evolution: maximising compression results in simple systems that *lose information* and minimising error results in complicated, *informative* systems. In formalising acquisition, a learner wants to lossily compress their experience of the universe into mental representations to robustly reconstruct the world [38], as argued by control theory [39]. As argued by Zaslavsky et al. [22], the process of communication (i.e., source coding) is also lossy compression: speakers compress meanings into language in order for listeners to reconstruct the meaning successfully.

In an RDT model, we need to specify (1) the domain (i.e., the conceptual universe of possible world states/meanings); (2) a source distribution over this domain (e.g., the environmental frequency or communicative need); and (3) a cost function that captures the impact of misrepresenting the source domain for a downstream task (e.g., communication/reconstruction). With these assumptions, we can then describe the optimal trade-off between compression (complexity) and distortion (information loss).

Following Zaslavsky et al. [22], we use the Kullbach–Leibler (KL) divergence [40] as a cost function, which results in a well-studied rate-distortion problem known as the Information Bottleneck (IB) [41,42]. For IB problems, the cost function requires us to specify the expected imprecision or similarity (generalisation) gradient for each item in the domain. For example, in Zaslavsky et al. [22]’s model of colour communication, the domain is colour meanings, and the precision of a specific colour meaning is given by a Gaussian distribution over perceptual colour space, centred at that colour meaning. This distribution places an implicit order over the domain, governing the compression patterns.

We propose that the source distribution across acquisition and communication are roughly the same. Thus, the main difference between the acquisition and communication RDT problems lies in the generalisation gradients over world states. In acquisition, this distribution reflects a learner’s *hypotheses* about concepts, whereas in communication, this distribution reflects the precision of *meanings*. Given that hypotheses and meanings are distributions over the same conceptual universe, it would make sense to align or combine them; however, hypotheses and meanings are theoretically distinct and it has been argued that different biases influence them. For example, informativity is thought to influence communication, not acquisition, as summarised in the introduction.

**Figure 1:**
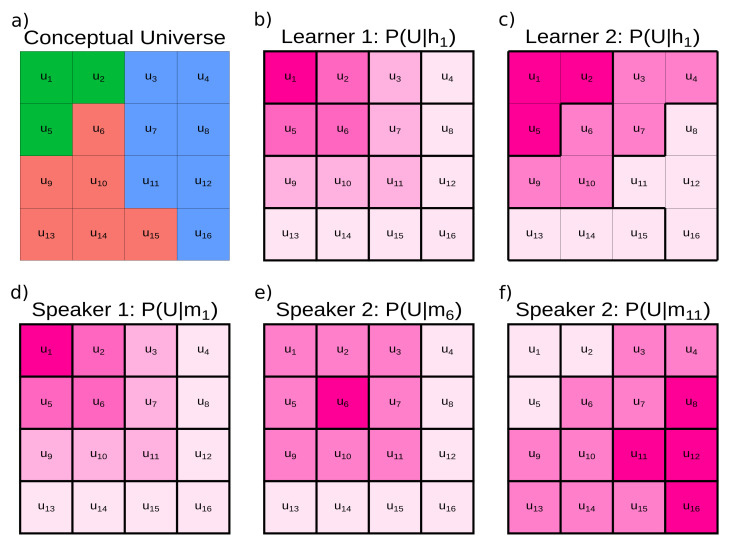
Illustration of learners’ hypotheses and speakers’ meanings. Iconicity occurs when learners’ hypotheses and speakers’ meanings are isomorphic, i.e., when there is a correspondence between a hypothesis *h* and a meaning *m* for every state of the world *u* (e.g., between Learner 1 and Speaker 1). (**a**) A conceptual universe of world states U and the relevant distinctions in the world reflected by fill colour. (**b**) A learner’s hypothesis about how world states are related to the world state $u_{1}$, i.e., $P(U|h_{1})$. The brightness of shading reflects probability, with darker shades for higher probabilities/stronger generalisations. (**c**) Another learner’s hypothesis for the same underlying world state: $P(U|h_{1})$. In comparison to the first learner, this learner readily generalises to some adjacent states (e.g., compare $u_{2}$ and $u_{5}$ across learners) but does not generalise as readily to others (e.g., compare $u_{11}$). (**d**) A speaker’s meaning when intending to communicate about world state $u_{1}$. (**e**) A second speaker’s meaning when intending to communicate about world state $u_{6}$. (**f**) The second speaker’s meaning when intending to communicate about world state $u_{11}$. The differences between the speaker’s meanings for $u_{6}$ and $u_{11}$ are to illustrate that meanings for different world states are not required to have a fixed rule or analytical form, even within a speaker.

In our analysis, iconicity results from a structural isomorphism between hypotheses and meanings [43,44], that is, learners will generalise a world state similarly to how speakers will extend a meaning. For illustration, let us consider a conceptual universe of world states U and some relevant distinctions over the states of the world (the coloured partitioning in Figure 1a). A learner wants to reconstruct this structure based on their hypotheses $h_{u}$ about how world states are related $P(U|h_{u})$. We can imagine a learner that has a unique hypothesis for every world state, placing a high probability (darker shading) on the corresponding world states and a decreasing probability (lighter shading) on the neighbouring world states (Figure 1b). We can also imagine a learner that does not have a unique hypothesis for each world state (e.g., in Figure 1c $P\left(U|h_{1}\right)=P\left(U|h_{5}\right)=P\left(U|h_{2}\right)$), but still places a high probability on some world states and a decreasing probability on the neighbouring world states. For both learners, reconstructing the world involves compressing hypotheses to preserve relevant distinctions in the world, resulting in better hypotheses about how world states are related. However, the different generalisation patterns defined by the hypotheses mean that compressing world states for a learner using the first hypothesis space will result in a different acquisition trajectory than for a learner using the second hypothesis space.

We can visualise the communication RDT problem in the same way. Imagine a speaker equipped with meanings $P(U|m_{u})$ similar to our first learner (Figure 1d): a high probability mass on the corresponding world state with a decreasing probability on adjacent states. We can also imagine a speaker where the shape of $P(U|m_{u})$ differs for each world state (compare Figure 1e,f). Optimal communication requires the speaker’s meanings to be compressed into words such that listeners can reconstruct the intended meaning. If we compress the first speaker’s meanings, we will arrive at a different set of optimal communication systems than if we compress the second speaker’s meanings. The key insight of our approach is that if learners use the first hypothesis space (Figure 1a) and speakers use the first set of meanings (Figure 1d), the compression problem is identical. This is because there exists at least one structure-preserving mapping between the hypotheses and meanings, i.e., structural isomorphism.

Although this is a structural alignment, it may also correspond to the intuitive notion of iconicity by which our perceptions of the world (as guided by our hypothetical concepts) are directly reflected in the meanings of words as in [43], provided learners’ hypotheses reflect sensorimotor content. It’s worth noting that the direction of this relationship is unclear. While learnability has been appealed to in explanations of semantic universals [45] and typological prevalence [46], the presence of linguistic structure is often appealed to for learners identifying important conceptual distinctions in the world in the first place [47,48]. It is important to note that we use iconicity as a mapping between the space of learners’ hypotheses and the space of speakers’ meanings, whereas Smith and Culbertson [15] appealed to iconicity as a form-meaning mapping. We believe that our notion of structural iconicity is distinct from notions of iconicity that rely on direct mappings between the sensorimotor content of linguistic forms and the sensorimotor content of the world, as in onomatopoeia. That said, our formalisation may explain Haspelmath [35]’s *iconicity of markedness matching.* If a learner’s hypotheses and a speaker’s meanings are structurally aligned, then the environmental frequency of world states should be reflected in the need probability of words. Under noisy channel models of communication, word length correlates with the need probability of a word [49]. Therefore, words reflecting rare world states should have longer (marked) forms. Further research will be required to evaluate whether and how our formalism of iconicity might explain iconicity between meanings and linguistic forms.

**Figure 2:**
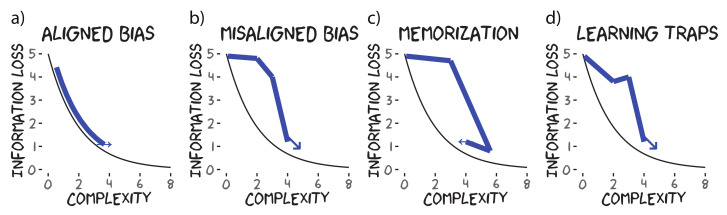
Communicative efficiency of possible acquisition trajectories (in blue). The space below the black line reflects languages with impossible trade-offs between information loss and complexity. The black lines represent languages that achieved an optimal trade-off between information loss and complexity. Possible acquisition trajectories in blue include (**a**) if learners’ hypotheses aligned with speakers’ meanings; (**b**) if learners’ hypotheses did not align with speakers’ meanings; (**c**) if learning was memorisation; and (**d**) when learning was influenced by the local data distribution.

As a consequence of structural iconicity between hypotheses and meanings, optimal communication is formally equivalent to optimal acquisition, and acquisition trajectories should trace the optimal communication trade-off between information loss and complexity (the IB frontier) as shown in Figure 2a. Hypotheses and meanings can also be misaligned. Consider number communication and acquisition. Humans have two representational systems for number cognition: the approximate number system [50] and the object-file system [51]. In modelling the communication problem for number marking, Mollica et al. [52] used approximate number system representations for meanings to explain typological diversity, whereas models of number word acquisition often rely on object-file representations for the hypothesis space ([53], although see [25] for a model that combines both representations). Due to the representational mismatch, the acquisition model bypasses communicatively optimal systems with approximate number meanings (e.g., *a few/paucal*) for systems with sub-optimal simplicity-information loss trade-offs (Figure 2b). That said, we still expect the acquisition trajectory to move towards complex, informative systems as they see more data. In other words, *simple* hypotheses for a learner may reflect complex, less-informative partitions for communication.

Now, we are interested in more than just the consequences of representational misalignment on *optimal* communication/acquisition. While optimal models of acquisition equipped with proper inductive biases/constraints often closely track learning behaviours [54], our analysis is generalisable to other learning models. For example, we can consider a theoretical learning algorithm that simply memorises data veridically (Figure 2c). It should also be noted that the above patterns are expected when marginalising over the individual data distribution given to a learner (i.e., a group effect). As different learners receive independent data drawn from the target languages, we expect some variation in the acquisition trajectory. Notably, we might expect the local distribution of data to encourage generalisations that are not globally representative of the data, i.e., learning traps [55] or conceptual garden pathing [56]. These local learning phenomena could result in nonlinear kinks or possibly even U-shaped learning (e.g., Figure 2d). We expect these kinks to be most notable early on in the trajectories when sampling variance is the greatest.

For our current purposes, we have provided a formal explanation for when a learning bias for iconicity is equivalent to a learning bias for informativity, i.e., when the learner’s hypotheses align with the speaker’s meanings. We believe this reinforces the arguments made by both Carr et al. [14] and Smith and Culbertson [15] that the learning biases previously attributed to informativity were actually due to iconicity. It should be noted that Carr et al. [14] did not directly argue for iconicity. Instead, they argued that an inductive bias for simplicity will push towards a compact (informative) category structure, where a compact category structure might arise from either communicative pressures or through “similarity”. They proceeded to argue that communicative pressures are unlikely (see [57]). Thus, we choose to interpret “similarity” as either compression over a structure in the world or a compressed mental representation of the structure in the world, both following Sims [38]’s treatment of similarity and thus, iconic. Further dovetailing with Carr et al. [14], we propose that this equivalence will be stronger when both hypotheses and meanings are highly compressed, as in the early periods of acquisition or highly speaker-efficient languages. To test our account, we simulated Beekhuizen and Stevenson [36]’s colour-term learner, plotted the acquisition trajectories in terms of communicative efficiency using Zaslavsky et al. [22]’s model of optimal colour compression, and compared the simulated acquisition trajectories against the predictions in Figure 2.

We can also use our analysis to check for an additional informativity bias in learning, as suggested by Fedzechkina et al. [32]. In the iterative learning experiments demonstrating a bias for informativity using grammatical distinctions, the hallmark of an informativity bias is that a learner’s acquisition trajectory contains a language that is more informative than the target language generating the data. Although the formal analysis outlined in this section does not predict such a pattern, the formal analysis assumes ideal learning. It might be the case that the assumptions of our implemented model are wrong or too simplified. For example, memorisation or hypotheses/meanings with very tight generalisation gradients might recreate the hallmark pattern (see Figure 2c; and Appendix A for further explanation). Similarly, chance sampling of the data might result in order effects that demonstrate this pattern (Figure 2d). Therefore, we also look to see if such a pattern occurs and aim to explain it in terms of the learner’s data distribution and inductive biases of the model.

## 3. Model Implementations

**Figure 3:**
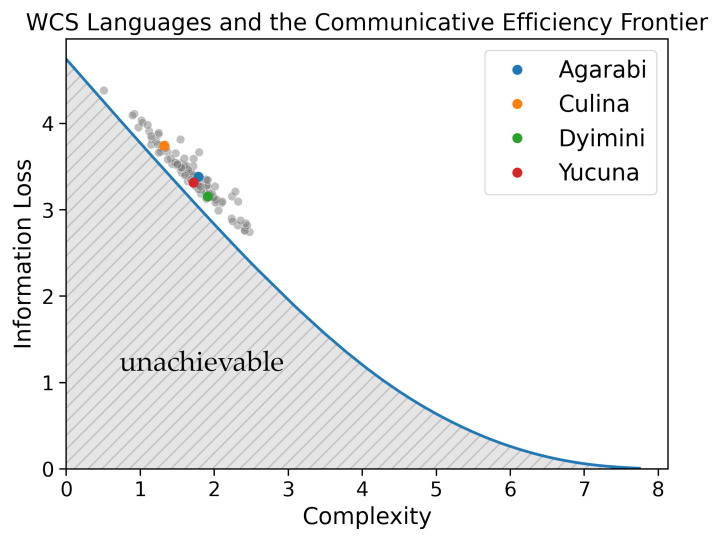
We plot the WCS languages, as scored in the communicative efficiency space, and highlight four typologically diverse languages: Agarabi, Culina, Dyimini, and Yucuna. Similar to Zaslavsky et al. [22], we find that natural languages cluster along the frontier.

To evaluate the communicative efficiency of languages along the acquisition trajectory, we re-implemented Zaslavsky et al. [22]’s model of colour-term compression. Using the World Colour Survey (WCS, [58]), Zaslavsky et al. [22] demonstrated that human languages embody the trade-off between complexity and information loss and achieve nearly optimal communicative efficiency as predicted by the information bottleneck principle. To validate our implementation, we replicate this analysis in Figure 3. The solid blue line reflects the ideal communicative-efficiency trade-off, i.e., the Information Bottleneck (IB) frontier, between complexity (*x*-axis) and information loss (*y*-axis). The area below the IB frontier reflects unachievable colour-naming systems. The attested colour-naming systems from the WCS are plotted as points. Replicating Zaslavsky et al. [22], we found that natural languages clustered along the frontier and that languages appear to trade off the complexity and information loss of the lexicon. Interestingly, natural languages lie close to the IB frontier at lower values of the trade-off parameter, reflecting a preference for simpler systems. Several researchers have attempted to explain the lack of attested languages distributed across the frontier, e.g., in terms of industrialisation [59] and communicative need [23,60].

**Figure 4:**
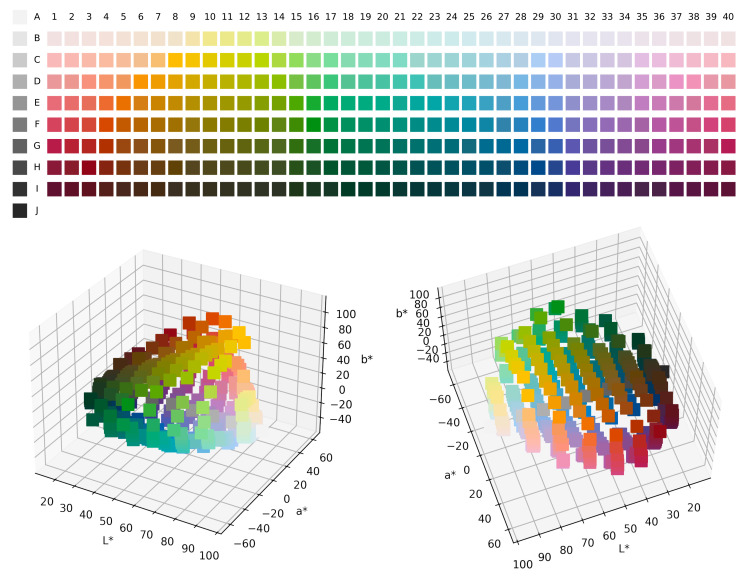
(**Top**) The World Colour Survey stimulus palette. Numbers above columns are the Munsell hues for the corresponding columns, and capital letters on the left indicate the lightness values of the corresponding rows. Each chip is a colour with maximum available saturation under the hue of its column and the lightness of its row. (**Bottom**) Distribution of the World Colour Survey stimuli in 3D CIELAB colour space.

As the goal of this work is to chart out the communicative efficiency of acquisition trajectories, we needed a formalisation of acquisition that operated over the same conceptual universe as the communication model, i.e., the Munsell colour chips from the WCS and CIELAB perceptual colour space, as illustrated in Figure 4. Furthermore, to draw interesting conclusions from this comparison, we were interested in using an acquisition model that could closely capture the biases that humans bring to learning. Although several model frameworks have been used to model colour-term acquisition [61,62,63,63], self-organising maps (SOM, [64]) have had the greatest success at learning complex attentional patterns over the perceptual space including the distinction between Russian light blue/dark blue [36,65]. More importantly, Beekhuizen and Stevenson [36] showed that SOMs are able to capture not only the topology of the input data thereby enabling colour discrimination in the model but also the colour-term acquisition patterns similar to those observed in children in particular over-extension errors. As such, we may expect that SOMs share various other properties with human colour-term acquisition. However, further cross-cultural research on children’s colour-term acquisition is sorely needed. For our simulations, we re-implemented the SOM learning model in Beekhuizen and Stevenson [36].

**Figure 5:**
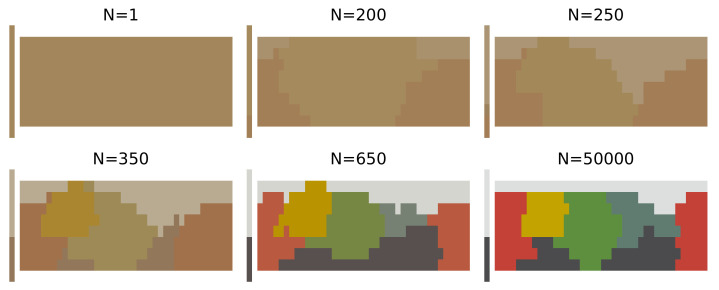
Visual development of the colour-naming distribution learned by a SOM for the attested language Agarabi. We plot the average colour of the most frequent colour term for all colour chips, commonly called a mode map.

We briefly outline the structure and training of SOMs as given by Beekhuizen and Stevenson [36] (for details and hyperparameter selection refer to Appendix B.1 and Beekhuizen and Stevenson [36]). A self-organising map consists of a square grid of cells, where each cell contains a vector that jointly represents the colour stimulus and colour-term features of the input data by concatenation. The training of a SOM proceeds iteratively (as shown in Figure 5). On each iteration, the SOM is presented with a new word-colour chip pair from the data set D and updates both the contents of the cell most similar to the data point while also affecting the most similar cell’s neighbourhood in some area determined by a hyperparameter σ. Over time as the SOM is exposed to more data, it eventually converges to a joint representation of the topology of the colour-stimulus space and the colour-term space, which we compare to the adult-like colour-naming distribution.

The inductive biases in the SOM, like all learning models, lie in the hypothesis space, the update rule used in acquisition, the starting state of the SOM, and the training regime used. The SOM’s hypothesis space is parameterised by $k^{2}$ number of *d*-dimensional vectors (one for each cell), where *k* is a hyperparameter and *d* is the dimensionality of the input. For the simulations presented in the text, we set k=13, which makes certain colour-naming systems (e.g., the language that gives a unique colour term to each of the 330 Munsell chips) unlearnable. As all attested languages have no more than 169 colour terms in their hypothesis space, this does not a priori hinder the acquisition of the attested languages. Nonetheless, the SOM’s hypothesis space places an inductive bias against the most complex colour systems. We confirmed the existence of this bias by running ablations of the SOM with k∈{1,⋯,13}. We observed a significant drop in performance and the loss of ability to acquire the attested naming distributions as *k* dropped below 6, although the acquisition trajectories looked largely unchanged for larger *k*. This effect was stronger for languages with a large number of colour terms such as Agarabi or Culina.

While the hypothesis space does not a priori rule out complex hypotheses, the SOM’s update rule places a stronger, additional bias against highly complex languages. Because the SOM updates not only the most closely matching cell but also those around that cell in the grid of cells, in a radius specified by σ, it encourages local similarity in the grid space, motivating the use of multiple exemplars for a colour term. This consequently makes a grid of 169 exemplars for 169 colour terms difficult to impossible to acquire. The strength of the inductive bias from the update rule is largely dependent on the starting value for σ and how we tune σ during learning; similar to Beekhuizen and Stevenson [61], we initialised σ to a relatively large value and then decreased σ by a small constant over the course of learning until a lower limit was reached. In theory, it may be possible to acquire highly complex languages with sufficiently large *k* if we are able to find a very specific setting and tuning for the radius σ; however, it is unknown whether such a regime would match children’s learning and in our simulations, we were not able to find such settings.

Further, the SOM’s update rule means that after one sample, the SOM would weakly classify all colours as the single colour of this sample, making extremely simple colour systems converge quickly. Other sorts of models might have the opposite inductive bias, where the initial states classify each colour chip separately, possibly through memorisation, and then learn to merge them, which would lead to very different patterns during acquisition. For example, this type of behaviour is seen in phoneme learning [66].

Finally, the distribution from which chips are selected during training would affect the acquisition patterns. Here, we followed Beekhuizen and Stevenson [36], who found that acquisition patterns are better matched by sampling from the joint distribution over words and colour chips P(W,C)=P(C|W)P(W) rather than by sampling uniformly. We calculated P(C|W) from the adult elicitation data (i.e., the WCS). In contrast to Beekhuizen and Stevenson [36] who used corpus frequencies, we also calculated P(W) from the elicitation data, as most languages in the WCS have no corpora available. This discrepancy in the choice of colour-term prior may have potentially biased our results, as the elicitation data were different from real-word usage; however, in Appendix B.2, we show that at least for English where data are available, this does not have a significant influence on our results.

### 3.1. Simulation Details

While Beekhuizen and Stevenson [36]’s simulations focused on English and Russian, we simulated the acquisition trajectory for each language in the WCS. As we have replicated that the languages in the WCS are communicatively optimal (Figure 3), we additionally simulate the acquisition trajectory for a permuted hypothetical variant of each language. This way, if we find that acquisition trajectories closely follow the communicative IB frontier, we can be confident that this is a property of the SOM, not a property of learning communicatively efficient adult-like languages. The hypothetical variants were calculated by permuting the colour chips along the hue dimension (i.e., horizontally on the mode maps) similar to Zaslavsky et al. [22]. Details of how the permutation was performed are in Appendix D. We selected the distance to shift such that the variant was the farthest permutation away from the communication frontier.

For each acquisition trajectory analysed, we simulated learning for 50 mutually independent learners (i.e., SOMs). Each SOM was trained for 50,000 iterations, though the models usually converged sooner. We averaged the results across all learners and display the standard deviation where possible. Following Beekhuizen and Stevenson [36], we assessed the learning trajectories of the SOMs based on three metrics: learning accuracy, complexity (i.e., the same as in the communication model), and the reconstructive error of the adult distribution. We assessed the convergence of the SOM using accuracy, the fraction of chips for which the colour term was predicted correctly. The ground truth for comparison was determined by the colour mode maps presented in the WCS. For more details on the assessment, refer to Appendix C.

### 3.2. Validating the Acquisition Model

**Figure 6:**
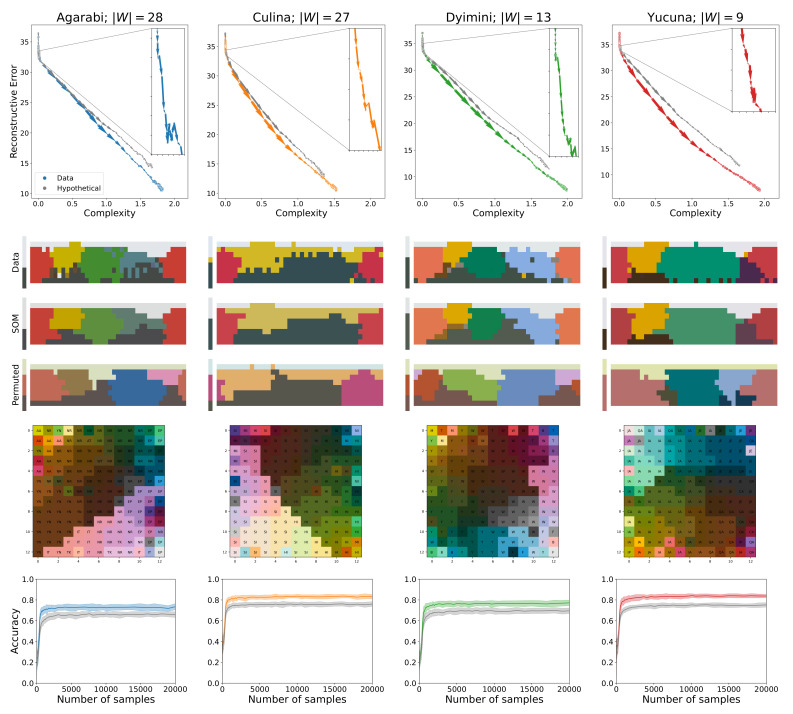
(**First row**) Averaged learning trajectories of the attested languages (shown in colour) plotted against the learning trajectories of their worst permuted variants (shown in grey). The arrows show the direction of learning as the number of samples increase. (**Second row**) Mode maps for the adult naming distribution (Data), the learned language (SOM), and the learned language from the worst permuted variant (Permuted). Images were generated with code by Zaslavsky et al. [22] using our own parameters. Mode map plotting code available at: https://github.com/nogazs/ib-color-naming (accessed 10 October 2022). (**Third row**) Learned colour space topology of the SOMs with the most likely colour term for each cell. The colour of a cell is given by the colour chip closest to the representation encoded in the cell. Plots were modelled after Beekhuizen and Stevenson [36]. (**Fourth row**) Plots of accuracy against the number of samples. We only plot the first 20,000 samples as the accuracy converges quickly. Shaded region shows standard deviation over 50 independent learners.

To illustrate how the SOM model acquires colour-naming distributions, we use four languages as examples from the WCS in this section and the rest of our results are presented in the Supplementary Materials. We use Agarabi (|W|=28), Culina (|W|=27), Dyimini (|W|=13), and Yucuna (|W|=9), ordered increasingly by the number of unique basic colour terms in each language. The latter three languages were selected following the choices of Zaslavsky et al. [22] to allow for an easier comparison of the results, whereas we opted to use Yucuna in place of English. The main results of the acquisition simulations for the four languages can be seen in Figure 6. The plots show the SOMs trained on samples from the adult naming distribution from the WCS and the permuted hypothetical variant.

Our simulated learners for all languages share a similar pattern. Each simulated learner starts with near-zero complexity and high information loss, as the early representations are close to the uniform distribution. Initially, the update weight σ is still large so for each new training sample, a large number of cells are affected in the SOM, which can result in large parts of the colour space changing their assigned colour terms from one sample to another. This effect can be seen on Figure 5 between N=200, N=250, and N=350 samples. This process also presents itself in a non-monotonically changing learning trajectory, as seen in the insets on the plots of the learning trajectories in Figure 6. After this initial warm-up phase, the SOM will have learned a basic representation of the topology of the input space. However, as can be seen in Figure 5, from the extremely low contrast among the colours, the naming distributions are still near-uniform across the colour chips, which makes individual colours difficult to distinguish, keeping the accuracy of the model low.

Nonetheless, at this point, the SOM’s information loss begins to linearly decrease while complexity increases. Smaller values of σ force the individual cells of the SOM to converge to a representation of a particular subspace of the colour space. This allows the model to better discriminate among the various colours, which results in a large increase in accuracy, as shown in the last row of the plots in Figure 6. The accuracy increases until the SOM converges, at which point new input data samples do not significantly change the topology of the acquired input space representation. The final accuracies reported here are lower than those reported in Beekhuizen and Stevenson [36] because we only used the perceptual semantic features. We were able to achieve similar numbers to Beekhuizen and Stevenson when we tested the SOMs using cross-linguistic semantic features. The topologies of the learned colour spaces are shown in the third row of plots in Figure 6, which shows that similar colours are located next to one another among the cells of the SOM. This aligns with the observations of Beekhuizen and Stevenson, who claimed that the SOM learns the topology of the input features, thereby enabling human-like colour discrimination in the model.

### 3.3. Acquisition of Communicatively Inefficient Languages

As the SOM is initialised to a language that is already communicatively efficient, namely the language with a single colour term, and then given inputs from a highly efficient language, it might not be surprising when the acquisition process in the SOM traces the IB frontier. As argued earlier, it is important to understand how the SOM behaves when applied to *communicatively inefficient* languages to ensure that our conclusions generalise to the properties of the model as opposed to the properties of communicatively efficient languages. Therefore, we performed additional analyses to address this question by training the SOM on communicatively inefficient languages and by initialising the SOM to a starting point that corresponds to some communicatively inefficient colour-naming system.

First, we note that the SOM has a strong inductive bias for learning convex colour-naming distributions due to its update rule. Training a SOM on just any randomly generated, non-convex, colour-naming distribution results in the SOM failing to acquire the language; therefore, we tested two ways of generating approximately convex languages that are still communicatively inefficient. First, we looked at the hypothetical permuted variants of each attested language, as mentioned in Section 3.1. Although the hypothetical variants are communicatively inefficient and convex as they are derived from the attested languages through shifting, they still lie relatively close to the IB frontier. To address this, we generated random convex partitions of the colour chip space, which resulted in communicatively inefficient languages. For the purpose of an easier comparison to an attested language, a randomly generated language had the same number of basic colour terms as the attested language. Finally, we also initialised the cells of the SOM randomly before training to place it farther away from the IB frontier. For the results shown in the text, we set each value in every cell to a value of 100 with a probability of 0.5; however, we also performed our analysis by uniformly sampling a number in the range [50, 100] and obtained very similar results.

**Figure 7:**
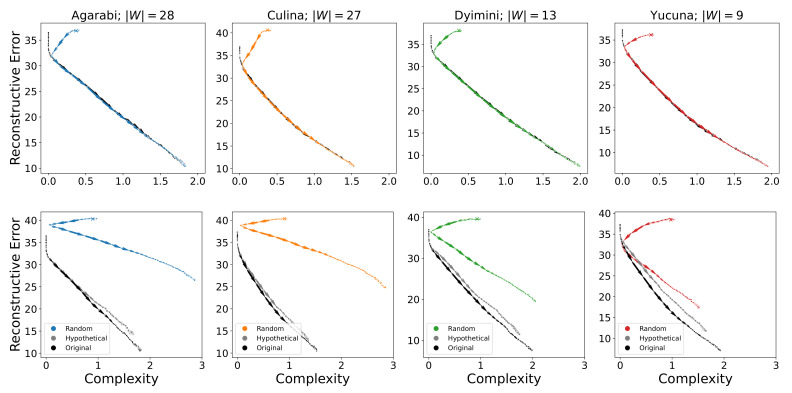
(**Top**) Average acquisition trajectories of attested languages when initialised to some random communicatively inefficient point. The starting position is marked with an *X*. (**Bottom**) Comparison of acquisition trajectories of an attested colour-naming system, its hypothetical permuted variant, and a randomly initialised, randomly generated language with the same number of colour terms (always shown in colour on the plot).

The acquisition trajectories of the attested languages with random initialisation are shown in the top row of Figure 7. Here, we observe the same behaviour across languages. For the first few hundred samples, the SOM orbits around its initial position. After this point, it closely approaches the trajectory of the learner that was initialised to the simplest optimal language. Both trajectories then follow the same path. The reason for this behaviour is due to the strong bias introduced by the update rule of the SOM. During the first few hundred samples, the update radius σ is still large so each new sample affects large parts of the acquired representation space of the SOM. This, in effect, overrides the random initialisation of the SOM with a near-uniform representation from which learning can proceed, as seen for the attested languages.

In addition, the average acquisition trajectories for sampling communicatively inefficient languages are shown in the bottom row of Figure 7. Here, we see that permuted variants are less easily acquired by the SOM than the actual adult naming distributions. Their learning trajectories consistently achieve worse complexity-reconstructive error trade-offs for each language, whereas their accuracy stays lower on average (as shown in Figure 6). That said, the permuted variant is still closely related to the attested language and follows a similar trajectory to the attested language.

However, the SOM is also able to learn randomly initialised, randomly generated inefficient languages despite the much worse complexity-reconstructive error trade-off. The large deviation compared to the attested languages is exacerbated by increasing the number of colour terms of the randomly generated language, as this increases the degrees of variability during the random language generation process. Insofar as we are acquiring colour-naming systems that were approximately convex, these results suggest that the inductive biases of the SOM are sufficient to drive the acquisition of colour-naming systems even when those differ greatly from human languages. Taken together, this suggests that the SOM is not biased to acquire communicatively efficient languages and the SOM’s inductive biases will overcome initialisation.

## 4. Assessing the Communicative Efficiency of Acquisition Trajectories

Having established that the SOM model can well approximate colour-naming systems, we can score the SOM models in terms of communicative efficiency to study their acquisition trajectories. Recall the several possible patterns for acquisition trajectories described in Section 2 and illustrated in Figure 2. For colour, we expect the acquisition trajectories to follow the communicative IB frontier (Figure 2a), suggesting that a learner’s hypothesis space (imbued with the SOM’s inductive biases) aligns with speakers’ meanings. In this case, following the argument in Section 2 and our reading of Carr et al. [14], optimal acquisition would be equivalent to optimal communication, and acquisition alone satisfies the criteria for the emergence of language, that is, *information loss* through regularisation and *informativity* through an iconic learning bias. However, we may find that learners’ hypotheses and speakers’ meanings do not perfectly align. In which case, we still expect languages to become more informative as complexity increases; however, we expect the pace of this increase to be non-monotonic (e.g., Figure 2b) as learners skip past communicatively optimal systems.

We are also interested in whether learners’ acquisition trajectories demonstrate the hallmark of an informativity bias in acquisition (e.g., as in [32]), that is, whether a learner’s acquisition trajectory contains a language that is more informative than the target language generating the data. As argued in Section 2, we might expect this pattern if the learning model’s inductive biases are wrong and learners simply memorise their input (Figure 2c) or if chance sampling of the data might result in order effects, i.e., kinks in the smoothness of the acquisition trajectory (as seen in the insets in Figure 6). If there was an inductive bias for informativity, it would weigh against the argument that language evolution is necessarily shaped by both language acquisition and dyadic communication [13,14,57].

**Figure 8:**
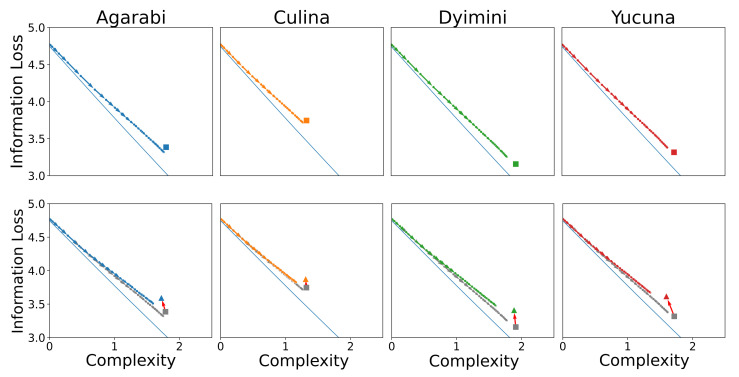
(**Top**) For the four attested languages, we map the averaged learning trajectories of the SOM to the communicative efficiency frontier; the attested languages are indicated by a square. (**Bottom**) We plot the averaged learning trajectories of the SOM for the permuted sub-optimal languages against the communicative efficiency frontier. The attested languages are indicated by a square and the sub-optimal languages are indicated by a triangle. The red arrows between the squares and the triangles highlight the impact of the convex transformation on the attested language. The original learning trajectories of the attested languages from the top row are shown in grey.

Without further ado, we score the learning trajectories of the SOM models in terms of their communicative complexity and information loss. In the top row of Figure 8, we plot the complexity and information loss as the SOM learns over time to show how the learning process maps to changes in communicative efficiency. The learning trajectory of the SOM approaches the attested language while staying close to the optimal communicative efficiency frontier. The SOM starts learning from the top left of the plot with a complexity of 0 and maximum information loss. This is because after just one sample seen, the SOM has only seen a single word and therefore its complexity and usefulness are low. As more samples are seen during training, the SOM learns additional words and therefore obtains a more useful lexicon (lower information loss) at the cost of higher complexity. Importantly, we do not see acquisition trajectories that include languages more efficient than the input language. The observed behaviour is in line with Carr et al. [14]’s hypothesis that communicative efficiency is explained by a learning pressure for simplicity acting over an iconic category structure, i.e., the learner’s hypothesis space aligns with speakers’ intended meanings. To additionally support this hypothesis, it should also be demonstrated that languages that are less iconic (e.g., those further away from the IB frontier) are more challenging to learn.

### 4.1. Ease of Acquisition of Efficient/Inefficient Languages

To determine whether languages closer to the communicative IB frontier are easier to learn than those farther away from it, we return to our simulations of *communicatively inefficient* colour systems. Recall that we generated less communicatively efficient versions of the WCS languages by *permuting* the assignments of chips to colours. Using permuted languages is arguably a fairer comparison than using random languages as non-convex languages have been argued to be difficult to learn [45], and in fact, proved unlearnable for the SOM model. The permuted languages are further from the frontier than the attested languages (*t*-test; t=9.45p<0.001, Cohen’s d=1.28). This is achieved primarily through an increase in information loss (t=5.58, p<0.001, Cohen’s d=0.76) and an insignificant decrease in the complexity (t=−1.23, p=0.22, Cohen’s d=−0.16).

**Figure 9:**
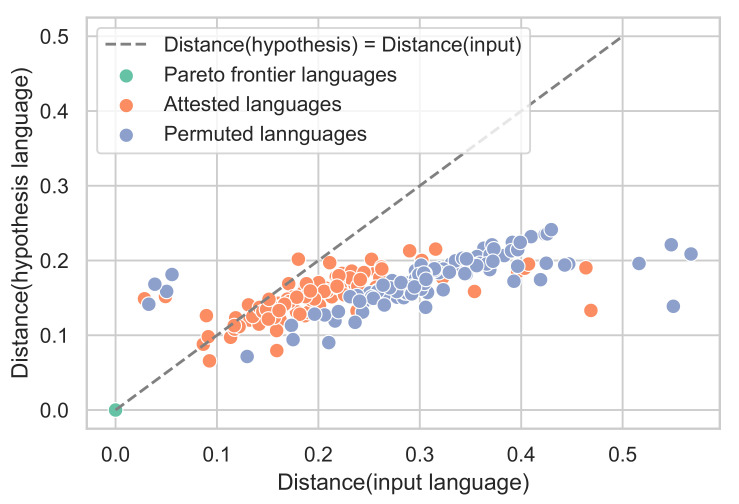
We find that after 50,000 steps, the colour system acquired by the SOM (the *hypothesis*) is closer to the communicative IB frontier than the input language for both the attested languages in the WCS (t=6.65, p<0.001, Cohen’s d=0.90) and the permuted languages (t=14.36, p<0.001, Cohen’s d=1.96). The effect is larger for permuted languages, which have larger starting distances from the frontier in the input. Languages on the frontier remain on the frontier (within the attested range of complexity). This points to the learned languages being “pulled toward” the frontier, i.e., an iconic learning effect.

Using these sub-optimal languages, we plot the resulting learning trajectories in the communicative efficiency space shown in the bottom row of Figure 8. The new points after the permutations are shown as triangles in Figure 8, and the red arrows map the transformation between an attested language and its permuted sub-optimal variant. Figure 8 shows the SOM learning trajectory tracking the IB frontier over time. We observe similar learning dynamics to those of the attested languages, where the SOM approaches the target language while staying close to the IB frontier. These languages are learned less accurately (t=7.63, p<0.001, Cohen’s d=−1.04) and take somewhat longer to converge (t=2.59, p=0.01, Cohen’s d=0.35). Notably, the hypotheses during the learning of permuted languages appear consistently close to the IB frontier, similar to the attested languages, despite the permuted languages themselves being further from the frontier. This observation is visualised at the end-point of learning in Figure 9.

We further studied the ability of the SOM to acquire colour-naming systems that were different from the attested languages using the randomly generated colour-naming systems from Section 3.3. Although we were only able to generate four of these languages, they are significantly further from the frontier than the attested (t=−11.36, p<0.001, Cohen’s d=−5.78) and permuted (t=−7.03, p<0.001, Cohen’s d=−3.58) languages. While the information loss is significantly lower for these languages than the permuted languages (t=8.55, p<0.001, Cohen’s d=4.35), their complexity is significantly higher (t=−9.66, p<0.001, Cohen’s d=−4.92), yielding an increased distance overall. Notably, this is the reverse of the relationship the permuted languages have with the attested languages, where complexity is lower and information loss is higher.

**Figure 10:**
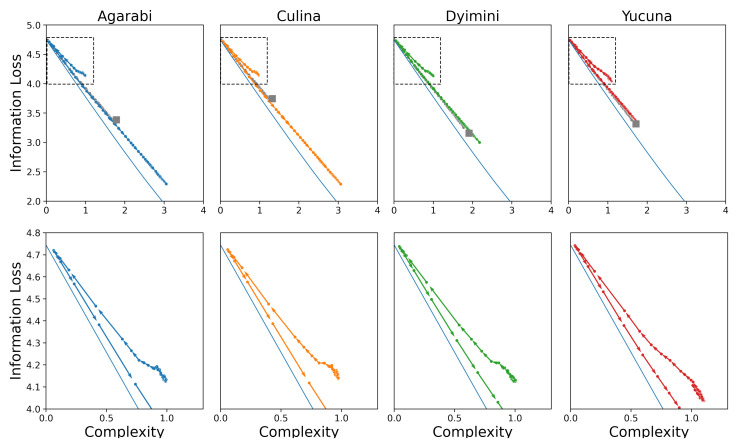
(**Top**) Communicative efficiency of acquisition trajectories of randomly generated languages. The randomly generated languages have the same number of basic colour terms as the corresponding attested language but are otherwise unrelated to one another. The grey squares and trajectories are the acquisition hypotheses of the attested language. The dashed squares mark the zoomed-in area shown in the bottom row of plots. (**Bottom**) The above acquisition hypotheses but zoomed-in on the information loss interval from 4 to 4.8, corresponding to the top left region of the above plots.

**Figure 11:**
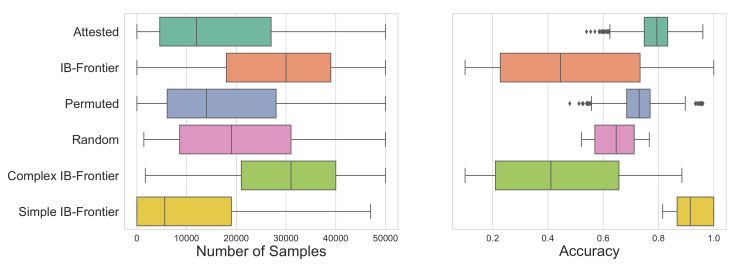
(**Left**) Distribution of how quickly the SOM converges to fit different types of languages. We find that attested languages converge slightly faster than sub-optimal ones, whereas the convergence of IB frontier languages depends on their complexities. Here, we also split the frontier languages into two groups, simple IB frontier languages (0.5<complexity<2.5) and complex IB frontier languages (2.5≤complexity). We find the differences in means between the three types of convergence distributions to be statistically significant (ps<0.01). (**Right**) Distribution of the accuracies for all types of languages. We observe that the accuracy for complex IB frontier languages to be significantly worse than for simple IB frontier languages. We also find a higher accuracy for the attested languages compared to their permuted variants.

The communicative efficiencies of the acquisition hypotheses are shown in Figure 10. As we have seen in Section 3.3, the SOM uses the first few hundred samples to “erase” the effects of random initialisation, effectively resetting its initial representation to a near-uniform distribution over the colour chips. From this point on, once again, the SOM approaches the target language while remaining close to the frontier. Accordingly, as shown in Figure 11, these languages are learned less accurately than the attested (t=5.01, p<0.001, Cohen’s d=2.57) and permuted (t=2.76, p=0.006, Cohen’s d=1.40) languages. However, we found no significant difference in the time to convergence.

### 4.2. Acquisition of Languages on the IB Frontier

Although the evidence in our current set of simulations points to acquisition accounting for the relative closeness of the attested colour systems to the communicative IB frontier (for a related argument see [45]), there is a measurable distance between attested systems and systems on the IB frontier (i.e., the communicatively optimal languages). The question then arises of whether this distance is due to the optimal systems being less *learnable* than the attested colour systems or to additional factors outside the scope of our analysis.

In order to address this, we sampled 42 languages uniformly spaced along the IB frontier and again applied the SOM learning algorithm. Following standard notation, we denote the trade-off between complexity and information loss on the IB frontier with β; a larger β means information loss is penalised more, resulting in more complex languages. When analysed together, this set of languages takes longer to converge and converges to lower final accuracies than either the attested languages or even the permuted languages (see Figure 11); however, this initial appearance is misleading as we find a strong linear association between the communicative complexity of a language and its acquisition ($R^{2}=0.95$).

**Figure 12:**
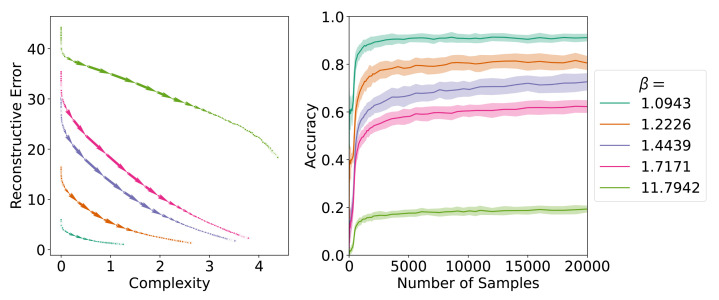
(**Left**) Learning trajectories for communicatively optimal languages. (**Right**) The evolution of accuracy across the number of samples seen for the optimal languages.

Considering only the languages on the frontier with complexities in the range of the attested languages, we then find that frontier languages converge slightly faster (t=7.42, p<0.001, Cohen’s d=−0.33) and have a much higher average accuracy (t=64.66, p<0.001, Cohen’s d=2.88) than the attested languages. We can see the different behaviours between frontier languages with low and high complexities reflected in the learning trajectories and the evolution of accuracy over the number of samples for five representative languages from the frontier in Figure 12.

Taken together, the finding that communicatively optimal languages within the range of attested complexity are more learnable than permuted languages suggests that acquisition may be able to explain the closeness of attested colour systems to the communicative efficiency frontier. Further, the portion of the communicative frontier that is unattested in real languages (with complexity >≈2.5) could be due to the low learnability of these languages at a single-learner level. This then has the additional implication that the fact that attested languages do *not* lie on the frontier is likely orthogonal to any questions about the role of learning biases and a communicative efficiency bias in the distribution of attested colour systems.

**Figure 13:**
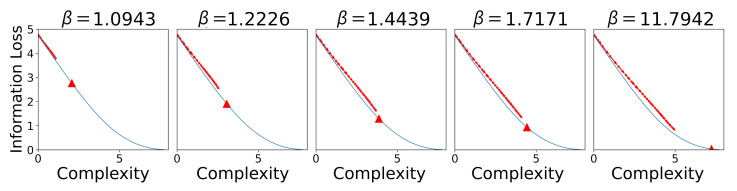
We plot the average SOM learning trajectories for different β values that correspond to different optimally communicative efficient languages along the frontier. The triangle in each plot represents the point on the frontier for the optimal language for a given β.

Similar to Section 4, we also score the SOMs as they learn the optimal languages on communicative efficiency in Figure 13. As before, we observe the learning trajectories of the SOM to track the communicative efficiency frontier. We observe that for high β values (e.g., β=11.7942), the SOM fails to approach the optimal point on the frontier during learning.

## 5. Discussion

Our first goal in this paper was to investigate if and how language evolution is shaped over two timescales: language acquisition and dyadic coordination. In order for a language to evolve, there needs to be a bias towards informativity and a loss of information (e.g., from a pressure to simplify). Empirical work in experimental semiotics has demonstrated that dyadic coordination provides a bias for informativity [10,17,18] and the shuffling of conversational partners affords information loss [19], thus satisfying the criteria for evolution without appealing to acquisition. At the same time, it is generally agreed that language acquisition has a bias for simplicity [9,67,68], resulting in information loss. Further, recent empirical work has made the case that language acquisition also has a bias towards informativity [32,34], thus satisfying the criteria for evolution without appealing to dyadic communication. Chicken-and-egg arguments aside, a language user must acquire language before they can use it; therefore, the requirements for language evolution would be minimally satisfied by acquisition. However, there is some uncertainty around the existence of informativity biases in learning [14,15].

Smith and Culbertson [15] and Carr et al. [14] suggested that simplicity and iconicity biases might provide the same functional pressure as an informativity bias. By formalising language acquisition and dyadic communications as rate-distortion models that operate over the same conceptual universe, we provided a formal explanation for how simplicity and iconicity pressures in learning serve the same function as an informativity bias: when a learner’s conceptual hypotheses about the world align with the meanings speakers want to convey, there is a formal equivalence between learning useful representations of the world and establishing a communicatively efficient language. If this is the case, acquisition trajectories should closely track the communicative IB frontier. We illustrated this with the colour-naming systems from the WCS, suggesting that learners’ hypotheses about colour terms align with speakers’ meanings for colour terms and acquisition is therefore sufficient to explain the evolution of colour-naming systems. This is perhaps unsurprising given the importance of incorporating human perceptual colour biases into computational models of the emergence of colour universals [22,63,69]. We refer to the structural alignment between learners’ hypotheses and speakers’ meanings as *iconic* both because we expect learners’ hypotheses about the world to directly reflect their experience of the universe [43] and because a speaker’s meanings are isomorphic to a learner’s hypotheses [43,44]. Therefore, to the extent that language is iconic, acquisition is sufficient for language evolution.

As our explanation does not preclude the existence of an informativity bias in learning [32], we also looked for a hallmark pattern: whether a learner’s acquisition trajectory contains a language that is more informative than the target language generating the data. In our simulations of colour acquisition, we did not observe such a pattern. To be fair, this pattern has been primarily observed when studying the acquisition of the grammatical components of language [32,33] as opposed to semantic categories. Therefore, our results do not rule out the possibility that such a bias exists.

Lastly, we note that not all communicatively efficient languages are easily learned by the acquisition model. Specifically, only optimal systems within the range of trade-off values observed in attested colour-naming systems are easily acquired. This suggests that learnability might be a good explanation for why attested languages do not span the entire range of possible trade-off values.

Of course, as with all formal analyses, our results are limited by the assumptions that we make. Specifically, our results are limited by the model formalisations, assumptions about communicative need and perceptual colour space, and the use of a SOM as a model of human learning. For example, we followed Zaslavsky et al. [22] in using a universal, capacity-achieving prior as a proxy for communicative need; however, communicative need is notoriously difficult to measure [23,24] and we know that communicative need systematically varies across communities [70]. Future work should seek to characterise the robustness of our results to assumptions about communicative need. As another example, although SOMs as learning models for colour terms showed better fit than other models, the cross-linguistic data the models were evaluated against were largely limited to over-extension data from a small set of languages. Future work is desperately needed to chart the empirical trajectory of children’s colour-naming systems.

Our account also leaves open several areas for future research including both the robustness of our results and phenomena for further explanation. While our account provides a potential explanation for the limited range of attested languages across the communicative frontier, we still do not know why attested languages lie *near* as opposed to *on* the IB frontier. It is likely we will need to model additional constraints on language to explain the gap. Although our account lays out the communicative efficiency and acquisition trajectory of linguistic systems, our account does not yet account for the topology of communicative systems across the frontier, i.e., both semantic universals and the recurrence of similar words/structures across solutions [45].

Additionally, it should be noted that there are potentially significant differences between the types of semantic domains, as suggested by the discrepancies between Carr et al. [14]’s and Carstensen et al. [34]’s results. Most formal work on learnability and/or communicative efficiency has focused on continuous, well-ordered (i.e., with known generalisation gradients) models of a semantic domain (i.e., hypotheses/meanings). To the extent that cognition is symbolic, we have barely scratched the surface in applying and drawing insights about the interactions between acquisition and efficient communication. It will be important to see how accounts of cultural/language evolution work for linguistic systems that do not reduce to closed-domain, well-ordered, continuous states of the world (e.g., kinship) and for domains where speakers’ meanings are not aligned to learners’ hypotheses (e.g., number).

In conclusion, our results suggest that to the extent that language is iconic and thus communicatively informative, language acquisition may be sufficient to explain language evolution. The diversity of attested systems is then governed by the amount of complexity invested in encoding the world (as argued by Zaslavsky et al. [22]). However, given that a large, interesting percentage of language is non-iconic [71], it is important to chart the acquisition, communicative, and goal-achievement constraints that shape the evolution of linguistic systems. It would not be surprising at all if language evolution over both the acquisition and dyadic coordination timescales (as argued by [12,13,14]) is required to explain the full complexity of natural language evolution. We offer our analysis approach, i.e., multiple rate-distortion problems constrained by shared assumptions, as one possible method for characterising these influences on cognitive technologies.

## Figures and Tables

**Figure A1:**
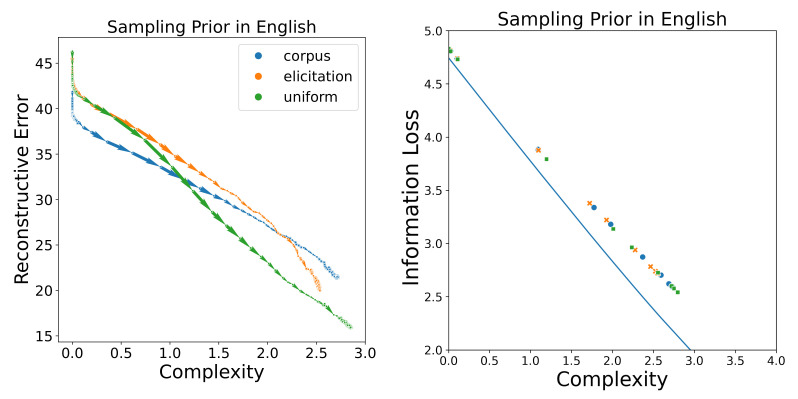
(**Left**) Acquisition trajectories with different sampling priors p(W) over the 11 basic colour terms of English. (**Right**) The communicative efficiency of selected acquisition hypotheses. We do not show entire trajectories as they overlap and make the individual trajectories indiscernible.

## Data Availability

The data for this work are from the World Colour Survey, which is available publicly online at https://www1.icsi.berkeley.edu/wcs/ accessed on 10 October 2022.

## Supplementary Materials

The following are available online at https://www.mdpi.com/article/10.3390/e24111542/s1, Supplementary Materials with model results for all languages attested in the World Colour Survey are available online at https://osf.io/9cnrm/ accessed on 9 May 2022.

## Appendix A. Theoretical Learning Algorithm: Memorisation

Memorisation, without generalisation, is an example of a theoretical learning algorithm that may show learners acquiring a more informative language than the language generating their input. In this situation, a learner initially starts with the degenerate language of one word to rule them all. As it memorises a few initial data points, the complexity of the language would increase much faster than the information loss decreases because the learner lacks a way to generalise/compress across world states until adjacent world states are observed. When adjacent world states are observed, the model can begin to compress them, resulting in a linear decrease in information loss as complexity increases until almost all adjacent states are observed. Notably, when there are multiple non-adjacent clusters that are encoded by the same word/concept in the target language but have not been merged by the learner due to missing observations, the learner’s languages are both more complex and more informative than the target language generating the learner’s data. Once these missing data points are observed by the memoriser, the system decreases in complexity and loses more information. Therefore, if learners do not have generalisation gradients (perhaps even if they have very small generalisation gradients), they may show a bias for informativity, as in Fedzechkina et al. [32], i.e., a learner’s acquisition trajectory contains a language that is more informative than the target language generating the data.

## Appendix B. Details of the Self-Organising Map

### Appendix B.1. Training Details

We re-implemented Beekhuizen and Stevenson [36]’s self-organising map (SOM) model. The SOM hypothesis space is parameterised by the $k^{2}$ number of *d*-dimensional vectors, where *k* is a hyperparameter and *d* is the dimensionality of the input. A training input to the SOM is the concatenation of term features and stimulus features. For a given language with |W| colour terms, a term is one-hot encoded and then weighted by a term-importance hyperparameter *a* so that the final term features took the form $f^{w}=\left[0\cdots a\cdots 0\right]$ and $|f^{w}\left|=|W\right|$. To encode the colour stimulus given as a colour chip c∈C, we could directly use the chip’s representation in the CIELAB space. However, to follow Beekhuizen and Stevenson’s implementation as closely as possible, we encode *c* as a vector $f^{c}$ so that $|f^{c}\left|=|C\right|$ and $f_{i}^{c}=||c-c_{i}\left|\right|$, that is, the stimulus feature vector represents a colour chip by its distances relative to the other chips. Given a data point, the SOM updates the contents of the cell in the hypothesis space most similar to the data point while also affecting the most similar cell’s neighbourhood in some area determined by a hyperparameter σ. We initially set σ=5.0. Over training, σ is linearly decreased by the learning rate hyperparameter α.

To derive a colour-naming distribution p(W|C) from the SOM’s hypothesis space for a given c∈C, we find the best matching cell in the hypothesis space by comparing the distances among only the stimulus features (i.e., term features are ignored for calculating the distance), which gives us the Best Matching Unit BMU(c). Using BMU(c) and given a word w∈W, we derive p(w|c) as

(A1) $$p\left(w|c\right)=\frac{value(w,BMU(c))}{\sum_{w^{\prime }}value\left(w^{\prime },BMU\left(c\right)\right)}.$$

The above value(w,BMU(c)) denotes the value for *w* in the term feature encoding of BMU(c).

We ran a grid search over the possible hyperparameters, checking all combinations of the following values:

k∈{7,9,11,13}α∈{0.01,0.025,0.075,0.1}a∈{0.01,0.025,0.075,0.1}.

Based on the results of the search, we decided to use k=13, α=0.1, and a=0.025 across all languages for the sake of simplicity and comparability, although this decision did cause most languages to reach accuracies that were lower than the best individual setting of the hyperparameters. Our setting of the hyperparameters resulted in most languages achieving at least 0.7 accuracy and qualitatively very similar mode maps to the adult-like data distribution. We also note that using the cross-linguistic semantic features of Beekhuizen and Stevenson [36] as the input representation gave higher accuracies; however, we did not use this representation because the communicative model operates on perceptual features. The choice of input representation and hyperparameters did not affect the pattern of acquisition trajectories.

### Appendix B.2. Simulation Details

For learning with data from the attested languages, we sample data points from the adult-like joint naming distribution $p_{l}\left(W,C\right)=p_{l}\left(C|W\right)p_{l}\left(W\right)$ given a language *l* in the WCS. The conditional distribution $p_{l}\left(C|W\right)$ and the prior $p_{l}\left(W\right)$ were estimated from the adult elicitation data from the WCS by calculating the normalised occurrence frequencies.

In contrast to Beekhuizen and Stevenson [36], our prior distribution $p_{l}\left(W\right)$ over colour terms is derived from elicitation data due to a lack of corpora for most languages in the WCS. This could have introduced potential biases to our sampling process as the elicitation data are significantly different from the real-world distributions of colour-term usage, which could be reflected in a corpus frequency-based prior. Although we could not address this issue for most languages due to the lack of data, we do demonstrate the effects of different sampling priors using English data, i.e., the corpus frequency-based prior for colour terms taken directly from Beekhuizen and Stevenson [36].

The averaged acquisition trajectories for corpus-based, elicitation-based, and uniform prior are shown in Figure A1. The closer the prior is to real-life usage (in decreasing order from corpus to elicitation to uniform), the easier it is for the SOM to learn an initial representation from fewer samples; however, it achieves a worse final complexity-reconstructive error trade-off. This is because the corpus-based prior prefers some basic colour terms over others, which means early on, the SOM learns the representation of the more frequent colour terms quicker but later on cannot adjust for new, unseen samples as the update rule’s radius σ would have significantly decreased by then. We also plotted the acquisition trajectories in Figure A1 according to their communicative efficiency, but we found that they were not affected by the choice of prior and all trajectories traced along the IB frontier.

When acquiring the *communicatively optimal* languages, our training data are sampled from the joint p(W,C), which is derived from the optimal naming system p(W|C) lying on the IB frontier and the universal capacity-achieving prior $p_{LI}\left(C\right)$. We trained all of the models on 50,000 samples, and for each attested language in the WCS, we individually simulated and averaged our metrics over 50 learners.

## Appendix C. Evaluation Metrics for the Learning Problem

We assessed the acquisition trajectories of the system based on three metrics: accuracy, information loss, and complexity.

### Appendix C.1. Accuracy and Convergence

Accuracy was used to evaluate the convergence of the SOM and how well the learned colour-naming model compared to the attested colour-naming model from the WCS. Our definition of accuracy followed Beekhuizen and Stevenson [36]. It was calculated as the fraction of chips for which the colour term was predicted correctly, where the ground truth for comparison was given by the colour mode maps presented in the WCS. For a particular colour chip c∈, the predicted colour term is given by

(A2) $$w_{c}{=}_{w\in W}p\left(w|c\right).$$

To decide whether a model had converged, we applied a simple early-stopping algorithm. For each acquisition trajectory, the model was deemed to have converged if it did not improve its accuracy by more than a threshold *t* in the next *p* (patience) steps. We set a threshold of t=1% and a patience of p=50 steps.

### Appendix C.2. Reconstructive Error

During acquisition, we needed to assess how well the current iteration of the model approximated the adult naming distribution $p_{l}\left(W,C\right)$ for a language *l* in the WCS by calculating the reconstructive error. We defined the reconstructive error for the acquisition problem as the KL-divergence between the joint distribution p(W,C) and the adult distribution $p_{l}\left(W,C\right)$:

(A3) $$D[p\left(W,C\right)||p_{l}\left(W,C\right)]=\sum_{w,c}p\left(w,c\right)\log\frac{p(w,c)}{p_{l}\left(w,c\right)}.$$

In order to calculate the joint distribution p(W,C)=p(W|C)p(C), we needed to define a prior over the colour chips p(C). Zaslavsky et al. [22] suggested invoking the principle of maximum entropy to arrive at a suitable prior p(C). However, for the space of colour chips, this would just be the uniform distribution. Therefore, Zaslavsky et al. instead calculated $p_{l}\left(C\right)$, which is the least informative prior for an attested language *l* and corresponding adult naming model $p_{l}\left(W|C\right)$ in the WCS. This was the prior that achieved maximum entropy over the colour chips while minimising the conditional uncertainty about the colour chips given the colour terms:

(A4) $$p_{l}\left(C\right){=}_{p^{\prime }\left(C\right)}H\left(C\right)-H\left(C|W\right).$$

This distribution can be efficiently found using the Blahut–Arimoto algorithm [72]. An issue with relying on $p_{l}\left(C\right)$ however is that many colour chips had zero probability under the prior, which is not a realistic property. Zaslavsky et al. [22] calculated a single universal colour prior $p_{LI}\left(C\right)$ by averaging over most $p_{l}\left(C\right)$, which solves this issue; however, we wanted to work with language-specific priors. Therefore, we chose to take a weighted combination of the language-specific prior $p_{l}\left(C\right)$ and the universal prior $p_{LI}\left(C\right)$ with weight parameter γ set to 0.9:

(A5) $$p\left(C\right)=\gamma \cdot p_{l}\left(C\right)+\left(1-\gamma \right)\cdot p_{LI}\left(C\right).$$

### Appendix C.3. Complexity

We defined complexity as the mutual information between the colour chips and colour terms I(W,C), where p(W) and p(C) are the marginal distributions of the joint p(W,C):

(A6) $$I\left(W;C\right)=\sum_{w,c}p\left(w,c\right)\log\frac{p(w,c)}{p(w)p(c)}.$$

## Appendix D. Convex Permutation of Chip Space

To study the learning of colour systems further from the frontier, we permuted the colour space of real languages to create permuted sub-optimal languages. These languages were entirely generated by permuting or shifting the 2D chip colour space. The 2D colour map, as seen in Figure 4, contained 8 rows of different Munsell values (lightness) and 41 columns (40 columns of Munsell hues and 1 of Achromatics). The Achromatic column contained 10 rows instead of 8, giving a total of 330 chips. For simplicity, we ignored the extra bottom and top rows of the Achromatic column in our permutation. We rolled the hues along their axes so that for a shift of s=1, the last column of hues became the first column and each column was shifted to the right by one. For each language in the WCS, we found the shift *s* in hues that maximised the Euclidean rate-distortion distance from the original point. This permutation was convex since the underlying colour structure remained the same but only the columns were rotated along the hue axis. For all permutations, the brightness of the modal chip for a colour term remained the same.
