# Supplementary material for: Communicative Efficiency or Iconic Learning: Do Acquisition and Communicative Pressures Interact to Shape Colour- Naming Systems?
Source: Entropy (Basel). 2022 Oct 26;24(11):1542. doi: 10.3390/e24111542 (PMC9689105; doi:10.3390/e24111542)
Supplement: Supplementary file 1 [file entropy-24-01542-s001.zip › SI.pdf]

Supplementary Materials: Communicative Efficiency or Iconic Learning: Do Acquisition and Communicative Pressures Interact to Shape Colour-Naming Systems?

Balint Gyevnar [1,†,*], Gautier Dagan [1,†], Coleman Haley [1,†], Shangmin Guo [1,†] and Frank Mollica [1]

The purpose of this Supplemental Material is to enumerate our results across all attested languages in the World Colour Survey [1].

1. Results for All Languages

We here present our results for every attested language in the World Colour Survey. Each subsection corresponds to a language in the WCS, and for each language we plot the following figures:

- The learning trajectory for the attested language and the learning trajectory for the most sub-optimal permuted variant.
- The learning trajectory mapped onto the communicative efficiency space.
- Mode maps for the adult-like colour naming system, the learnt colour naming system, and the *learnt* sub-optimal colour naming system.
- The colour topology of the learnt SOM model's cells with the corresponding most frequent colour term in each cell.
- The evolution of colour naming accuracy for the attested language and the most sub-optimal permuted variant.

1.1. *Abidji*; $|\mathcal{W}| = 9$

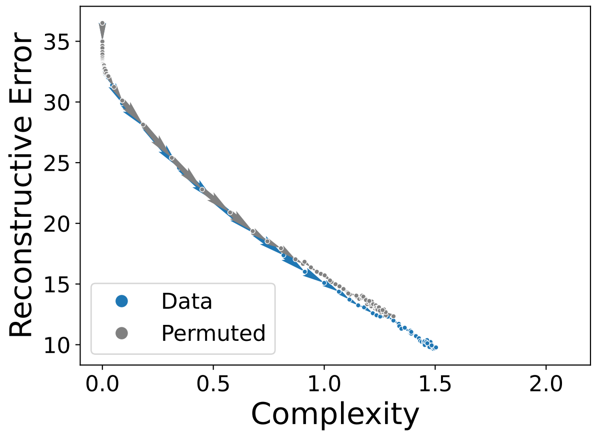

(**a**) Learning Space

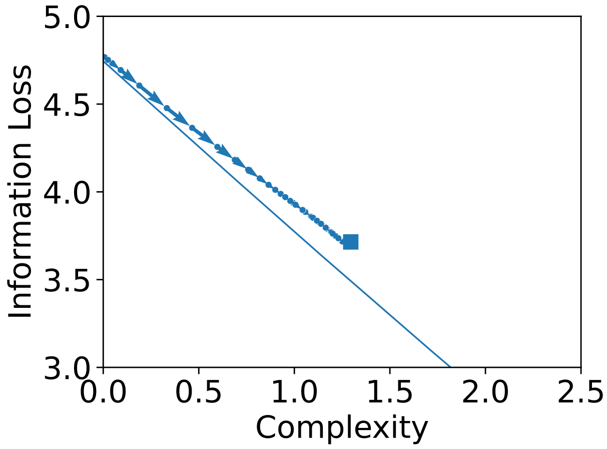

(**b**) Communicative Space

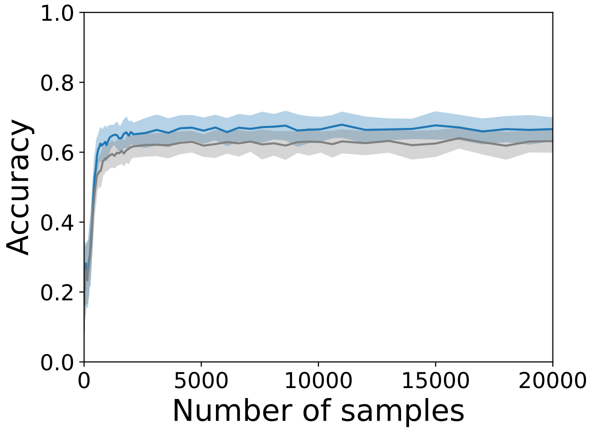

(**c**) Accuracy

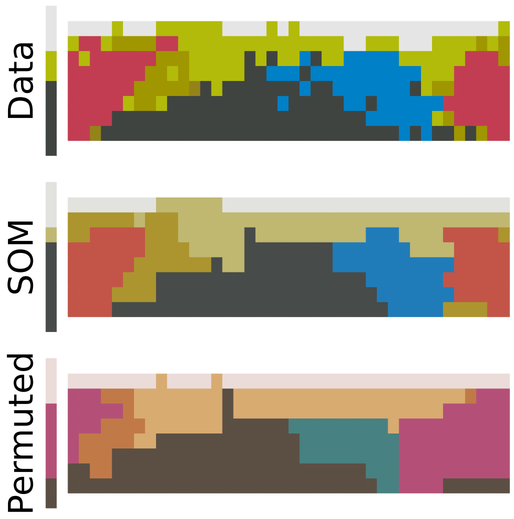

(**d**) Mode Maps

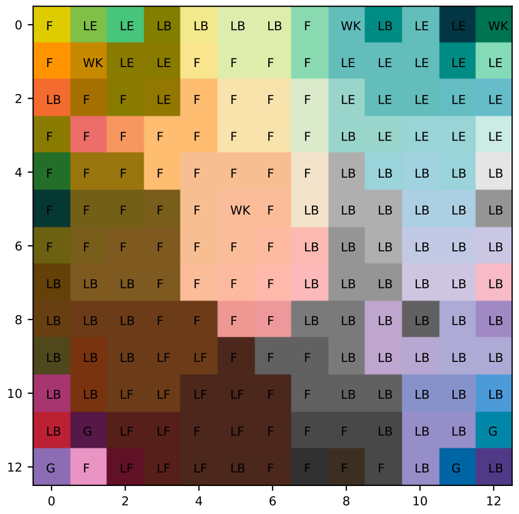

(**e**) Learnt SOM Cells

1.2. Agarabi; $|\mathcal{W}| = 28$

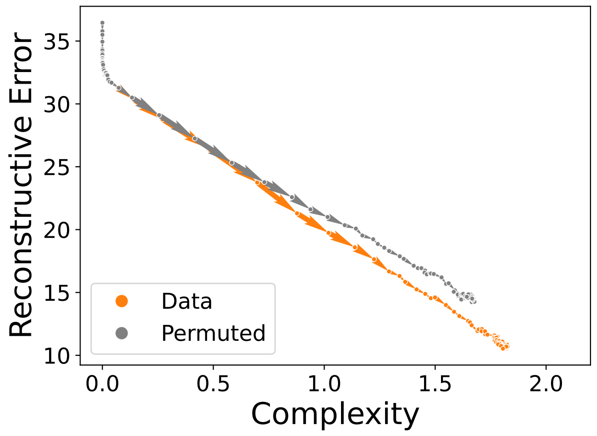

(**a**) Learning Space

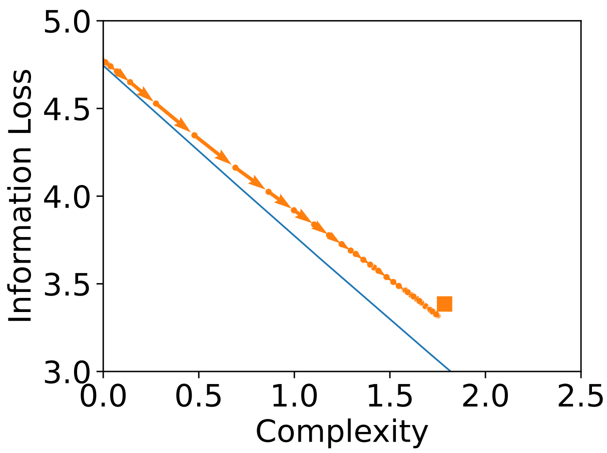

(**b**) Communicative Space

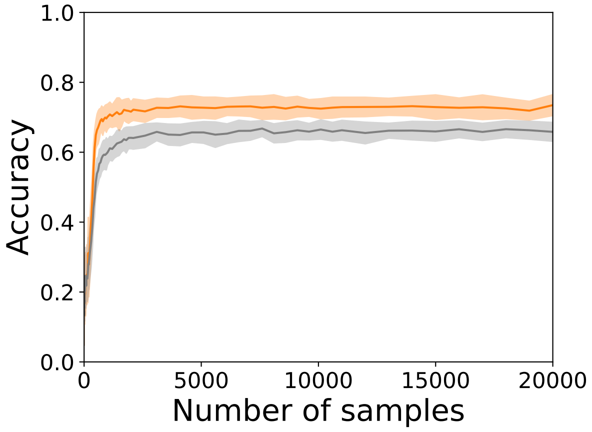

(**c**) Accuracy

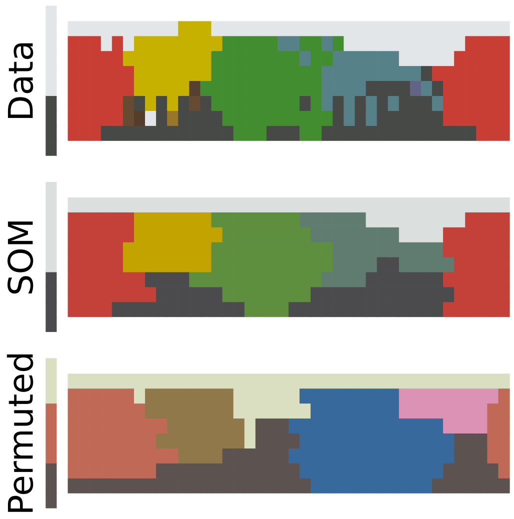

(**d**) Mode Maps

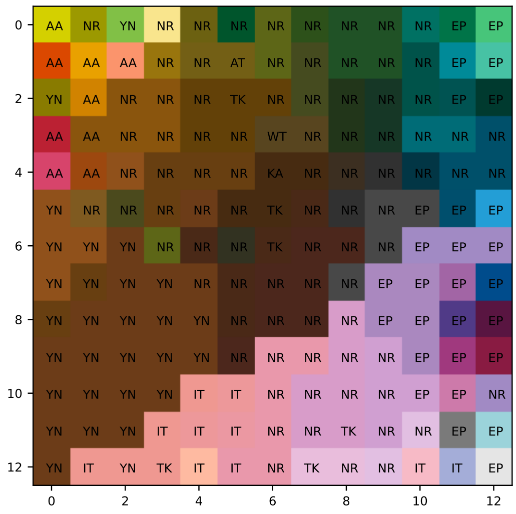

(**e**) Learnt SOM Cells

1.3. *Casiguran Agta*; $|\mathcal{W}| = 71$

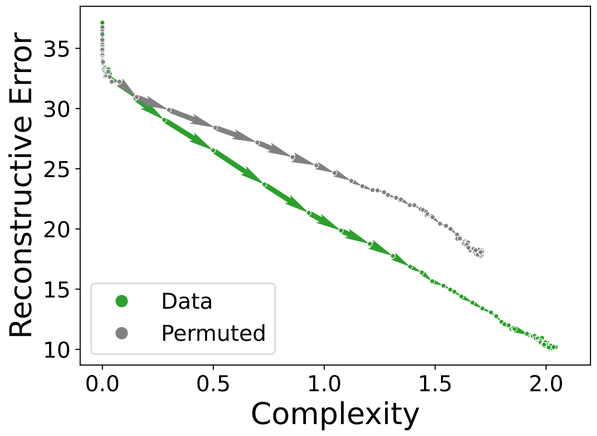

(**a**) Learning Space

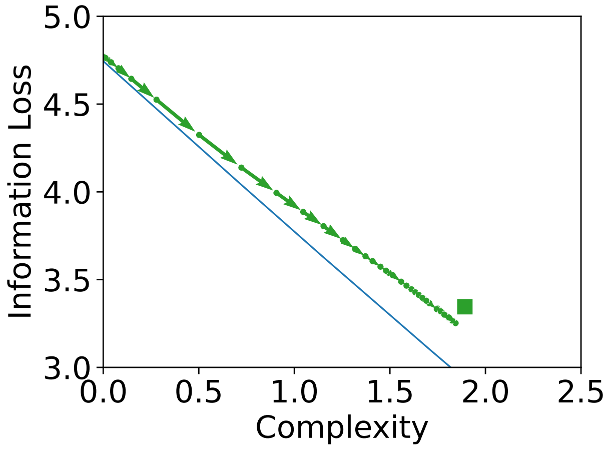

(**b**) Communicative Space

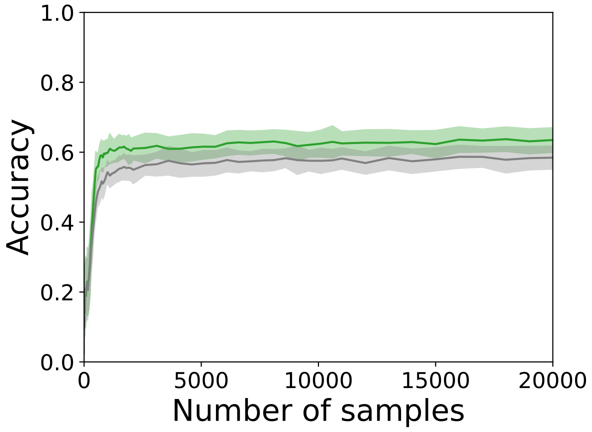

(**c**) Accuracy

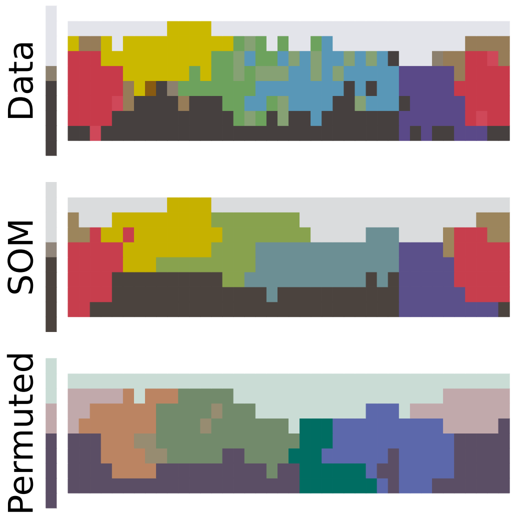

(**d**) Mode Maps

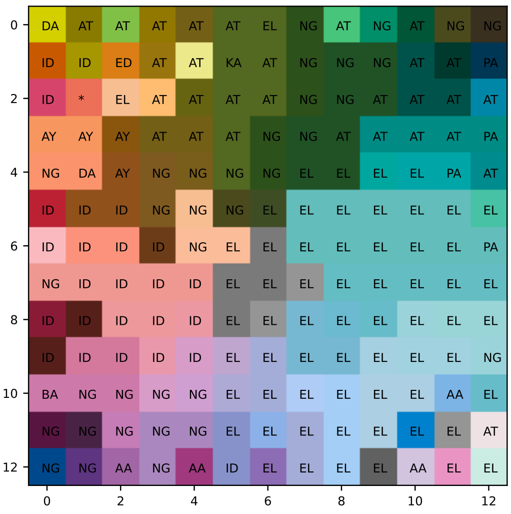

(**e**) Learnt SOM Cells

1.4. *Aguacateco*; $|\mathcal{W}| = 25$

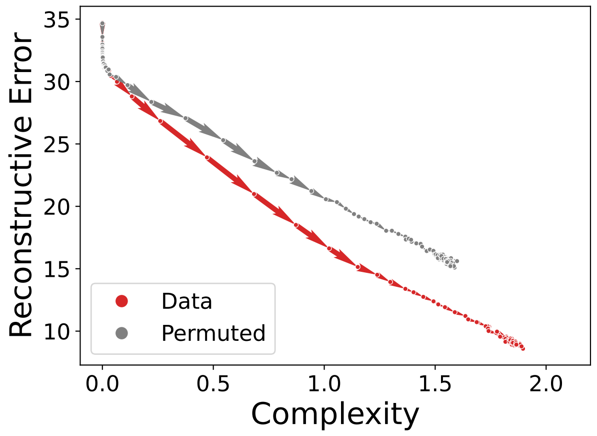

(**a**) Learning Space

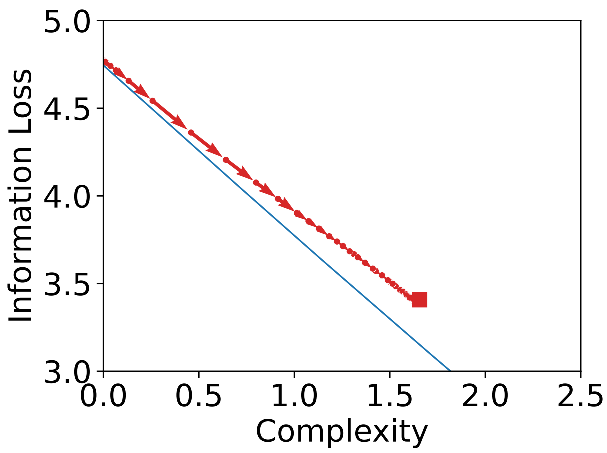

(**b**) Communicative Space

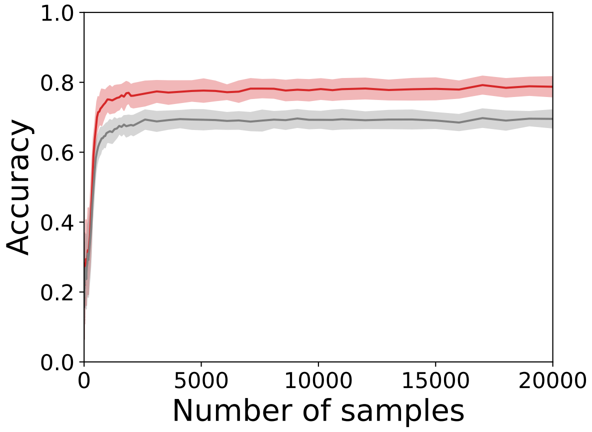

(**c**) Accuracy

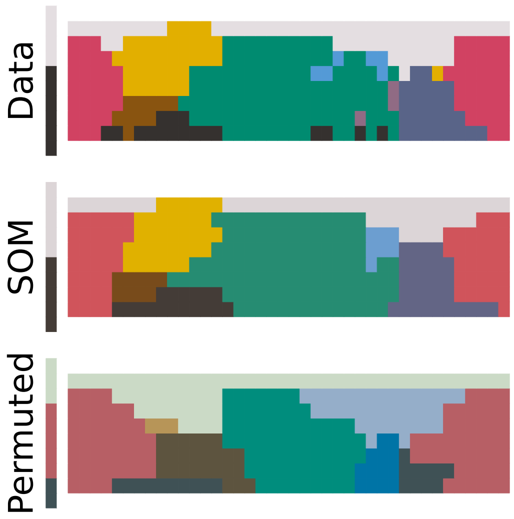

(**d**) Mode Maps

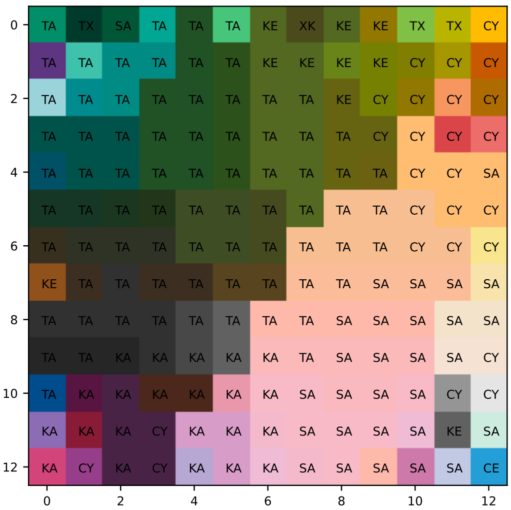

(**e**) Learnt SOM Cells

1.5. *Amarakaeri*; $|\mathcal{W}| = 9$

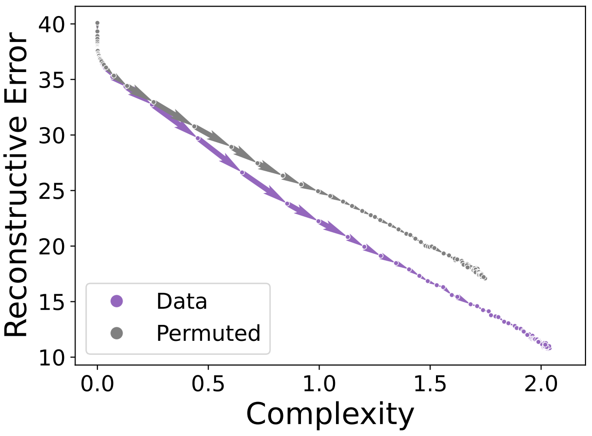

(**a**) Learning Space

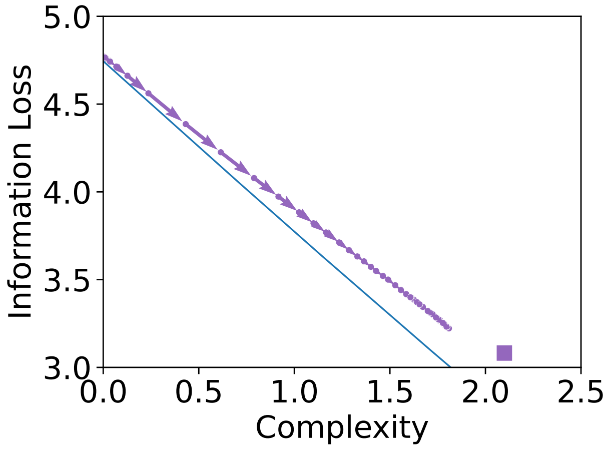

(**b**) Communicative Space

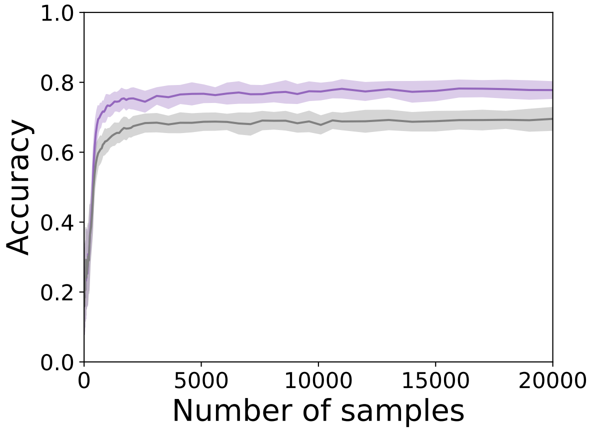

(**c**) Accuracy

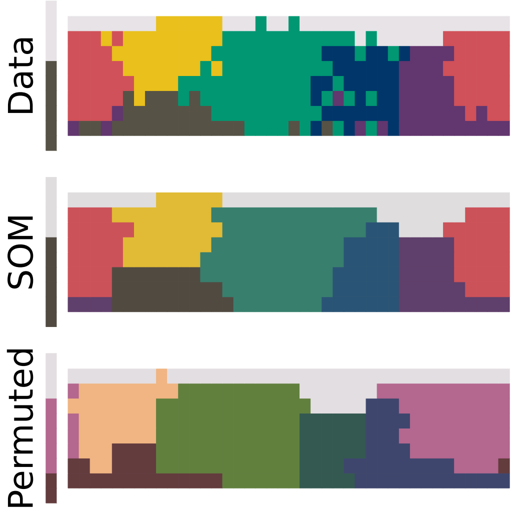

(**d**) Mode Maps

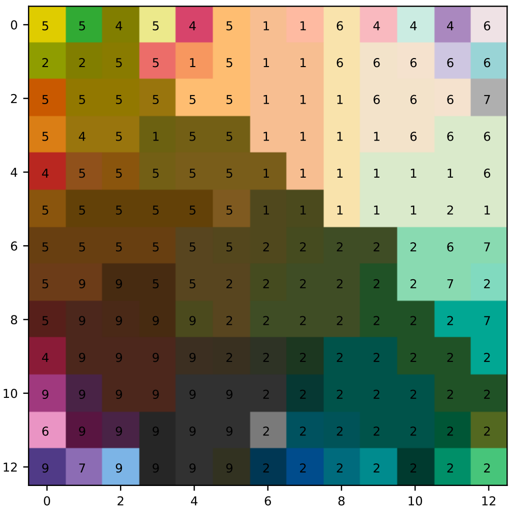

(**e**) Learnt SOM Cells

1.6. Ampeeli; $|\mathcal{W}| = 42$

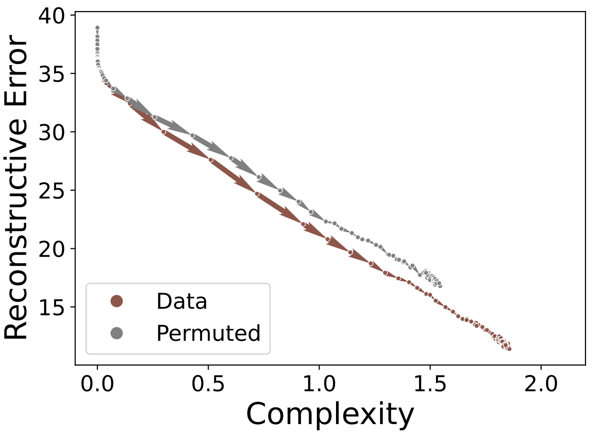

(**a**) Learning Space

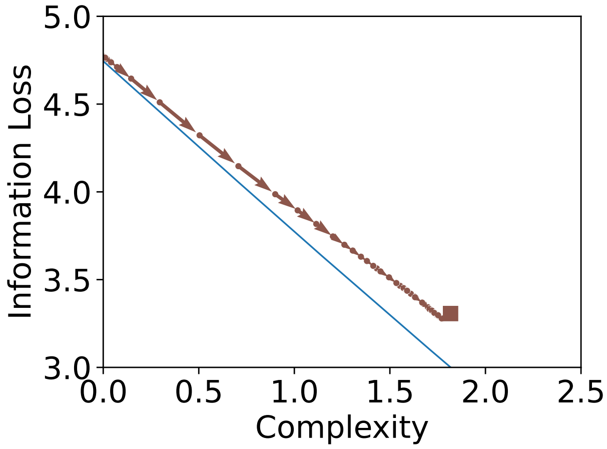

(**b**) Communicative Space

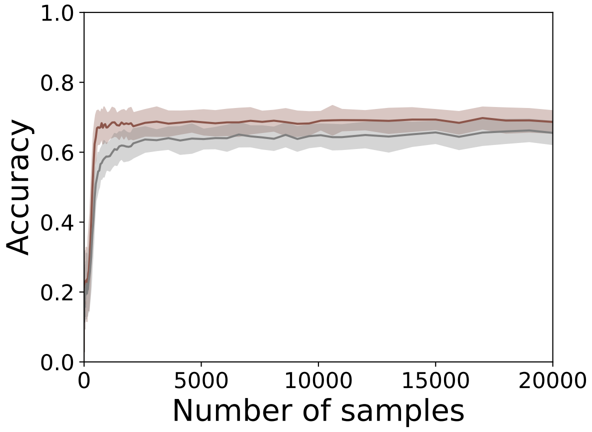

(**c**) Accuracy

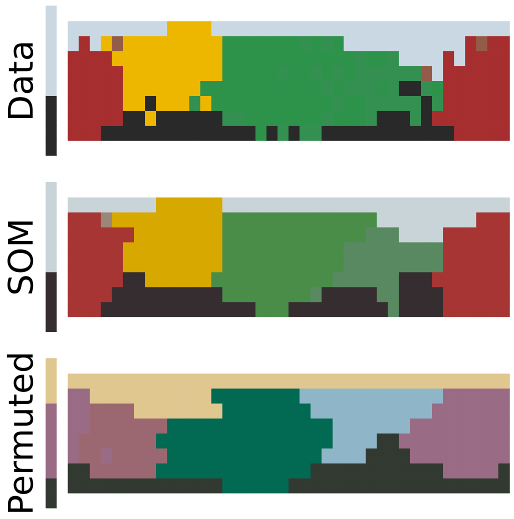

(**d**) Mode Maps

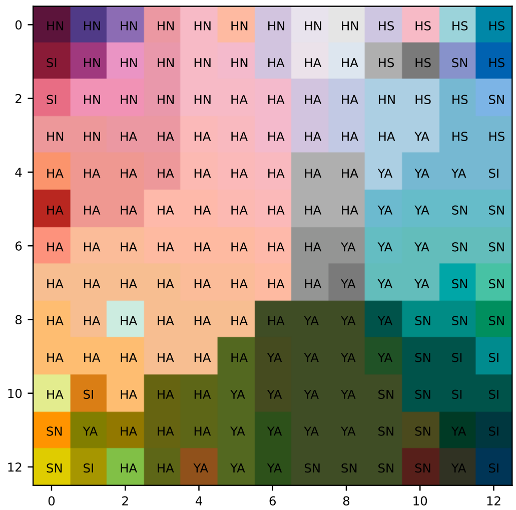

(**e**) Learnt SOM Cells

1.7. *Amuzgo*; $|\mathcal{W}| = 21$

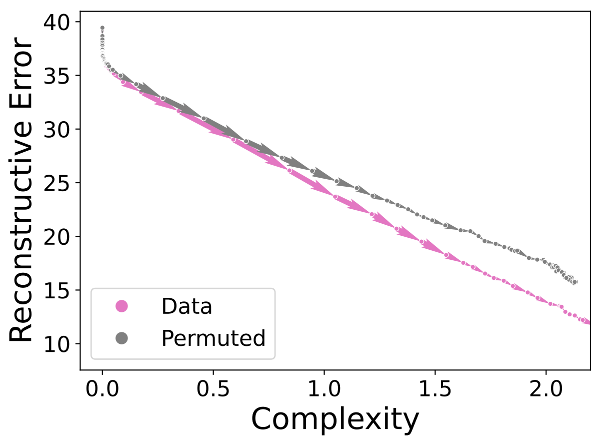

(**a**) Learning Space

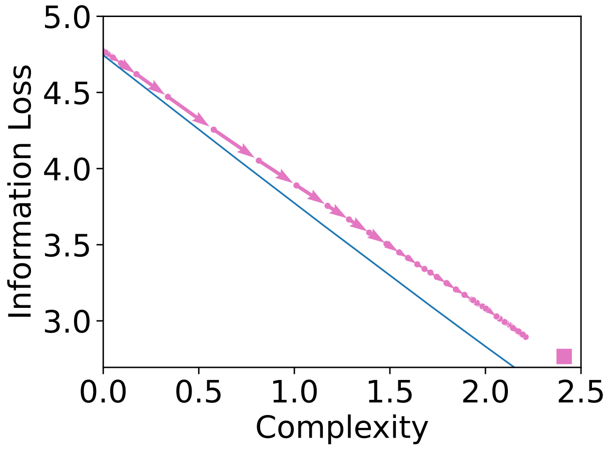

(**b**) Communicative Space

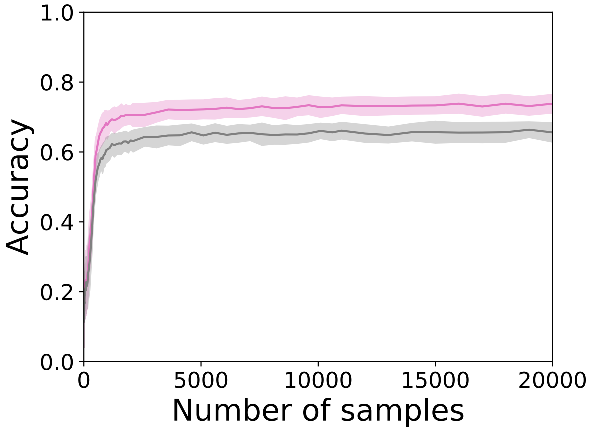

(**c**) Accuracy

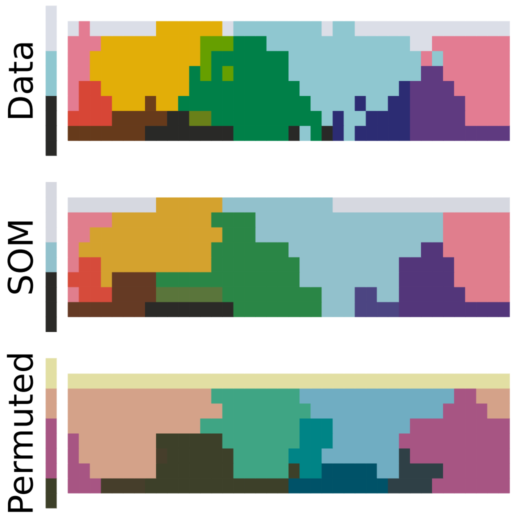

(**d**) Mode Maps

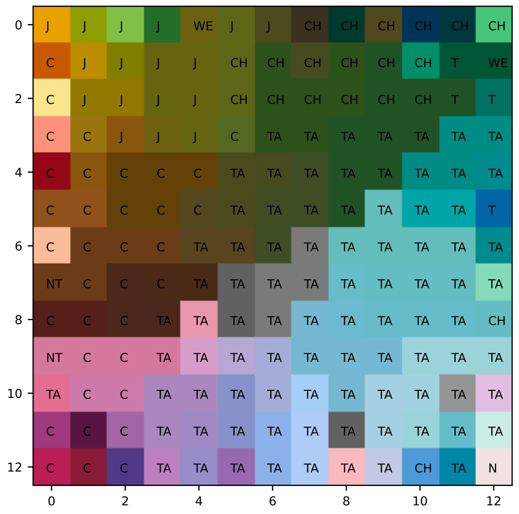

(**e**) Learnt SOM Cells

1.8. Angaatiha; $|\mathcal{W}| = 16$

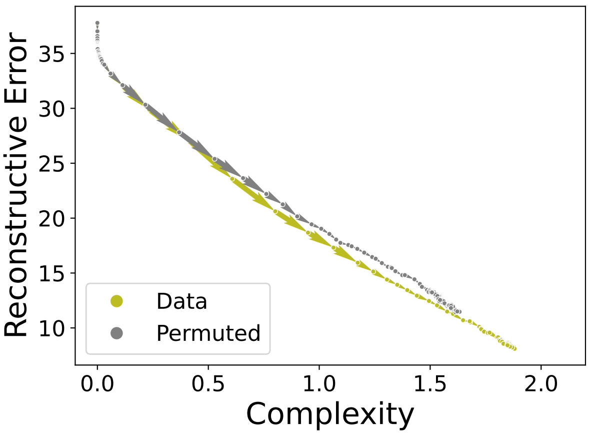

(**a**) Learning Space

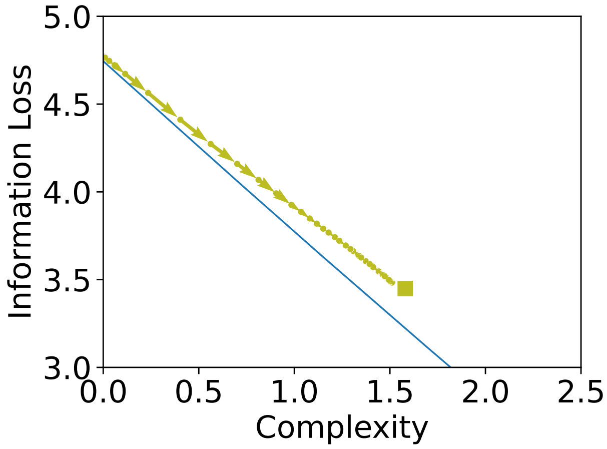

(**b**) Communicative Space

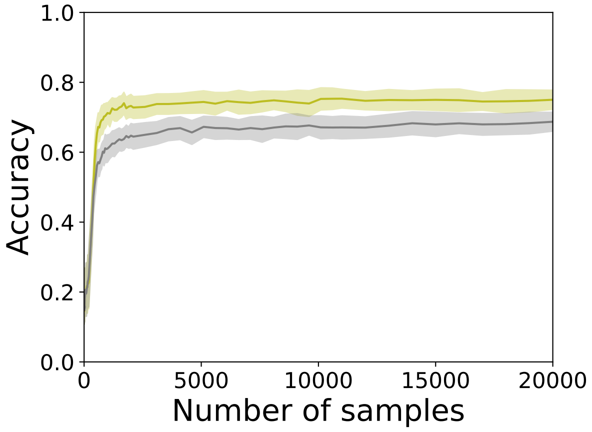

(**c**) Accuracy

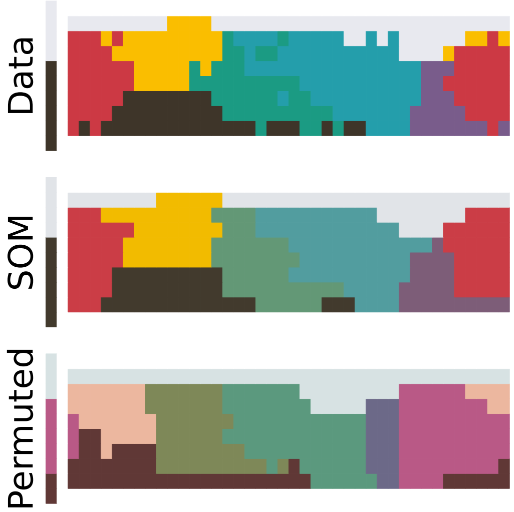

(**d**) Mode Maps

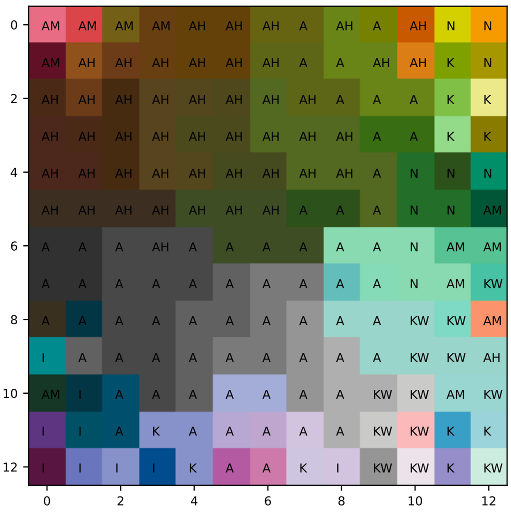

(**e**) Learnt SOM Cells

1.9. Apinaye; $|\mathcal{W}| = 13$

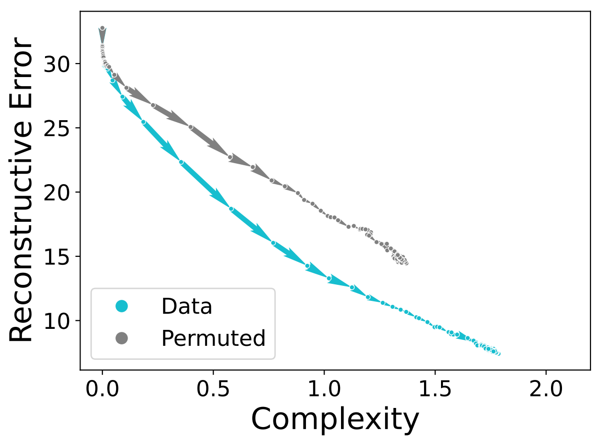

(**a**) Learning Space

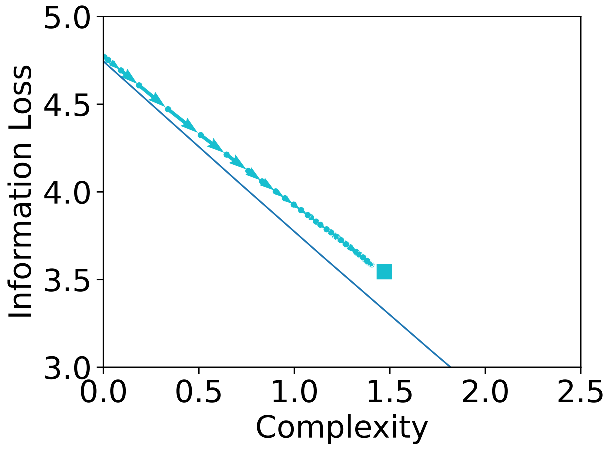

(**b**) Communicative Space

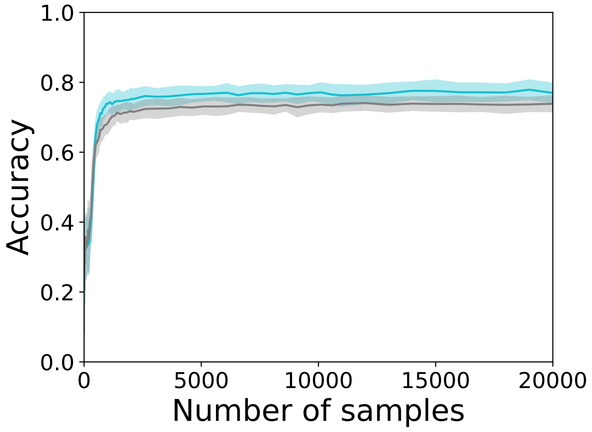

(**c**) Accuracy

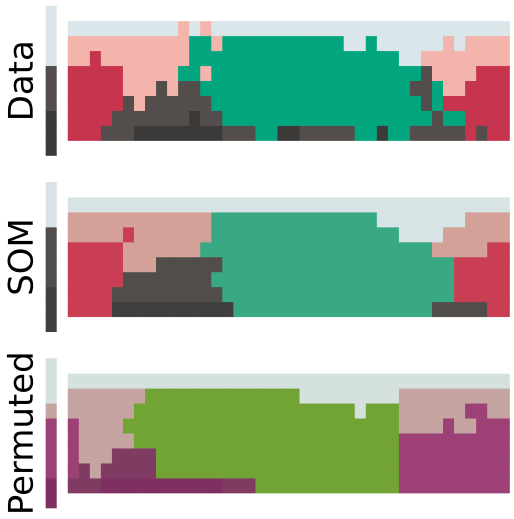

(**d**) Mode Maps

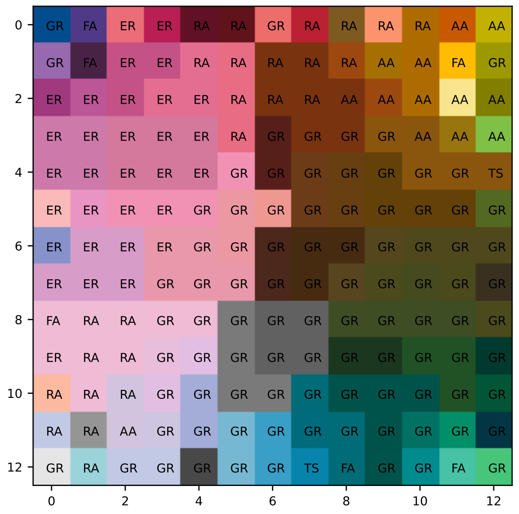

(**e**) Learnt SOM Cells

1.10. Arabela; $|\mathcal{W}| = 18$

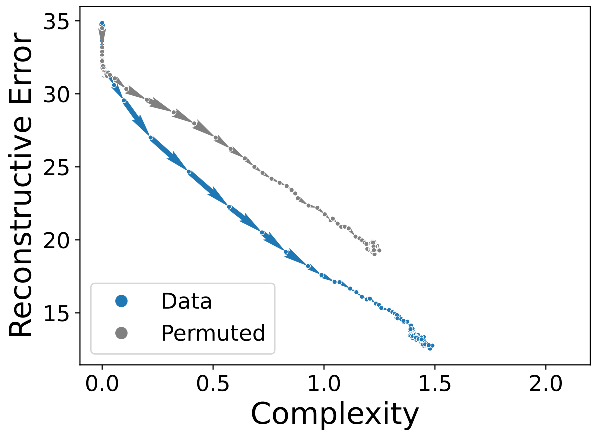

(**a**) Learning Space

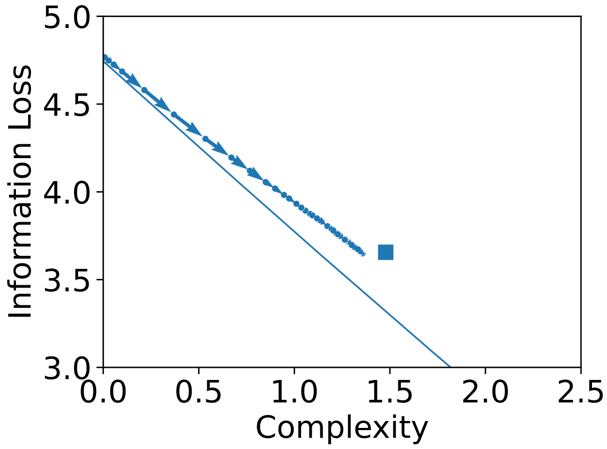

(**b**) Communicative Space

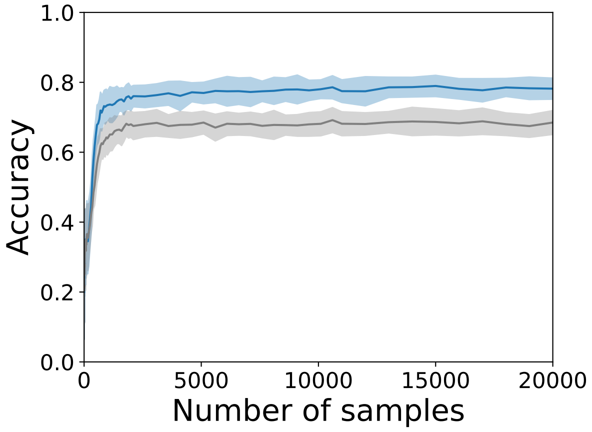

(**c**) Accuracy

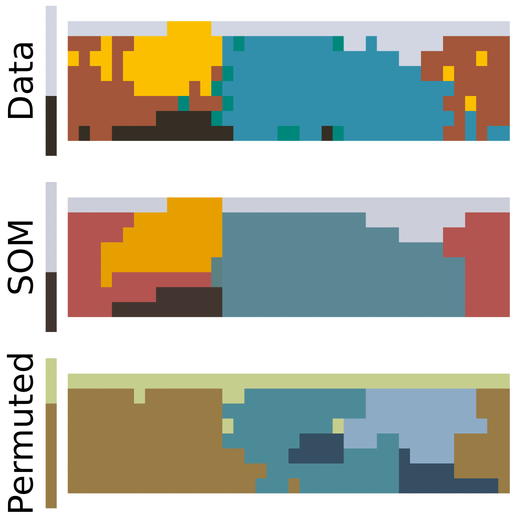

(**d**) Mode Maps

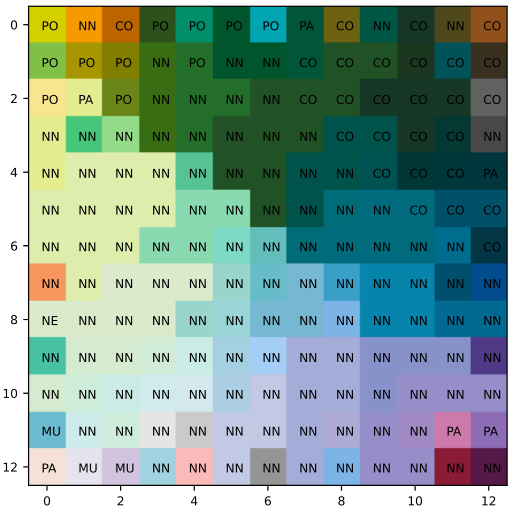

(**e**) Learnt SOM Cells

1.11. Bahinemo; $|\mathcal{W}| = 11$

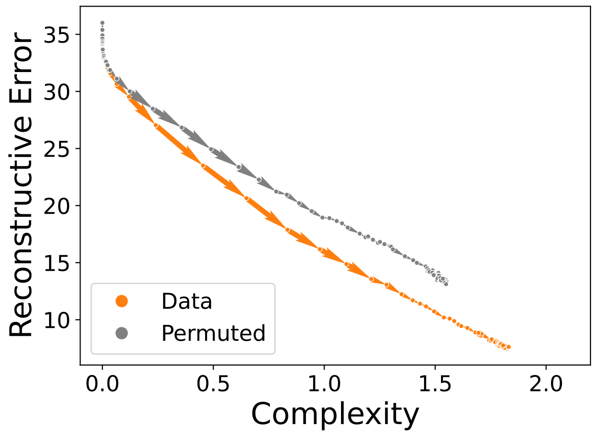

(**a**) Learning Space

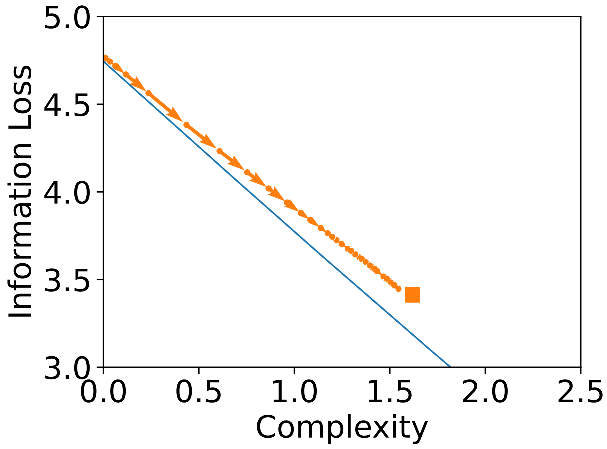

(**b**) Communicative Space

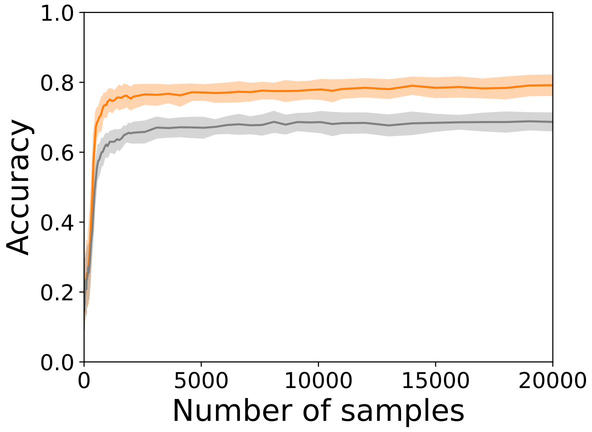

(**c**) Accuracy

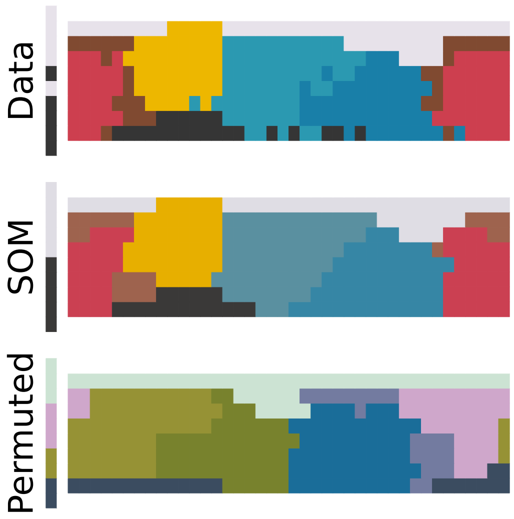

(**d**) Mode Maps

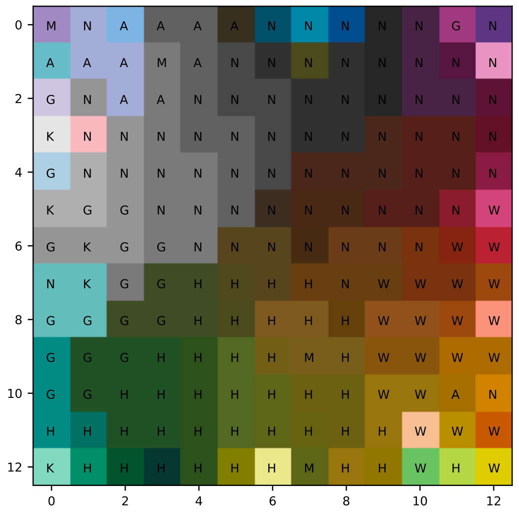

(**e**) Learnt SOM Cells

1.12. Bauzi; $|\mathcal{W}| = 7$

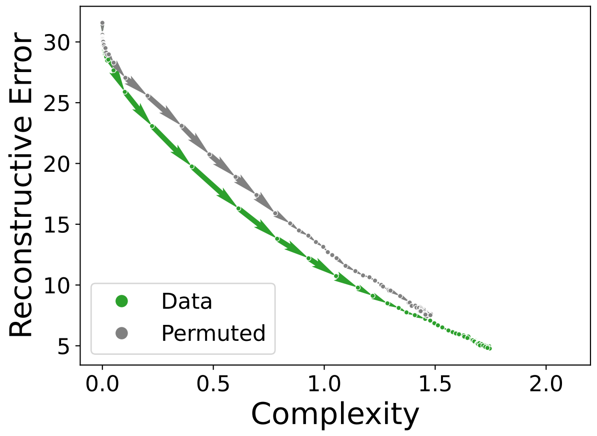

(**a**) Learning Space

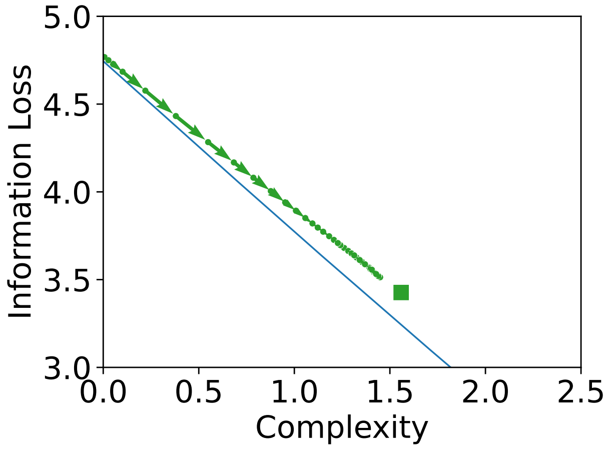

(**b**) Communicative Space

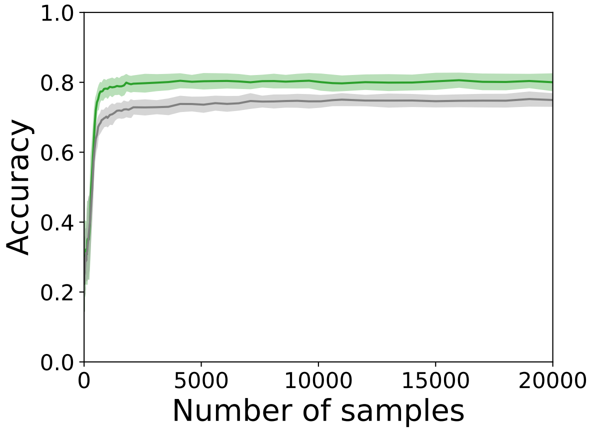

(**c**) Accuracy

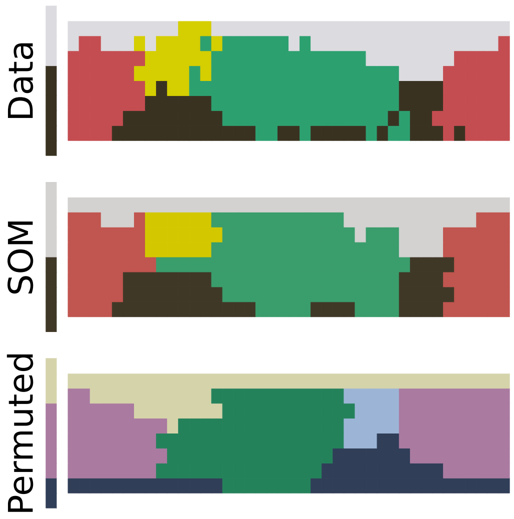

(**d**) Mode Maps

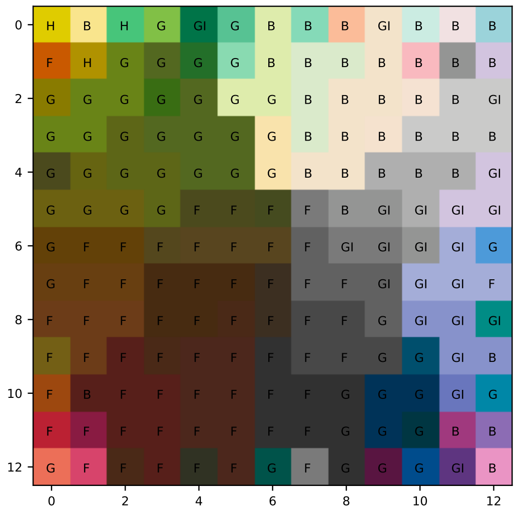

(**e**) Learnt SOM Cells

1.13. Berik; $|\mathcal{W}| = 30$

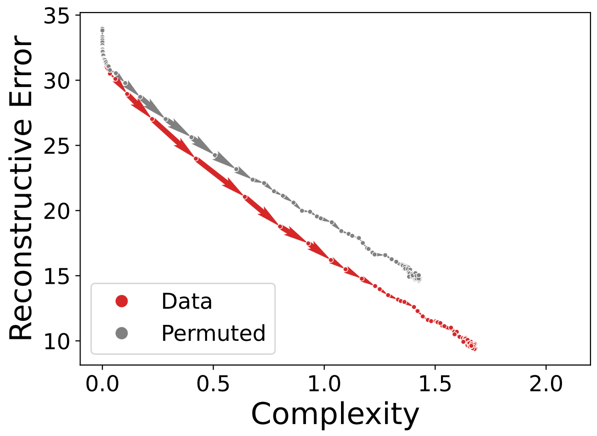

(**a**) Learning Space

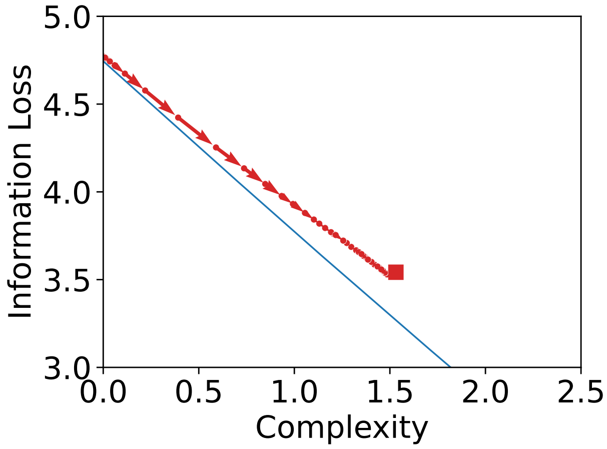

(**b**) Communicative Space

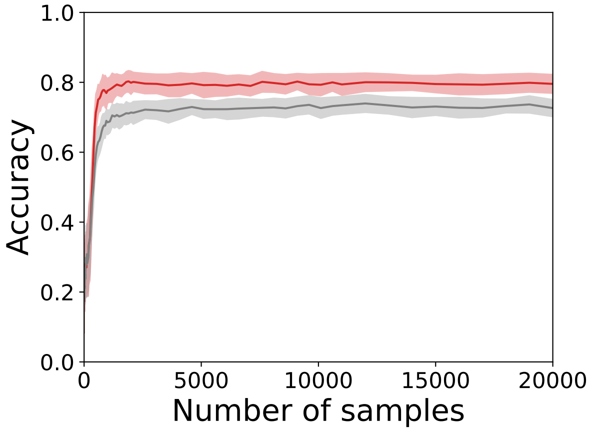

(**c**) Accuracy

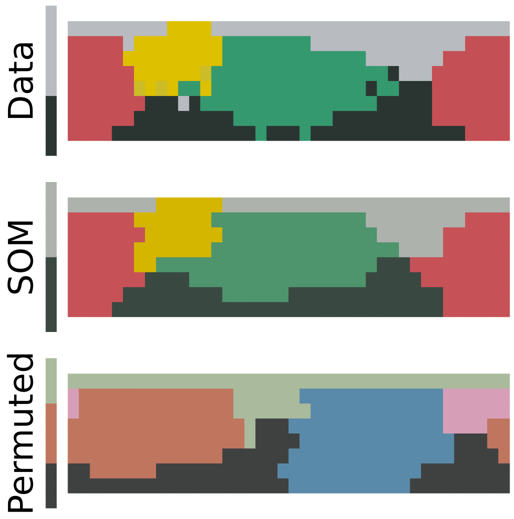

(**d**) Mode Maps

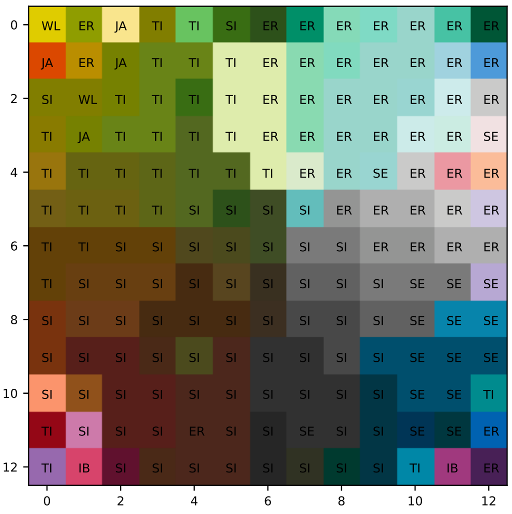

(**e**) Learnt SOM Cells

1.14. Bete; $|\mathcal{W}| = 23$

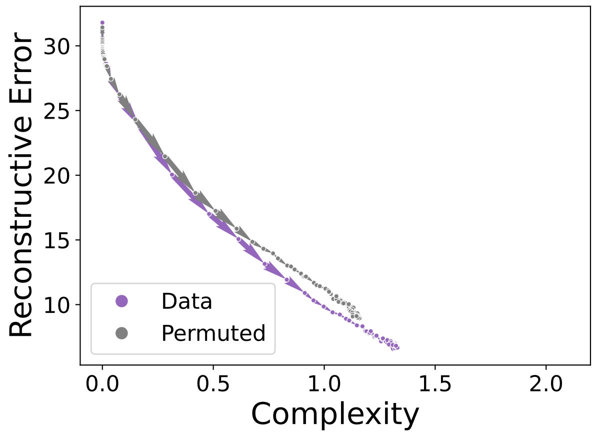

(**a**) Learning Space

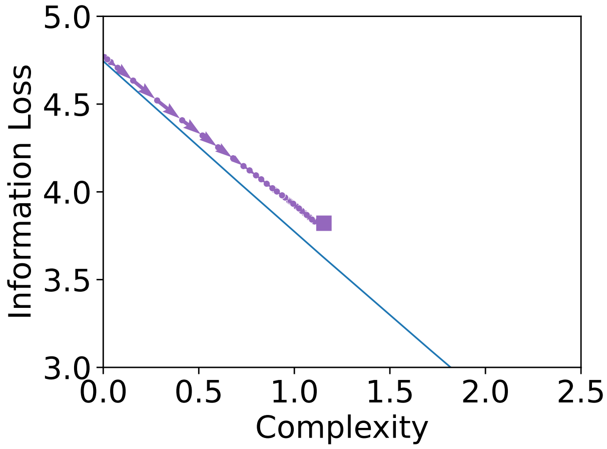

(**b**) Communicative Space

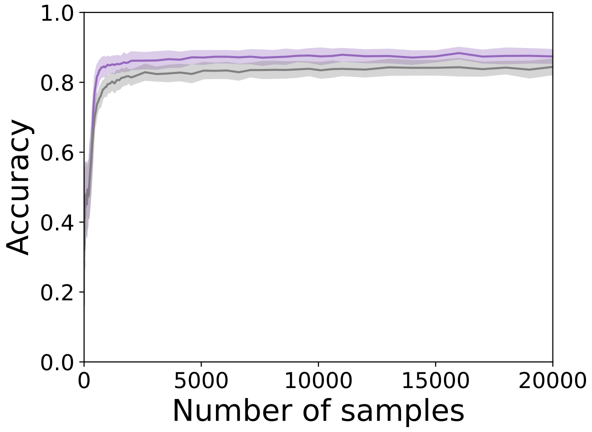

(**c**) Accuracy

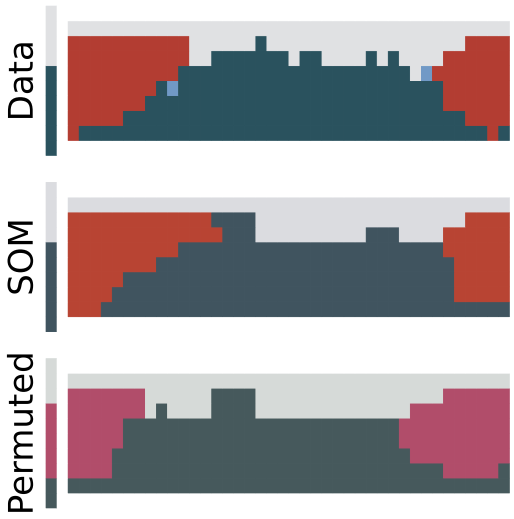

(**d**) Mode Maps

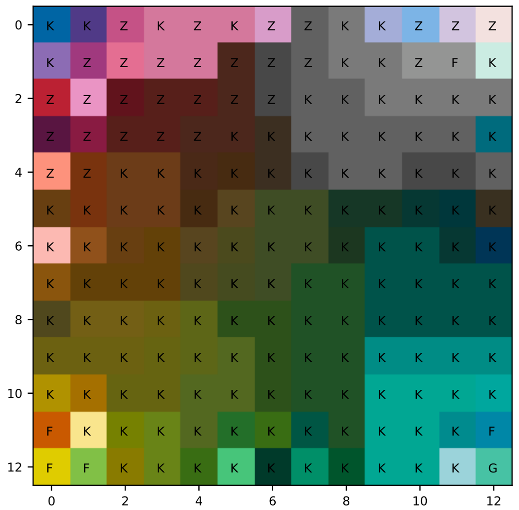

(**e**) Learnt SOM Cells

1.15. Bhili; $|\mathcal{W}| = 15$

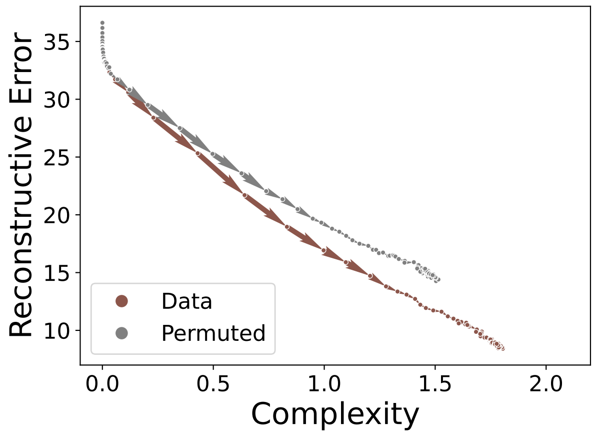

(**a**) Learning Space

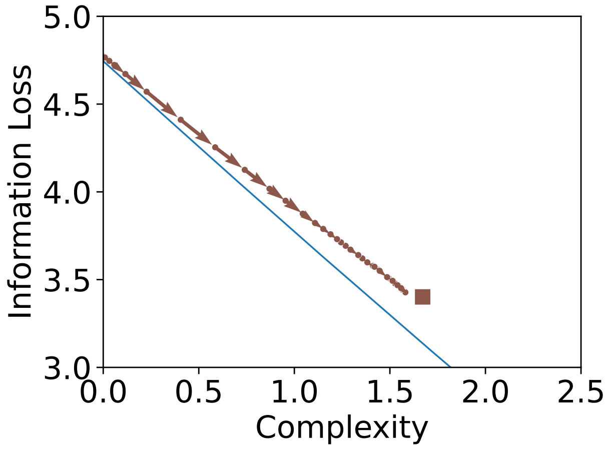

(**b**) Communicative Space

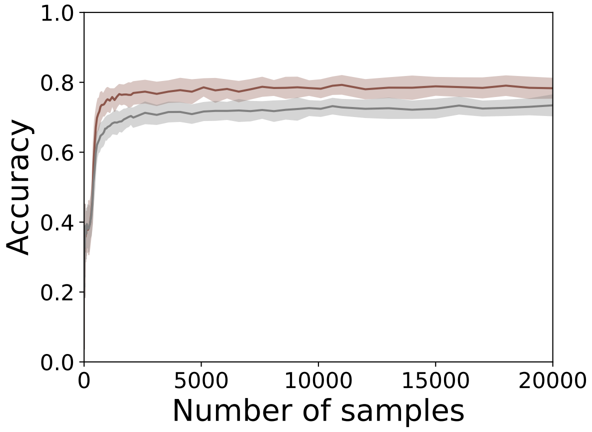

(**c**) Accuracy

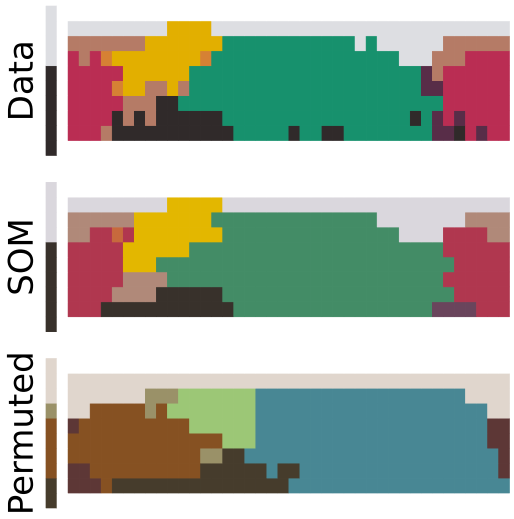

(**d**) Mode Maps

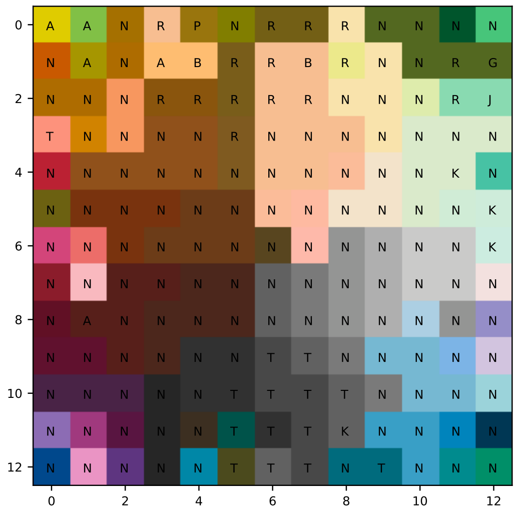

(**e**) Learnt SOM Cells

1.16. *Buglere*; $|\mathcal{W}| = 14$

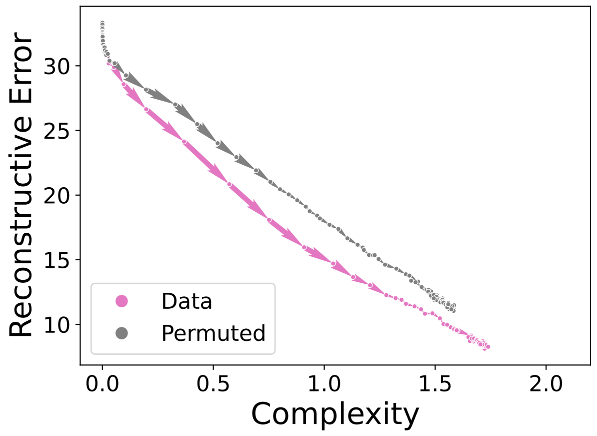

(**a**) Learning Space

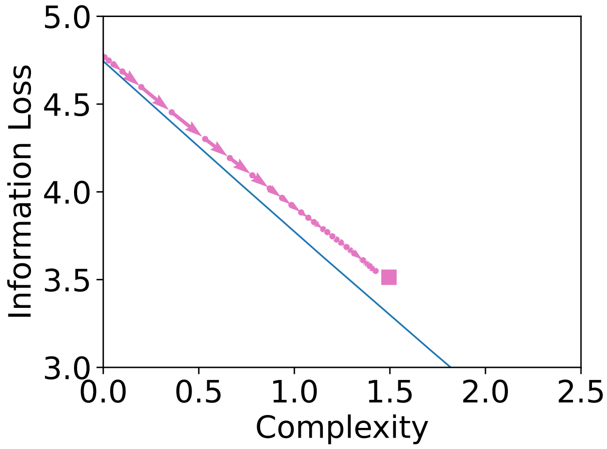

(**b**) Communicative Space

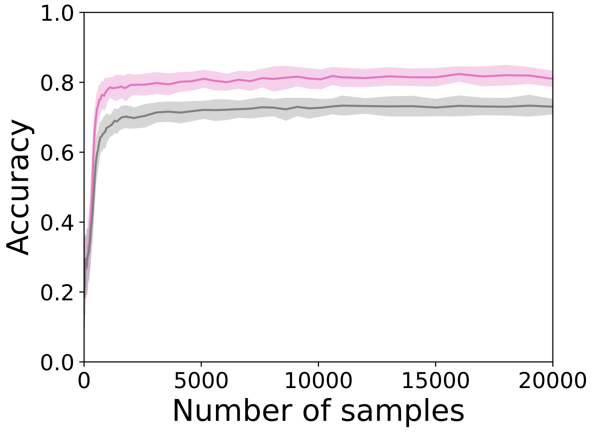

(**c**) Accuracy

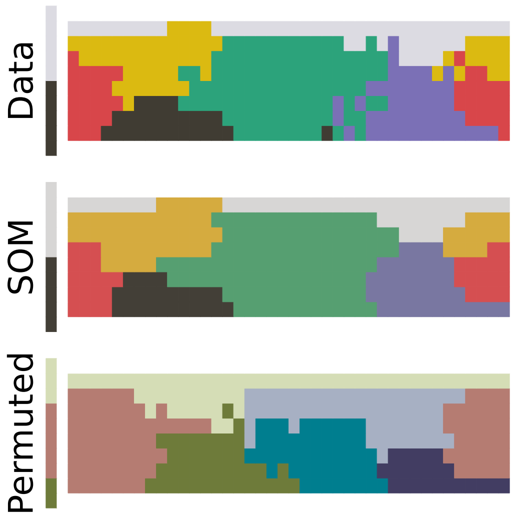

(**d**) Mode Maps

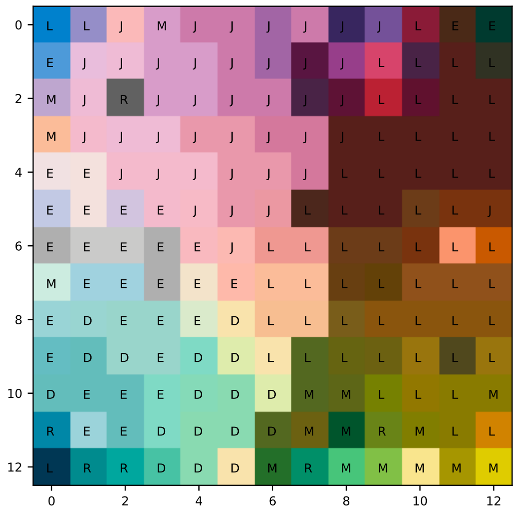

(**e**) Learnt SOM Cells

1.17. Cakchiquel; $|\mathcal{W}| = 28$

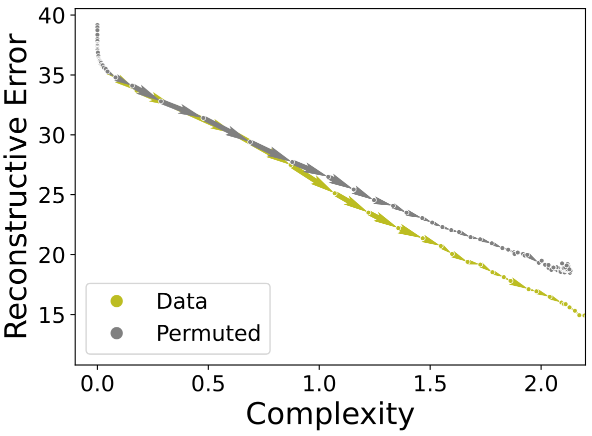

(**a**) Learning Space

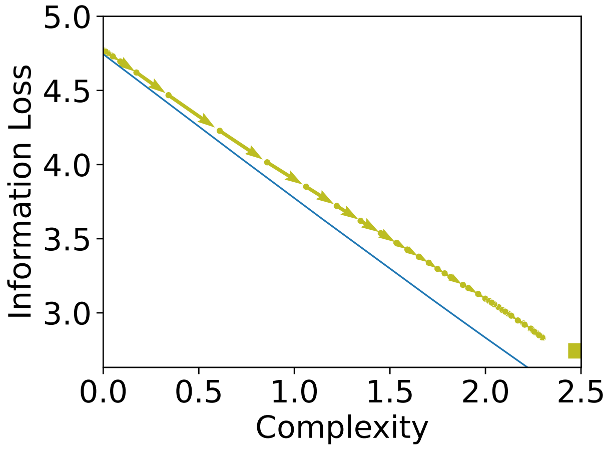

(**b**) Communicative Space

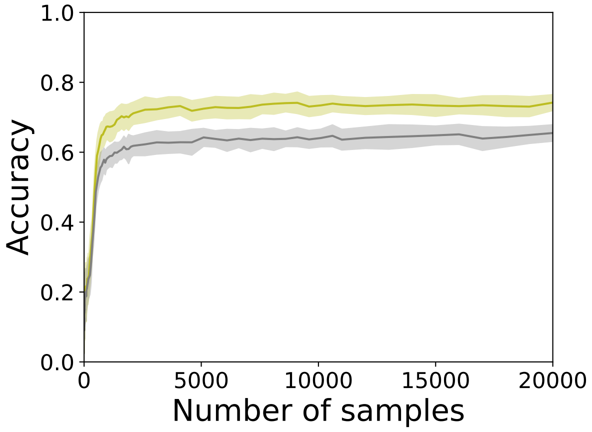

(**c**) Accuracy

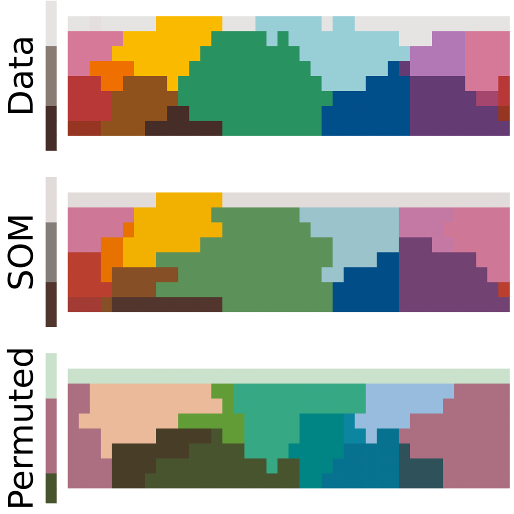

(**d**) Mode Maps

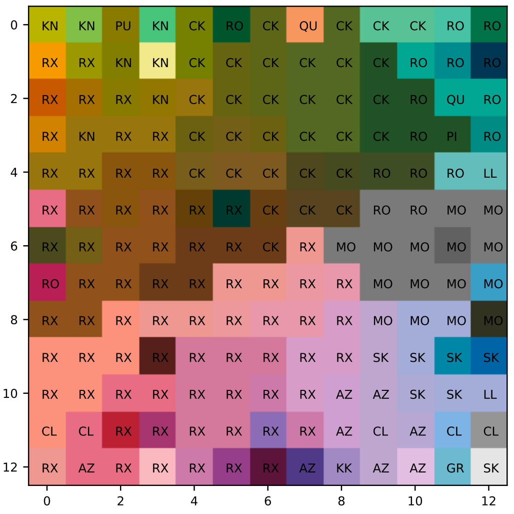

(**e**) Learnt SOM Cells

1.18. Ucayali Campa; $|\mathcal{W}| = 17$

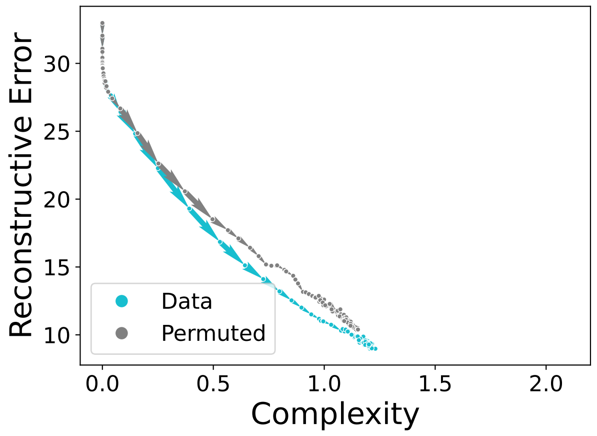

(**a**) Learning Space

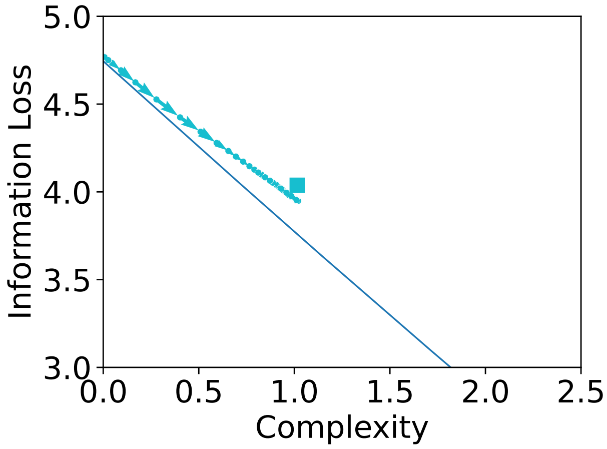

(**b**) Communicative Space

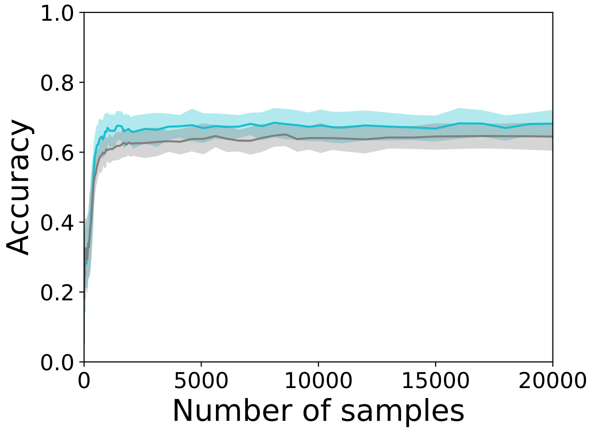

(**c**) Accuracy

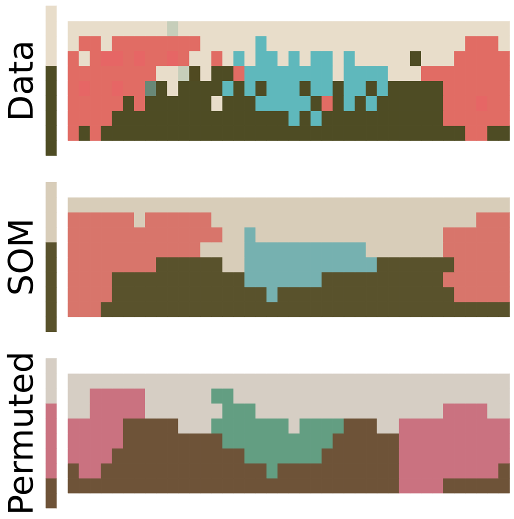

(**d**) Mode Maps

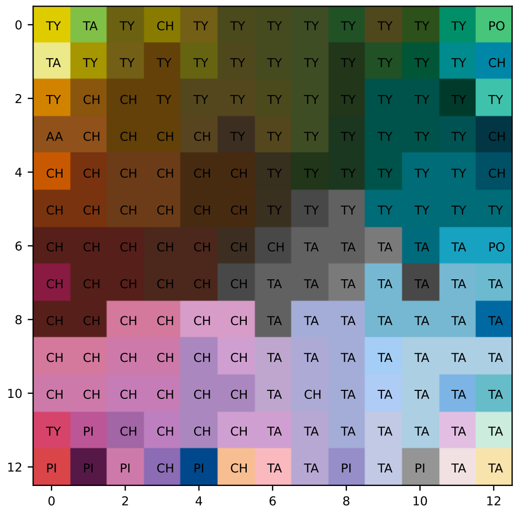

(**e**) Learnt SOM Cells

1.19. Camsa; $|\mathcal{W}| = 31$

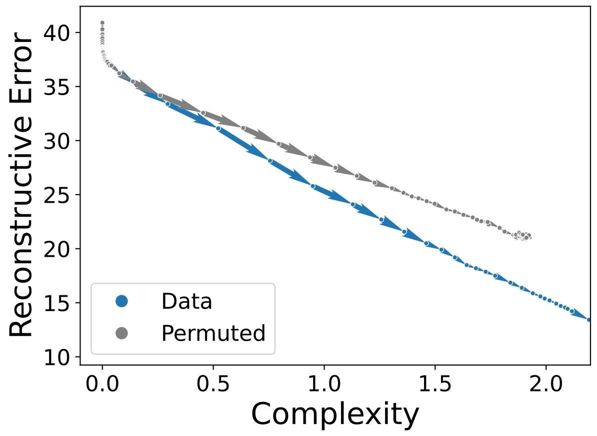

(**a**) Learning Space

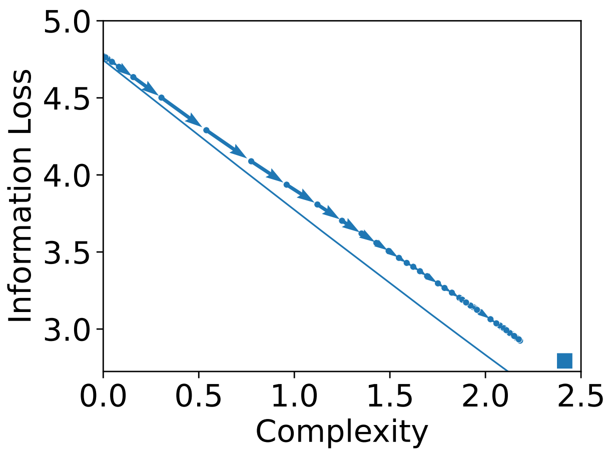

(**b**) Communicative Space

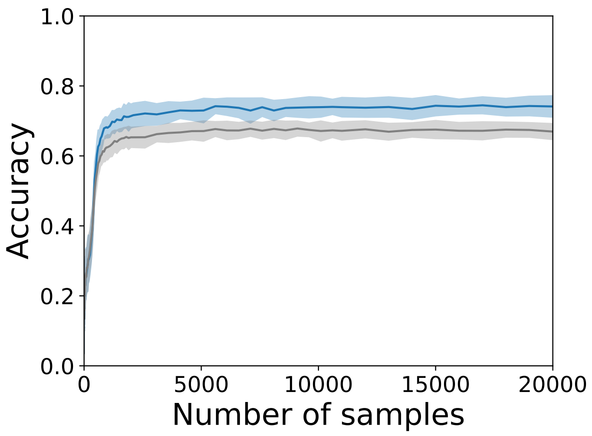

(**c**) Accuracy

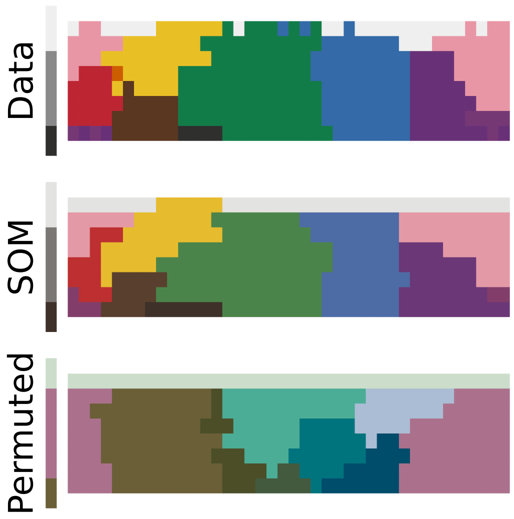

(**d**) Mode Maps

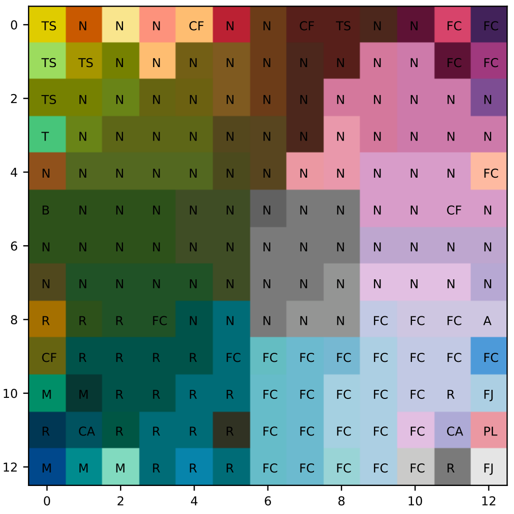

(**e**) Learnt SOM Cells

1.20. Candoshi; $|\mathcal{W}| = 19$

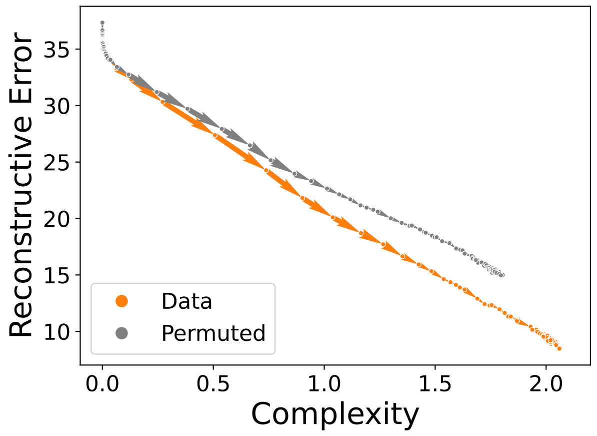

(**a**) Learning Space

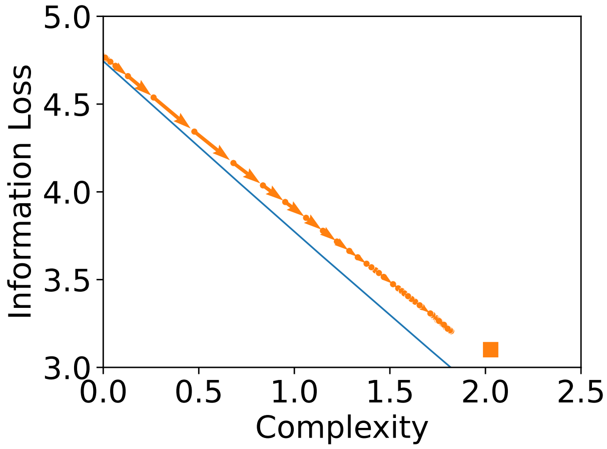

(**b**) Communicative Space

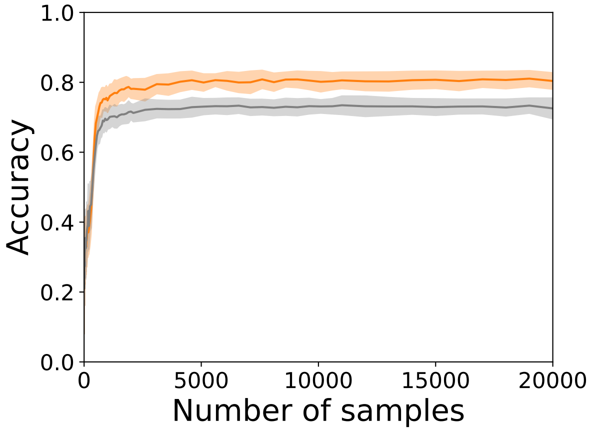

(**c**) Accuracy

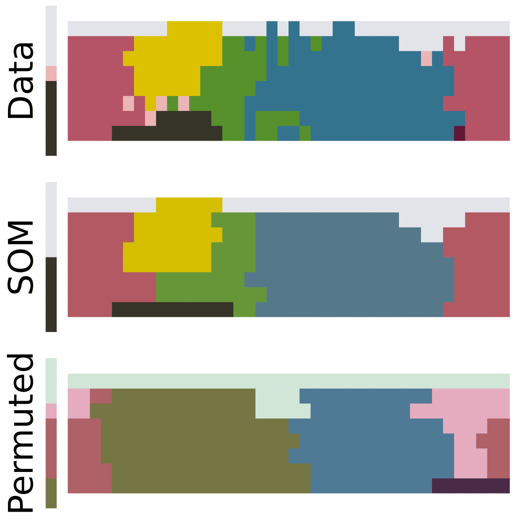

(**d**) Mode Maps

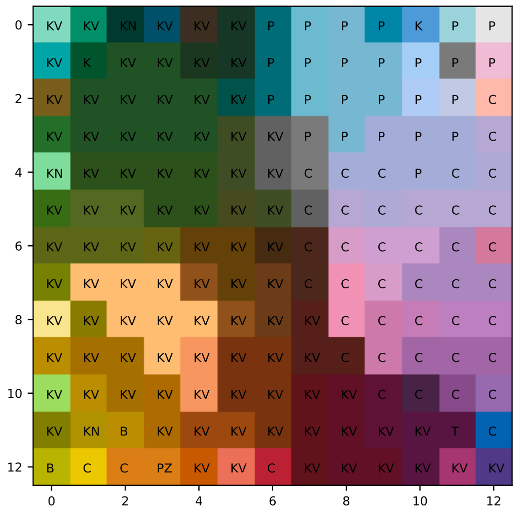

(**e**) Learnt SOM Cells

1.21. Cavineña; $|\mathcal{W}| = 10$

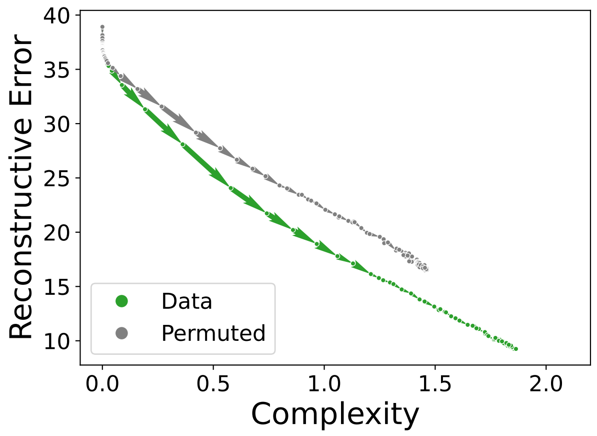

(**a**) Learning Space

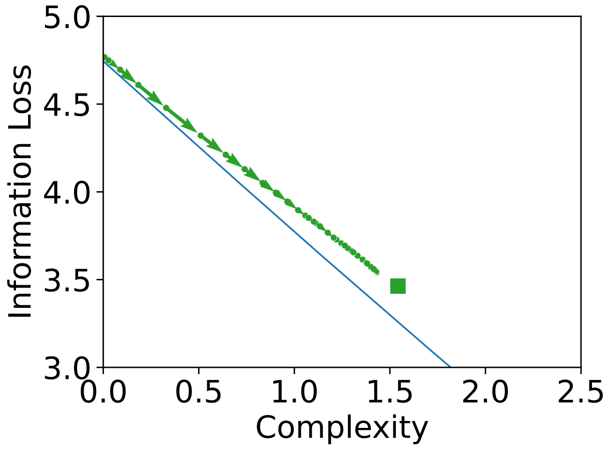

(**b**) Communicative Space

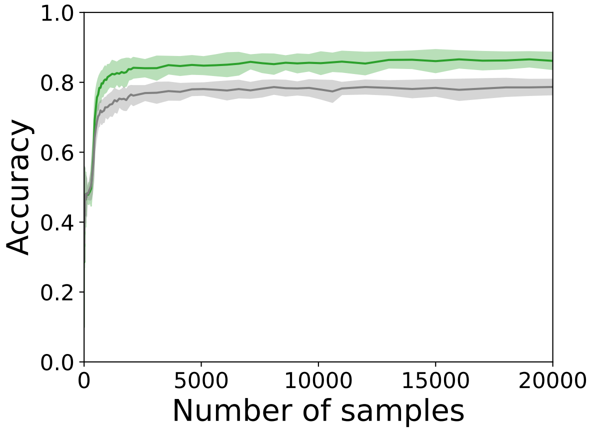

(**c**) Accuracy

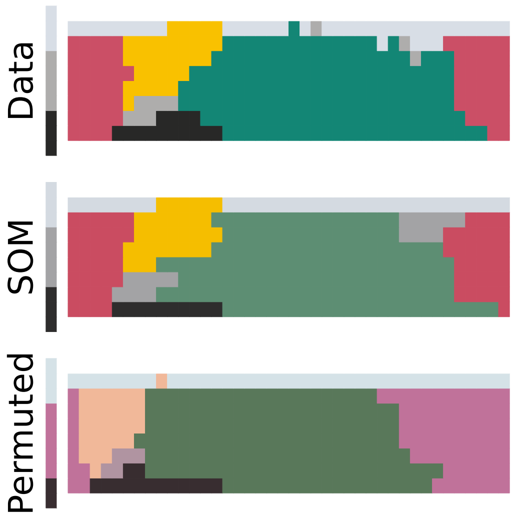

(**d**) Mode Maps

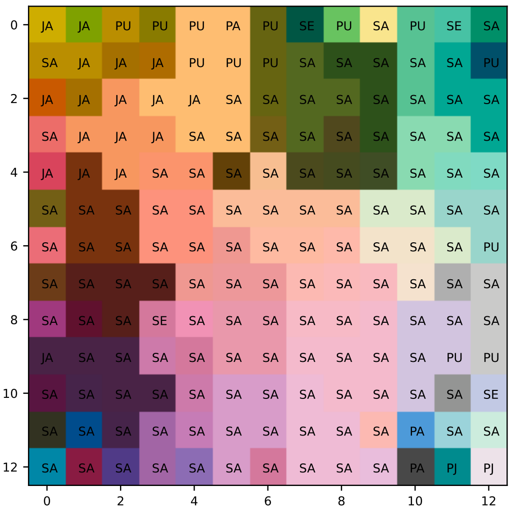

(**e**) Learnt SOM Cells

1.22. Cayapa; $|\mathcal{W}| = 13$

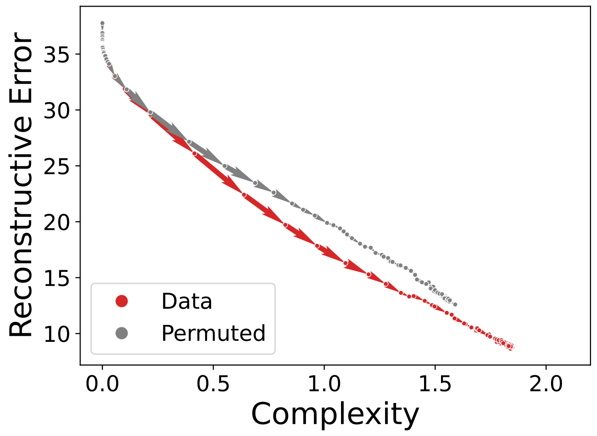

(**a**) Learning Space

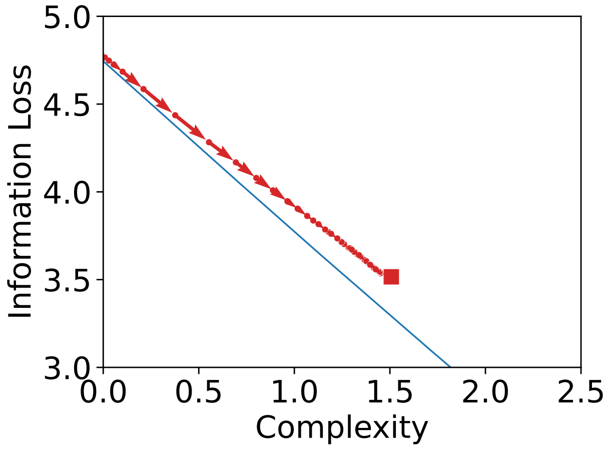

(**b**) Communicative Space

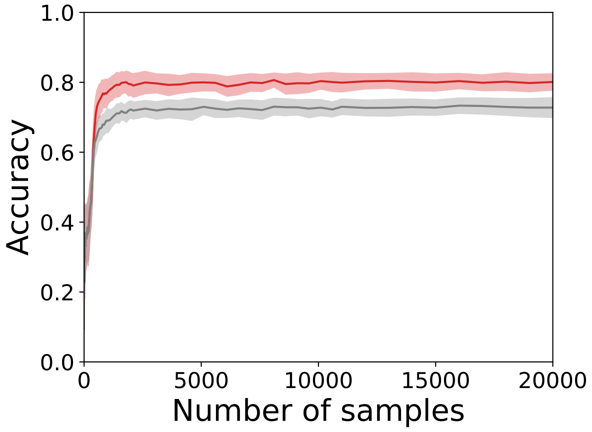

(**c**) Accuracy

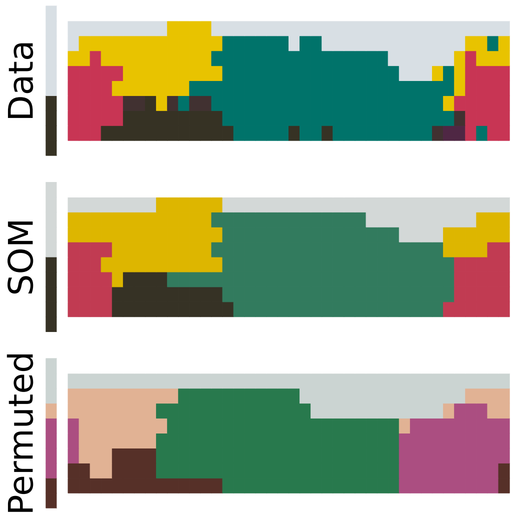

(**d**) Mode Maps

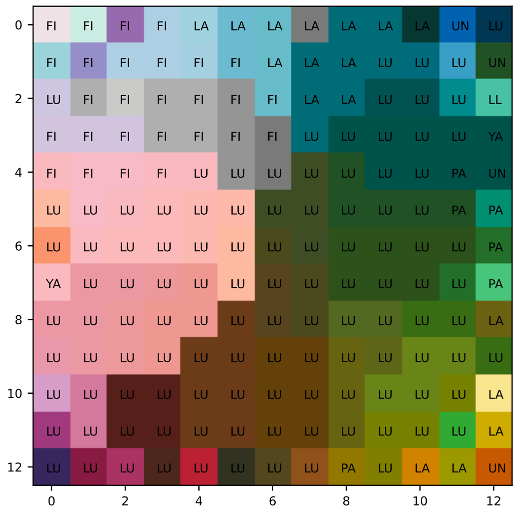

(**e**) Learnt SOM Cells

1.23. Chácobo; $|\mathcal{W}| = 6$

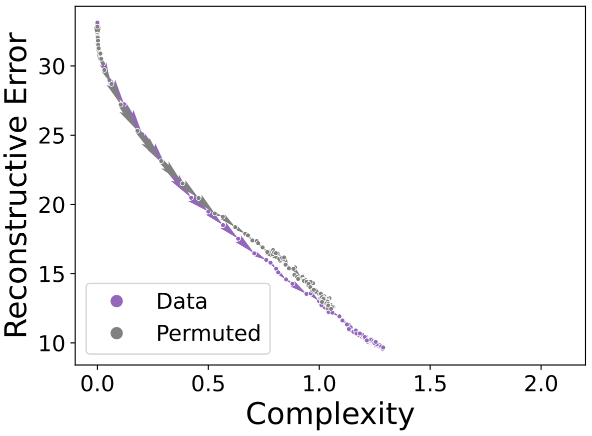

(**a**) Learning Space

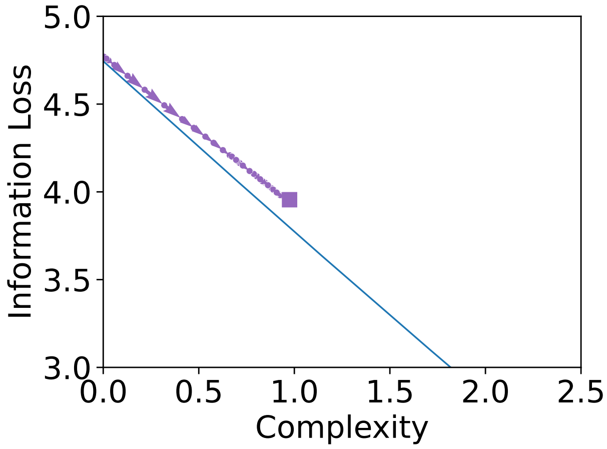

(**b**) Communicative Space

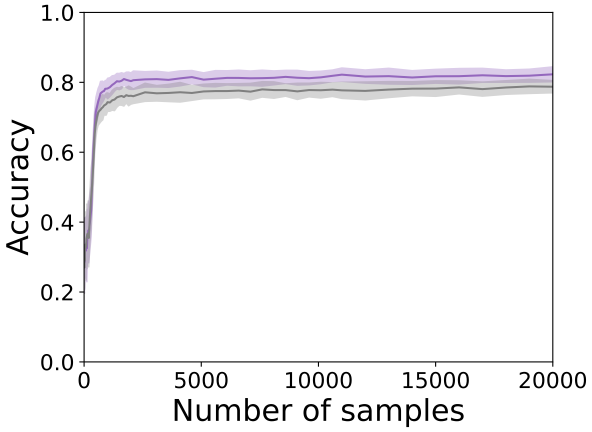

(**c**) Accuracy

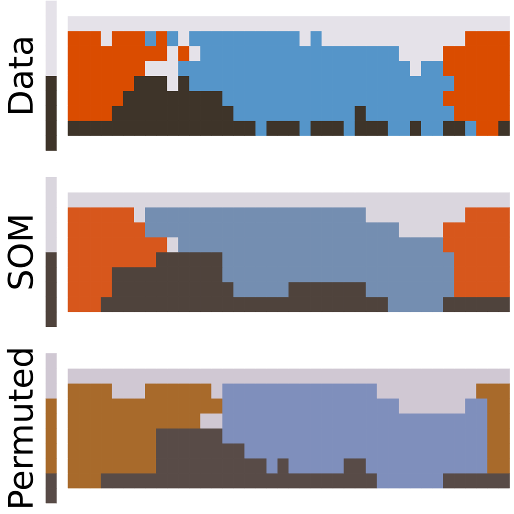

(**d**) Mode Maps

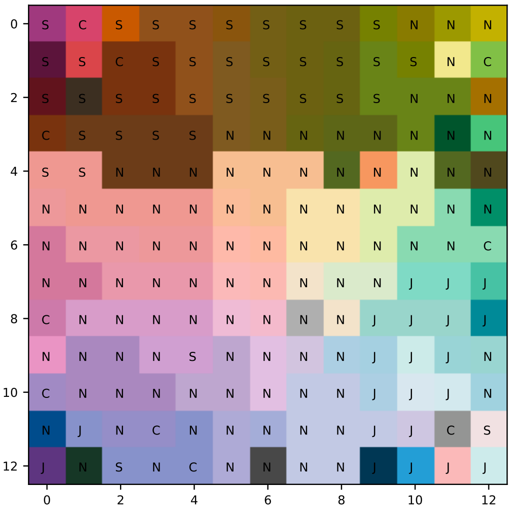

(**e**) Learnt SOM Cells

1.24. Chavacano; $|\mathcal{W}| = 19$

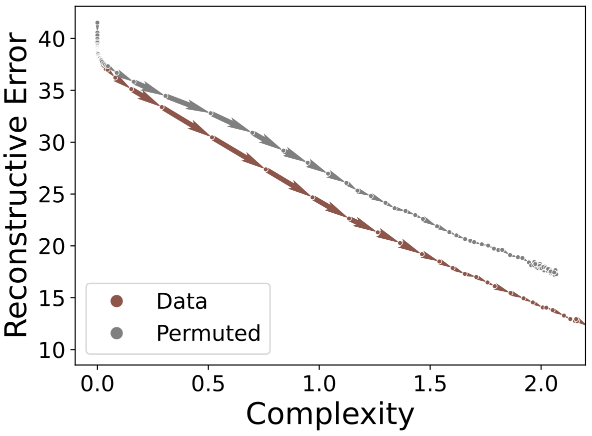

(**a**) Learning Space

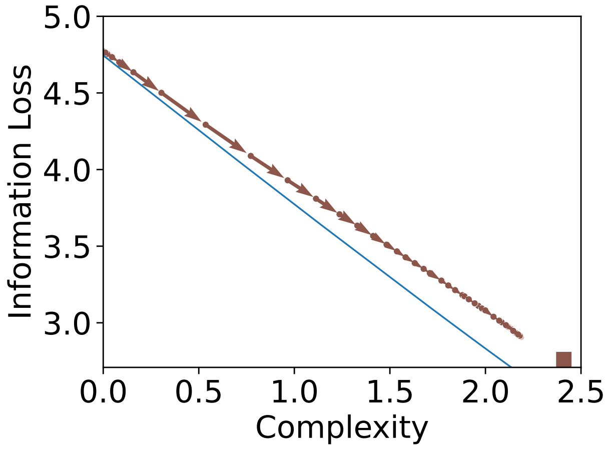

(**b**) Communicative Space

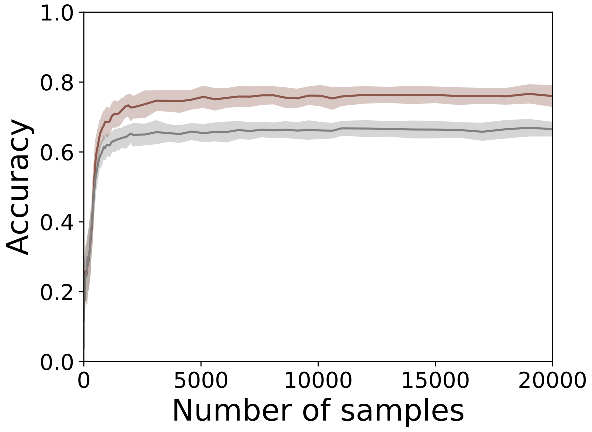

(**c**) Accuracy

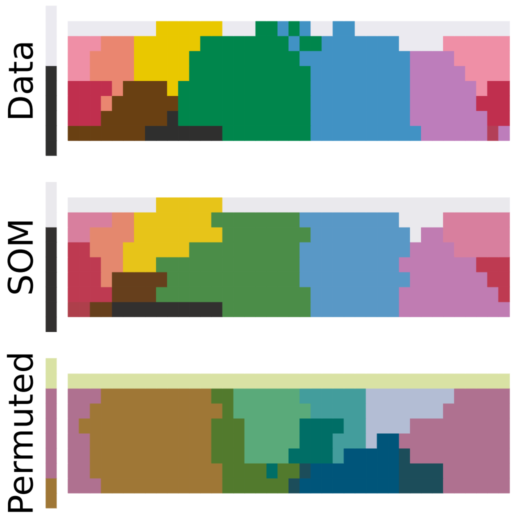

(**d**) Mode Maps

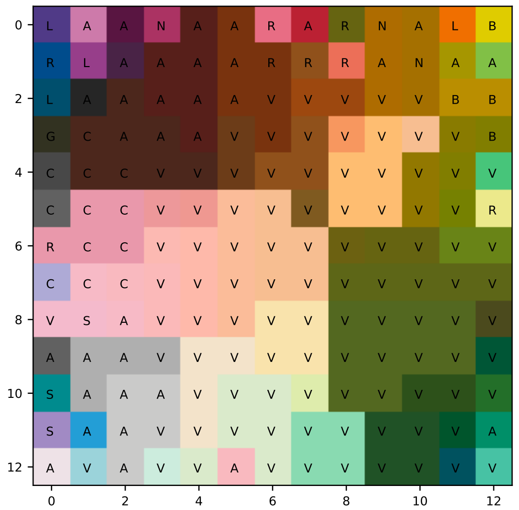

(**e**) Learnt SOM Cells

1.25. Chayahuita; $|\mathcal{W}| = 8$

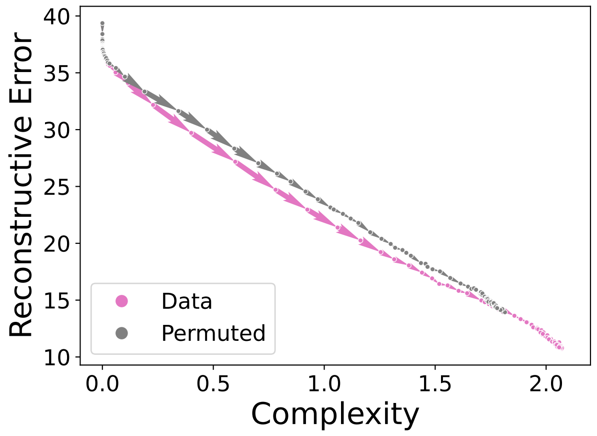

(**a**) Learning Space

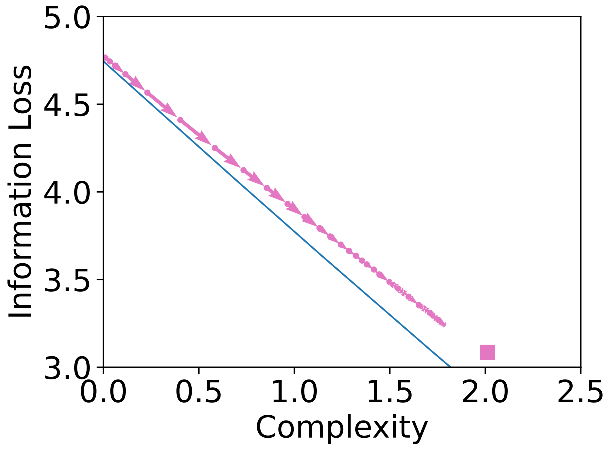

(**b**) Communicative Space

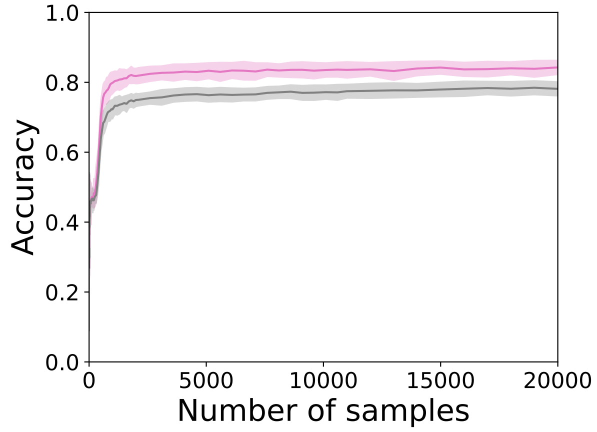

(**c**) Accuracy

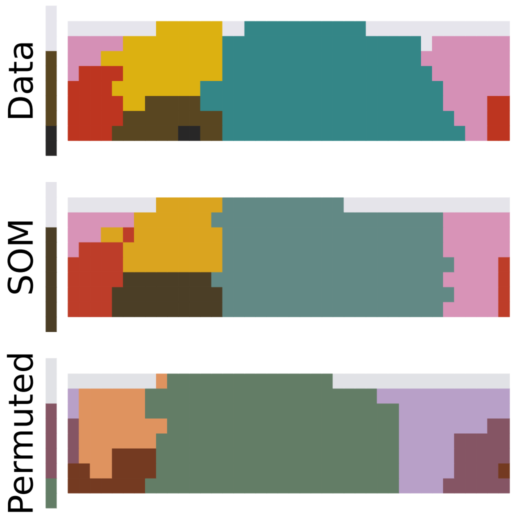

(**d**) Mode Maps

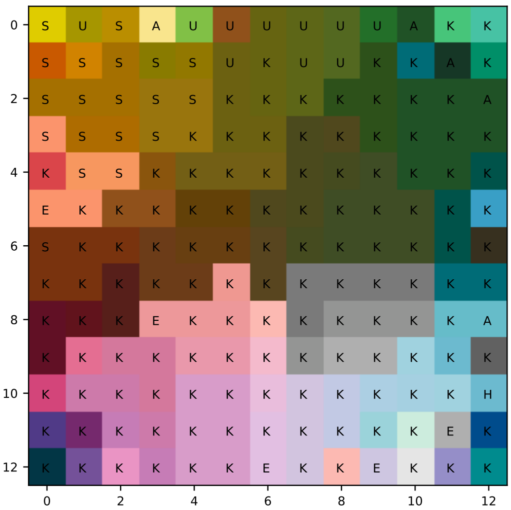

(**e**) Learnt SOM Cells

1.26. Chinanteco; $|\mathcal{W}| = 12$

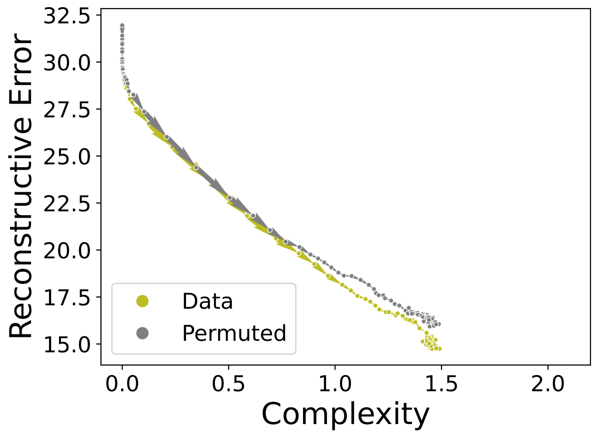

(**a**) Learning Space

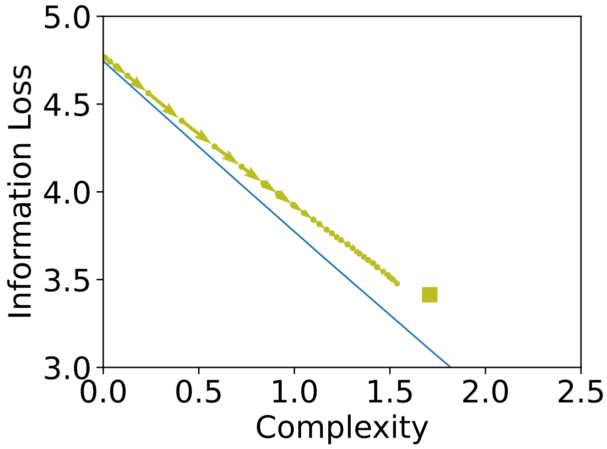

(**b**) Communicative Space

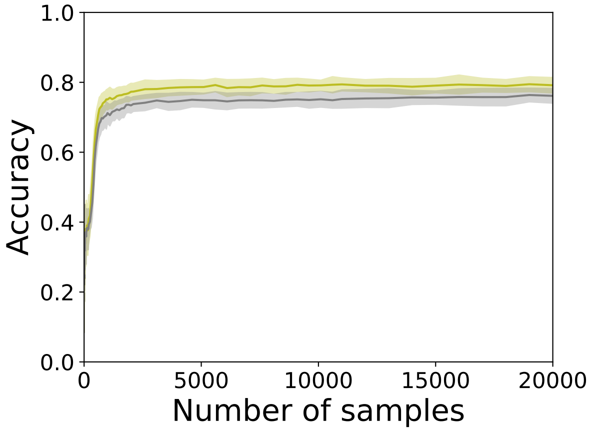

(**c**) Accuracy

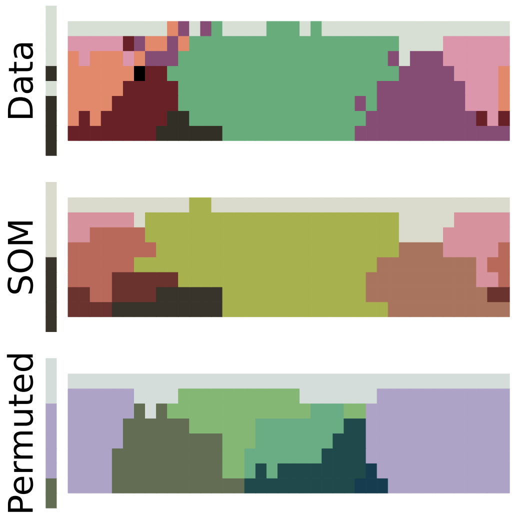

(**d**) Mode Maps

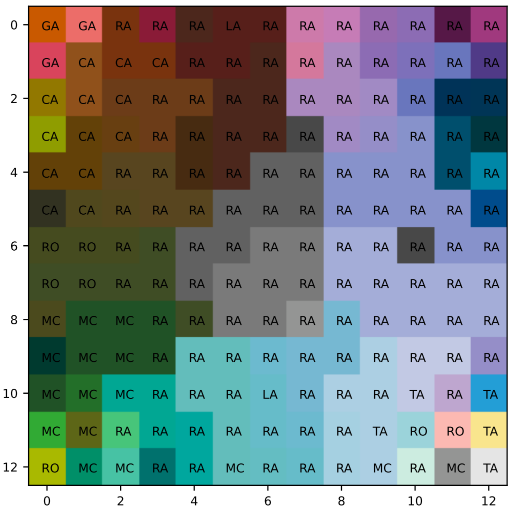

(**e**) Learnt SOM Cells

1.27. *Chiquitano*; $|\mathcal{W}| = 47$

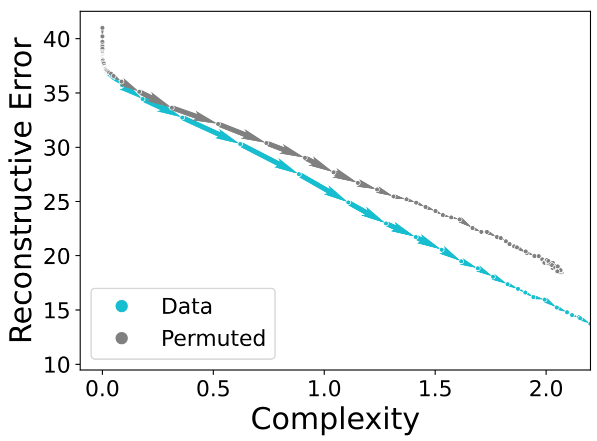

(**a**) Learning Space

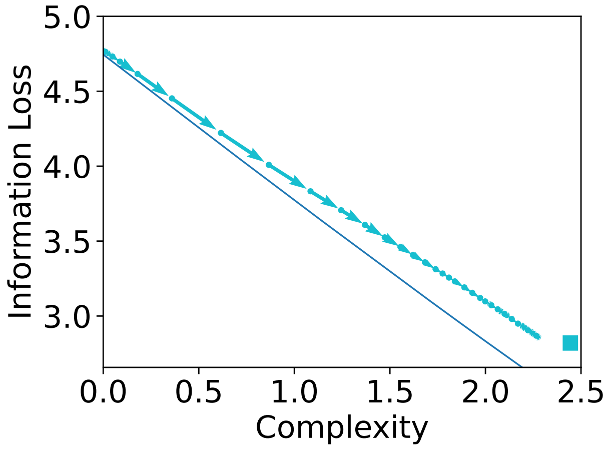

(**b**) Communicative Space

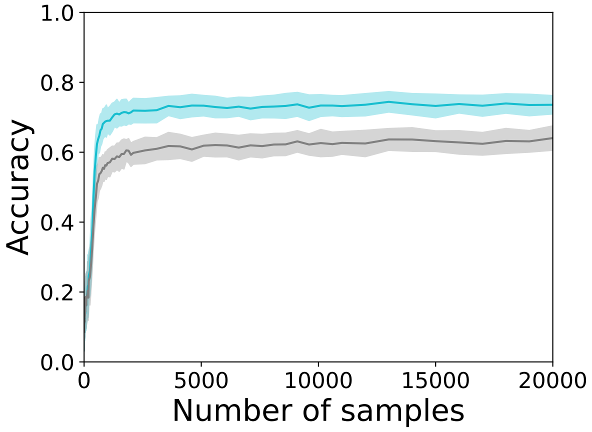

(**c**) Accuracy

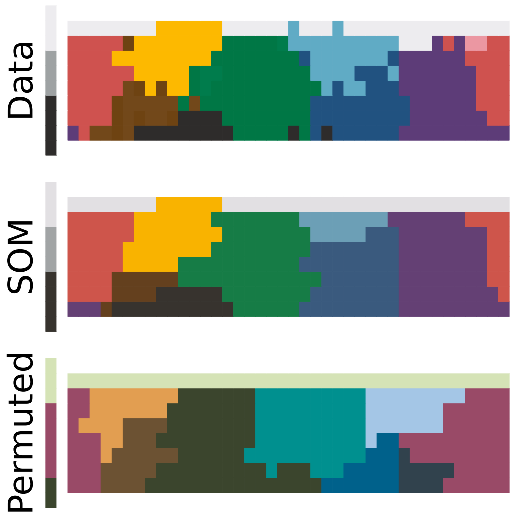

(**d**) Mode Maps

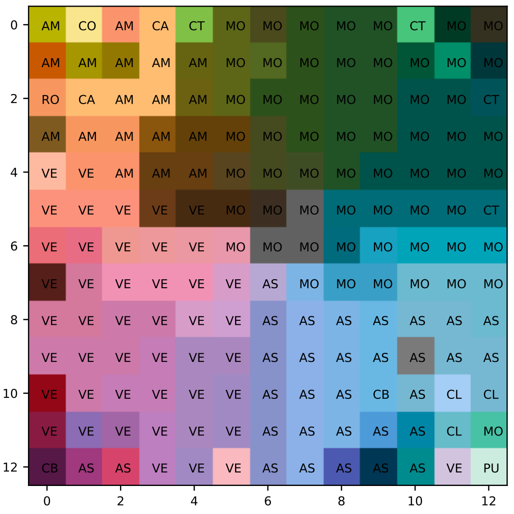

(**e**) Learnt SOM Cells

1.28. Chumburu; $|\mathcal{W}| = 24$

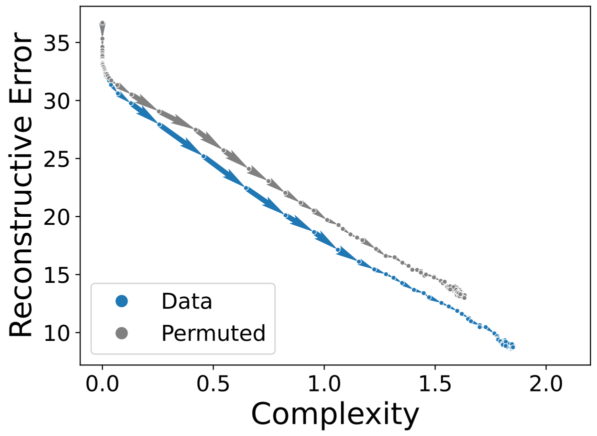

(**a**) Learning Space

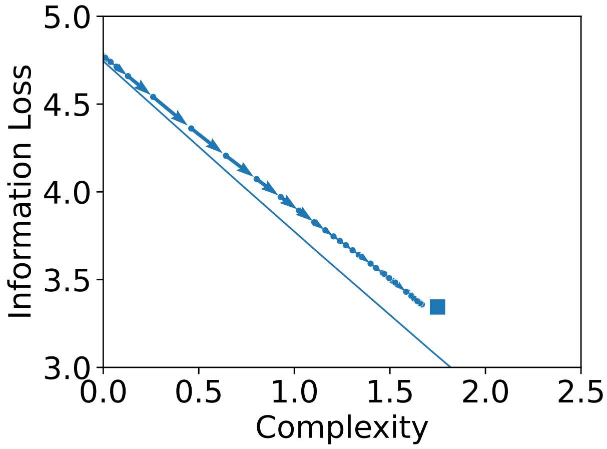

(**b**) Communicative Space

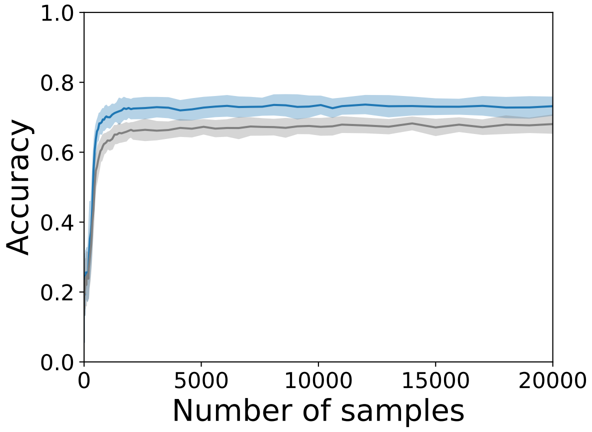

(**c**) Accuracy

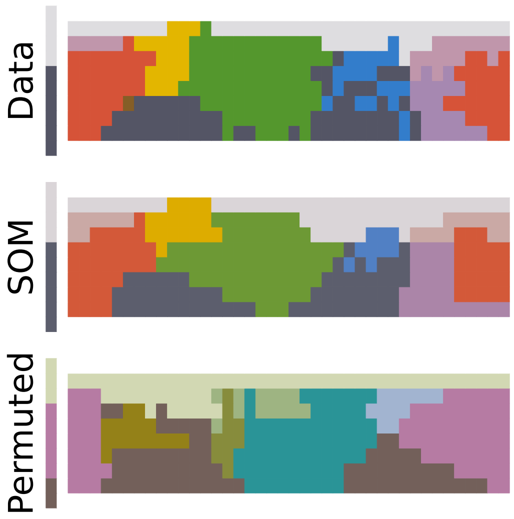

(**d**) Mode Maps

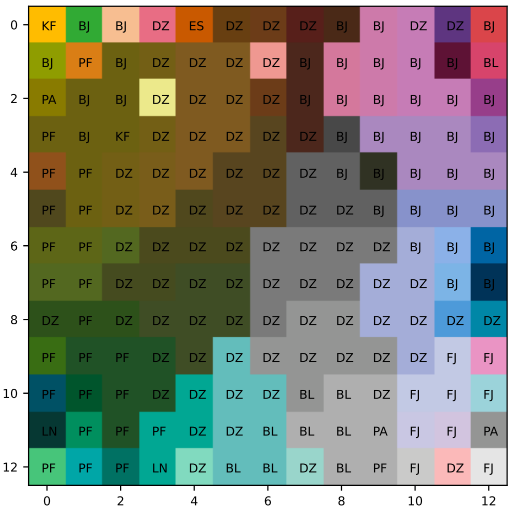

(**e**) Learnt SOM Cells

1.29. Cofán; $|\mathcal{W}| = 10$

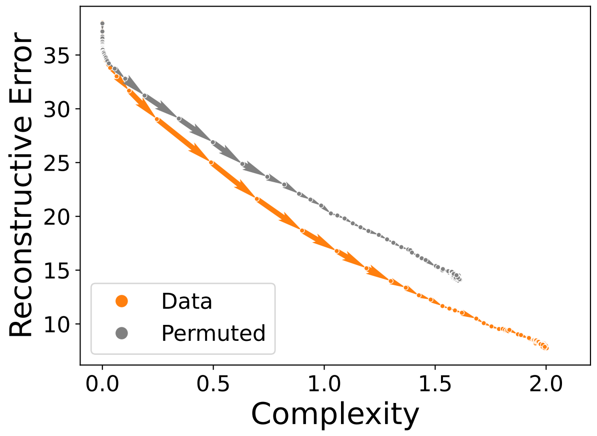

(**a**) Learning Space

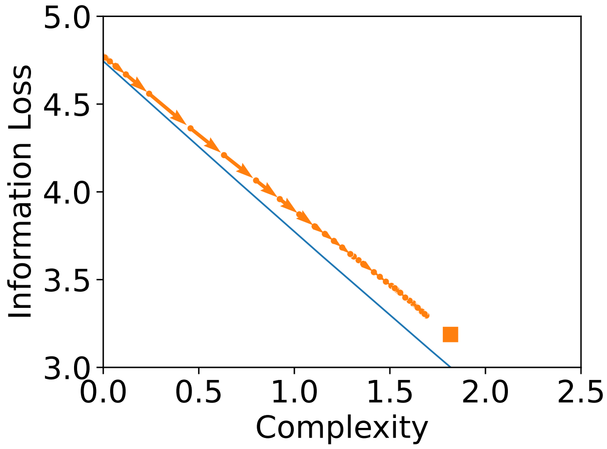

(**b**) Communicative Space

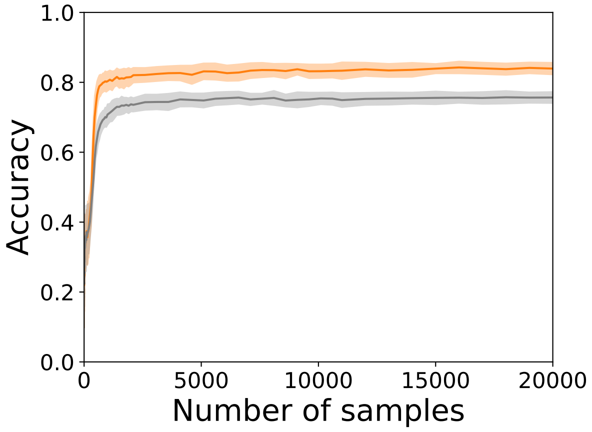

(**c**) Accuracy

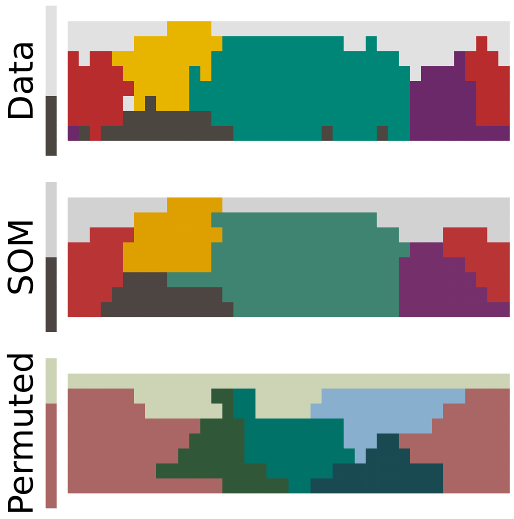

(**d**) Mode Maps

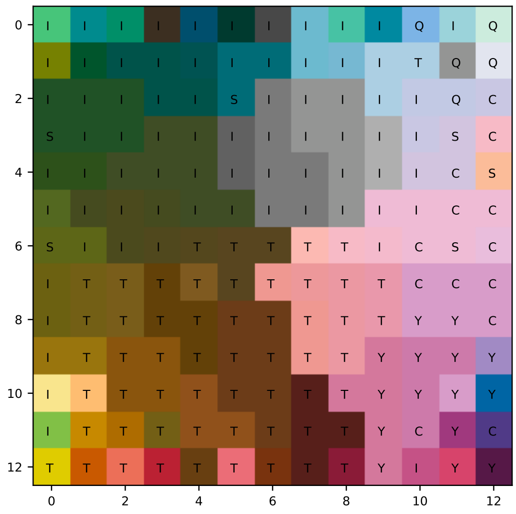

(**e**) Learnt SOM Cells

1.30. Colorado; $|\mathcal{W}| = 6$

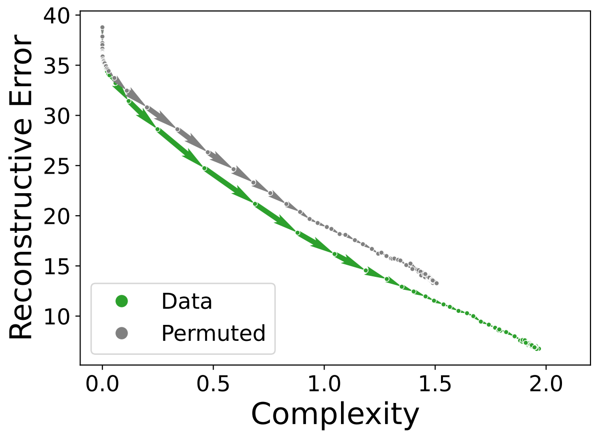

(**a**) Learning Space

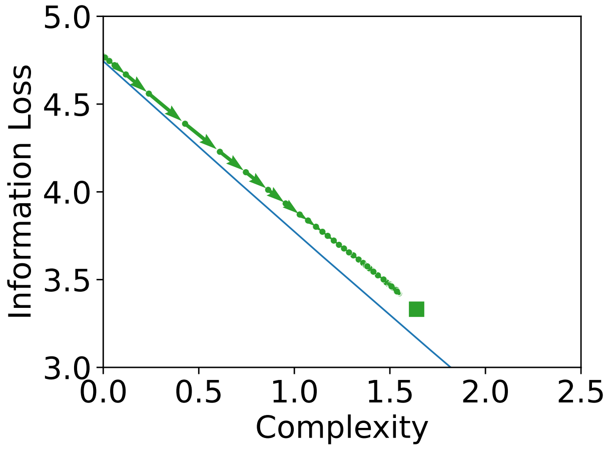

(**b**) Communicative Space

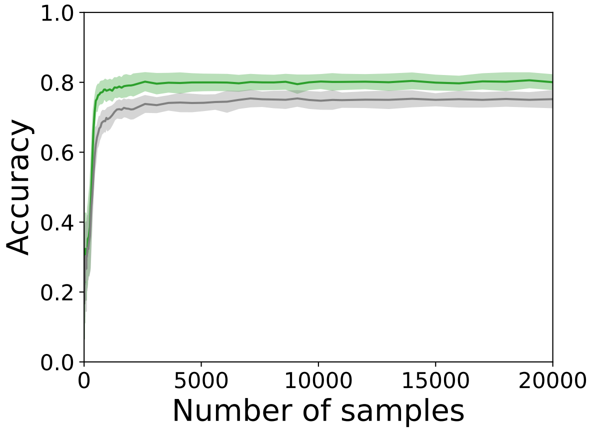

(**c**) Accuracy

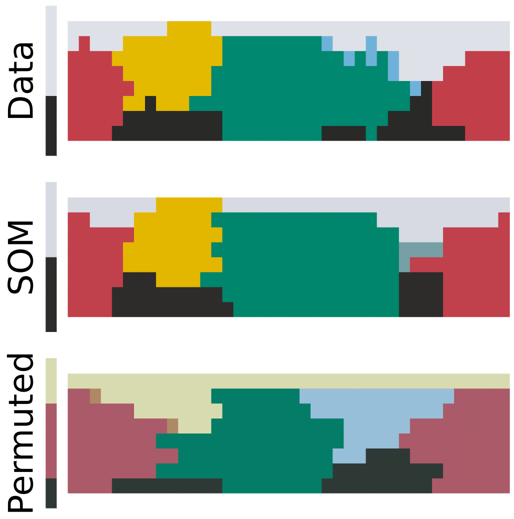

(**d**) Mode Maps

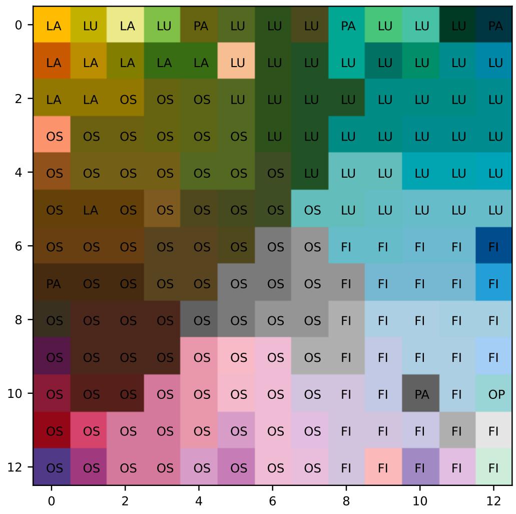

(**e**) Learnt SOM Cells

1.31. Eastern Cree; $|\mathcal{W}| = 8$

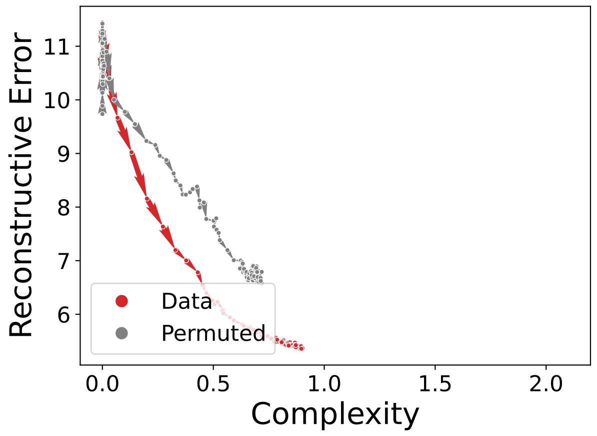

(**a**) Learning Space

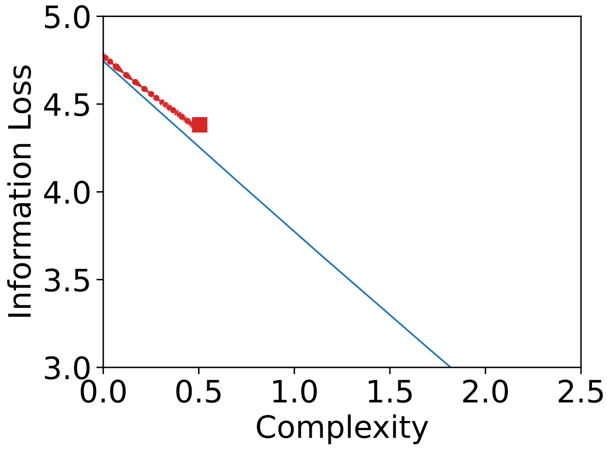

(**b**) Communicative Space

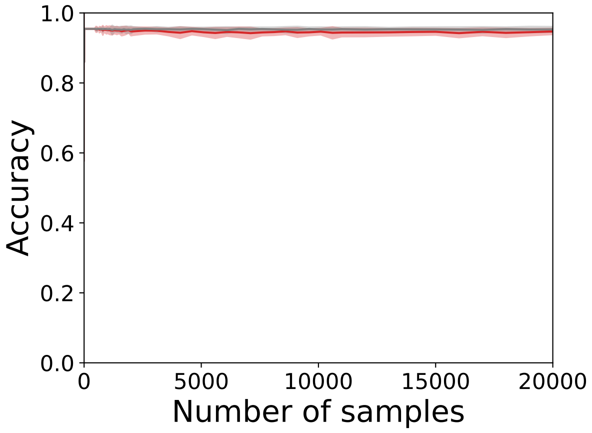

(**c**) Accuracy

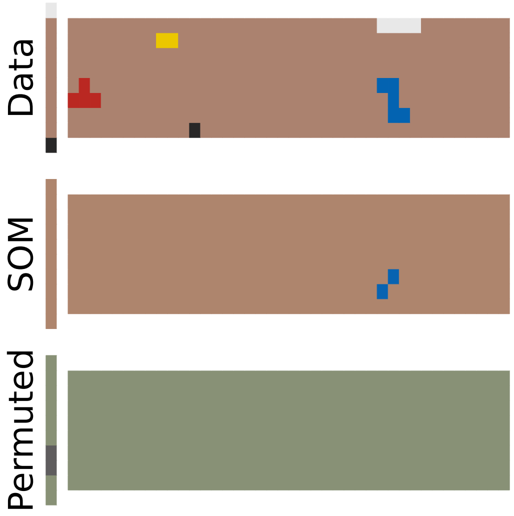

(**d**) Mode Maps

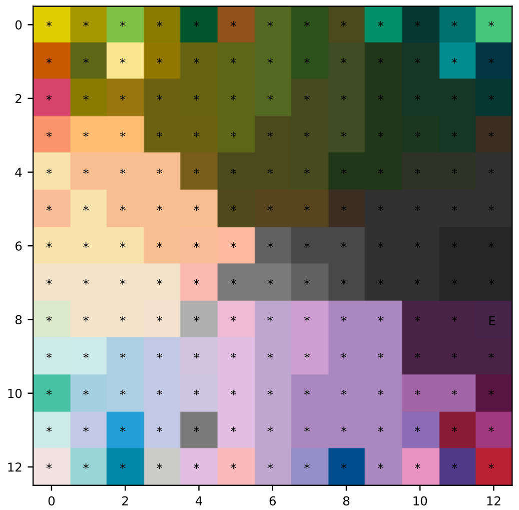

(**e**) Learnt SOM Cells

1.32. *Culina*; $|\mathcal{W}| = 27$

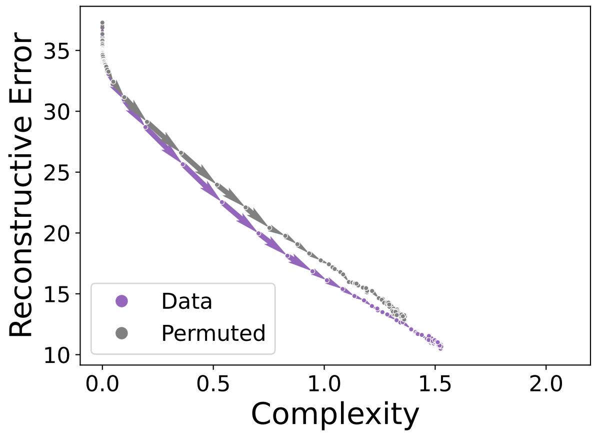

(**a**) Learning Space

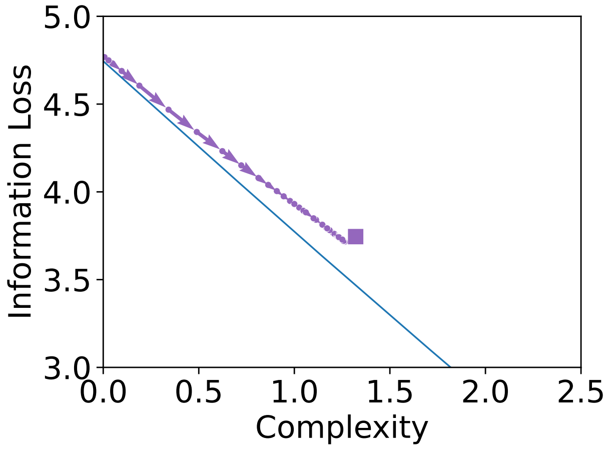

(**b**) Communicative Space

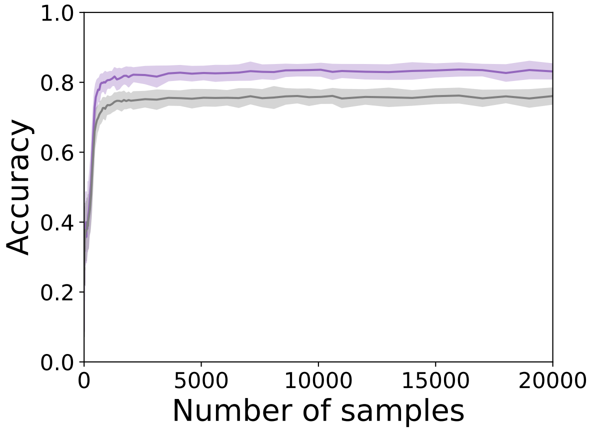

(**c**) Accuracy

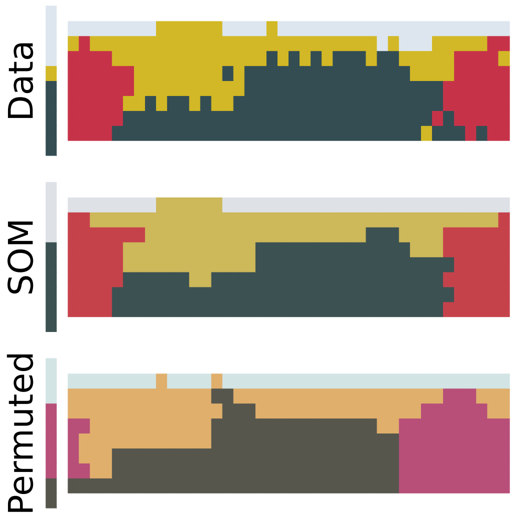

(**d**) Mode Maps

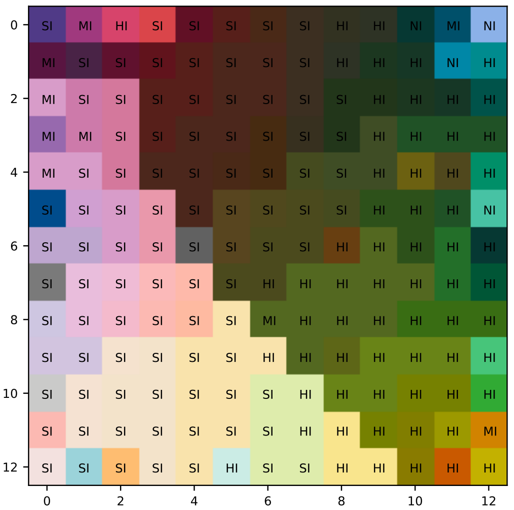

(**e**) Learnt SOM Cells

1.33. Didinga; $|\mathcal{W}| = 8$

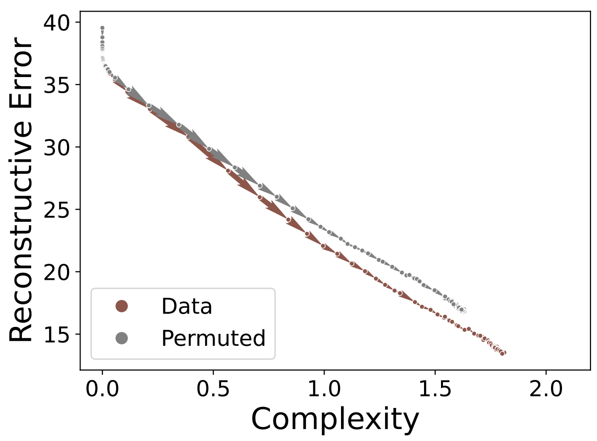

(**a**) Learning Space

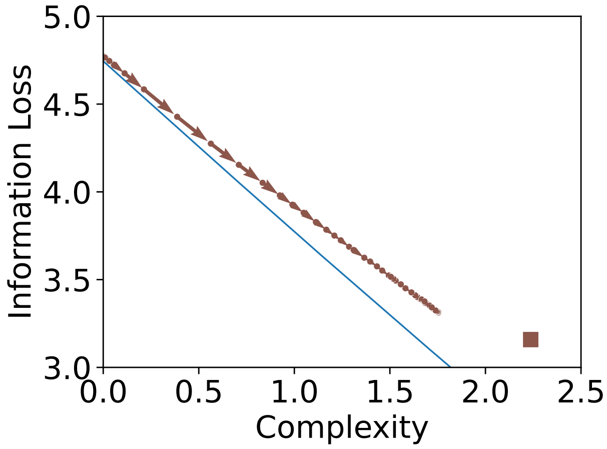

(**b**) Communicative Space

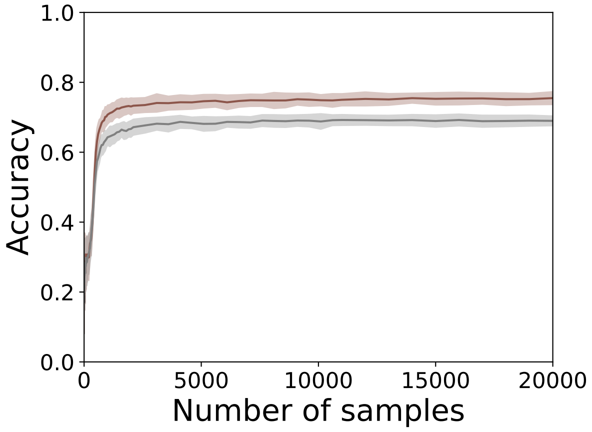

(**c**) Accuracy

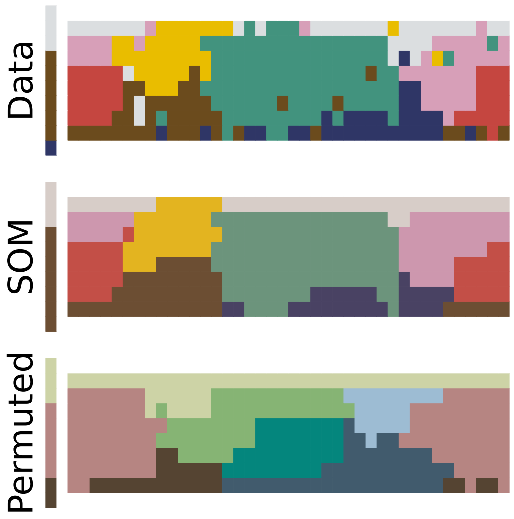

(**d**) Mode Maps

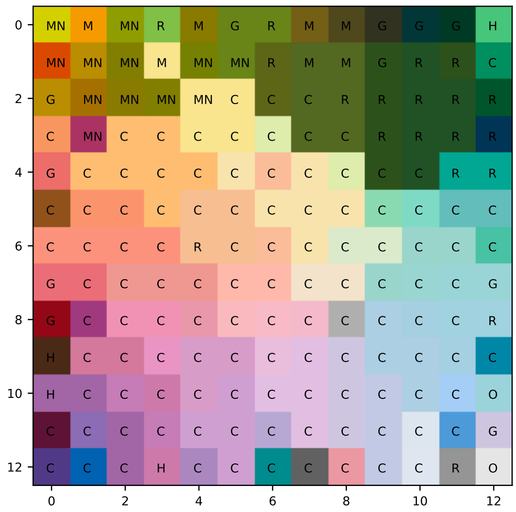

(**e**) Learnt SOM Cells

1.34. Djuka; $|\mathcal{W}| = 23$

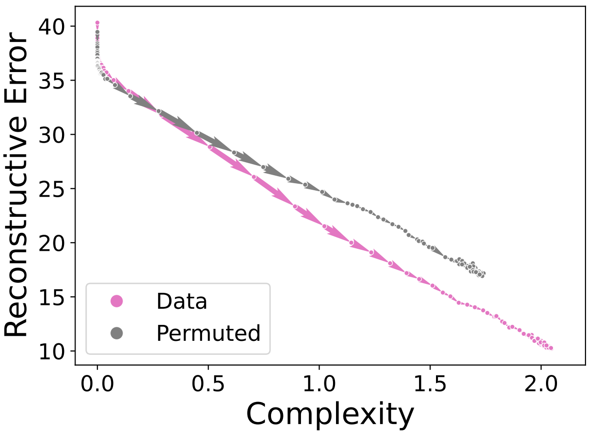

(**a**) Learning Space

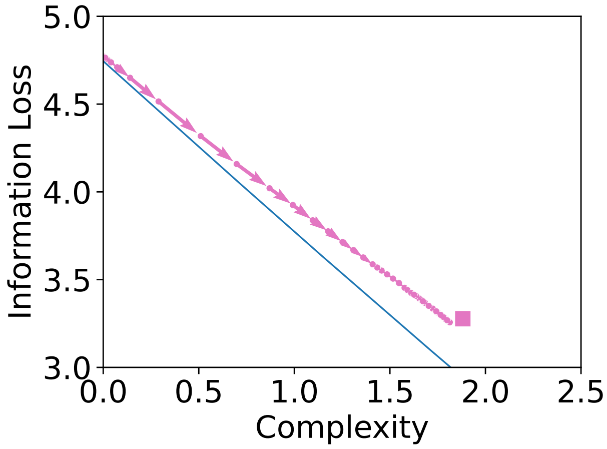

(**b**) Communicative Space

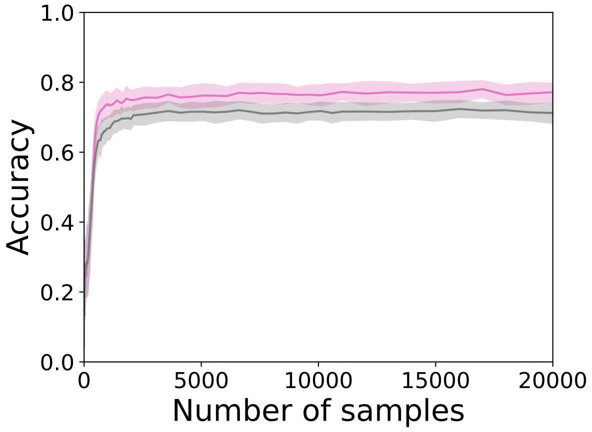

(**c**) Accuracy

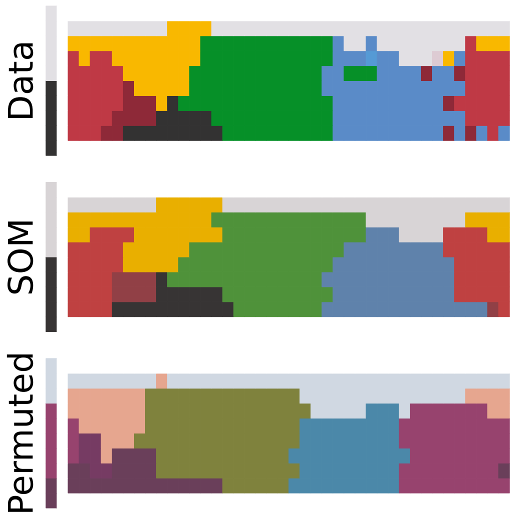

(**d**) Mode Maps

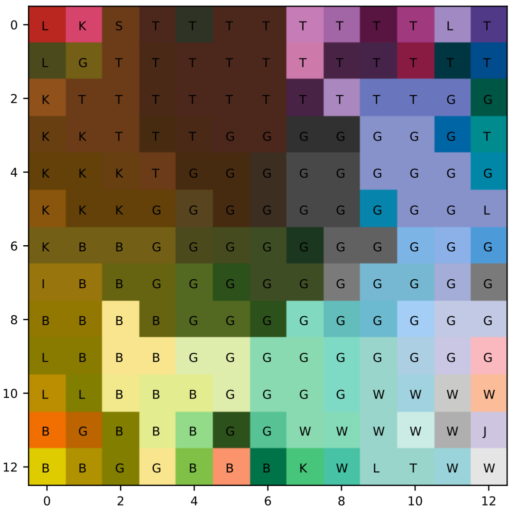

(**e**) Learnt SOM Cells

1.35. Dyimini; $|\mathcal{W}| = 13$

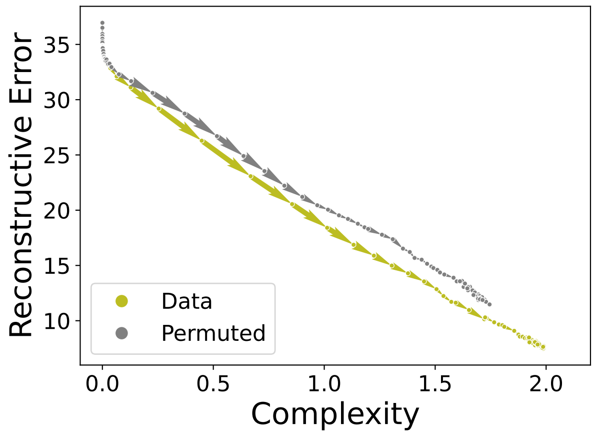

(**a**) Learning Space

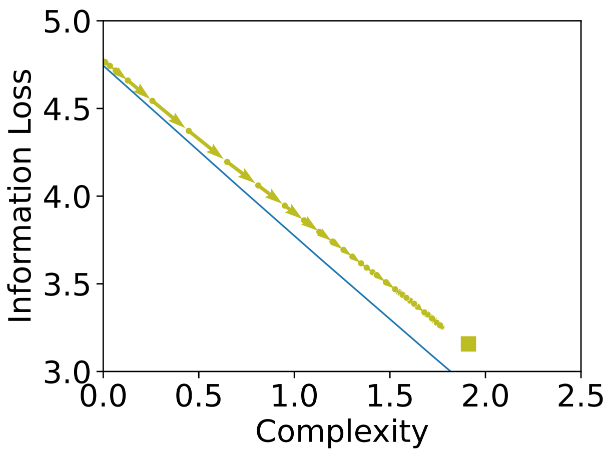

(**b**) Communicative Space

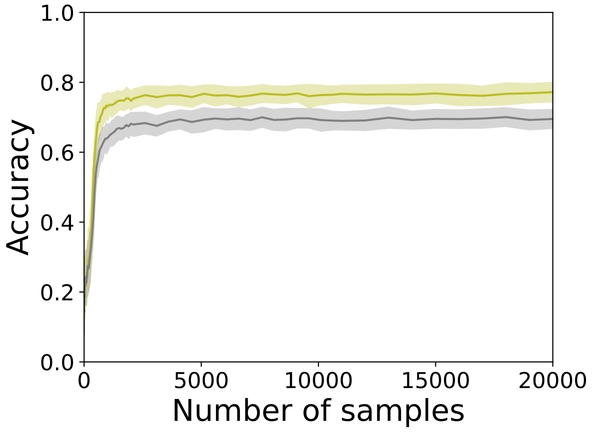

(**c**) Accuracy

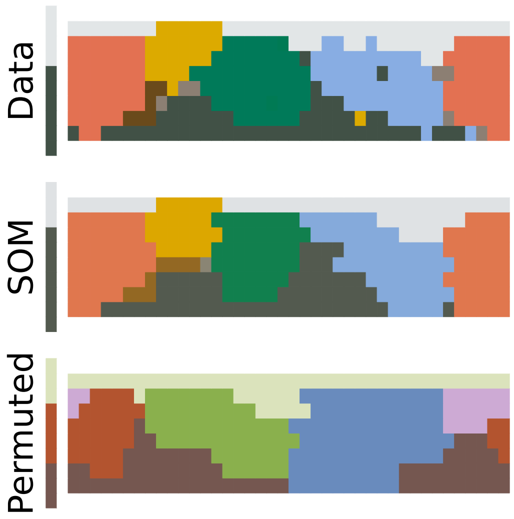

(**d**) Mode Maps

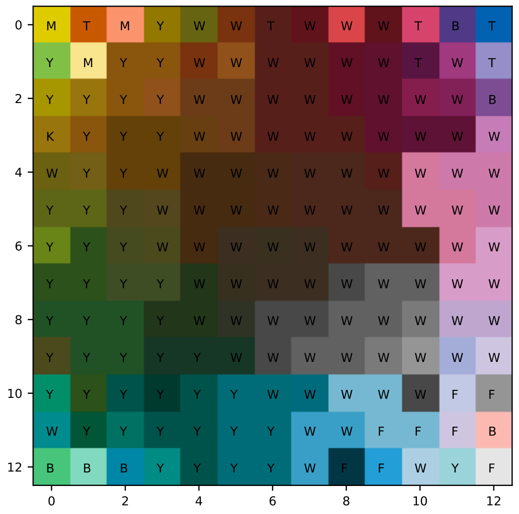

(**e**) Learnt SOM Cells

1.36. Ejagam; $|\mathcal{W}| = 8$

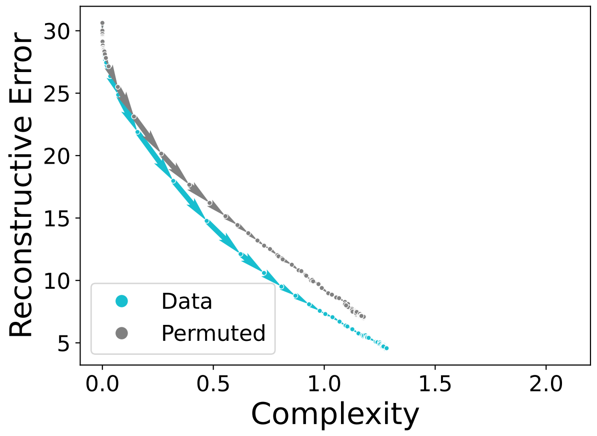

(**a**) Learning Space

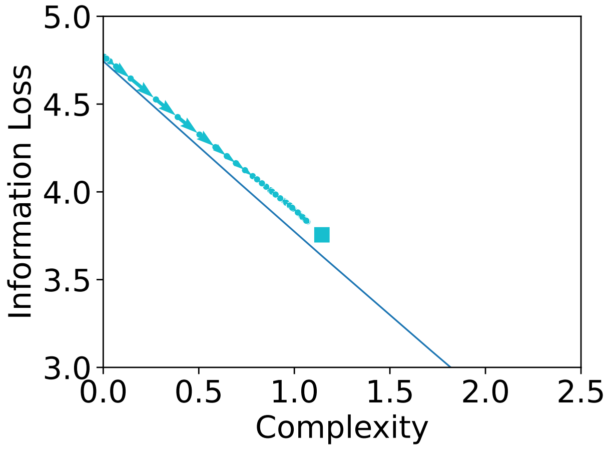

(**b**) Communicative Space

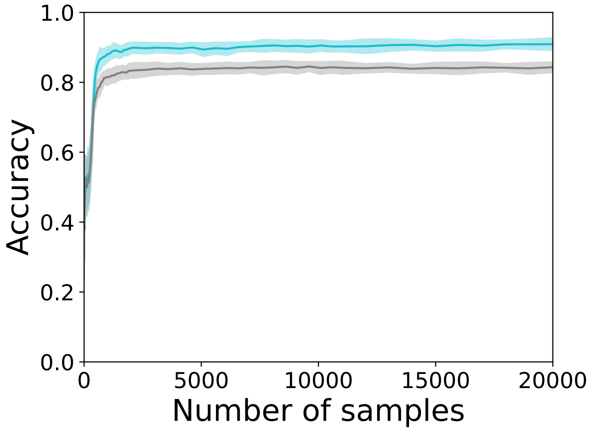

(**c**) Accuracy

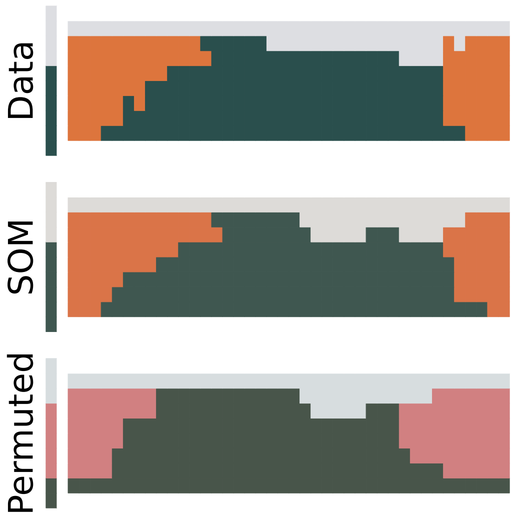

(**d**) Mode Maps

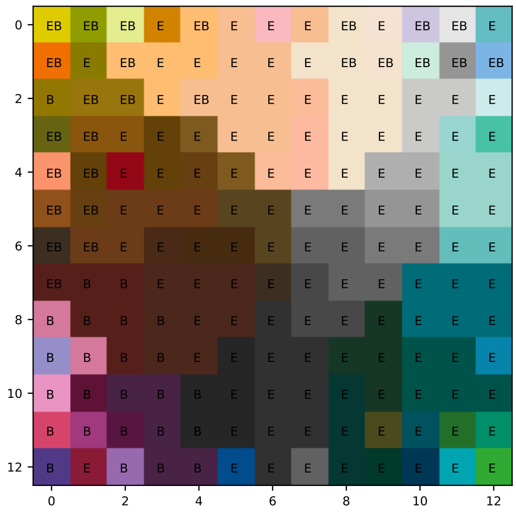

(**e**) Learnt SOM Cells

1.37. Ese Ejja; $|\mathcal{W}| = 13$

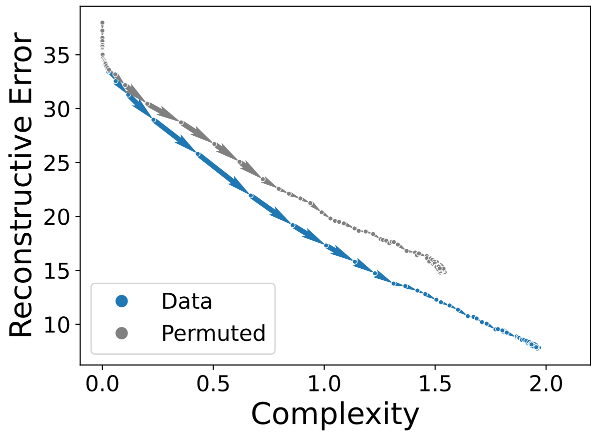

(**a**) Learning Space

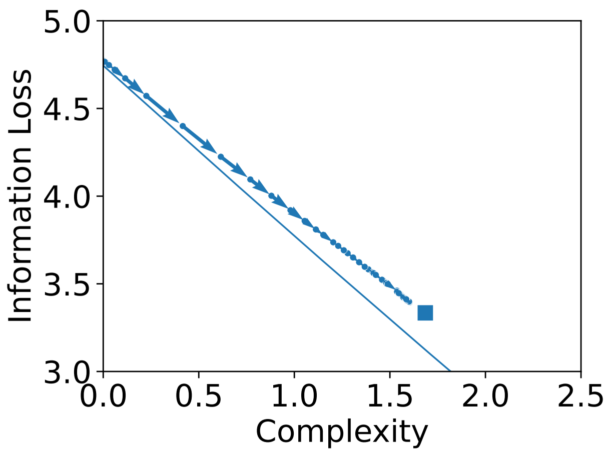

(**b**) Communicative Space

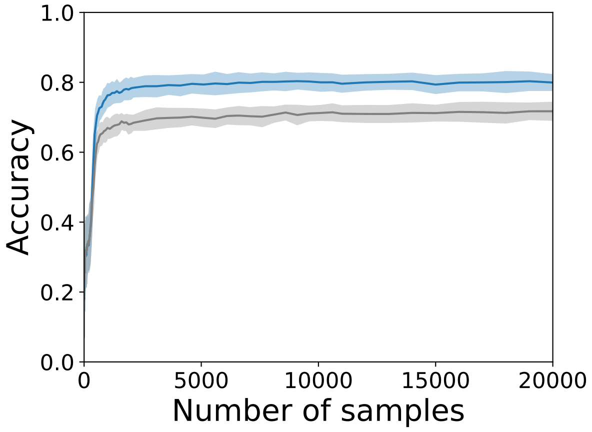

(**c**) Accuracy

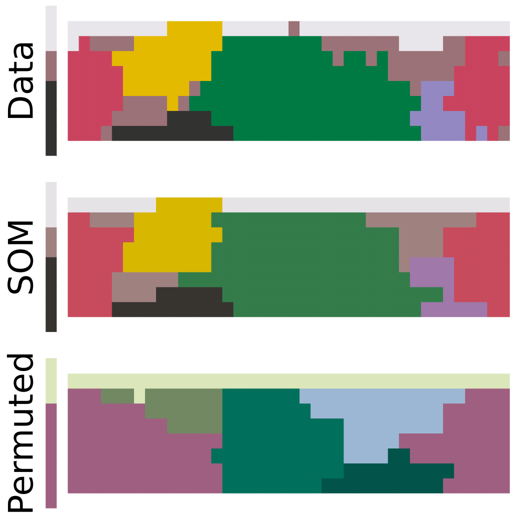

(**d**) Mode Maps

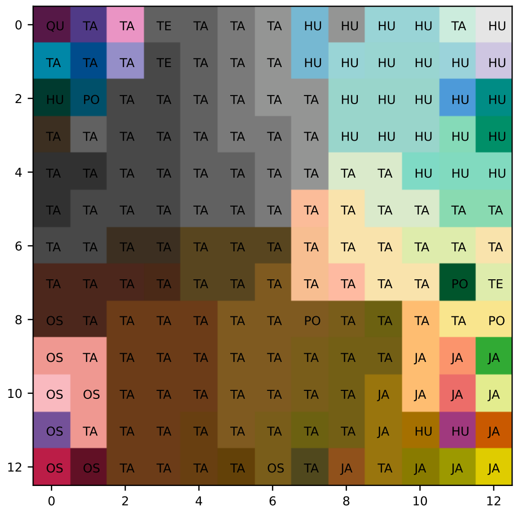

(**e**) Learnt SOM Cells

1.38. Carib; $|\mathcal{W}| = 19$

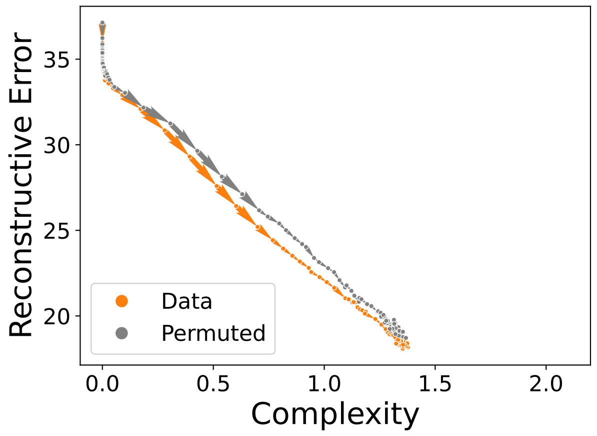

(**a**) Learning Space

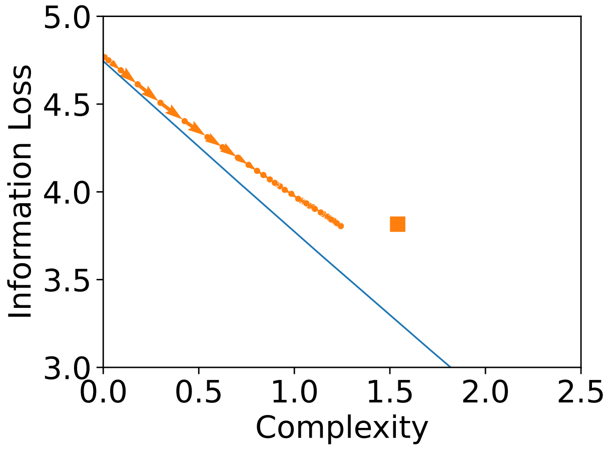

(**b**) Communicative Space

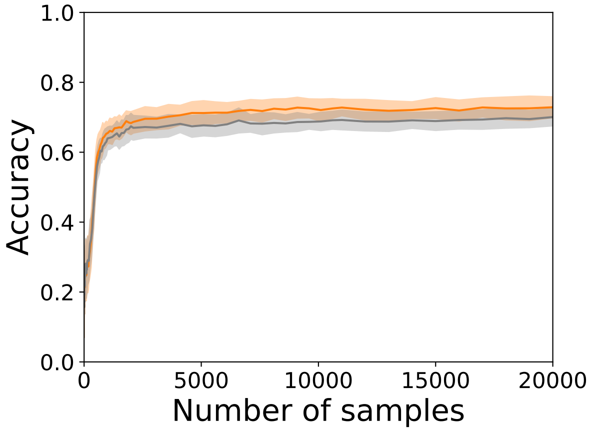

(**c**) Accuracy

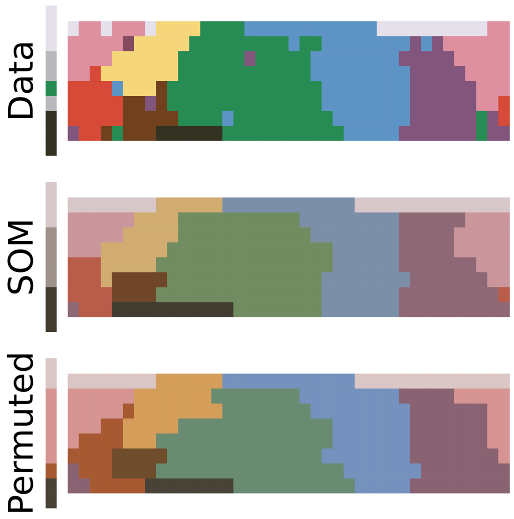

(**d**) Mode Maps

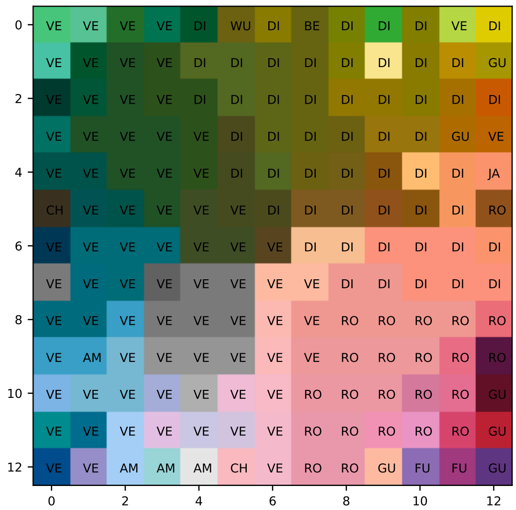

(**e**) Learnt SOM Cells

1.39. Guahibo; $|\mathcal{W}| = 15$

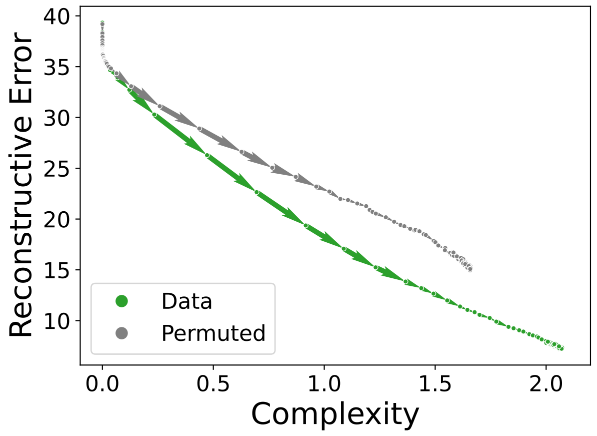

(**a**) Learning Space

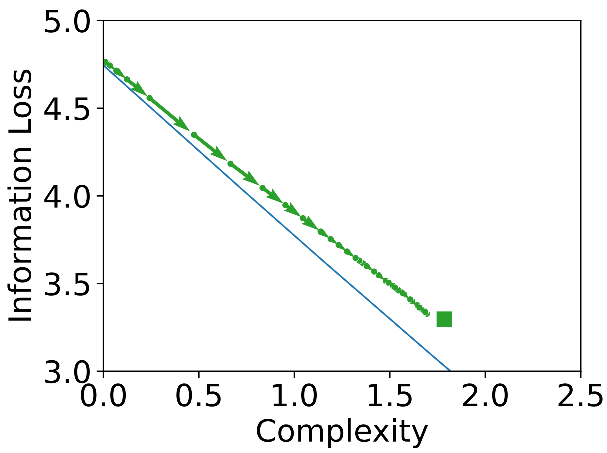

(**b**) Communicative Space

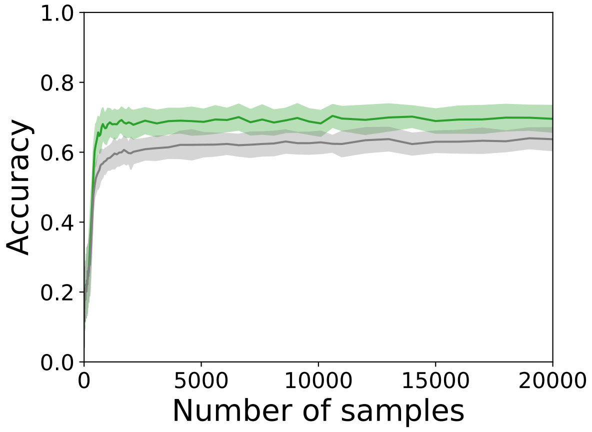

(**c**) Accuracy

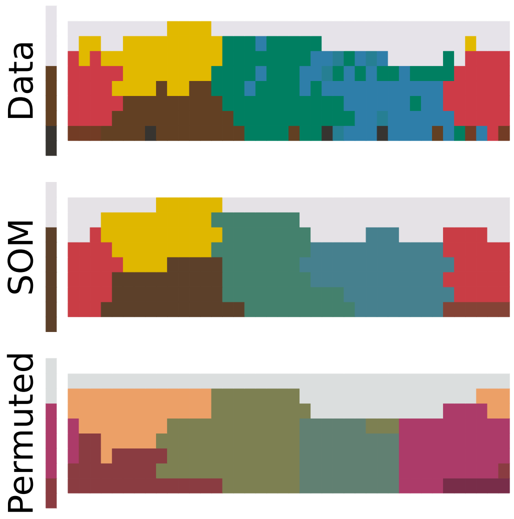

(**d**) Mode Maps

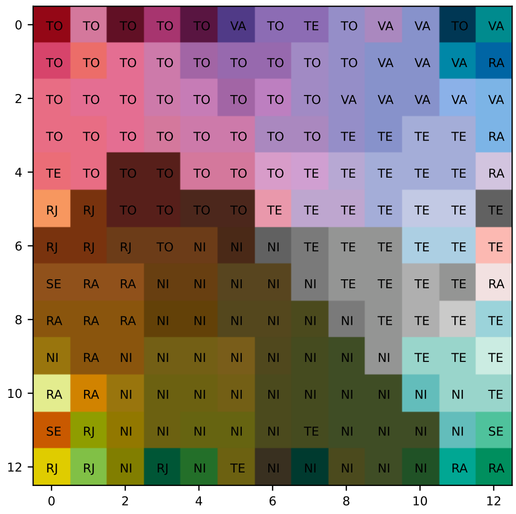

(**e**) Learnt SOM Cells

1.40. Guambiano; $|\mathcal{W}| = 9$

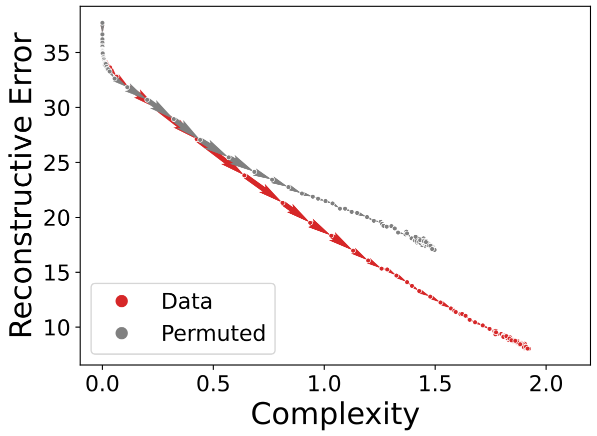

(**a**) Learning Space

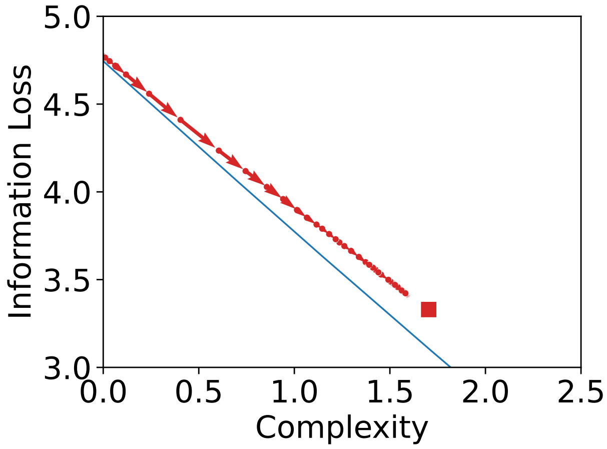

(**b**) Communicative Space

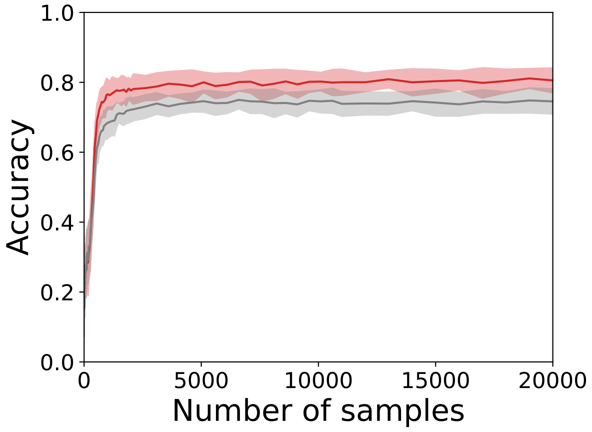

(**c**) Accuracy

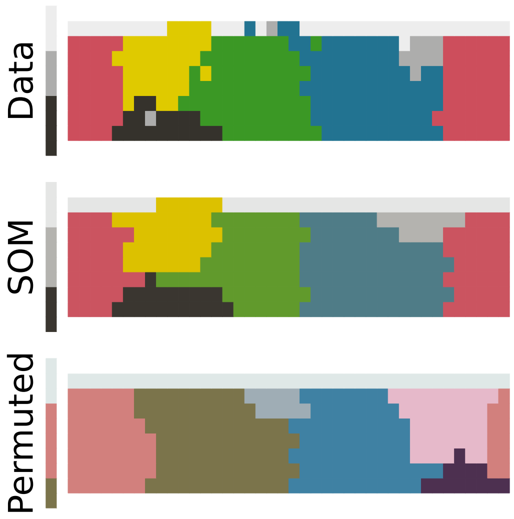

(**d**) Mode Maps

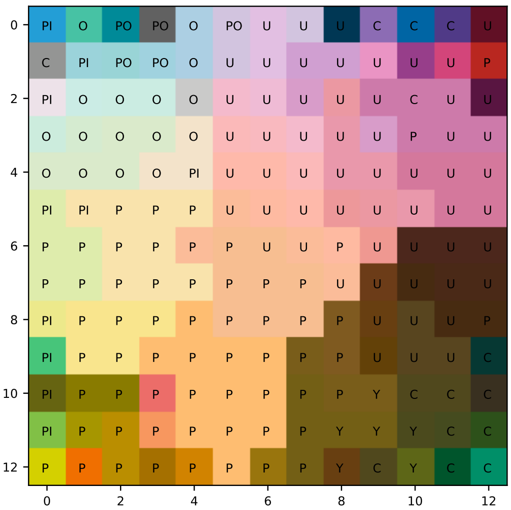

(**e**) Learnt SOM Cells

1.41. Guarijio; $|\mathcal{W}| = 13$

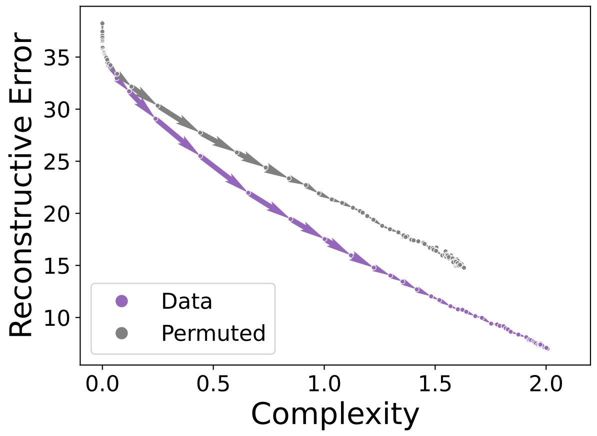

(**a**) Learning Space

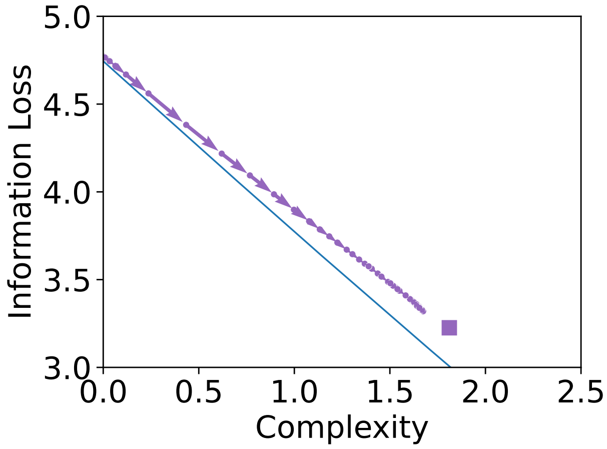

(**b**) Communicative Space

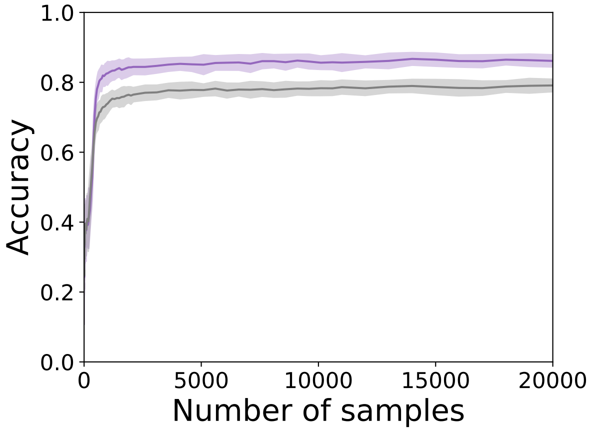

(**c**) Accuracy

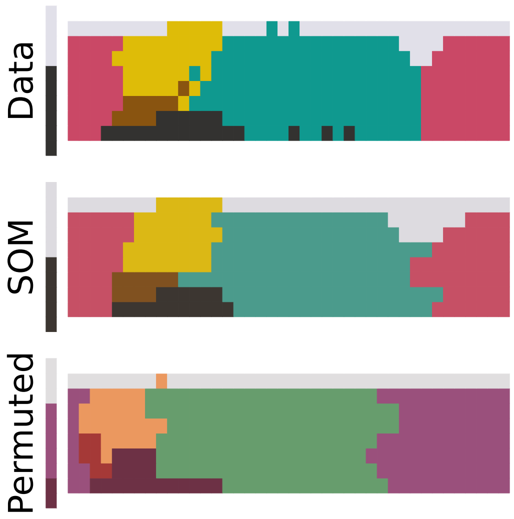

(**d**) Mode Maps

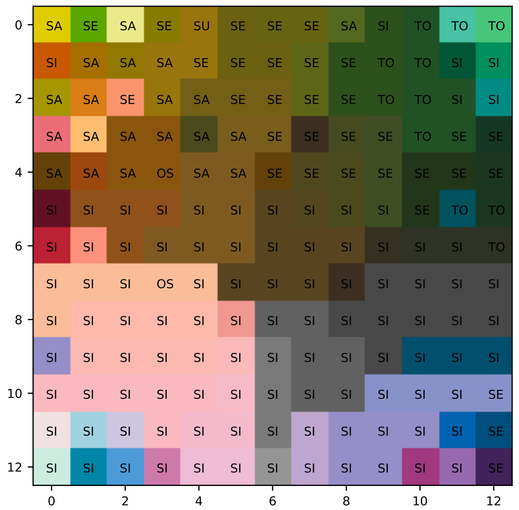

(**e**) Learnt SOM Cells

1.42. Ngäbere; $|\mathcal{W}| = 30$

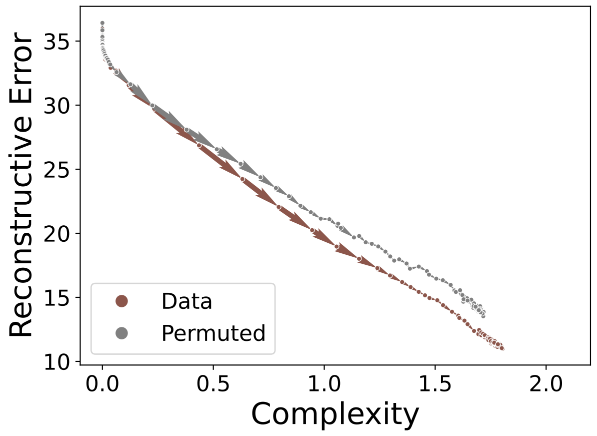

(**a**) Learning Space

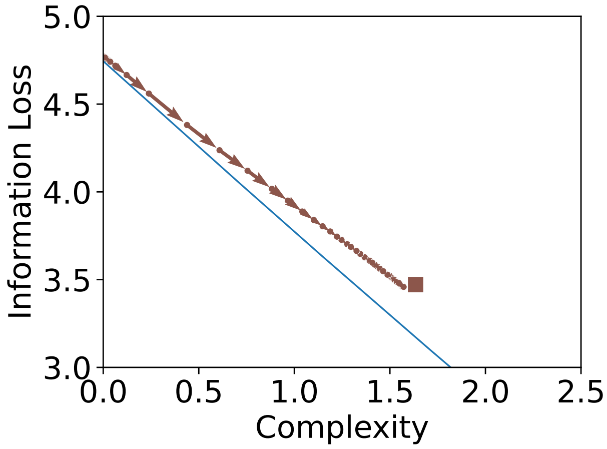

(**b**) Communicative Space

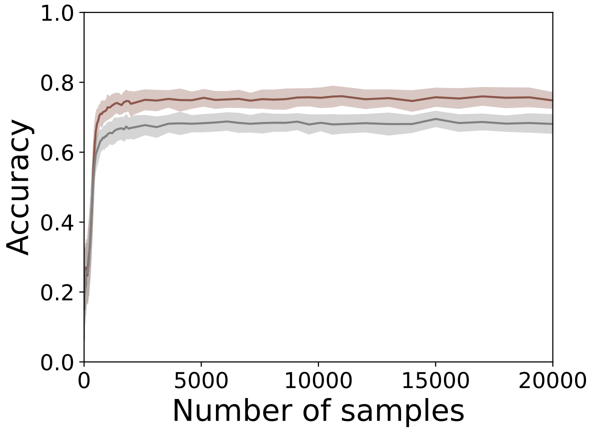

(**c**) Accuracy

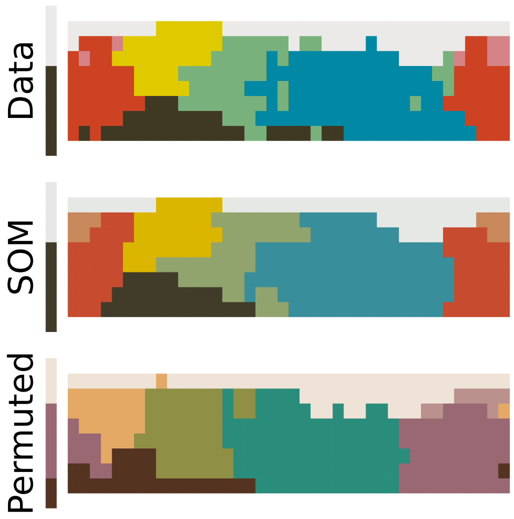

(**d**) Mode Maps

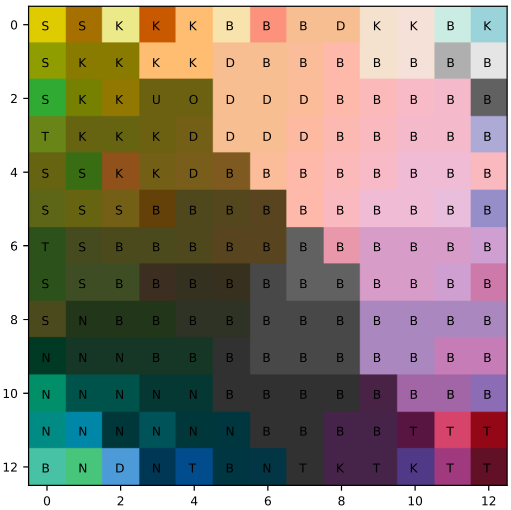

(**e**) Learnt SOM Cells

1.43. Gunu; $|\mathcal{W}| = 27$

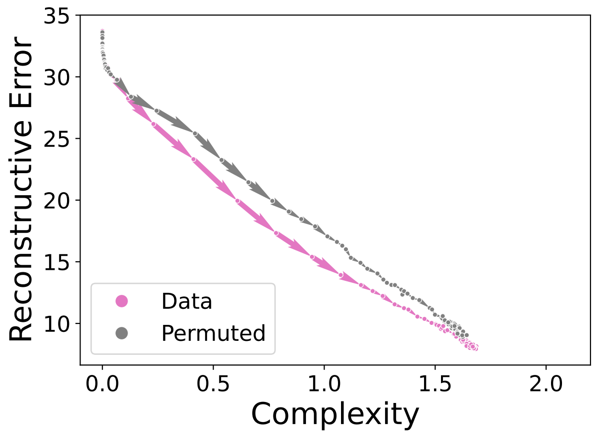

(**a**) Learning Space

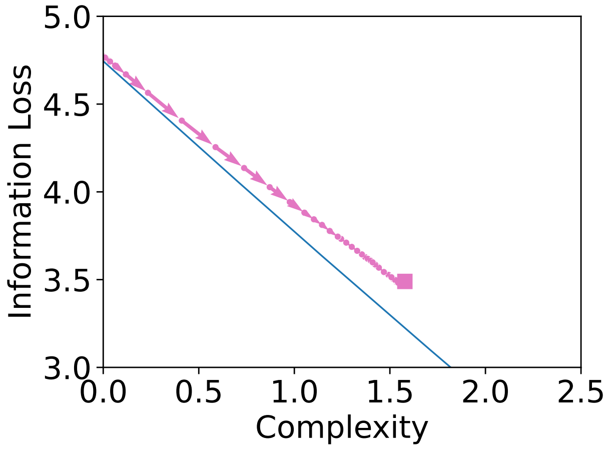

(**b**) Communicative Space

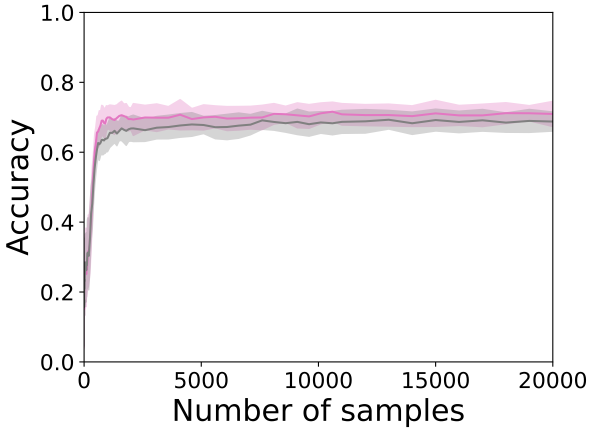

(**c**) Accuracy

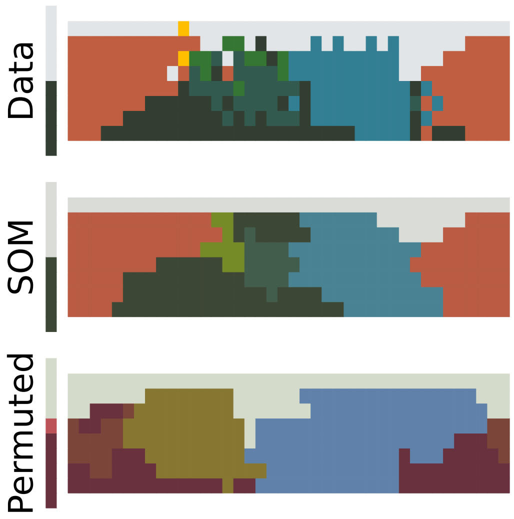

(**d**) Mode Maps

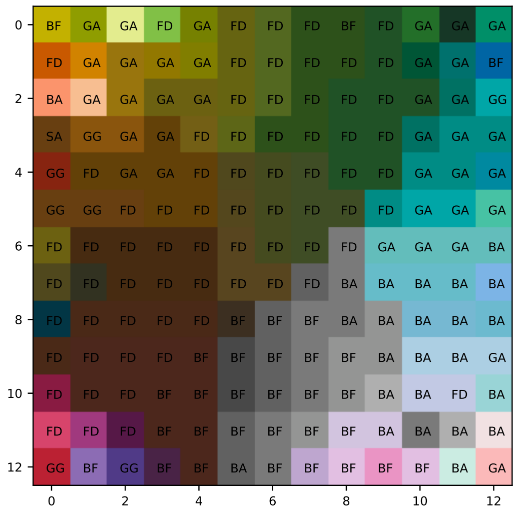

(**e**) Learnt SOM Cells

1.44. Halbi; $|\mathcal{W}| = 70$

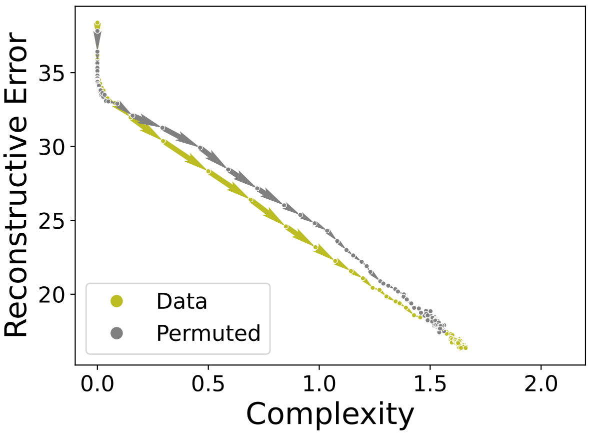

(**a**) Learning Space

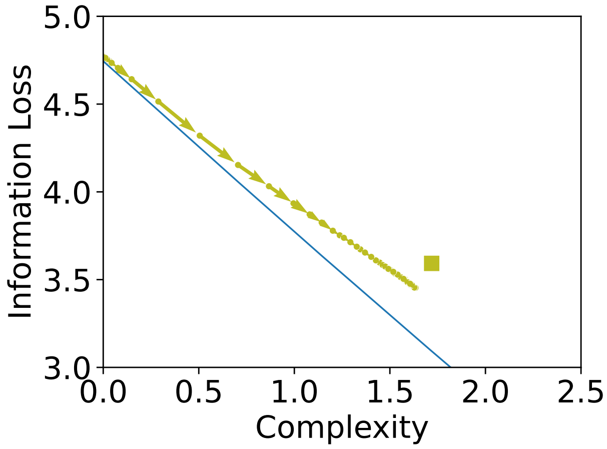

(**b**) Communicative Space

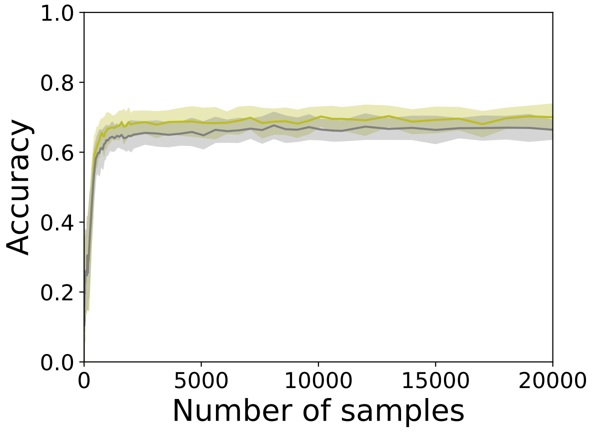

(**c**) Accuracy

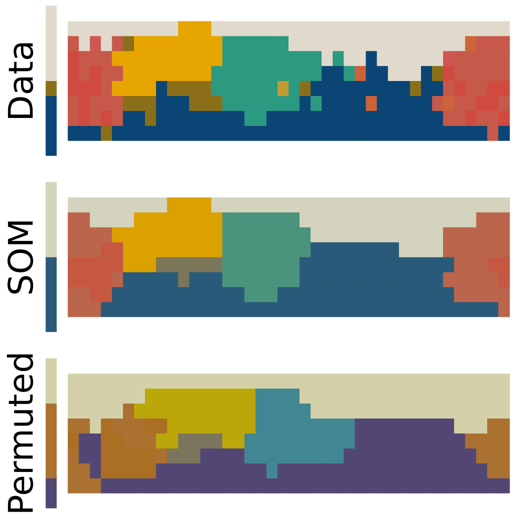

(**d**) Mode Maps

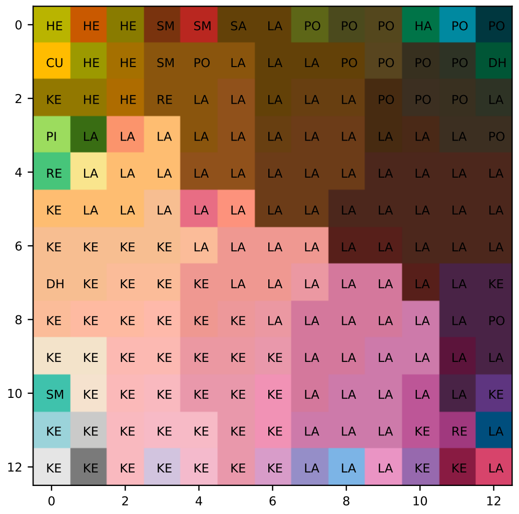

(**e**) Learnt SOM Cells

1.45. Huasteco; $|\mathcal{W}| = 13$

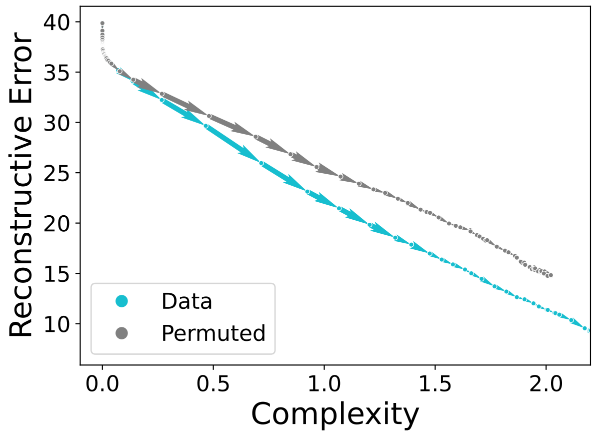

(**a**) Learning Space

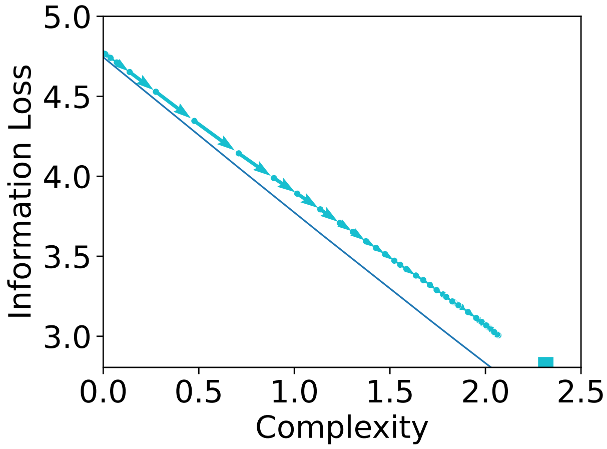

(**b**) Communicative Space

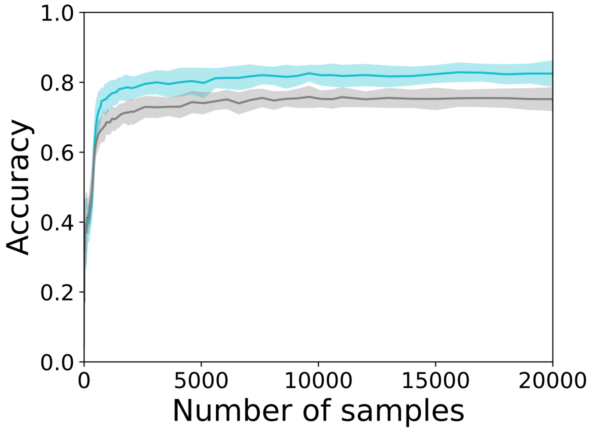

(**c**) Accuracy

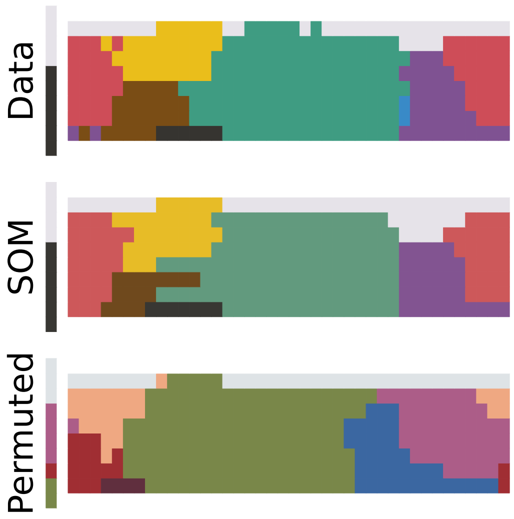

(**d**) Mode Maps

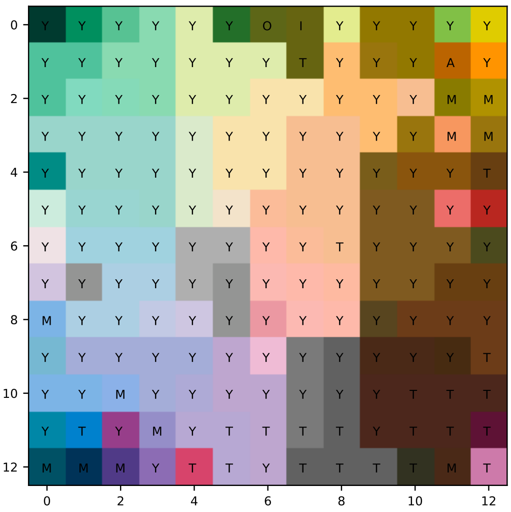

(**e**) Learnt SOM Cells

1.46. Huave; $|\mathcal{W}| = 17$

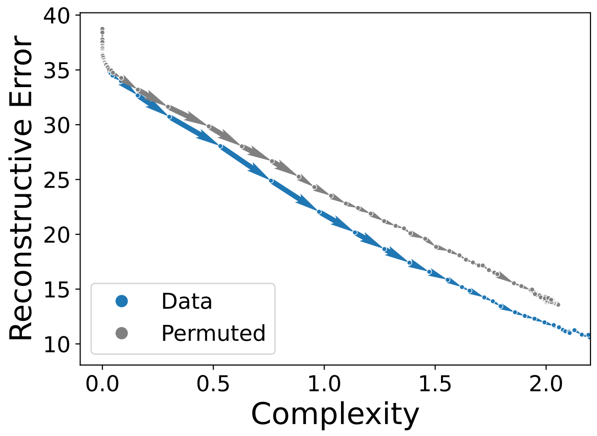

(**a**) Learning Space

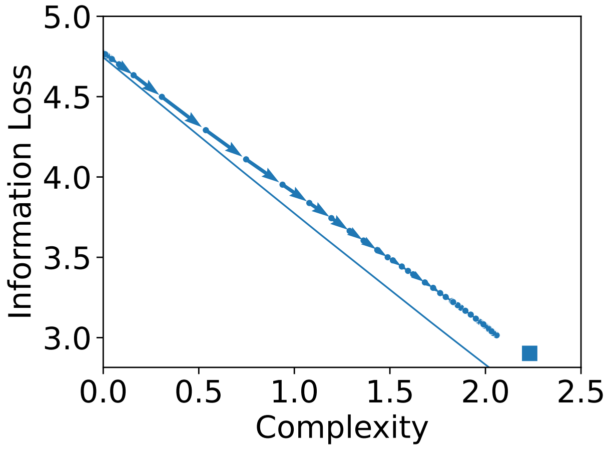

(**b**) Communicative Space

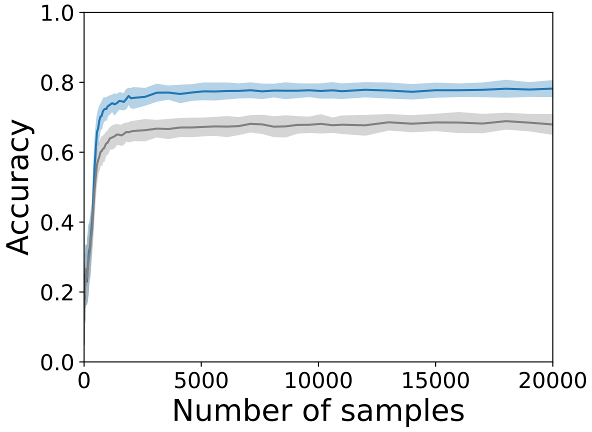

(**c**) Accuracy

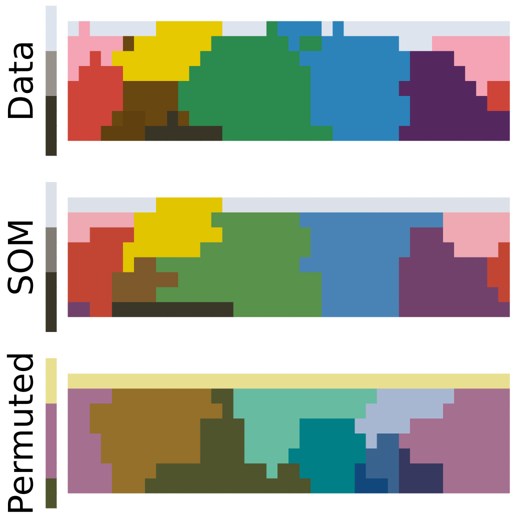

(**d**) Mode Maps

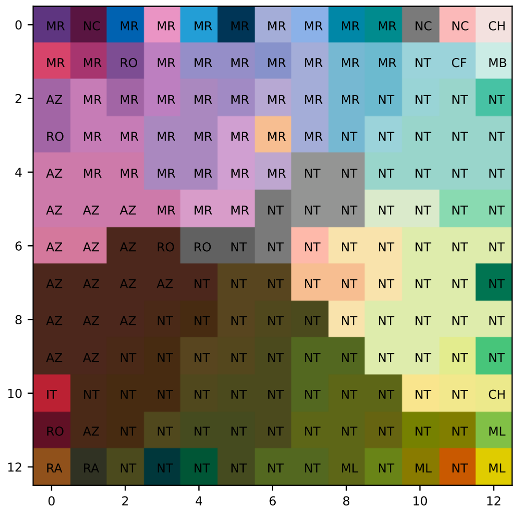

(**e**) Learnt SOM Cells

1.47. Iduna; $|\mathcal{W}| = 51$

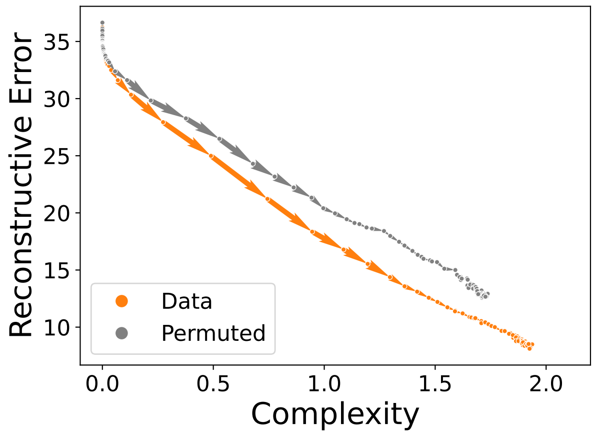

(**a**) Learning Space

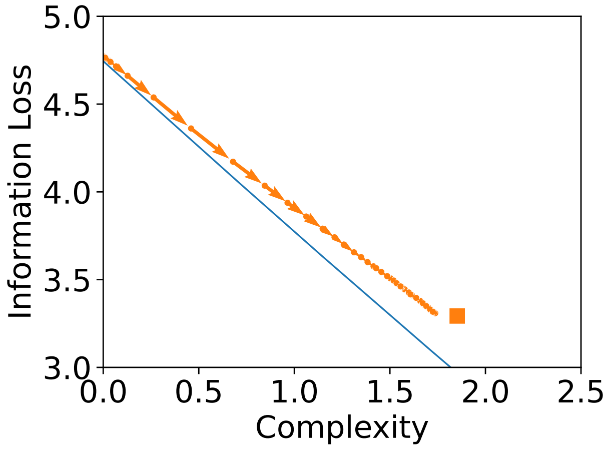

(**b**) Communicative Space

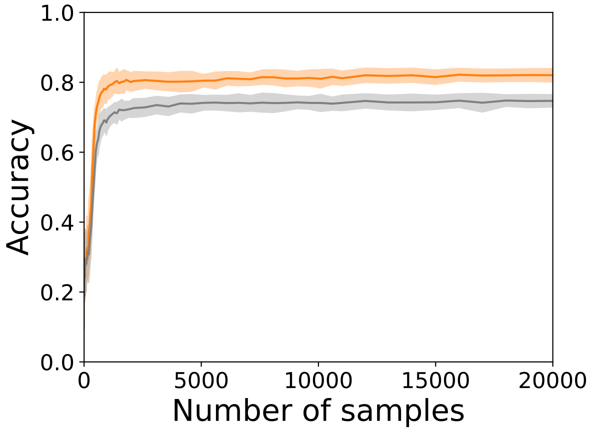

(**c**) Accuracy

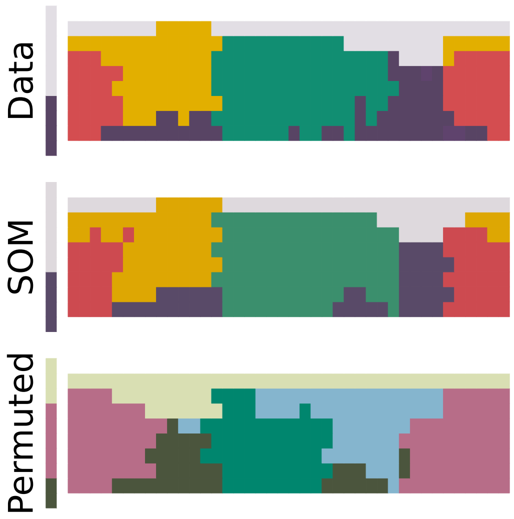

(**d**) Mode Maps

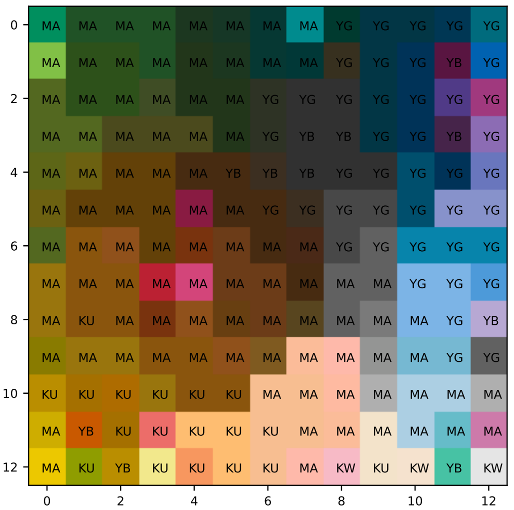

(**e**) Learnt SOM Cells

1.48. Ifugao; $|\mathcal{W}| = 23$

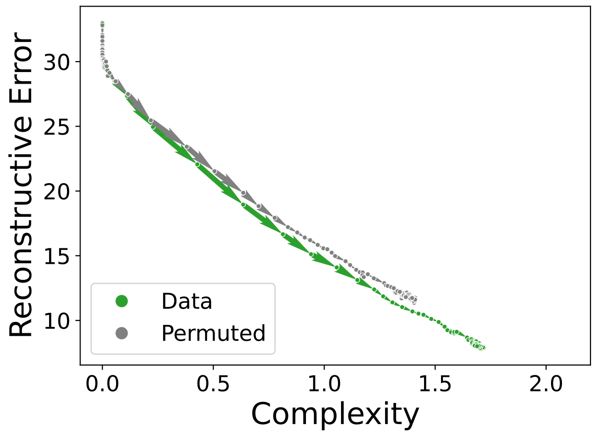

(**a**) Learning Space

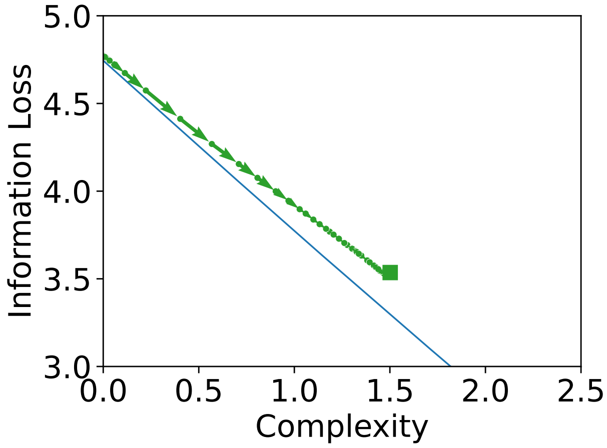

(**b**) Communicative Space

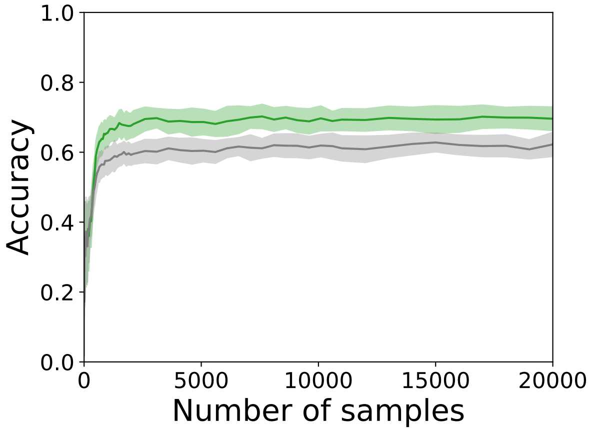

(**c**) Accuracy

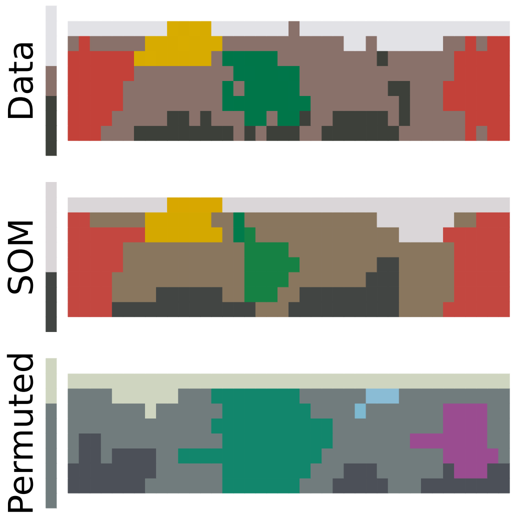

(**d**) Mode Maps

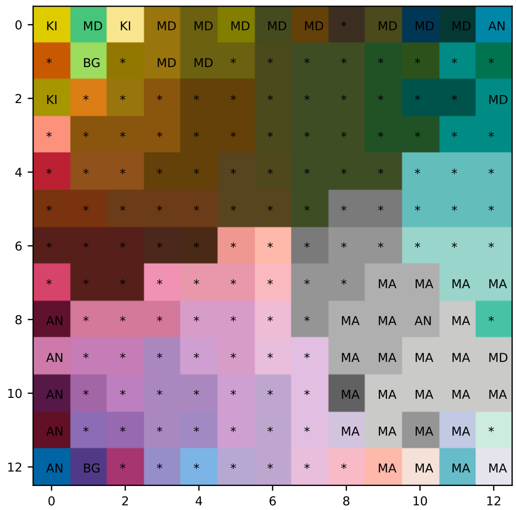

(**e**) Learnt SOM Cells

1.49. Sepik Iwam; $|\mathcal{W}| = 11$

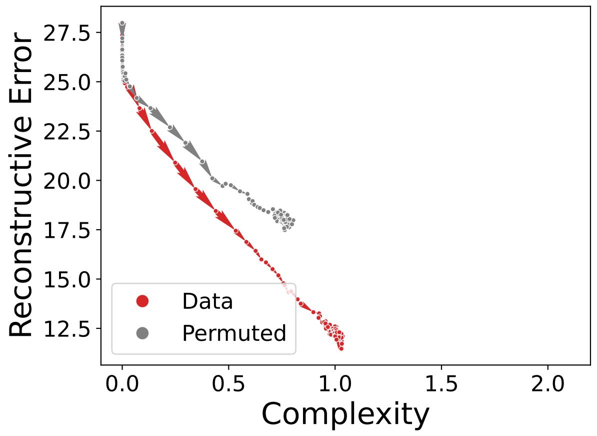

(**a**) Learning Space

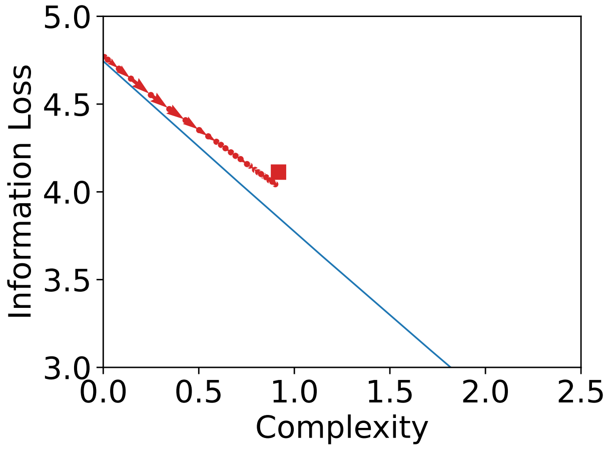

(**b**) Communicative Space

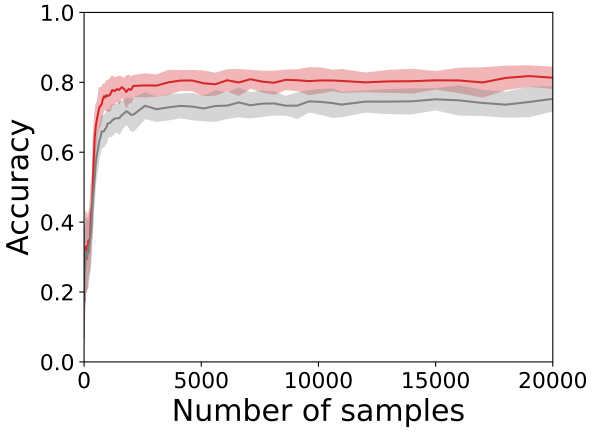

(**c**) Accuracy

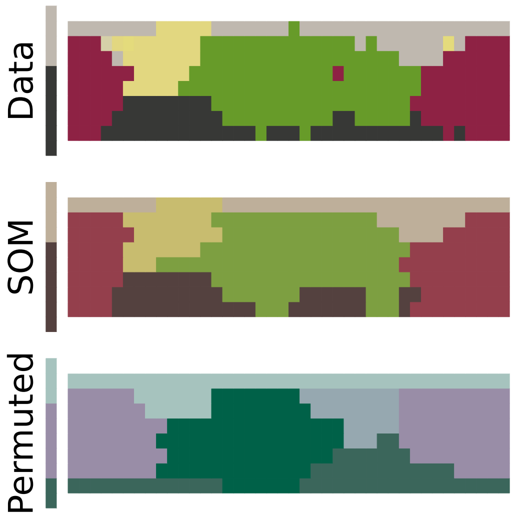

(**d**) Mode Maps

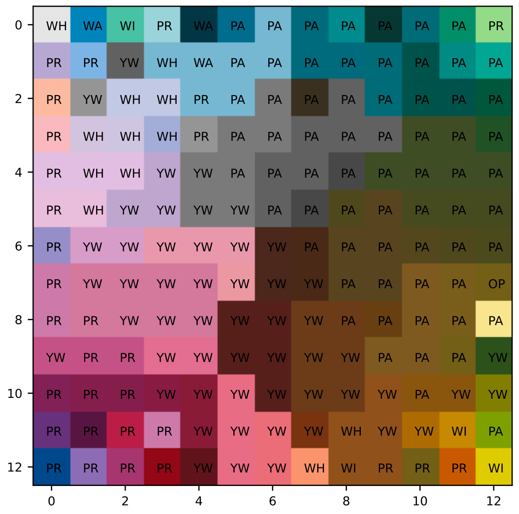

(**e**) Learnt SOM Cells

1.50. Jicaque; $|\mathcal{W}| = 10$

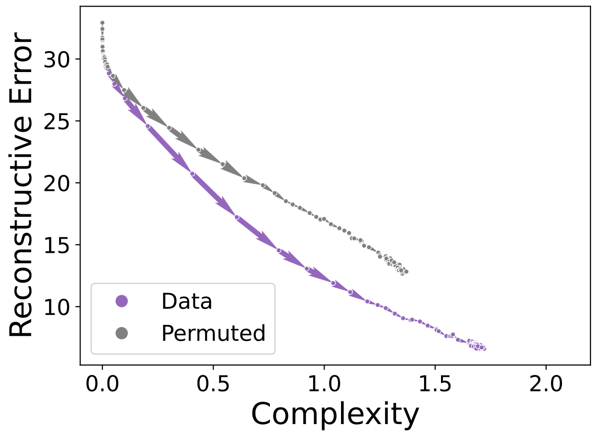

(**a**) Learning Space

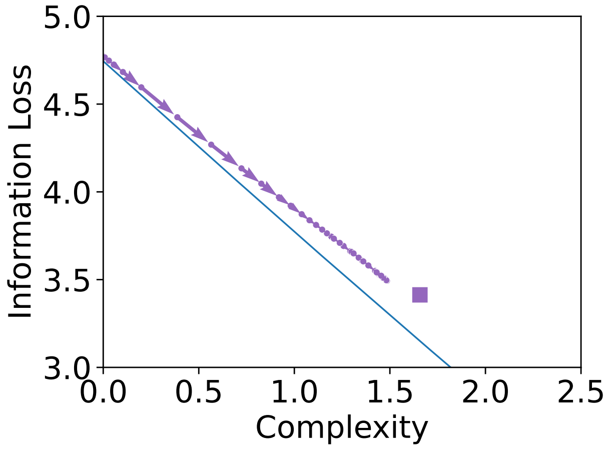

(**b**) Communicative Space

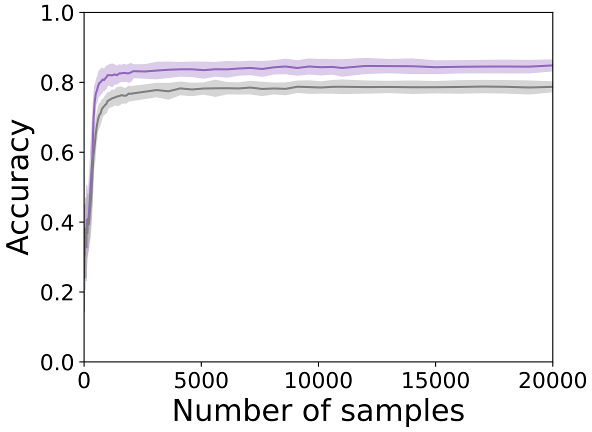

(**c**) Accuracy

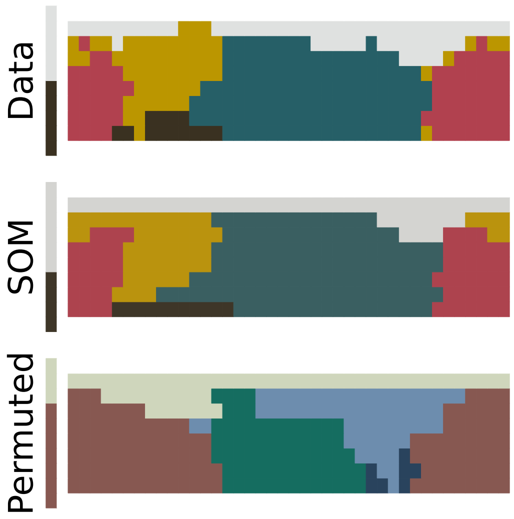

(**d**) Mode Maps

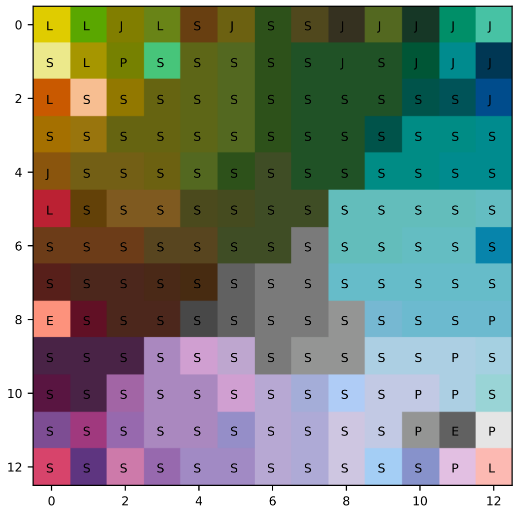

(**e**) Learnt SOM Cells

1.51. Kalam; $|\mathcal{W}| = 67$

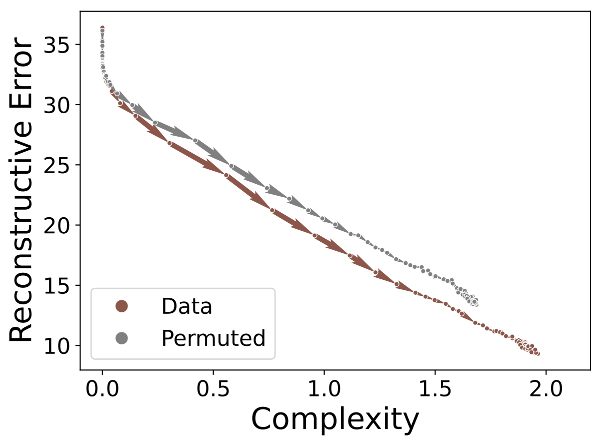

(**a**) Learning Space

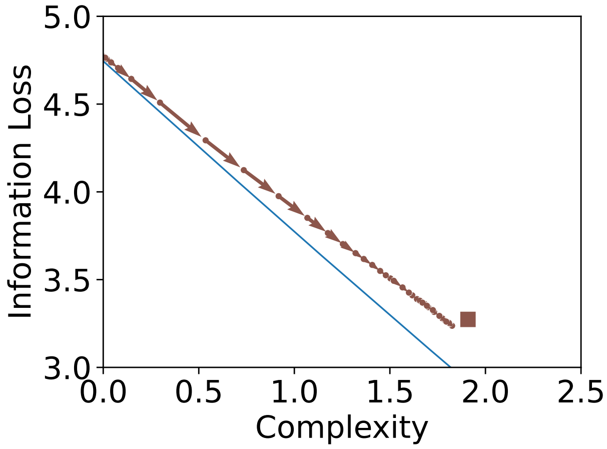

(**b**) Communicative Space

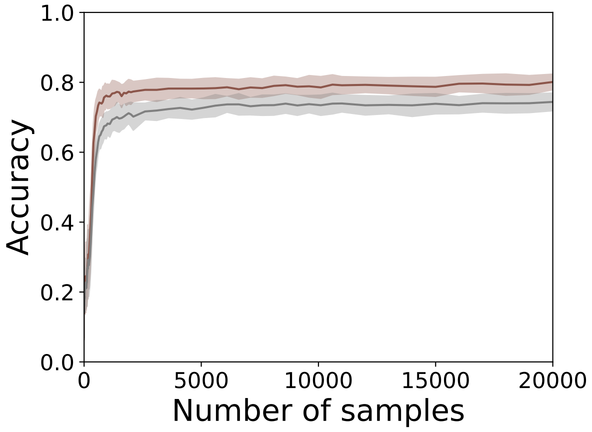

(**c**) Accuracy

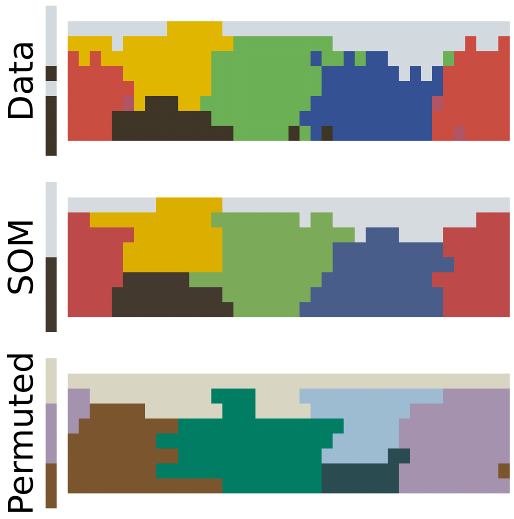

(**d**) Mode Maps

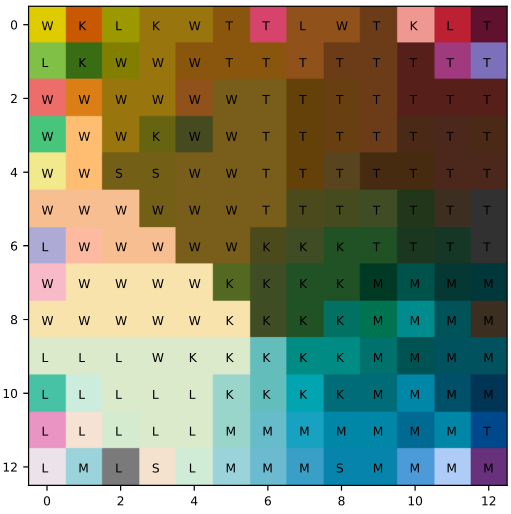

(**e**) Learnt SOM Cells

1.52. Kamano-Kafe; $|\mathcal{W}| = 33$

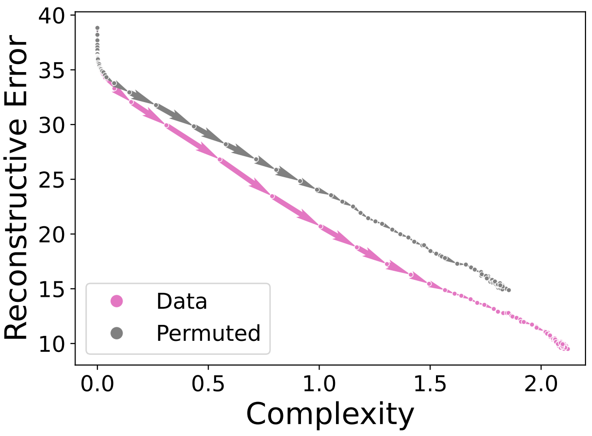

(**a**) Learning Space

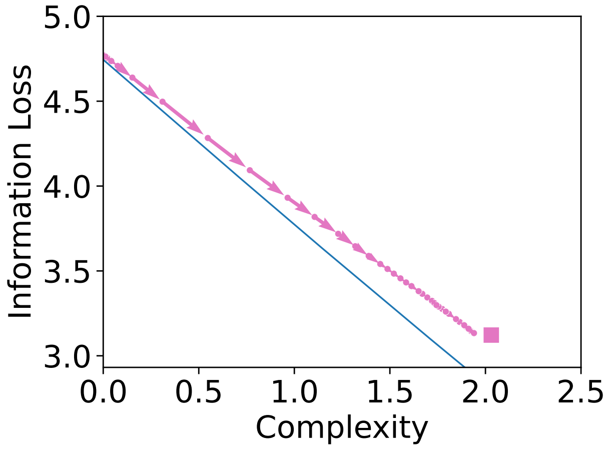

(**b**) Communicative Space

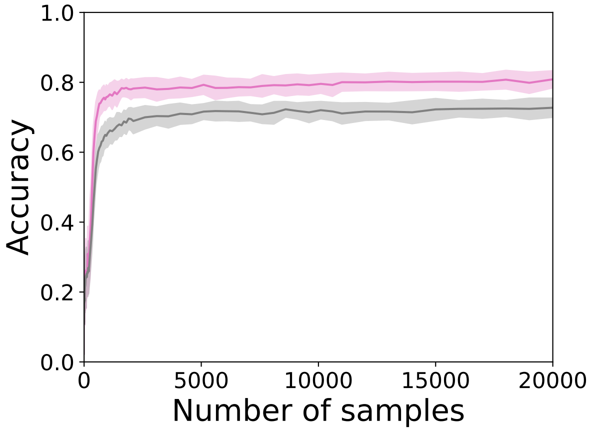

(**c**) Accuracy

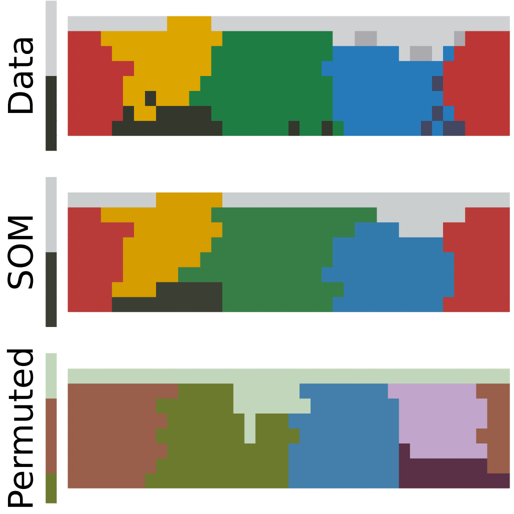

(**d**) Mode Maps

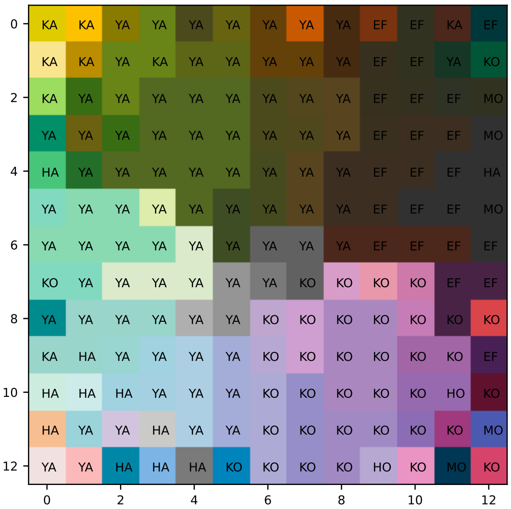

(**e**) Learnt SOM Cells

1.53. Karaja; $|\mathcal{W}| = 10$

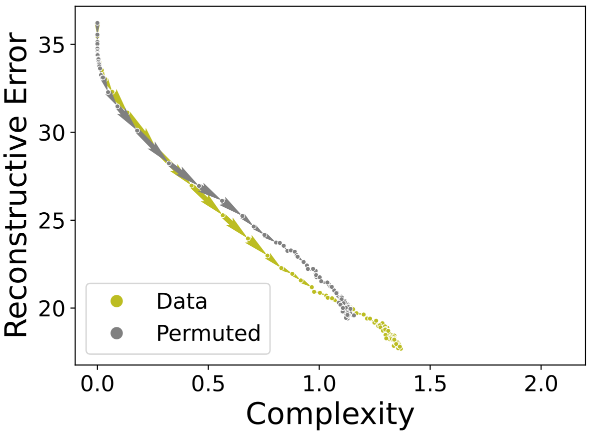

(**a**) Learning Space

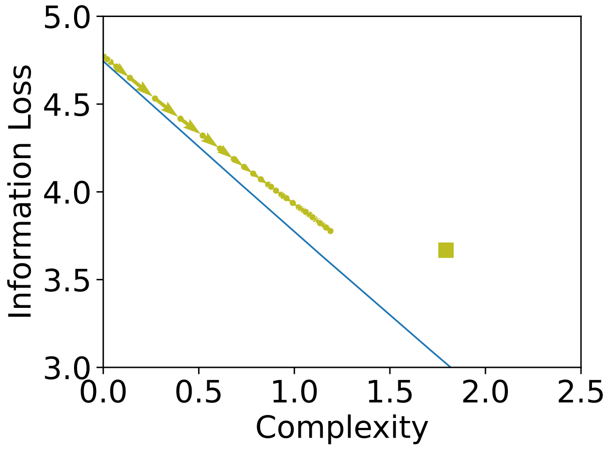

(**b**) Communicative Space

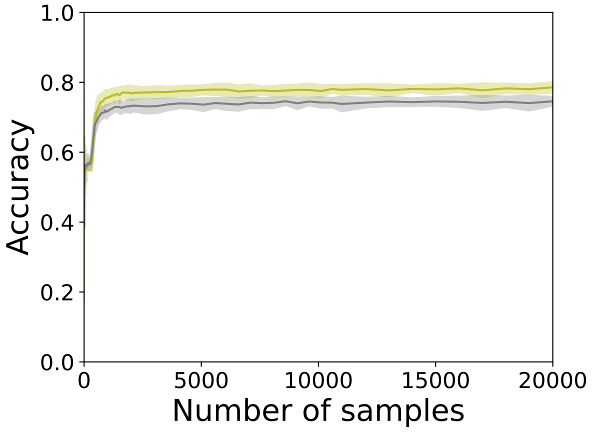

(**c**) Accuracy

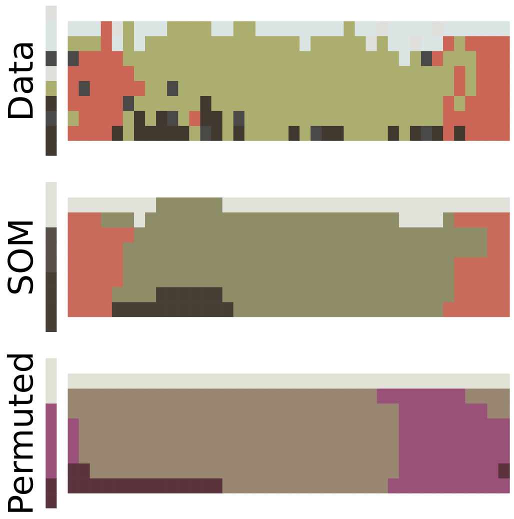

(**d**) Mode Maps

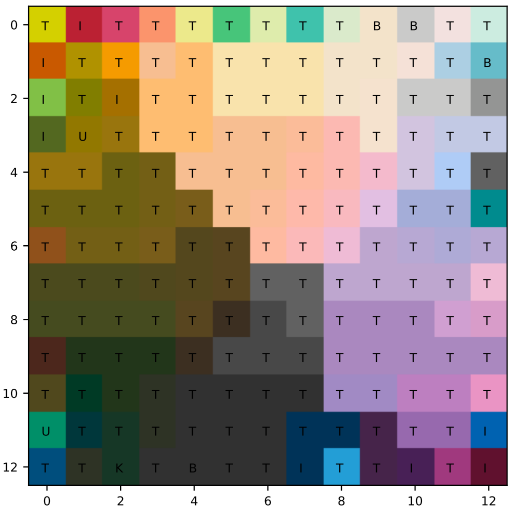

(**e**) Learnt SOM Cells

1.54. Kemtuik; $|\mathcal{W}| = 25$

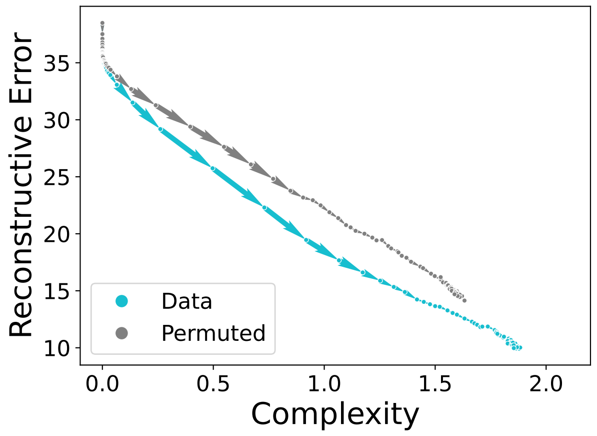

(**a**) Learning Space

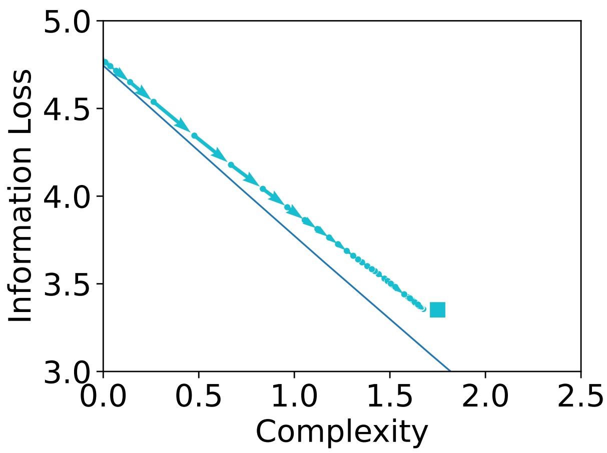

(**b**) Communicative Space

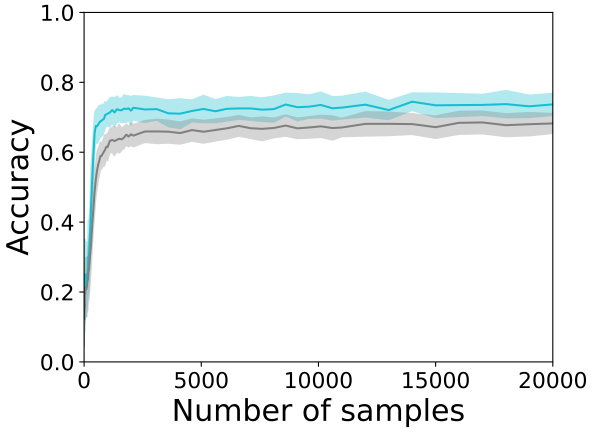

(**c**) Accuracy

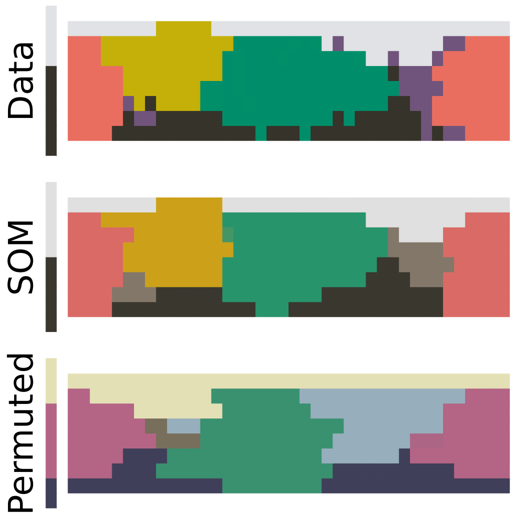

(**d**) Mode Maps

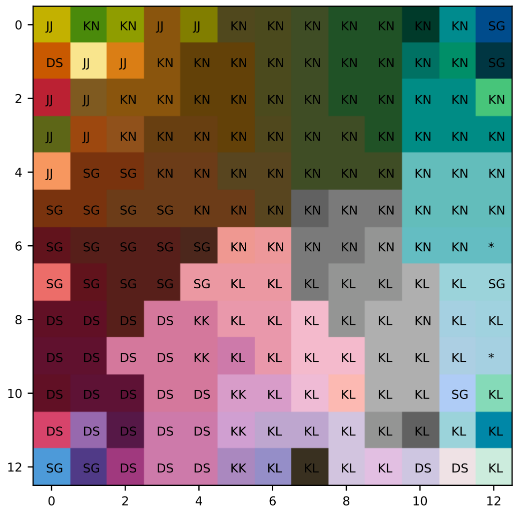

(**e**) Learnt SOM Cells

1.55. Kokoni; $|\mathcal{W}| = 11$

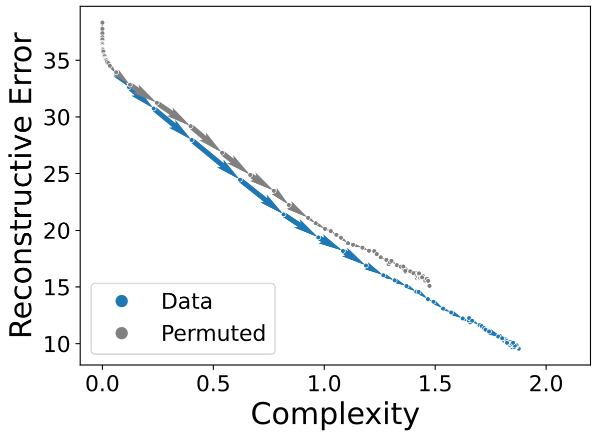

(**a**) Learning Space

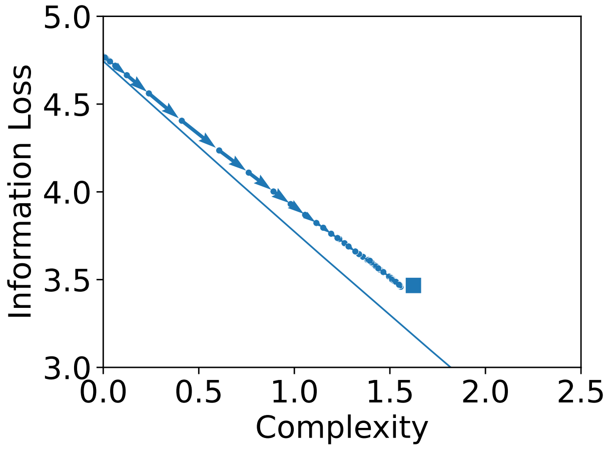

(**b**) Communicative Space

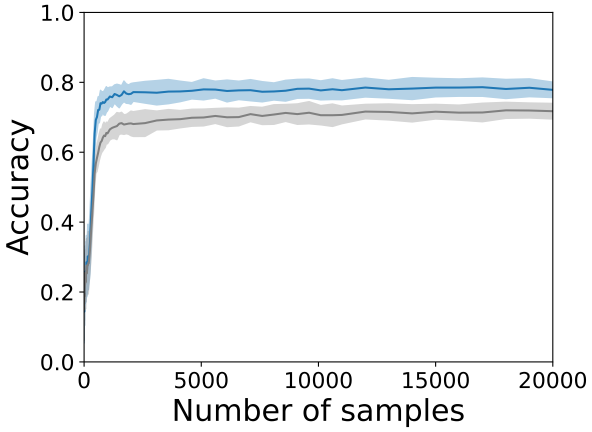

(**c**) Accuracy

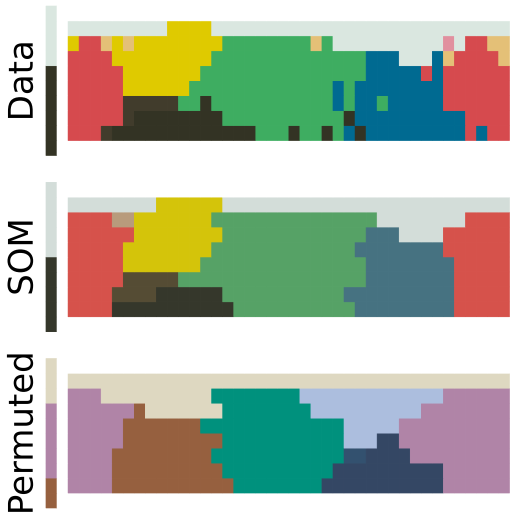

(**d**) Mode Maps

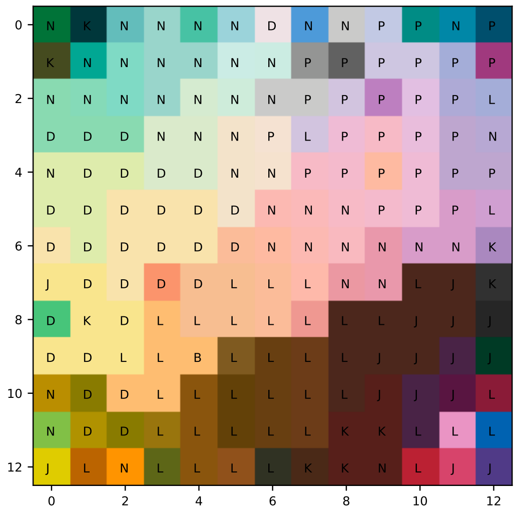

(**e**) Learnt SOM Cells

1.56. Konkomba; $|\mathcal{W}| = 21$

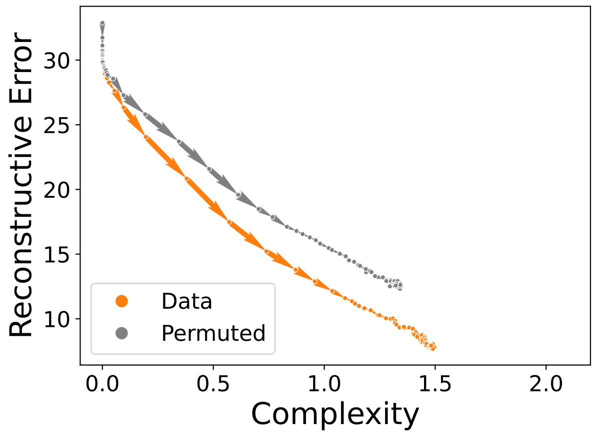

(**a**) Learning Space

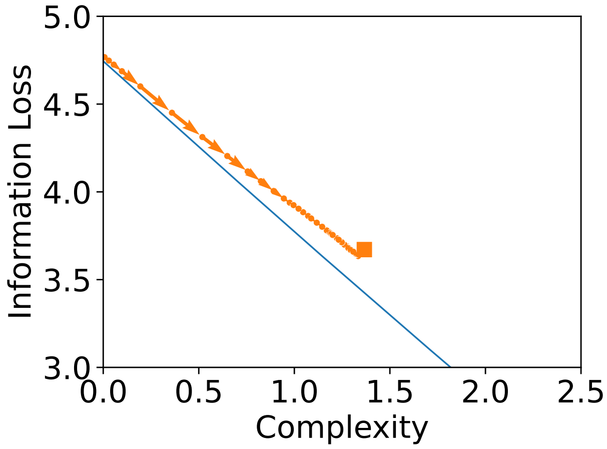

(**b**) Communicative Space

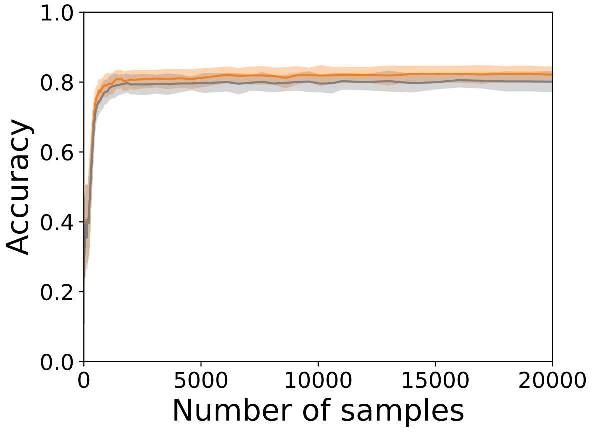

(**c**) Accuracy

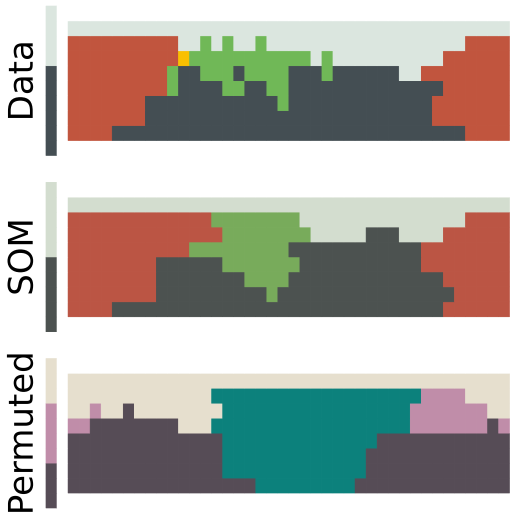

(**d**) Mode Maps

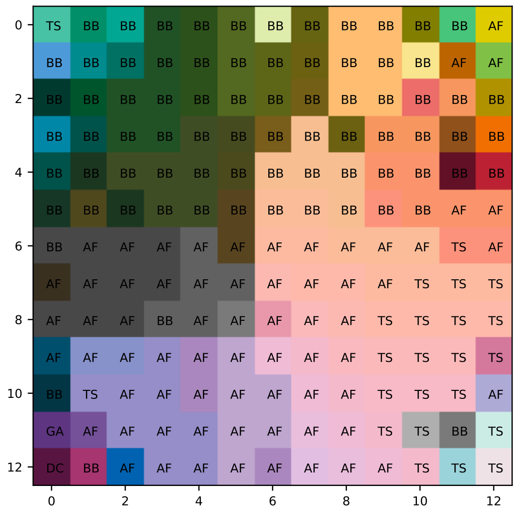

(**e**) Learnt SOM Cells

1.57. Kriol; $|\mathcal{W}| = 25$

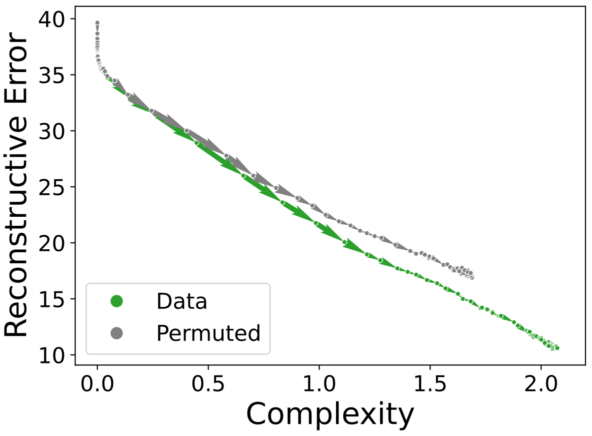

(**a**) Learning Space

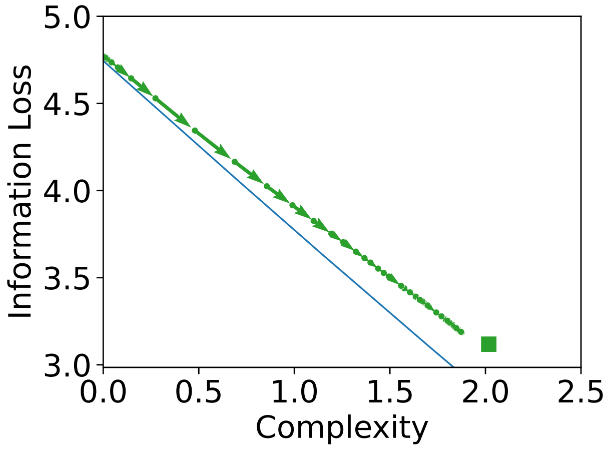

(**b**) Communicative Space

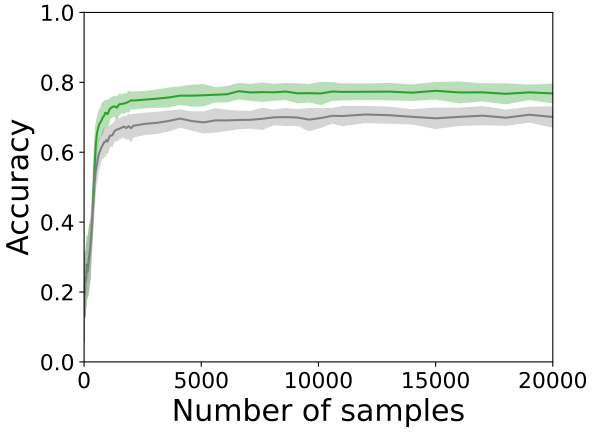

(**c**) Accuracy

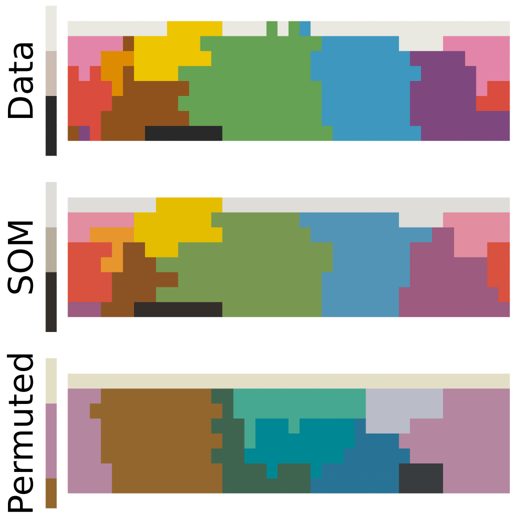

(**d**) Mode Maps

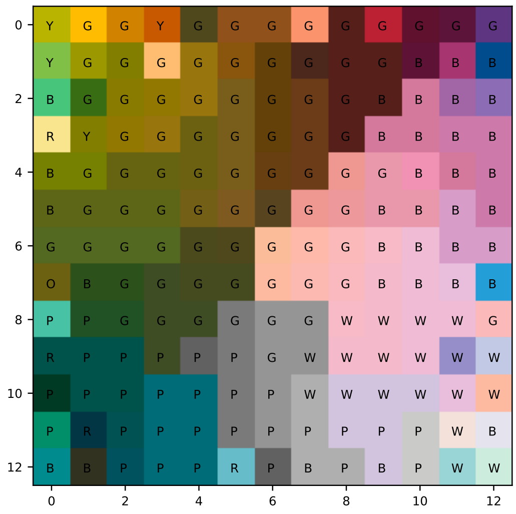

(**e**) Learnt SOM Cells

1.58. Kuku-Yalanji; $|\mathcal{W}| = 17$

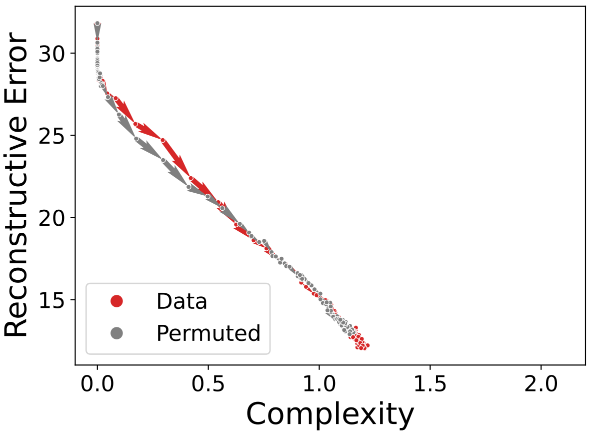

(**a**) Learning Space

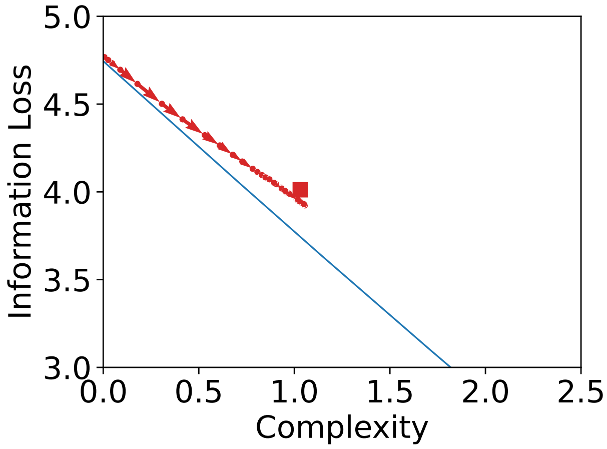

(**b**) Communicative Space

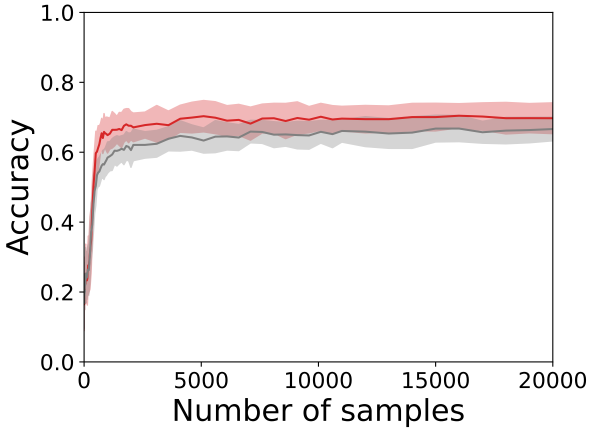

(**c**) Accuracy

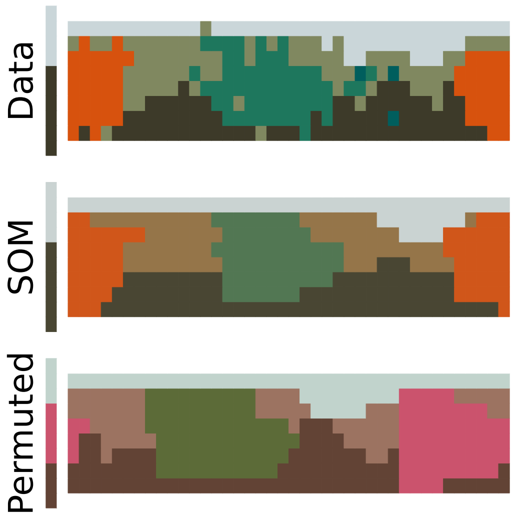

(**d**) Mode Maps

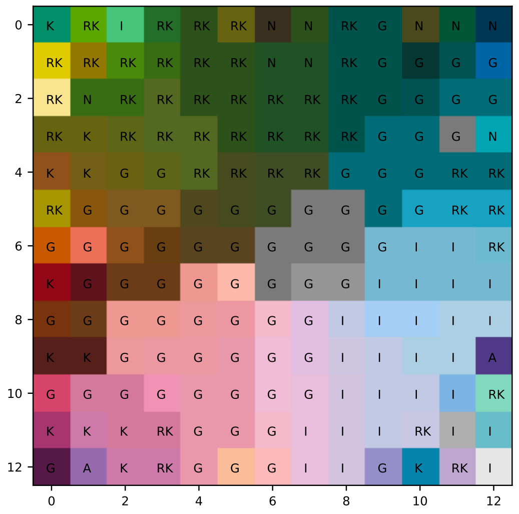

(**e**) Learnt SOM Cells

1.59. Long-haired Kuna; $|\mathcal{W}| = 46$

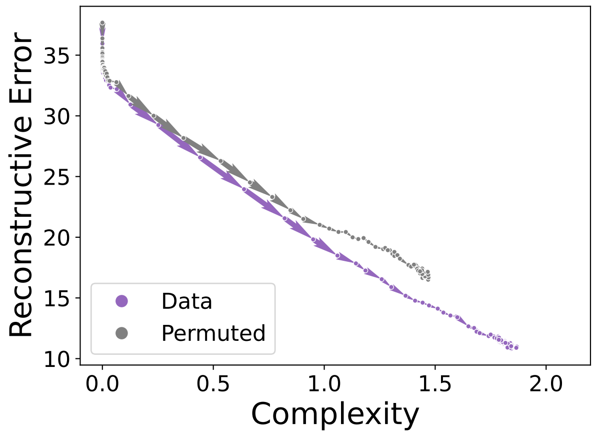

(**a**) Learning Space

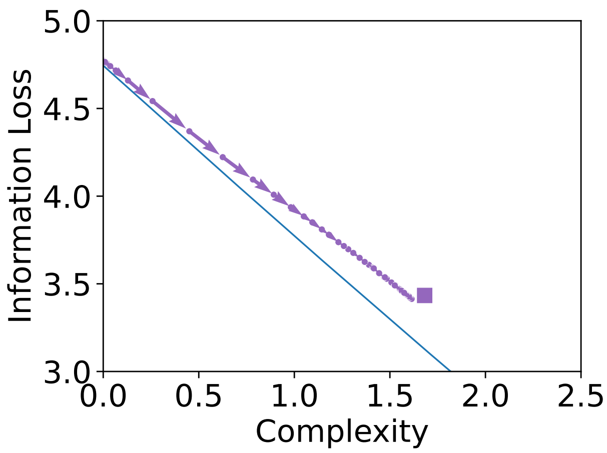

(**b**) Communicative Space

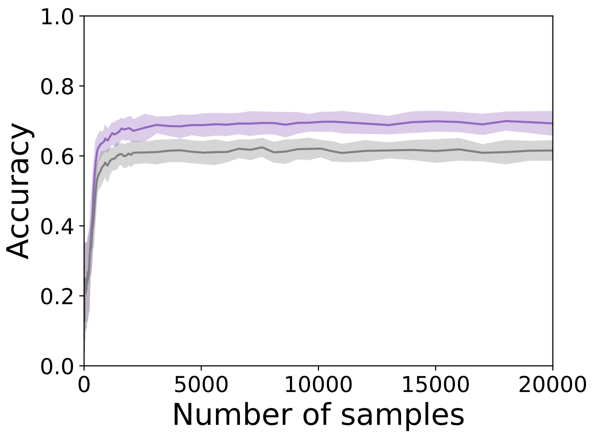

(**c**) Accuracy

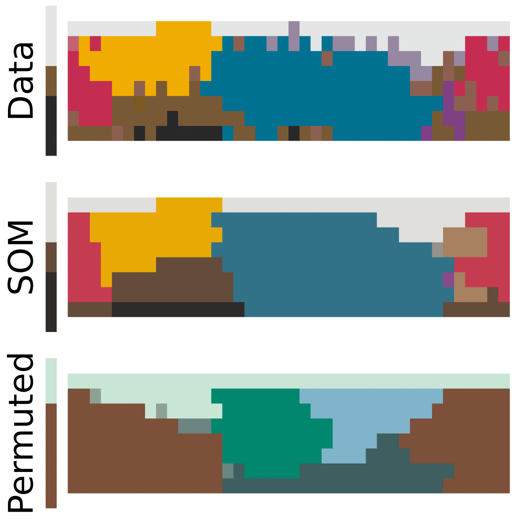

(**d**) Mode Maps

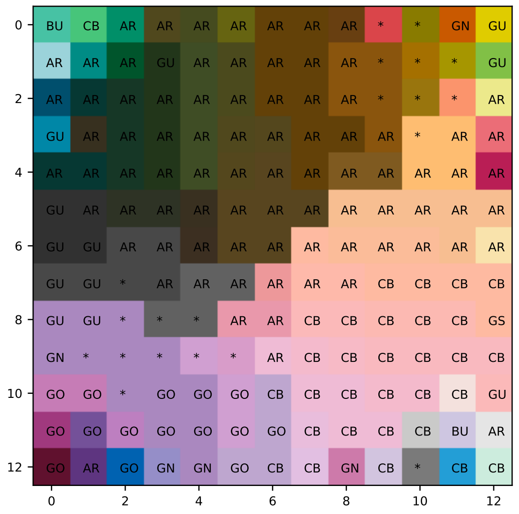

(**e**) Learnt SOM Cells

1.60. Kwerba; $|\mathcal{W}| = 22$

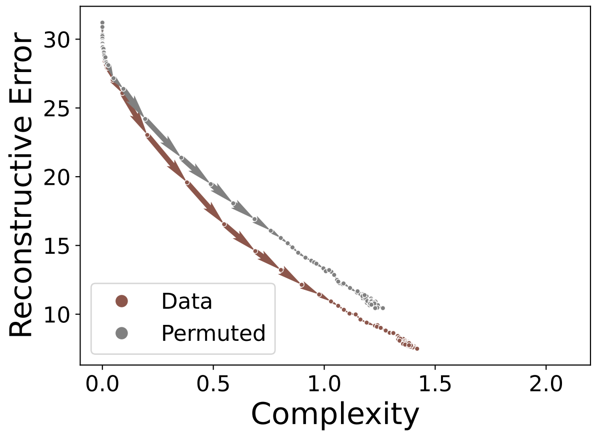

(**a**) Learning Space

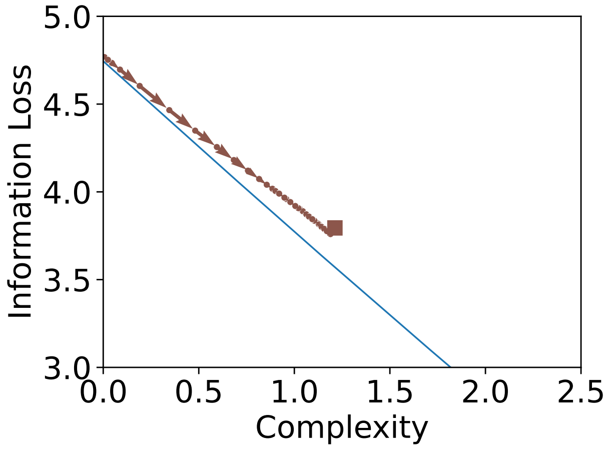

(**b**) Communicative Space

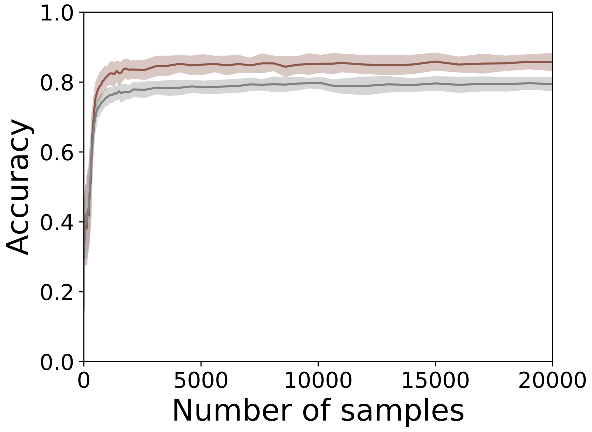

(**c**) Accuracy

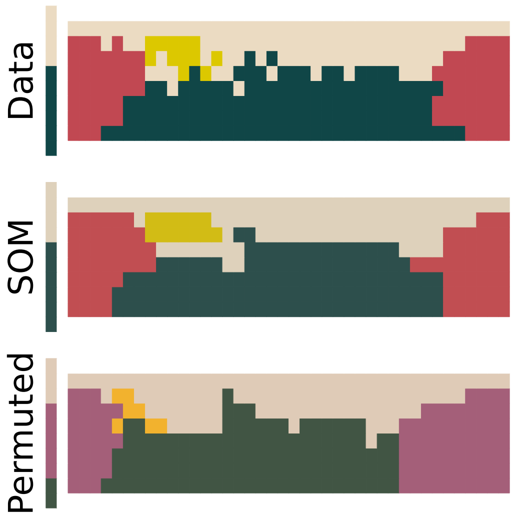

(**d**) Mode Maps

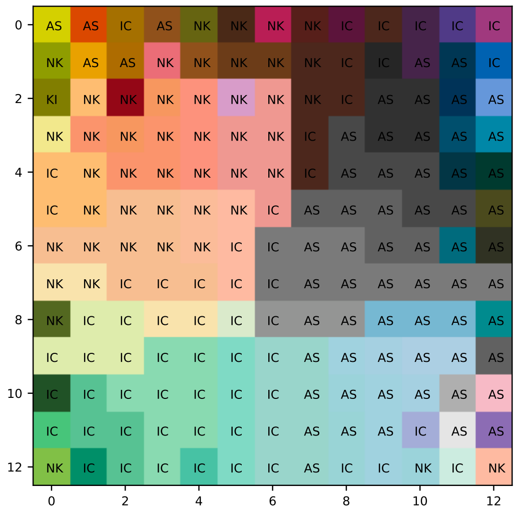

(**e**) Learnt SOM Cells

1.61. Lele; $|\mathcal{W}| = 20$

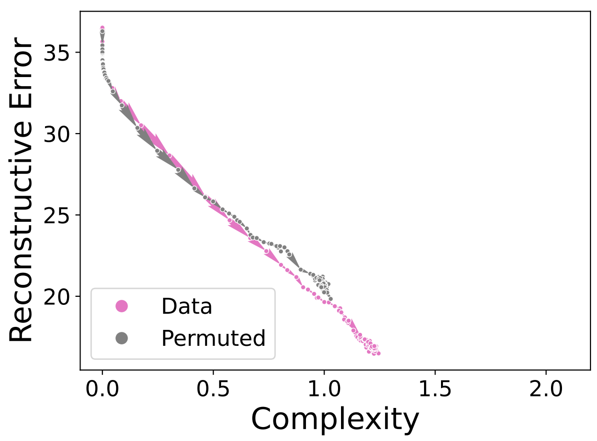

(**a**) Learning Space

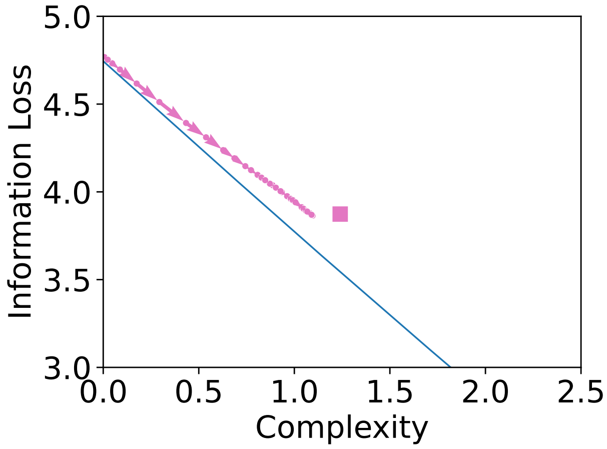

(**b**) Communicative Space

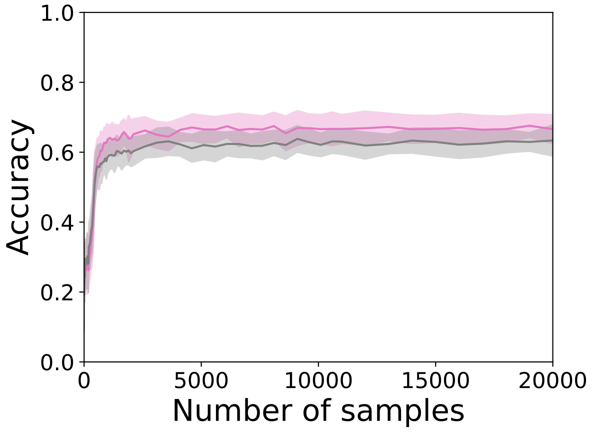

(**c**) Accuracy

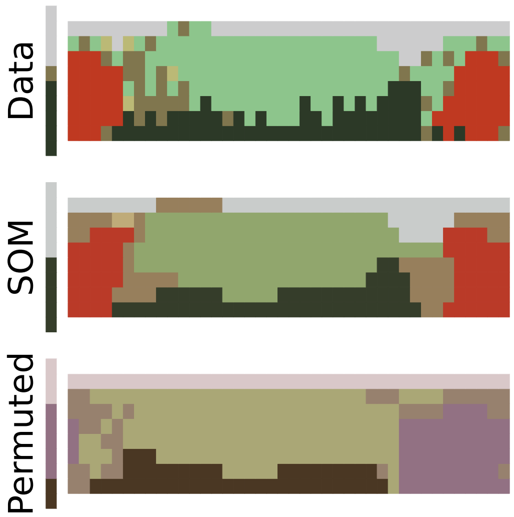

(**d**) Mode Maps

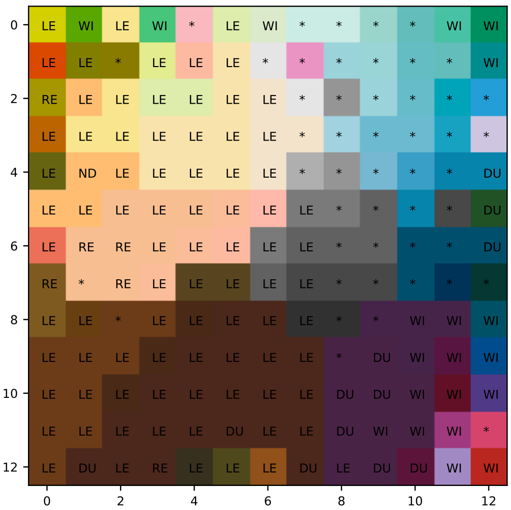

(**e**) Learnt SOM Cells

1.62. Mampruli; $|\mathcal{W}| = 80$

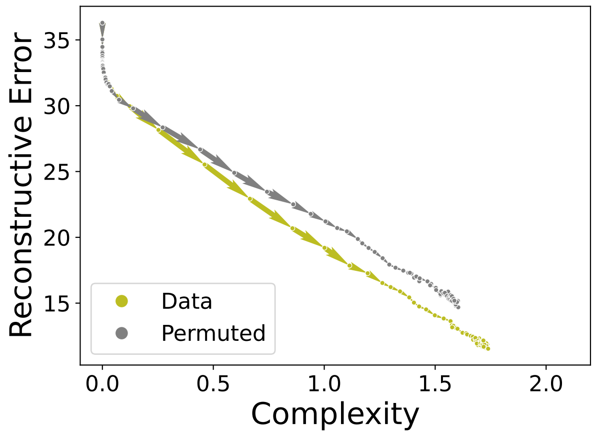

(**a**) Learning Space

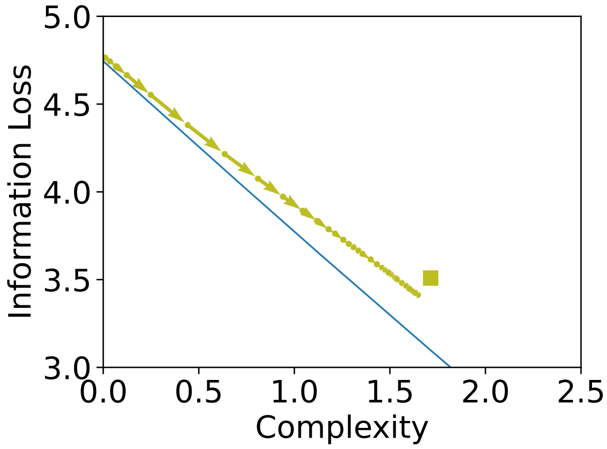

(**b**) Communicative Space

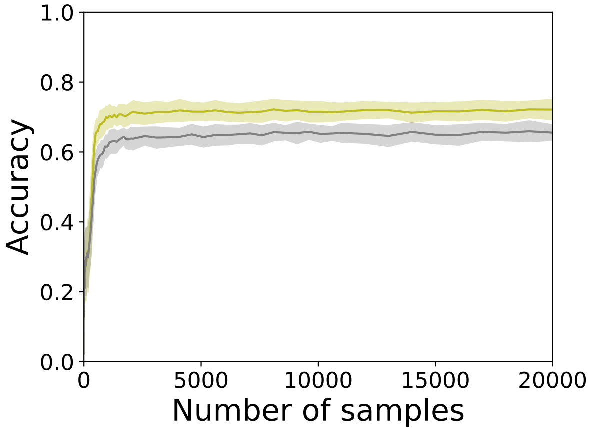

(**c**) Accuracy

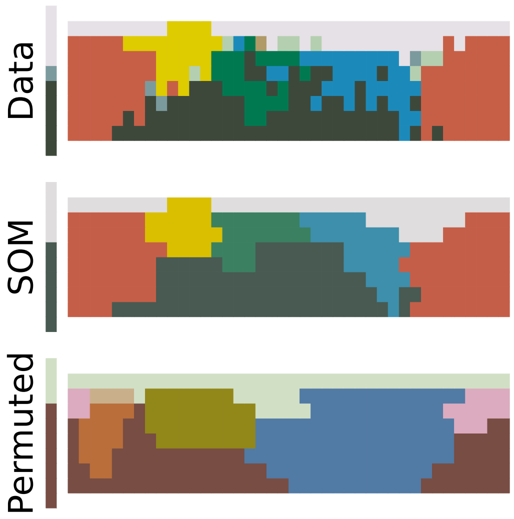

(**d**) Mode Maps

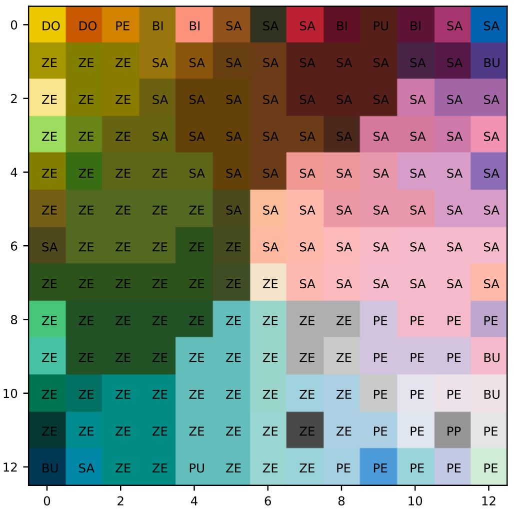

(**e**) Learnt SOM Cells

1.63. Maring; $|\mathcal{W}| = 33$

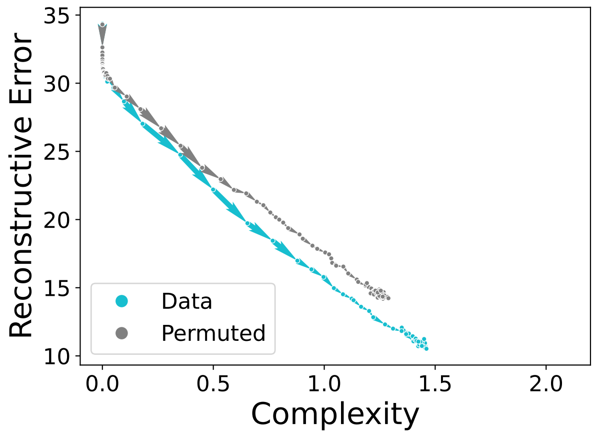

(**a**) Learning Space

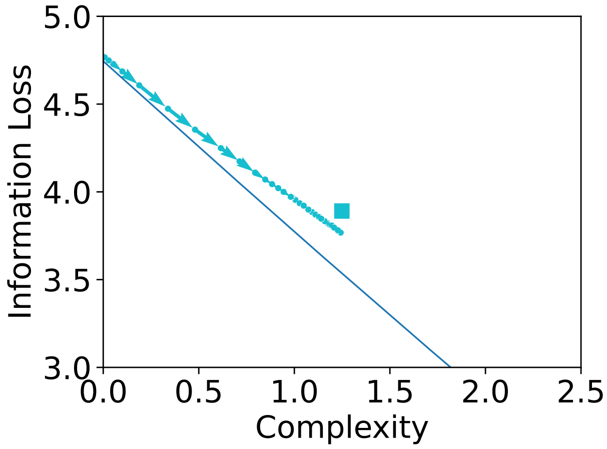

(**b**) Communicative Space

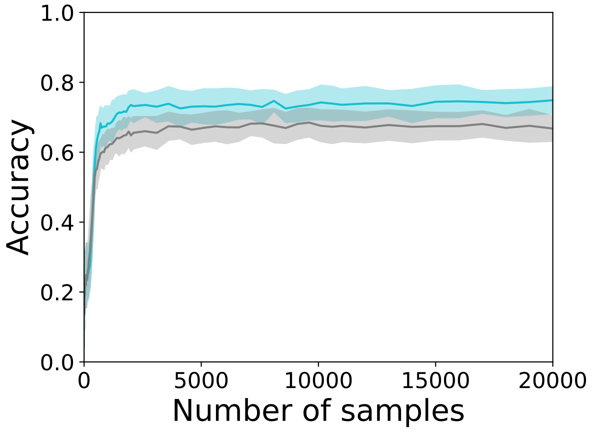

(**c**) Accuracy

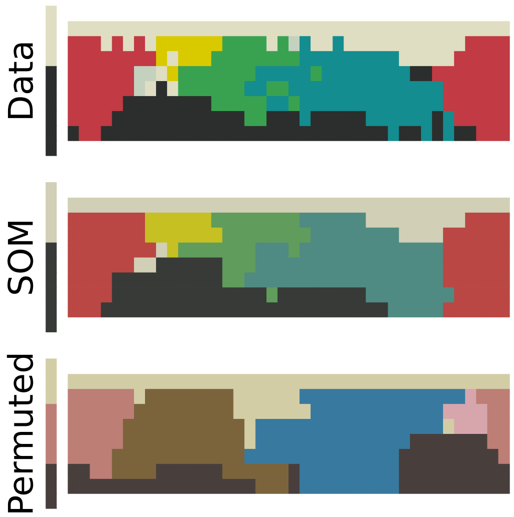

(**d**) Mode Maps

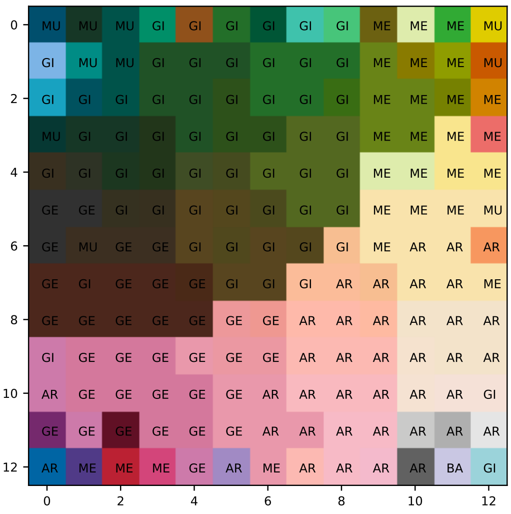

(**e**) Learnt SOM Cells

1.64. Martu Wangka; $|\mathcal{W}| = 45$

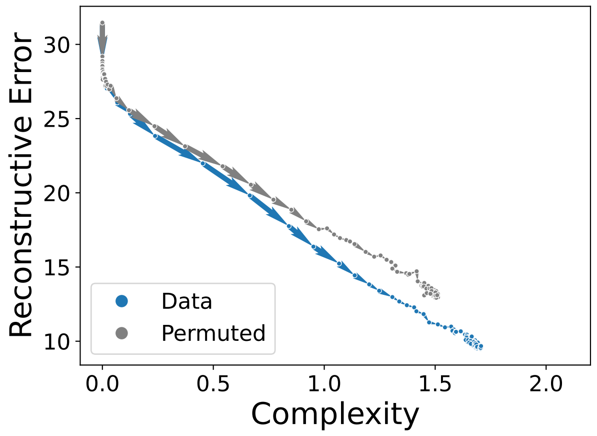

(**a**) Learning Space

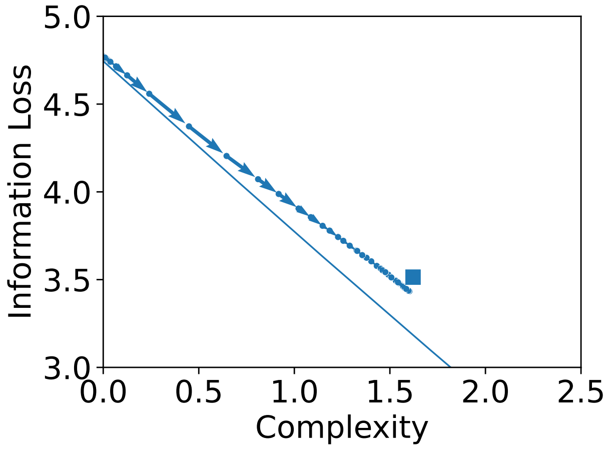

(**b**) Communicative Space

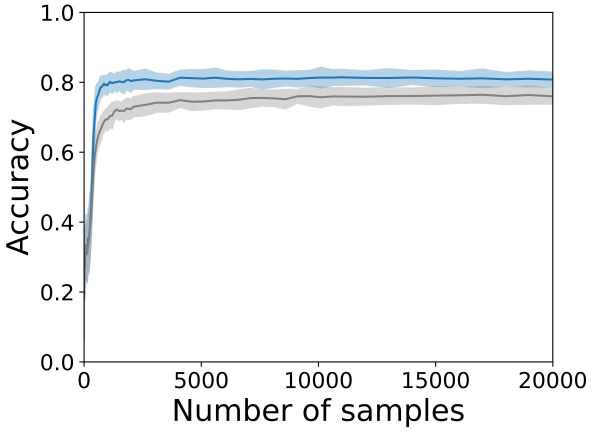

(**c**) Accuracy

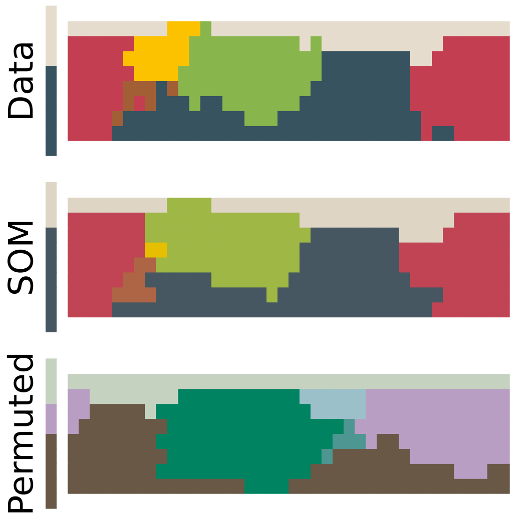

(**d**) Mode Maps

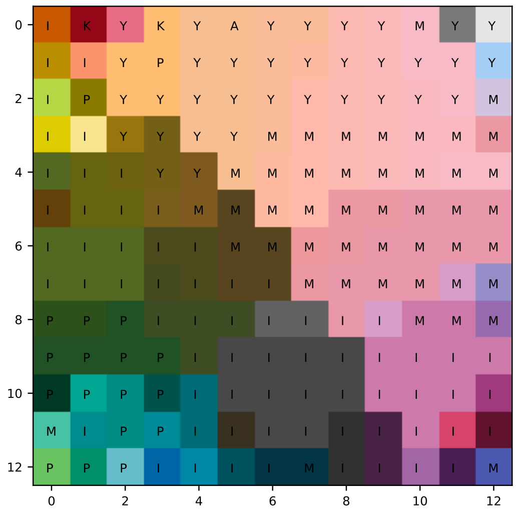

(**e**) Learnt SOM Cells

1.65. Mawchi; $|\mathcal{W}| = 9$

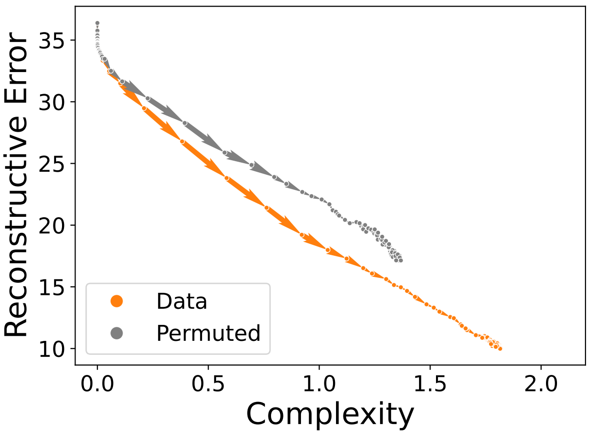

(**a**) Learning Space

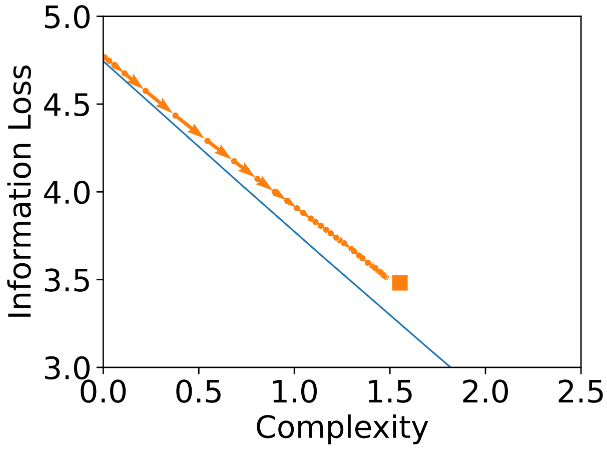

(**b**) Communicative Space

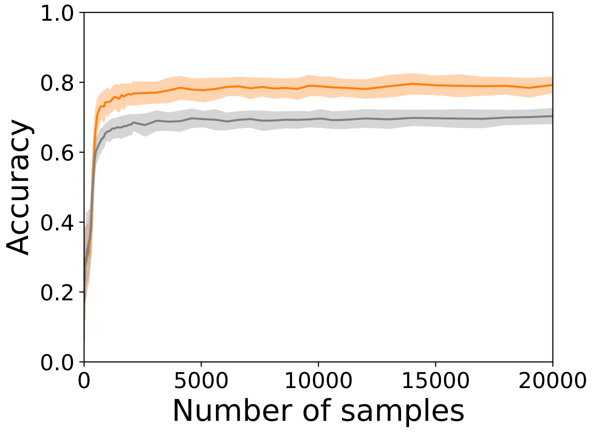

(**c**) Accuracy

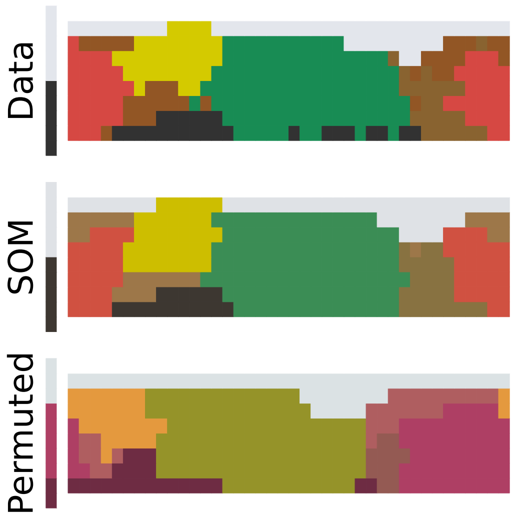

(**d**) Mode Maps

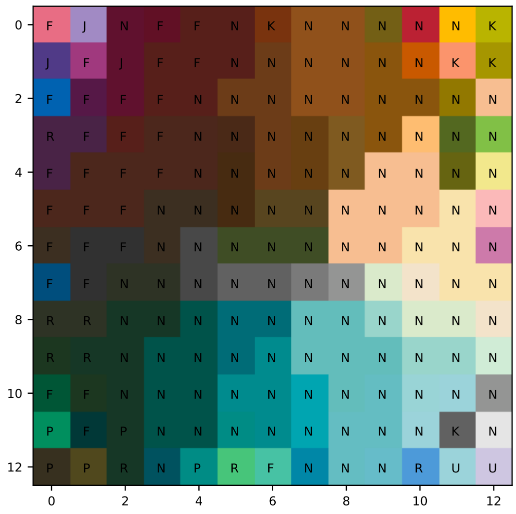

(**e**) Learnt SOM Cells

1.66. Mayoruna; $|\mathcal{W}| = 5$

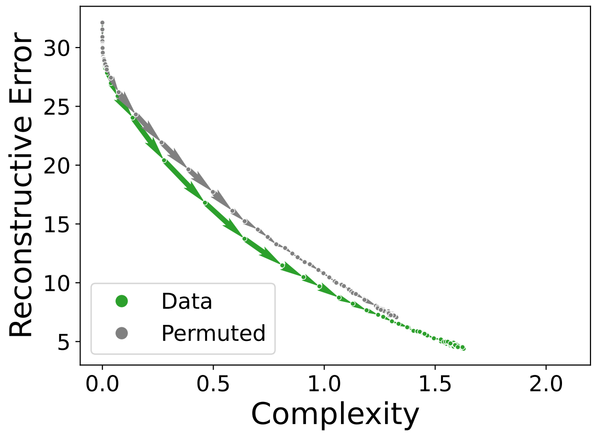

(**a**) Learning Space

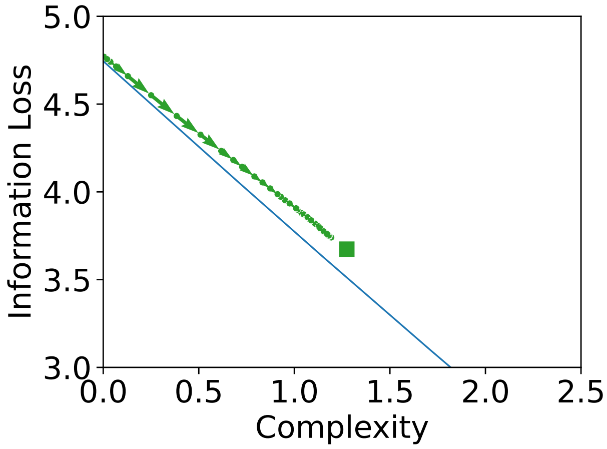

(**b**) Communicative Space

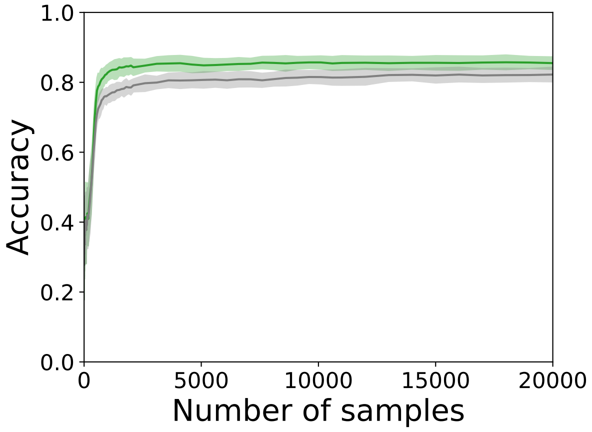

(**c**) Accuracy

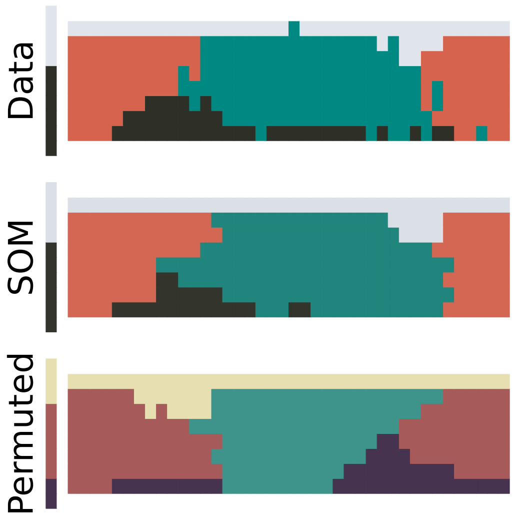

(**d**) Mode Maps

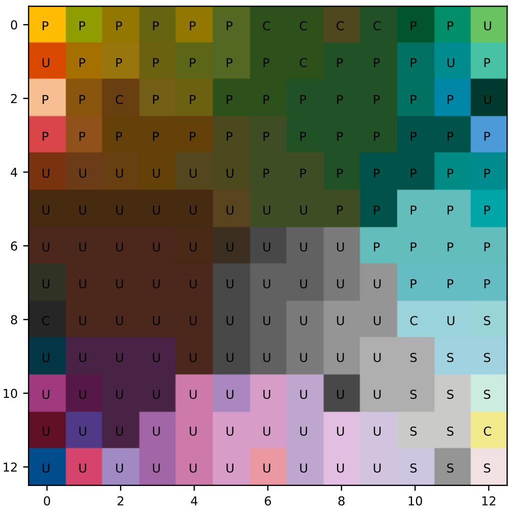

(**e**) Learnt SOM Cells

1.67. *Mazahua*; $|\mathcal{W}| = 52$

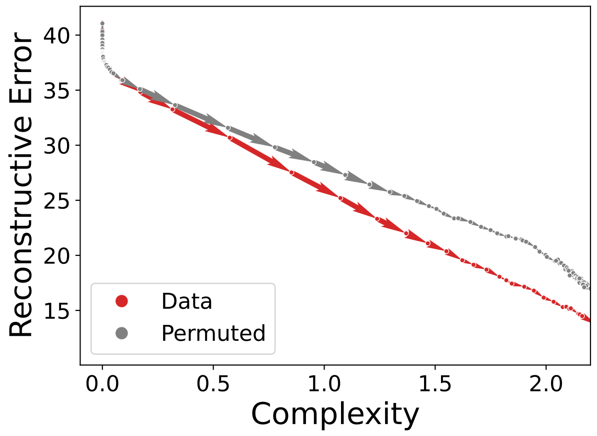

(**a**) Learning Space

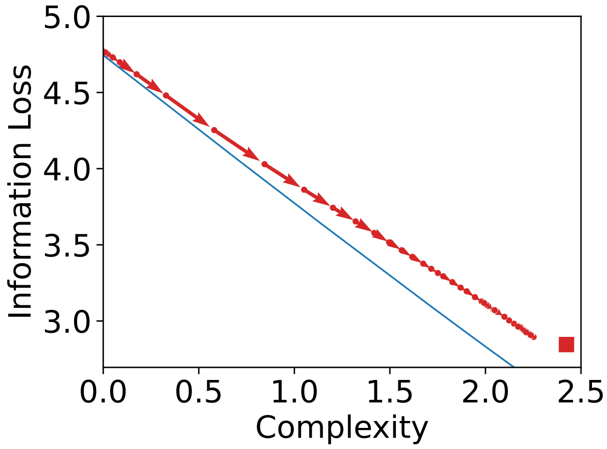

(**b**) Communicative Space

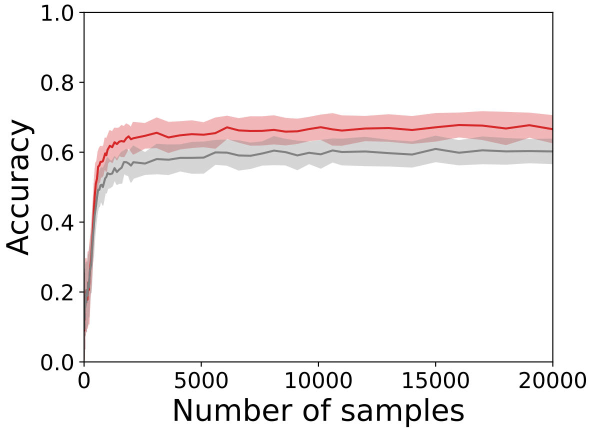

(**c**) Accuracy

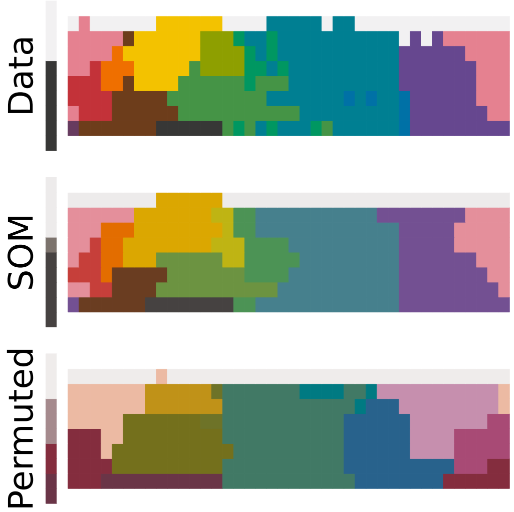

(**d**) Mode Maps

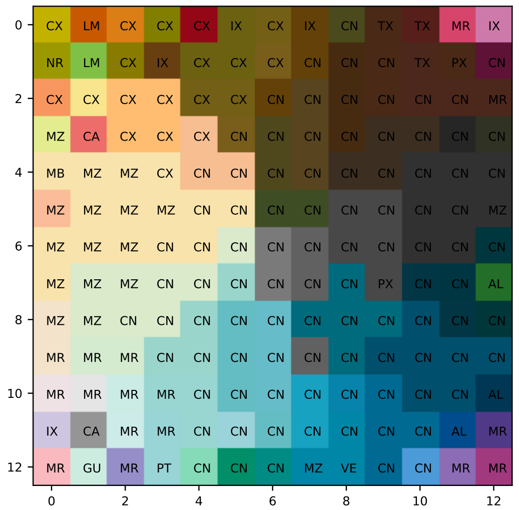

(**e**) Learnt SOM Cells

1.68. Mazateco; $|\mathcal{W}| = 20$

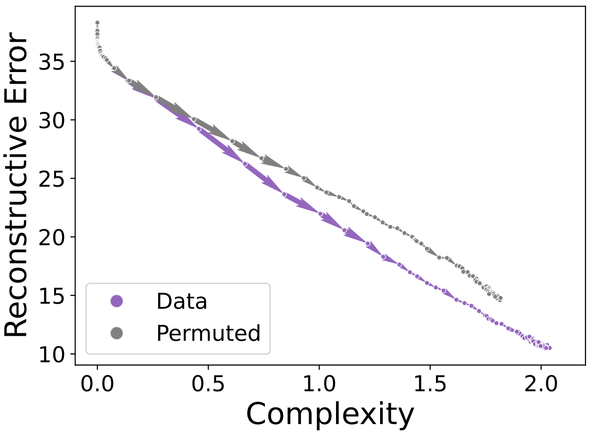

(**a**) Learning Space

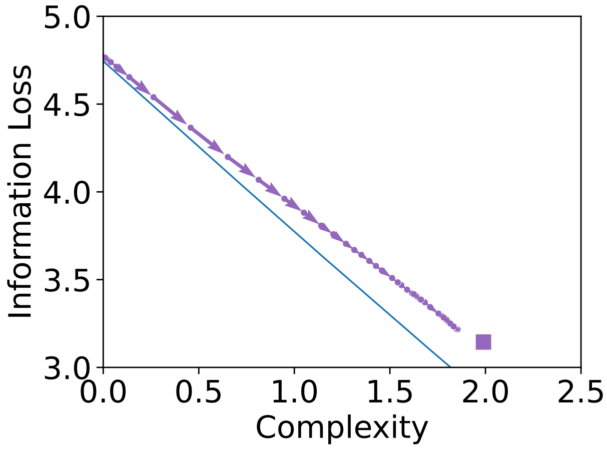

(**b**) Communicative Space

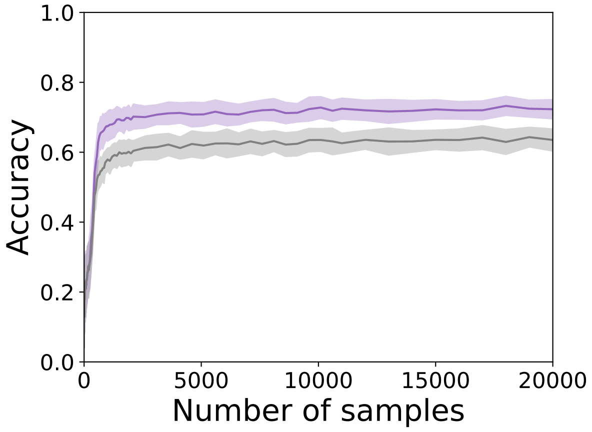

(**c**) Accuracy

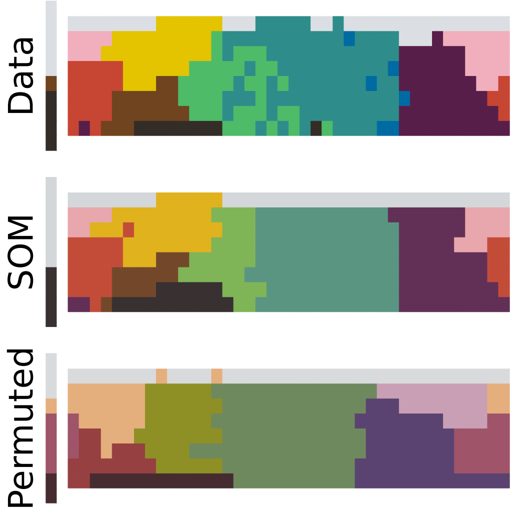

(**d**) Mode Maps

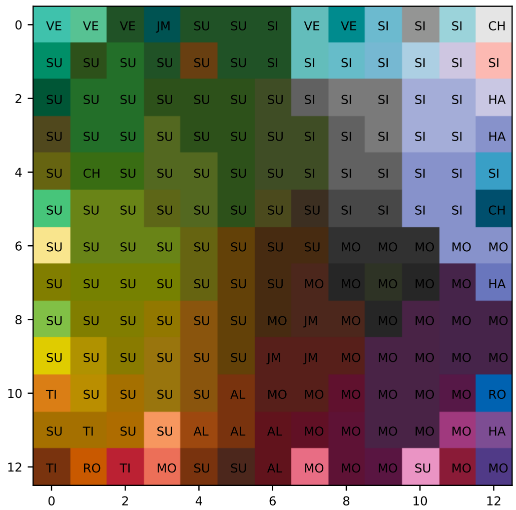

(**e**) Learnt SOM Cells

1.69. Menye; $|\mathcal{W}| = 17$

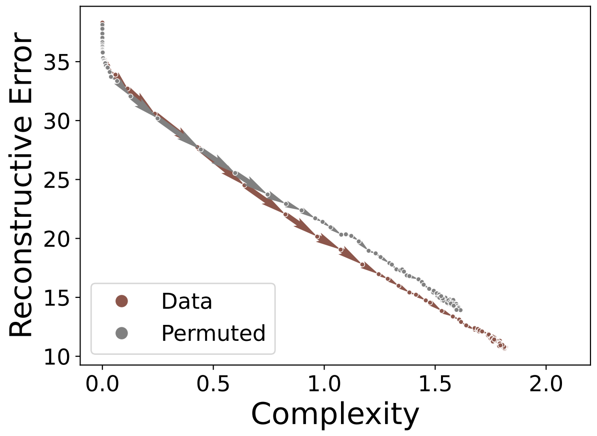

(**a**) Learning Space

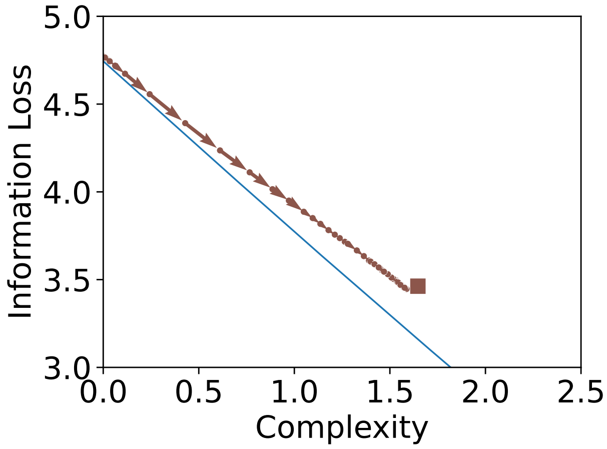

(**b**) Communicative Space

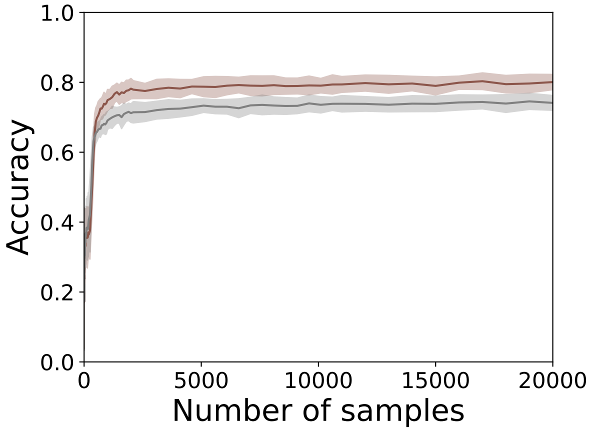

(**c**) Accuracy

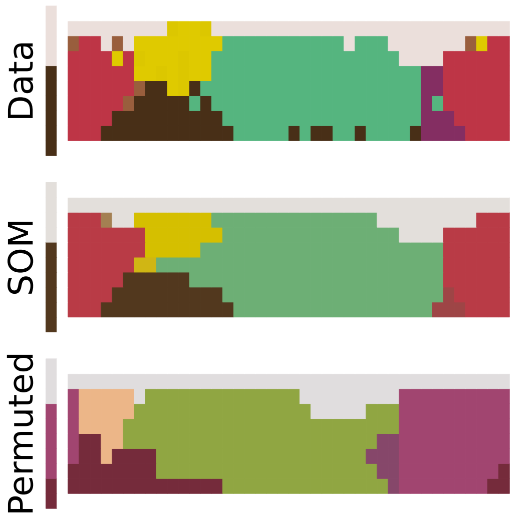

(**d**) Mode Maps

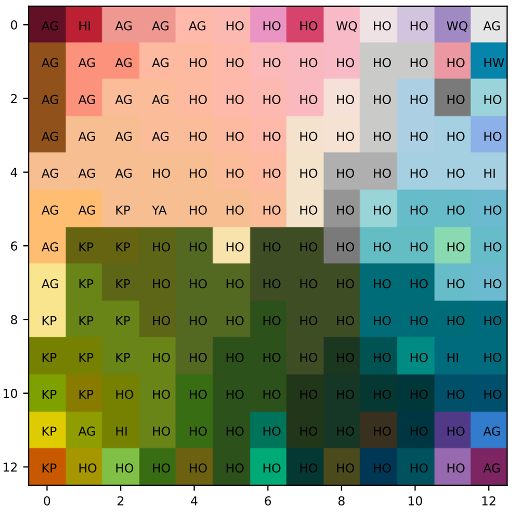

(**e**) Learnt SOM Cells

1.70. Micmac; $|\mathcal{W}| = 31$

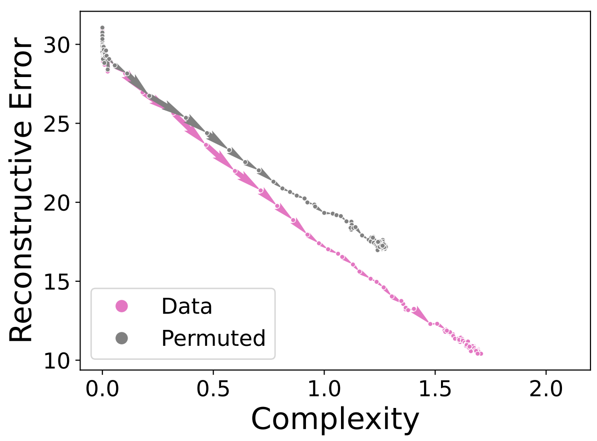

(**a**) Learning Space

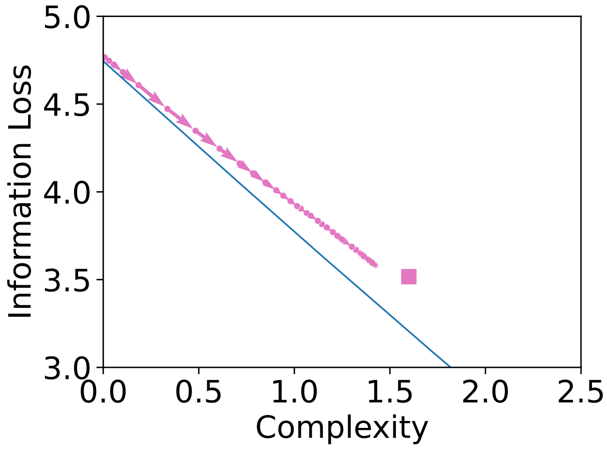

(**b**) Communicative Space

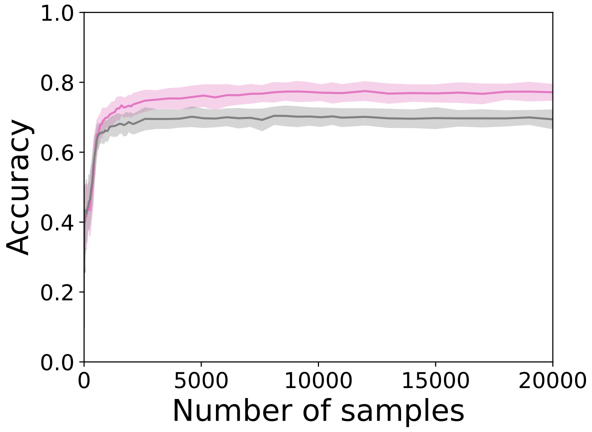

(**c**) Accuracy

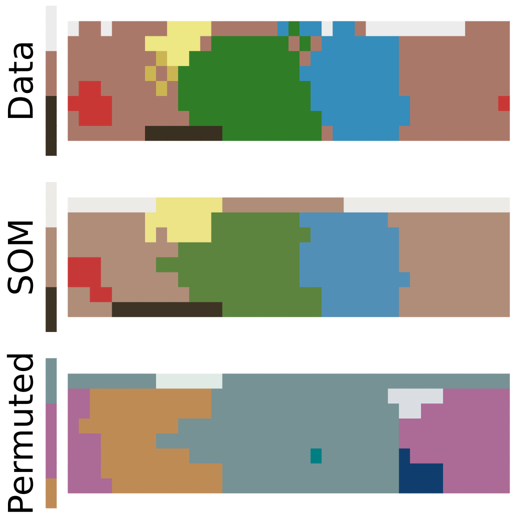

(**d**) Mode Maps

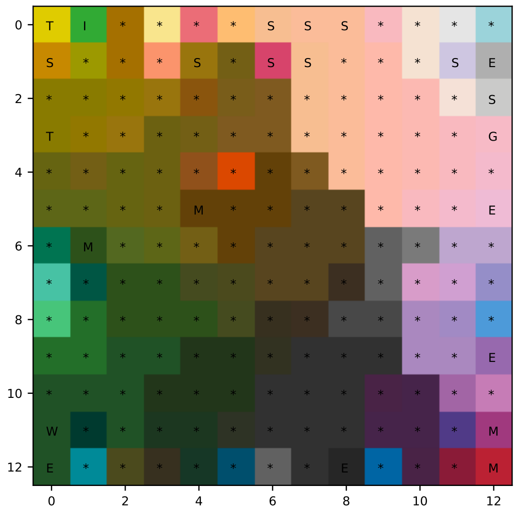

(**e**) Learnt SOM Cells

1.71. Mikasuki; $|\mathcal{W}| = 14$

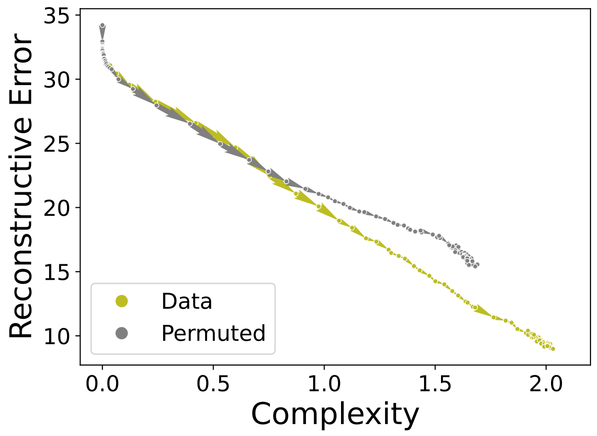

(**a**) Learning Space

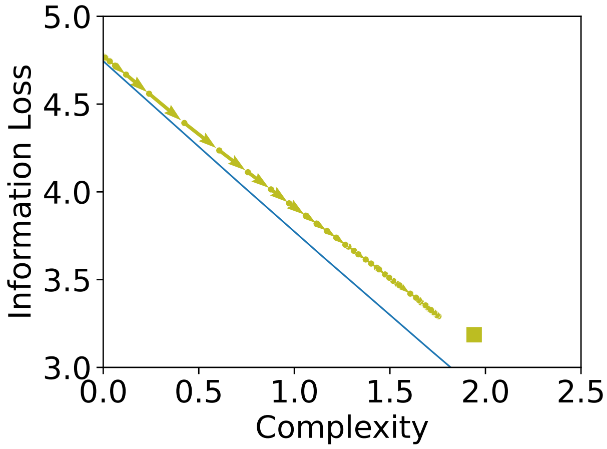

(**b**) Communicative Space

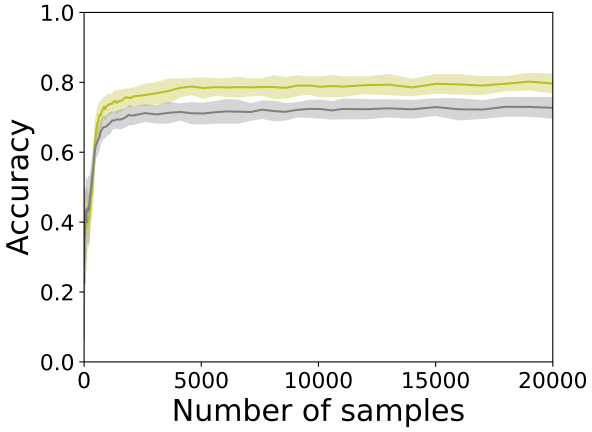

(**c**) Accuracy

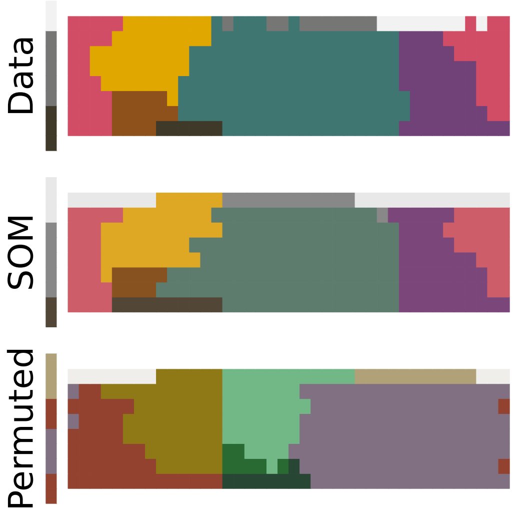

(**d**) Mode Maps

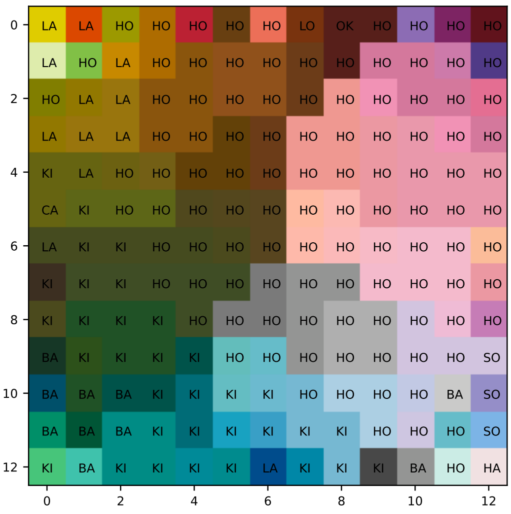

(**e**) Learnt SOM Cells

1.72. Mixteco; $|\mathcal{W}| = 30$

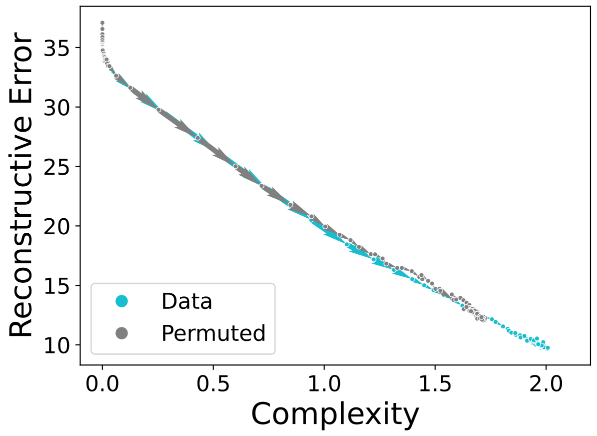

(**a**) Learning Space

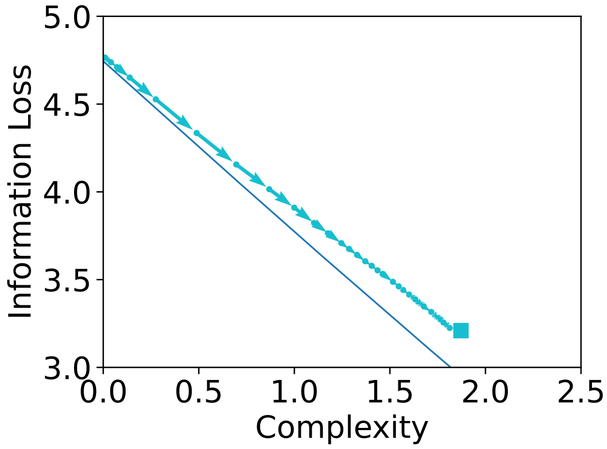

(**b**) Communicative Space

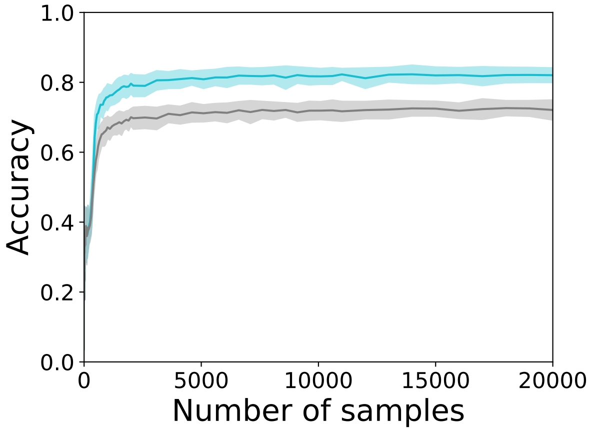

(**c**) Accuracy

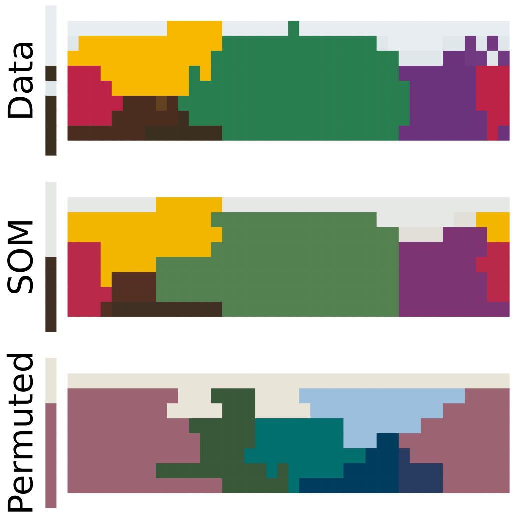

(**d**) Mode Maps

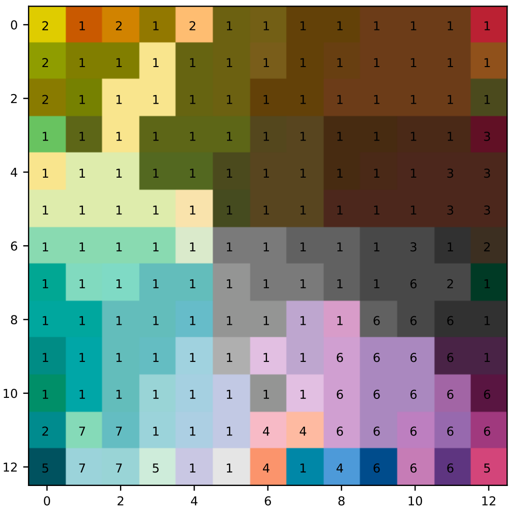

(**e**) Learnt SOM Cells

1.73. Mundu; $|\mathcal{W}| = 12$

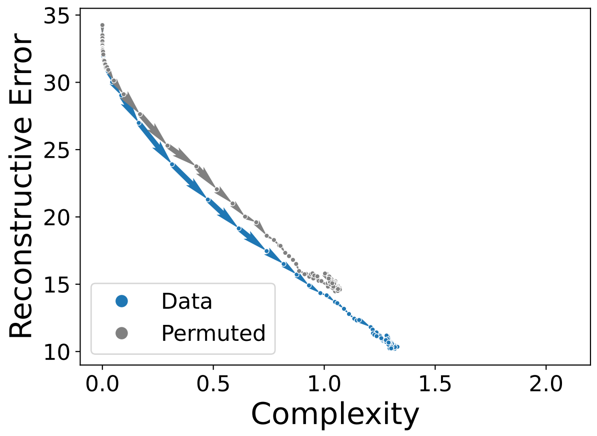

(**a**) Learning Space

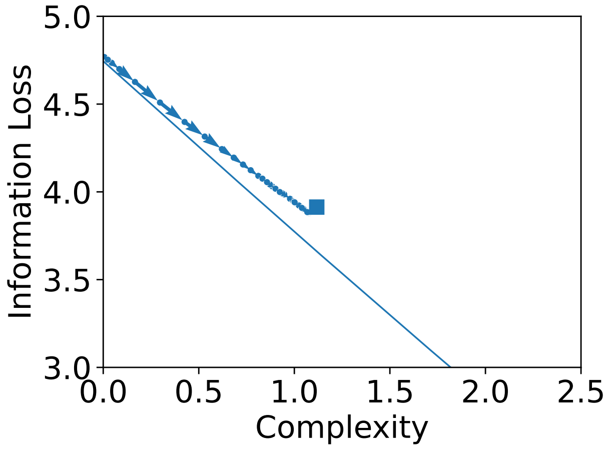

(**b**) Communicative Space

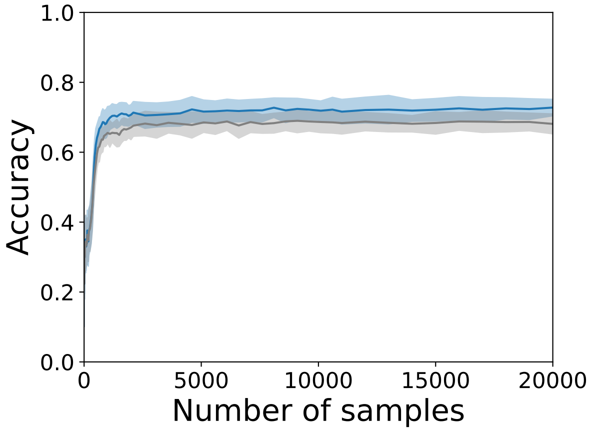

(**c**) Accuracy

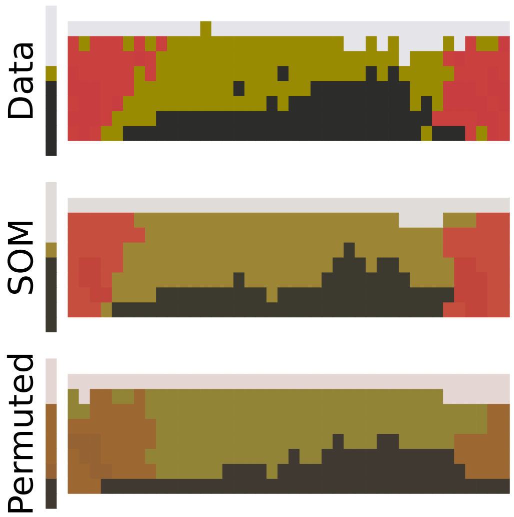

(**d**) Mode Maps

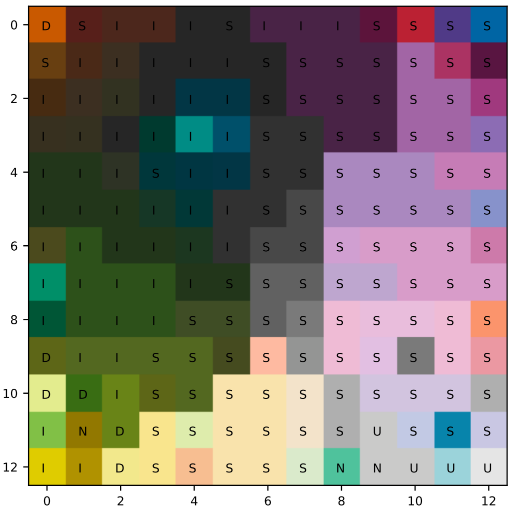

(**e**) Learnt SOM Cells

1.74. Múra Pirahá; $|\mathcal{W}| = 5$

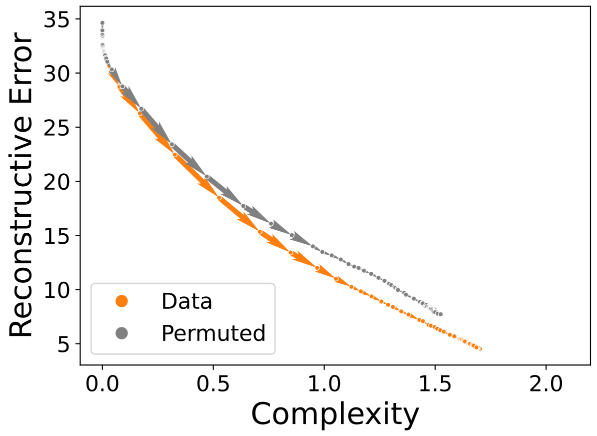

(**a**) Learning Space

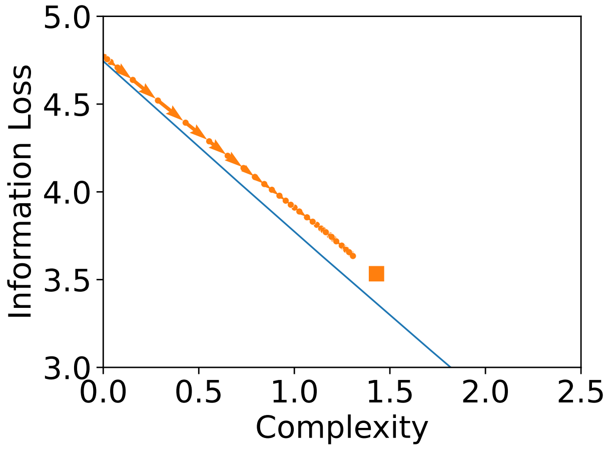

(**b**) Communicative Space

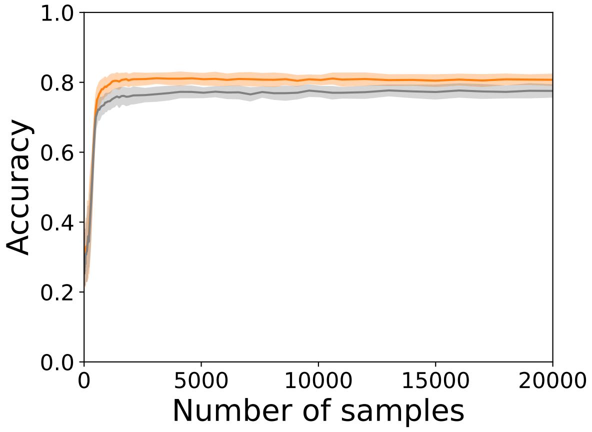

(**c**) Accuracy

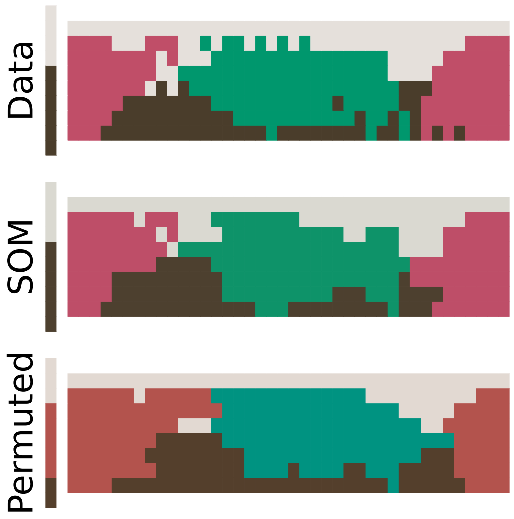

(**d**) Mode Maps

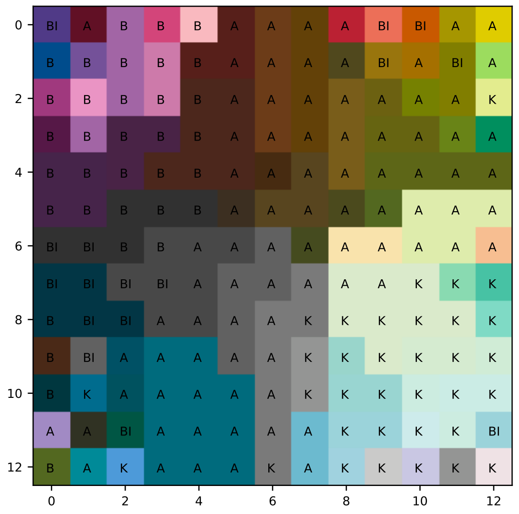

(**e**) Learnt SOM Cells

1.75. Murle; $|\mathcal{W}| = 15$

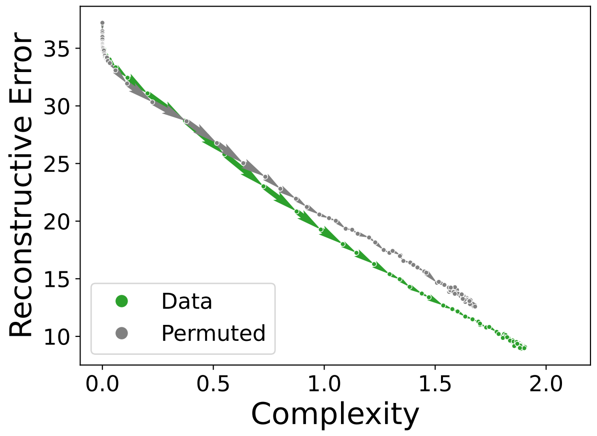

(**a**) Learning Space

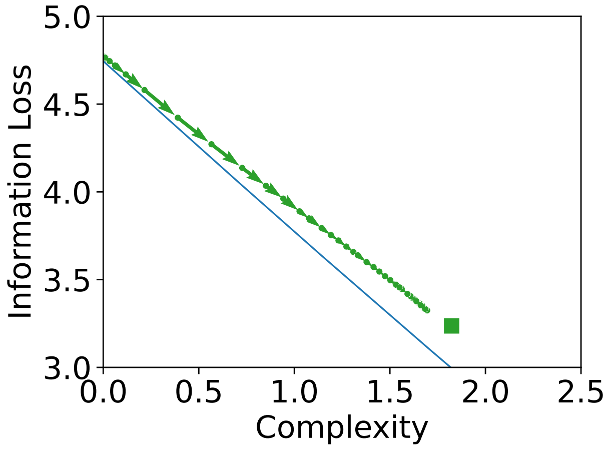

(**b**) Communicative Space

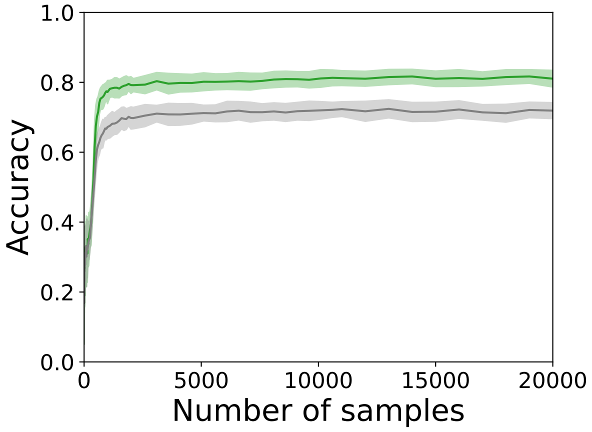

(**c**) Accuracy

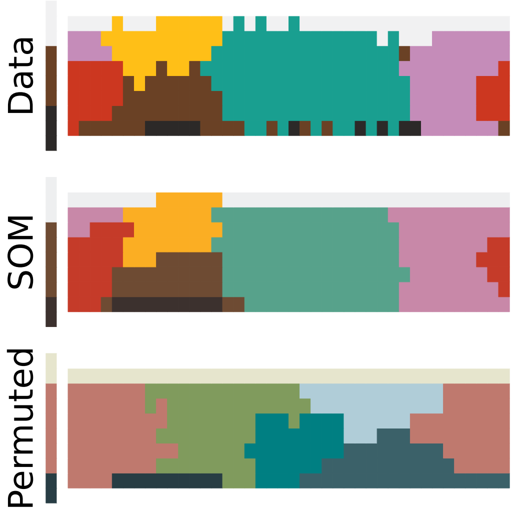

(**d**) Mode Maps

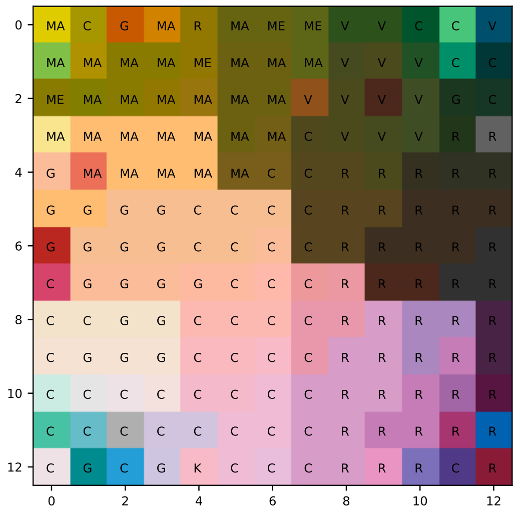

(**e**) Learnt SOM Cells

1.76. Murinbata; $|\mathcal{W}| = 22$

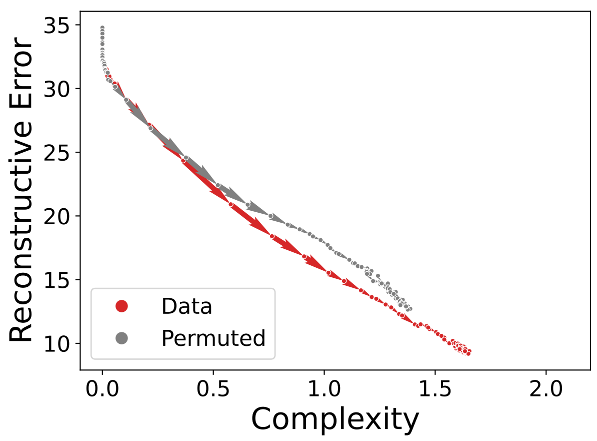

(**a**) Learning Space

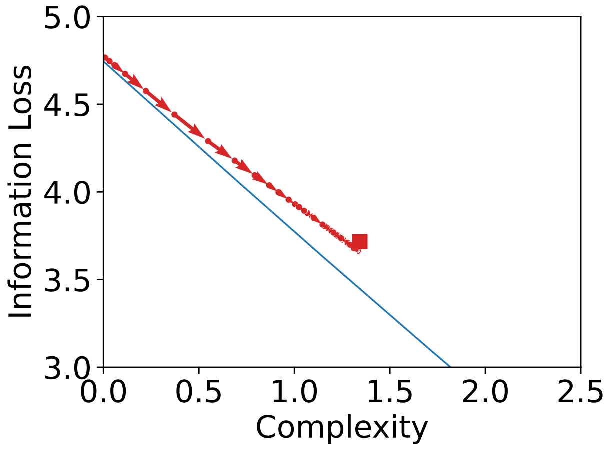

(**b**) Communicative Space

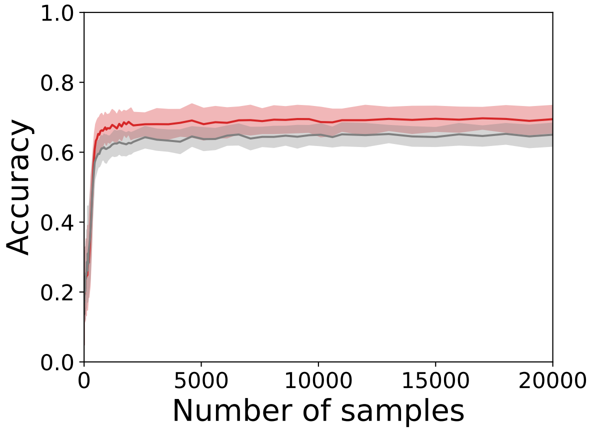

(**c**) Accuracy

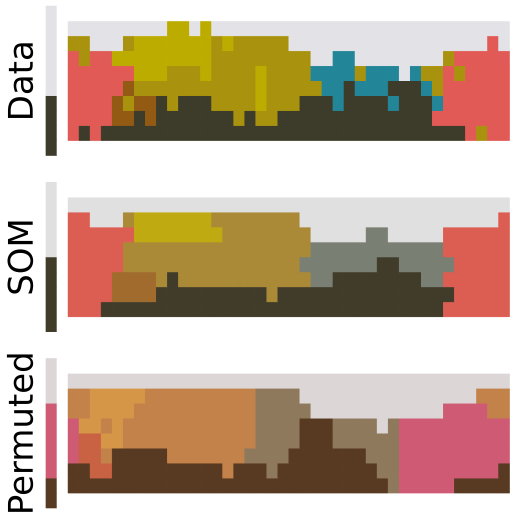

(**d**) Mode Maps

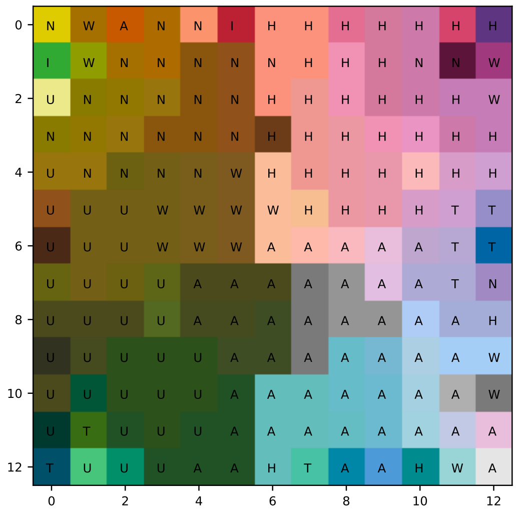

(**e**) Learnt SOM Cells

1.77. Nafaanra; $|\mathcal{W}| = 5$

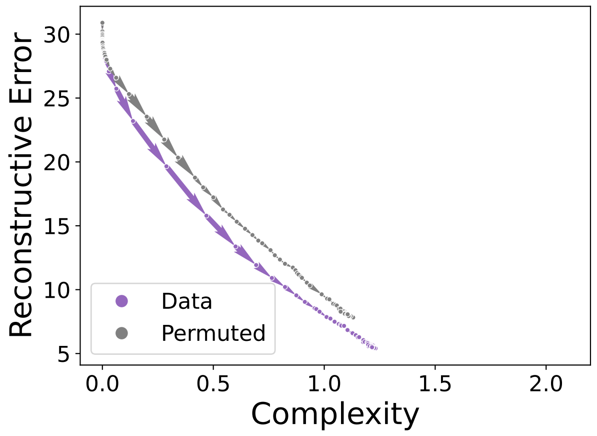

(**a**) Learning Space

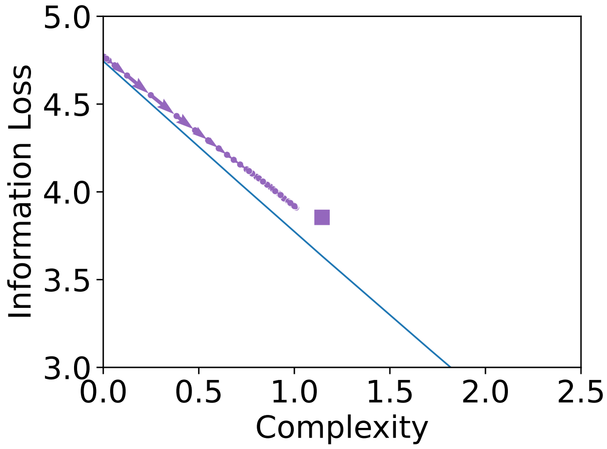

(**b**) Communicative Space

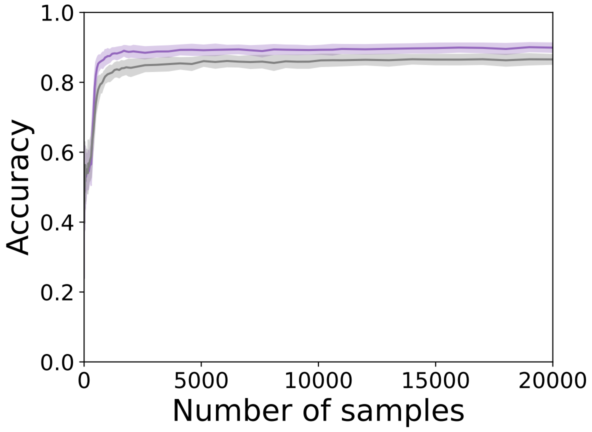

(**c**) Accuracy

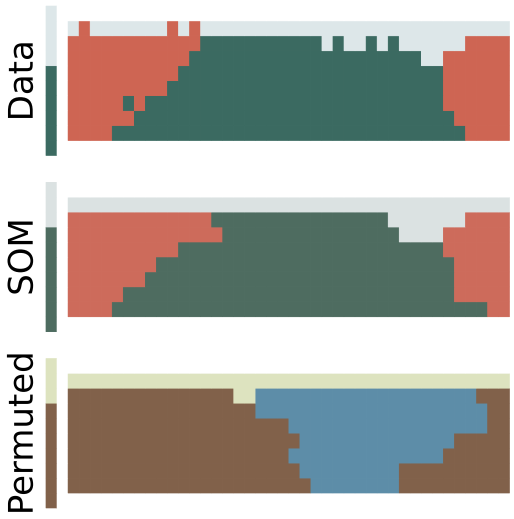

(**d**) Mode Maps

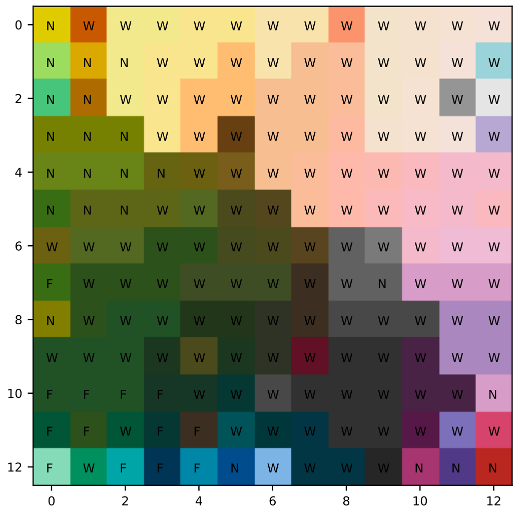

(**e**) Learnt SOM Cells

1.78. Nahuatl; $|\mathcal{W}| = 16$

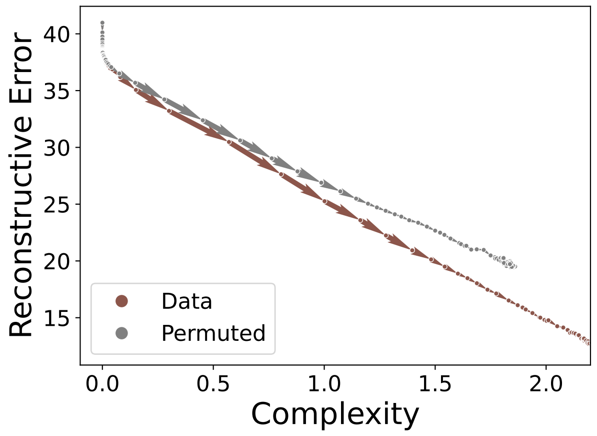

(**a**) Learning Space

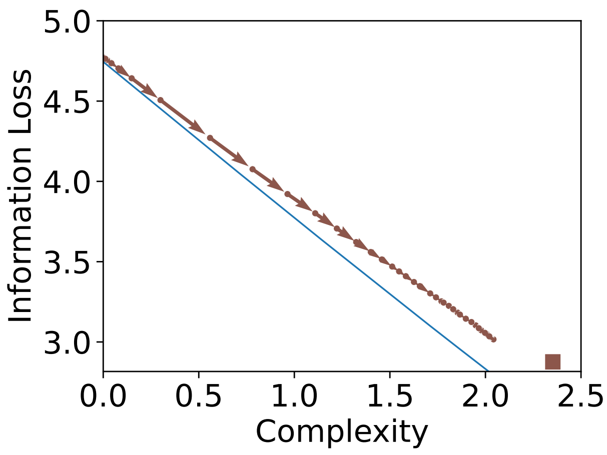

(**b**) Communicative Space

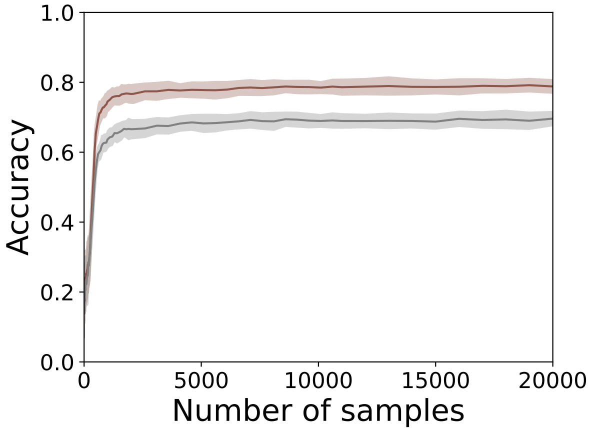

(**c**) Accuracy

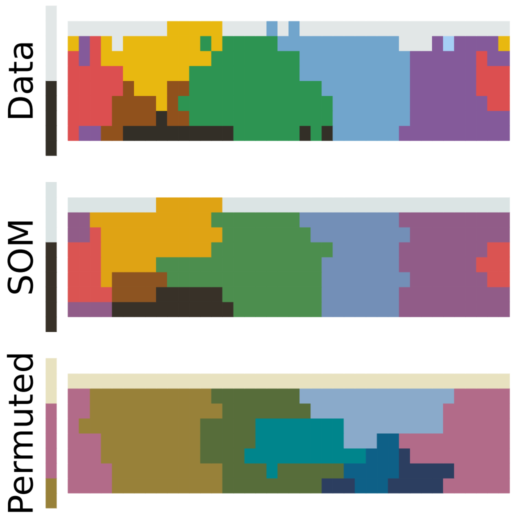

(**d**) Mode Maps

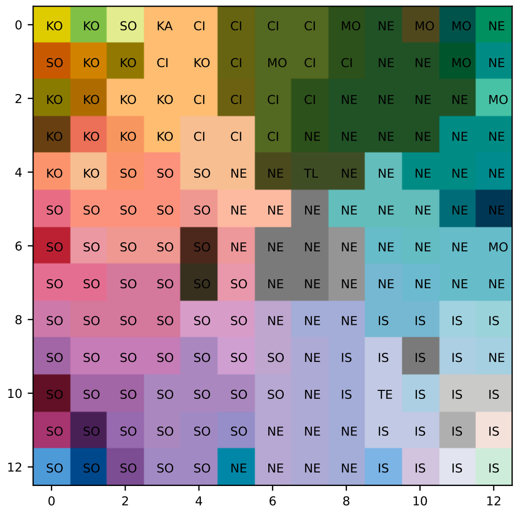

(**e**) Learnt SOM Cells

1.79. Ocaina; $|\mathcal{W}| = 12$

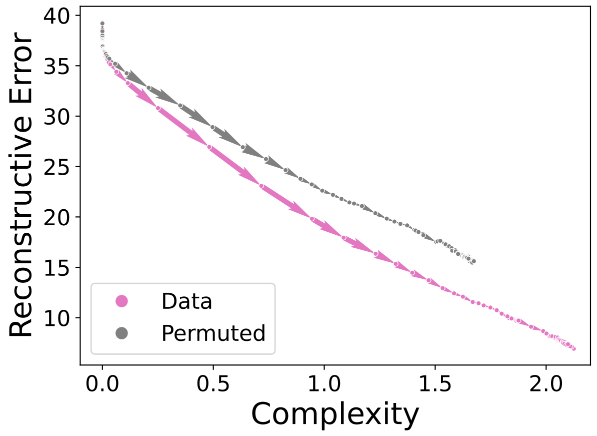

(**a**) Learning Space

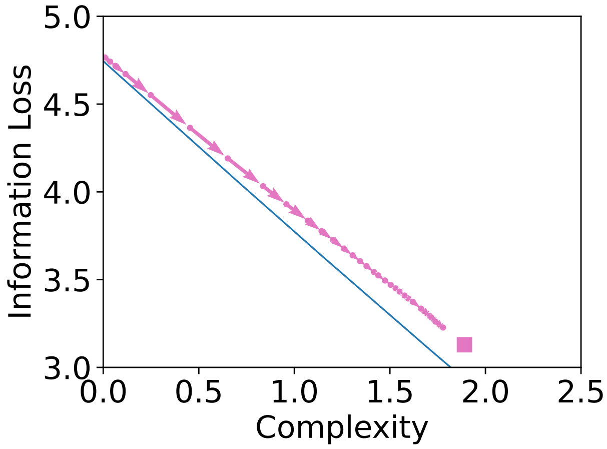

(**b**) Communicative Space

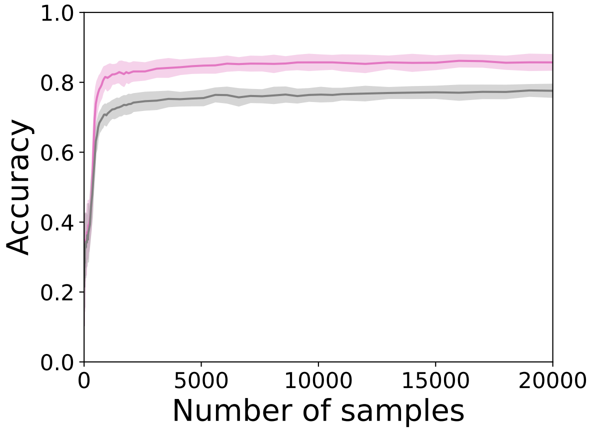

(**c**) Accuracy

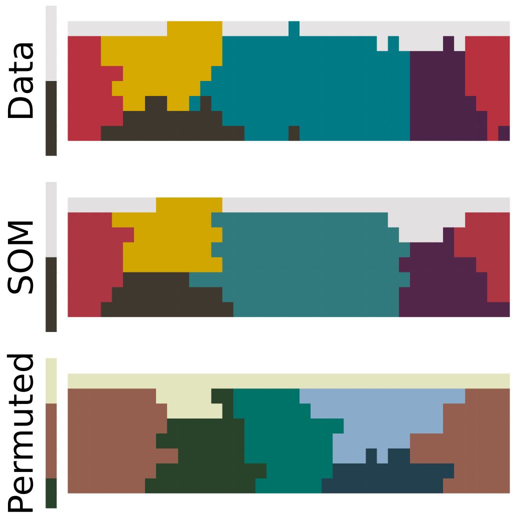

(**d**) Mode Maps

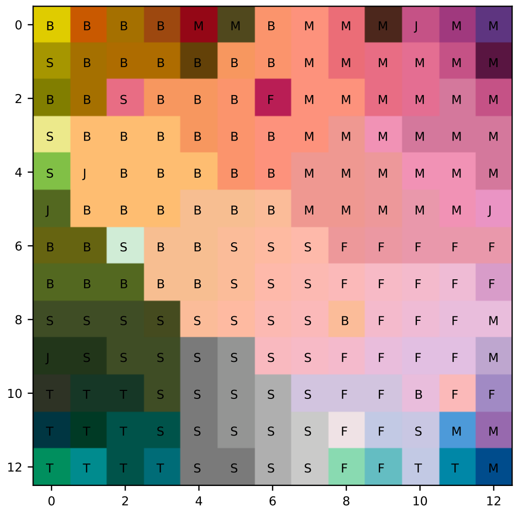

(**e**) Learnt SOM Cells

1.80. Papago; $|\mathcal{W}| = 26$

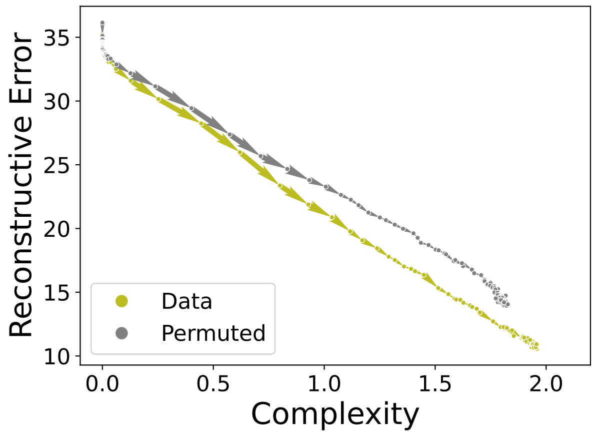

(**a**) Learning Space

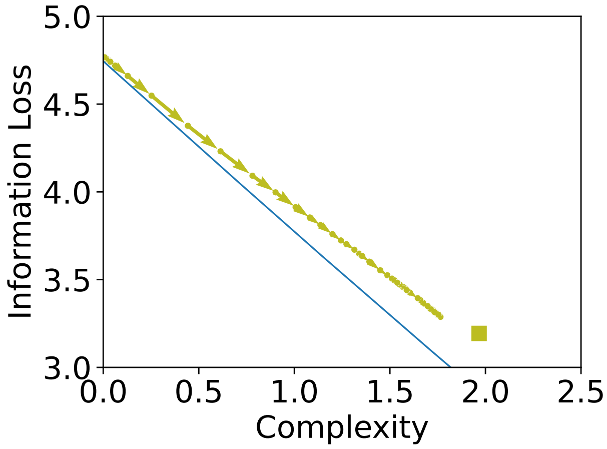

(**b**) Communicative Space

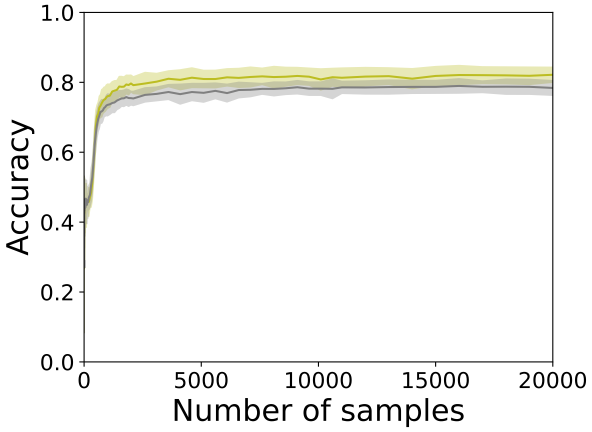

(**c**) Accuracy

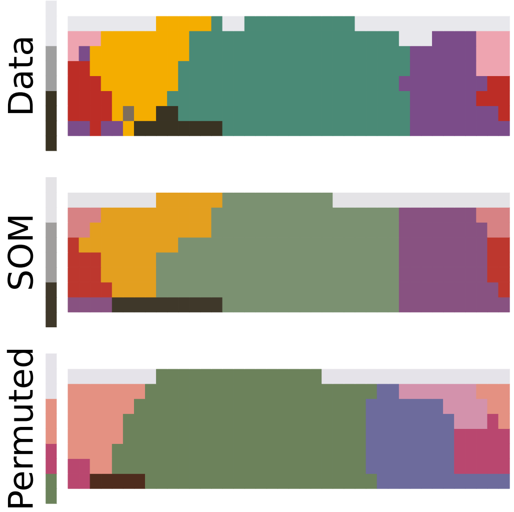

(**d**) Mode Maps

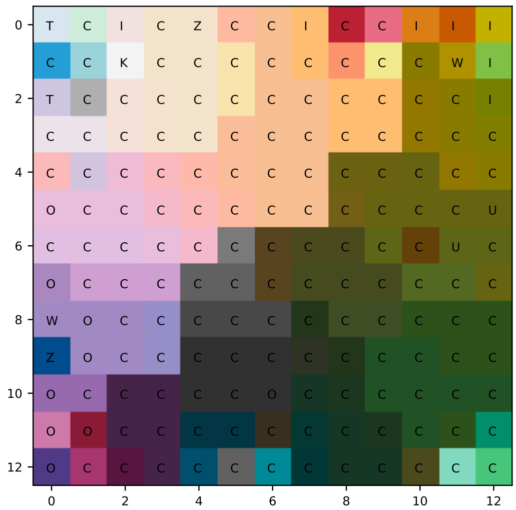

(**e**) Learnt SOM Cells

1.81. Patep; $|\mathcal{W}| = 15$

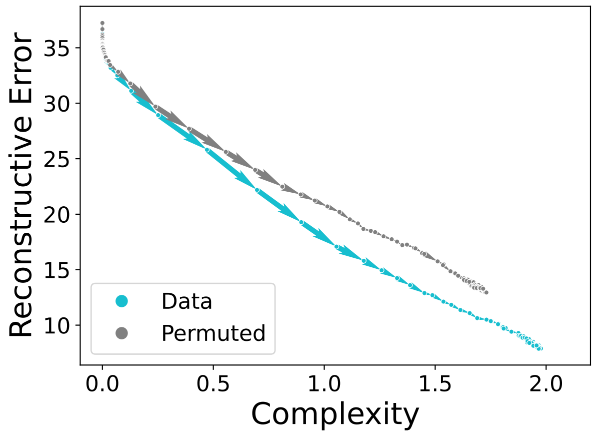

(**a**) Learning Space

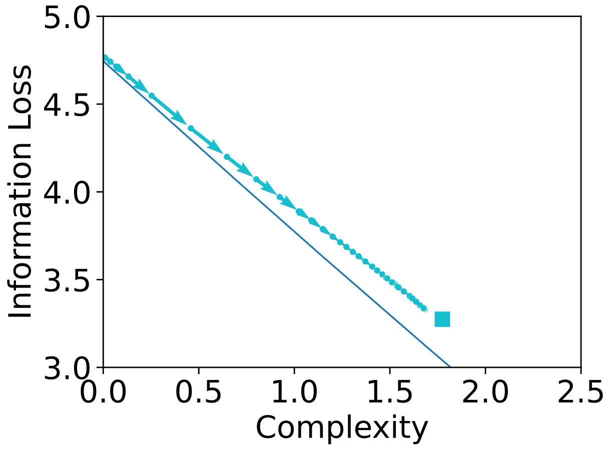

(**b**) Communicative Space

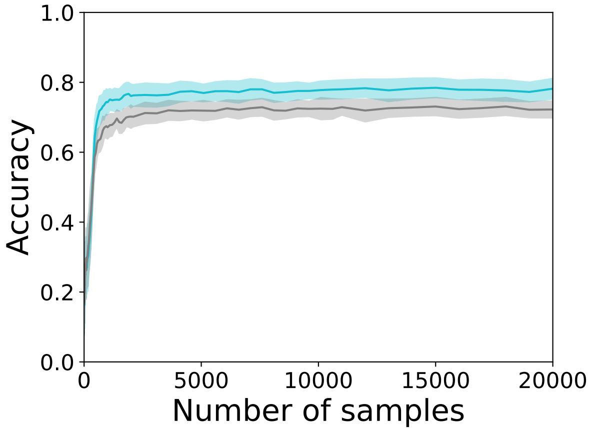

(**c**) Accuracy

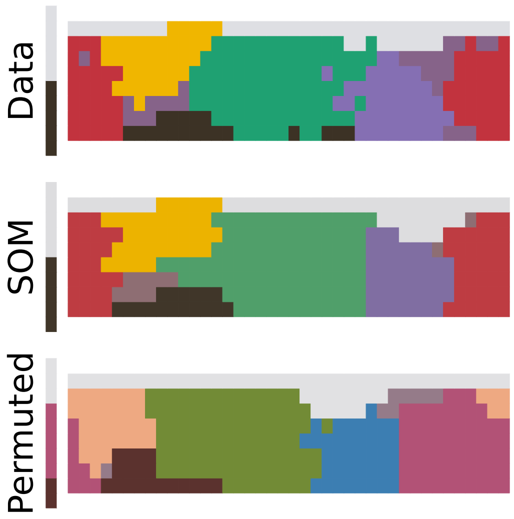

(**d**) Mode Maps

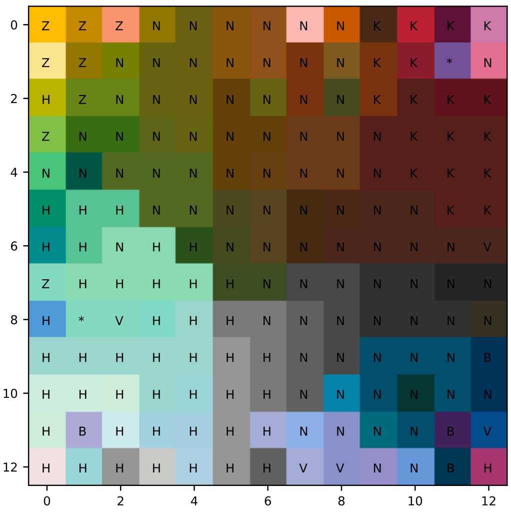

(**e**) Learnt SOM Cells

1.82. Paya; $|\mathcal{W}| = 13$

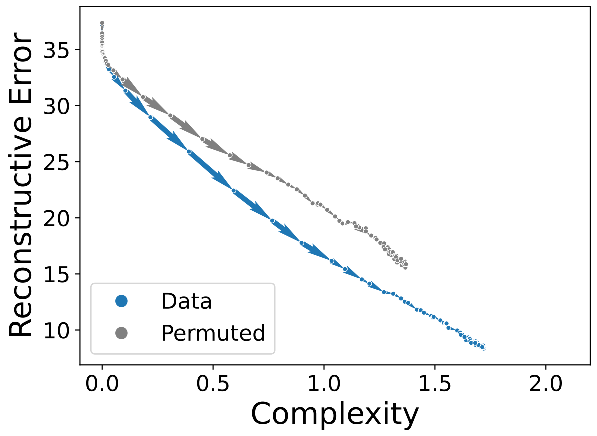

(**a**) Learning Space

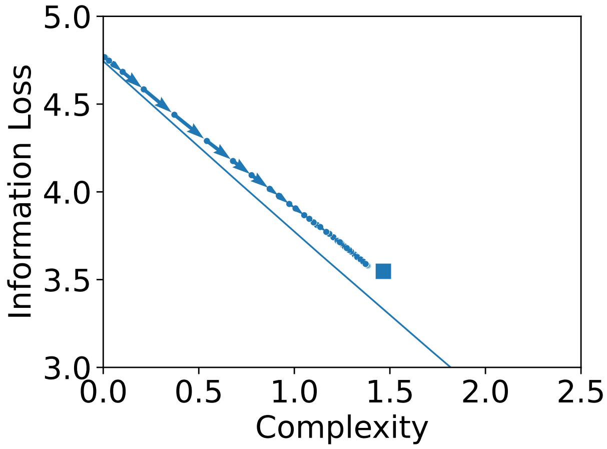

(**b**) Communicative Space

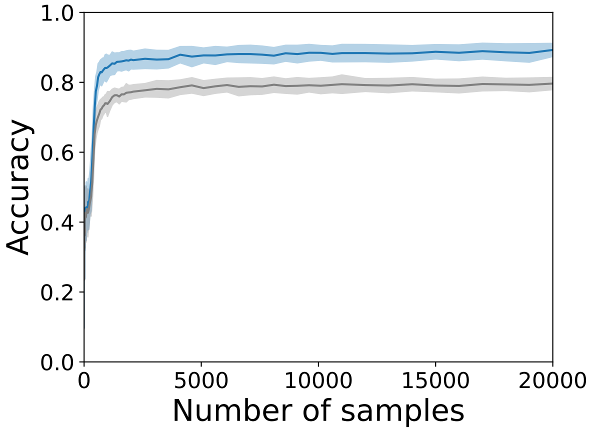

(**c**) Accuracy

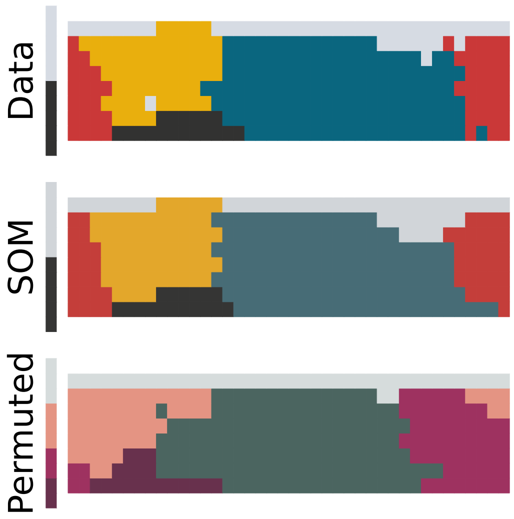

(**d**) Mode Maps

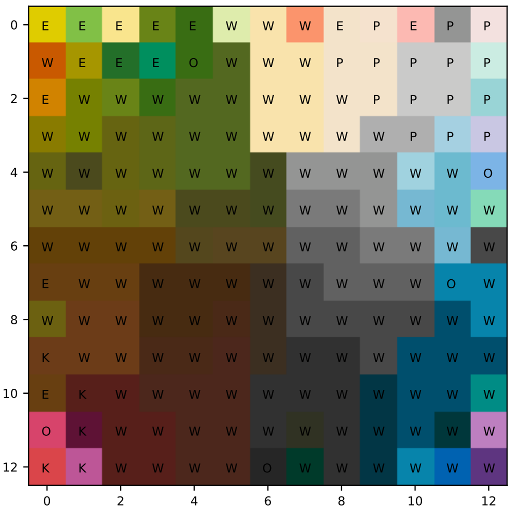

(**e**) Learnt SOM Cells

1.83. Podopa; $|\mathcal{W}| = 19$

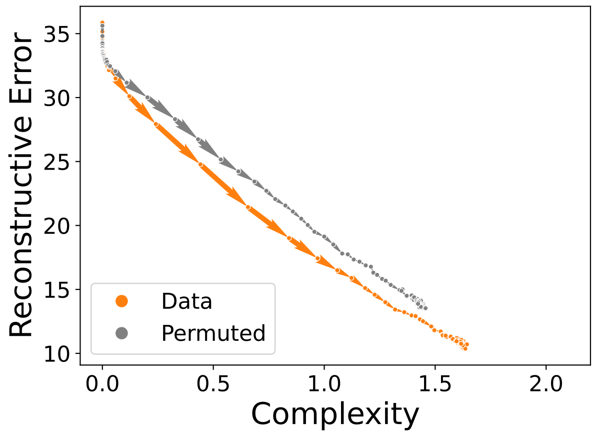

(**a**) Learning Space

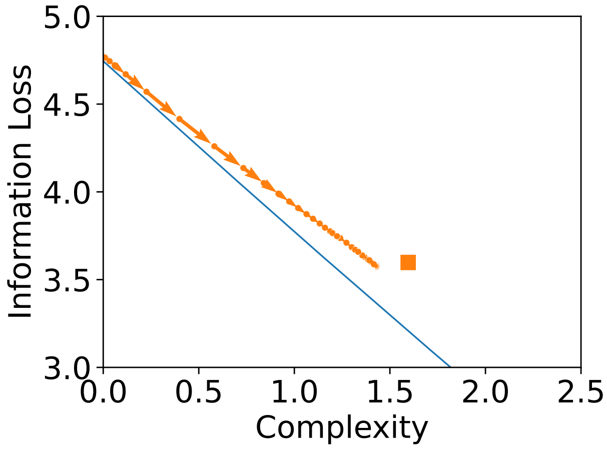

(**b**) Communicative Space

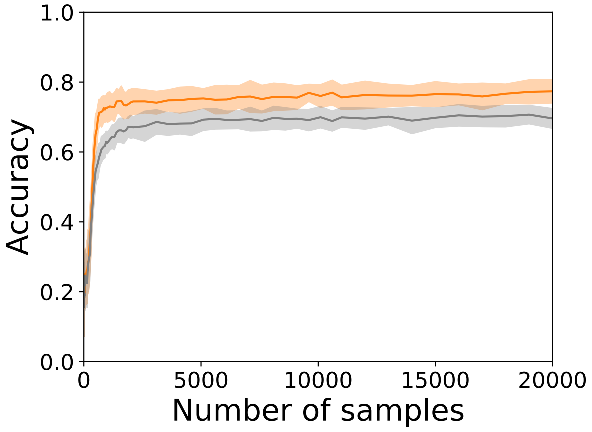

(**c**) Accuracy

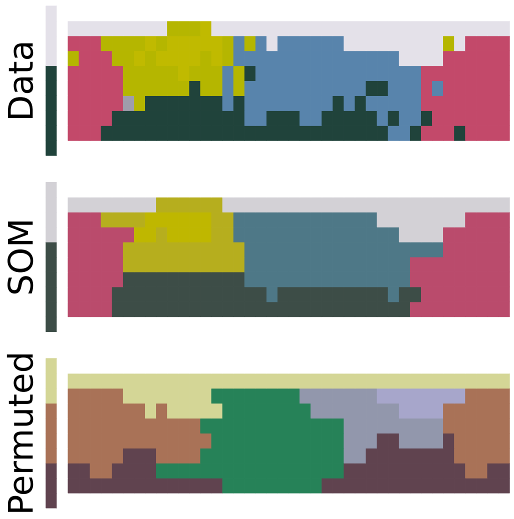

(**d**) Mode Maps

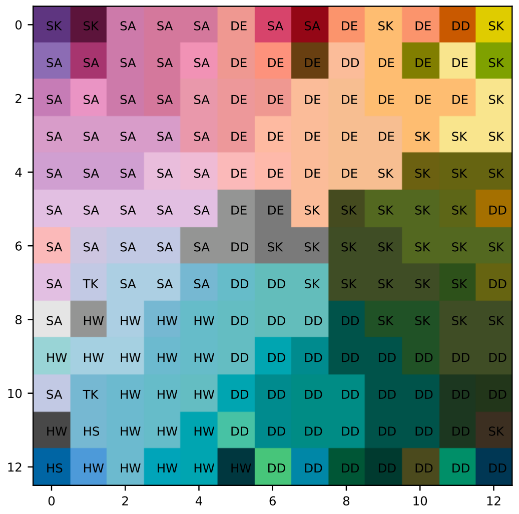

(**e**) Learnt SOM Cells

1.84. Saramaccan; $|\mathcal{W}| = 41$

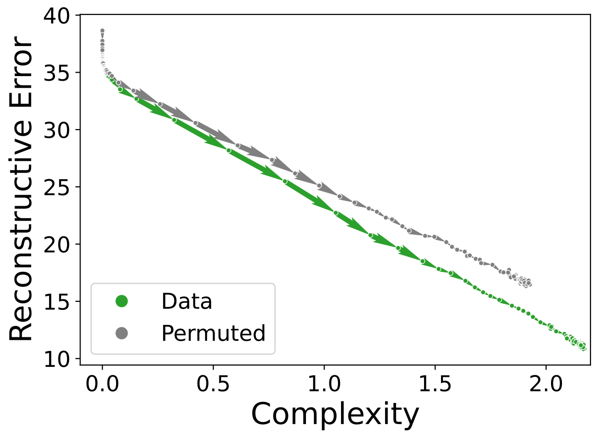

(**a**) Learning Space

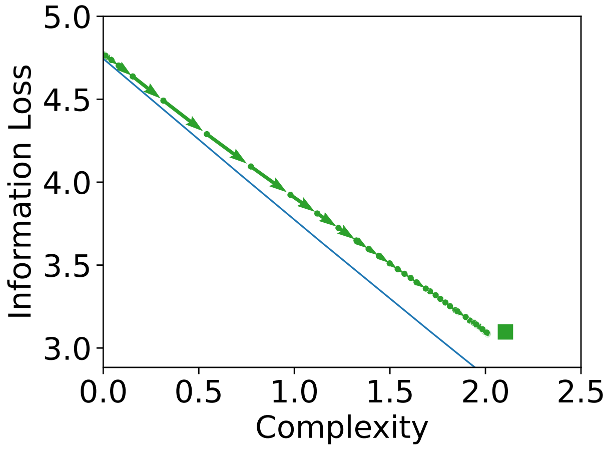

(**b**) Communicative Space

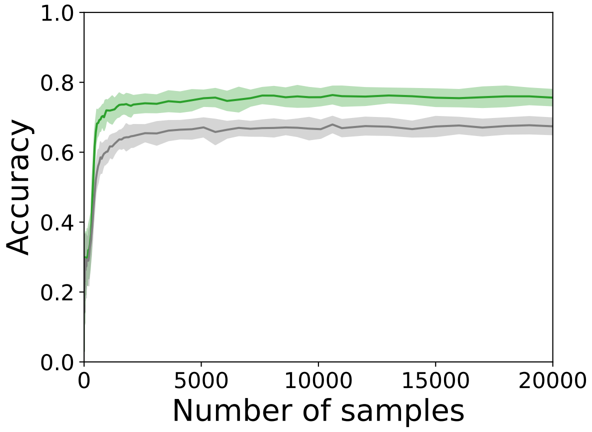

(**c**) Accuracy

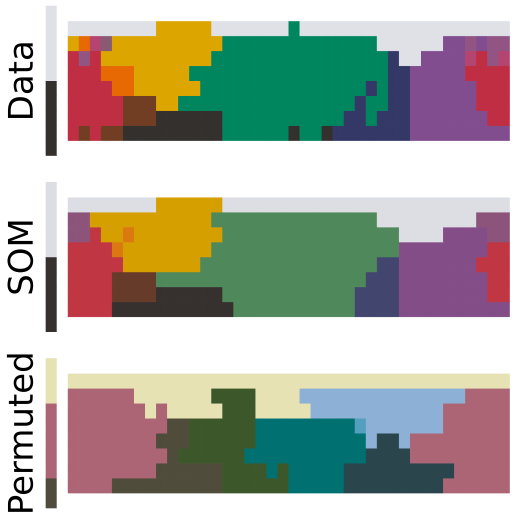

(**d**) Mode Maps

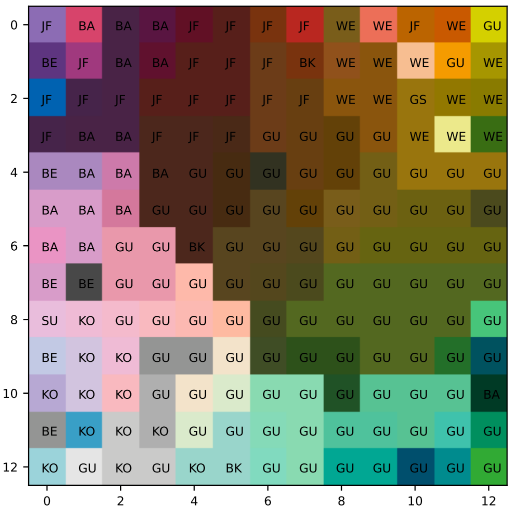

(**e**) Learnt SOM Cells

1.85. Seri; $|\mathcal{W}| = 12$

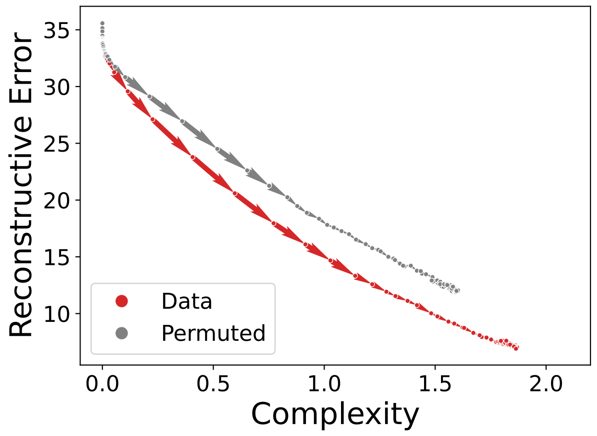

(**a**) Learning Space

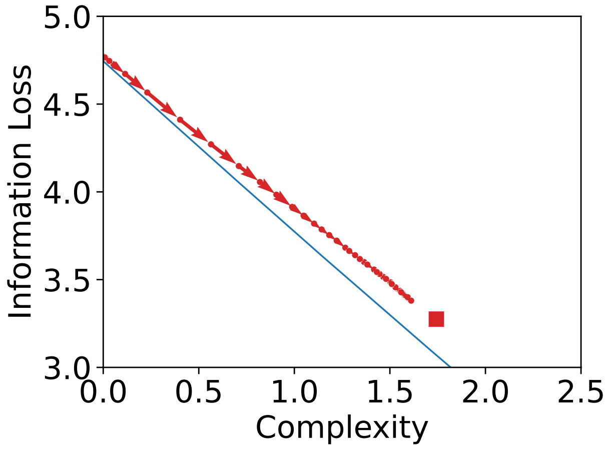

(**b**) Communicative Space

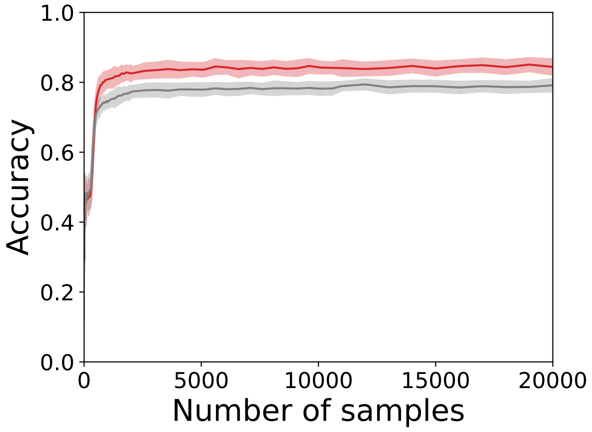

(**c**) Accuracy

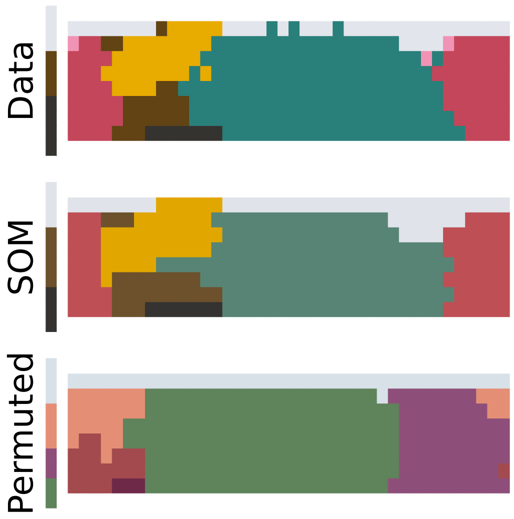

(**d**) Mode Maps

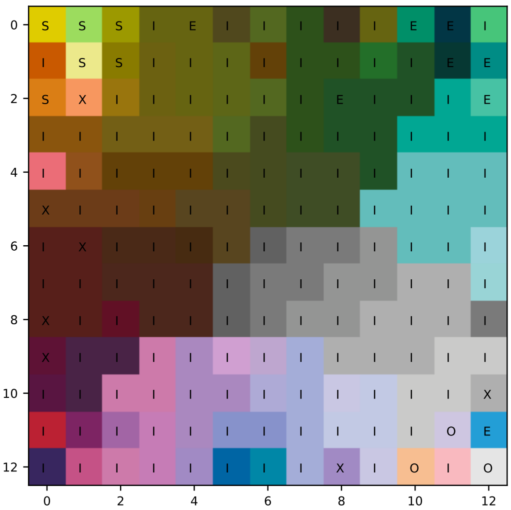

(**e**) Learnt SOM Cells

1.86. Shipibo; $|\mathcal{W}| = 21$

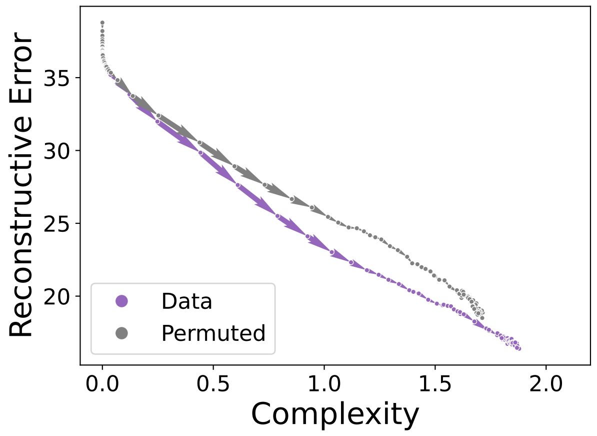

(**a**) Learning Space

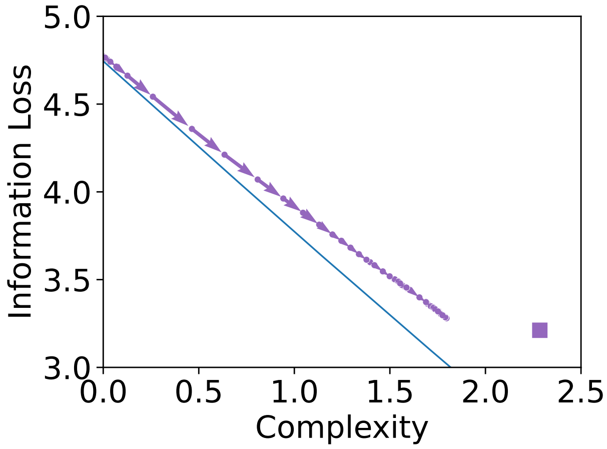

(**b**) Communicative Space

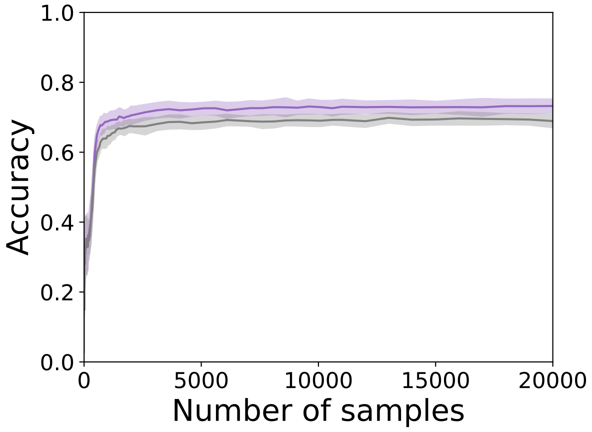

(**c**) Accuracy

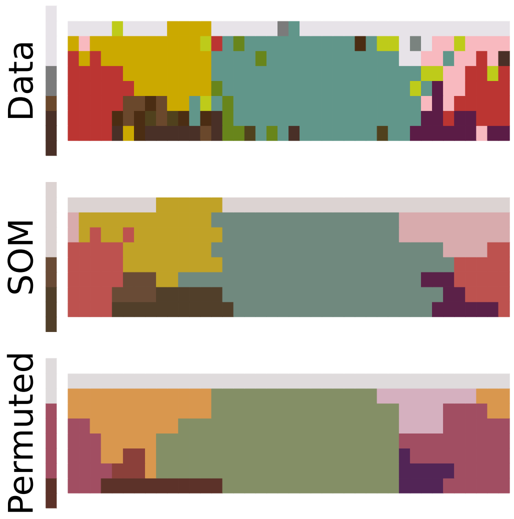

(**d**) Mode Maps

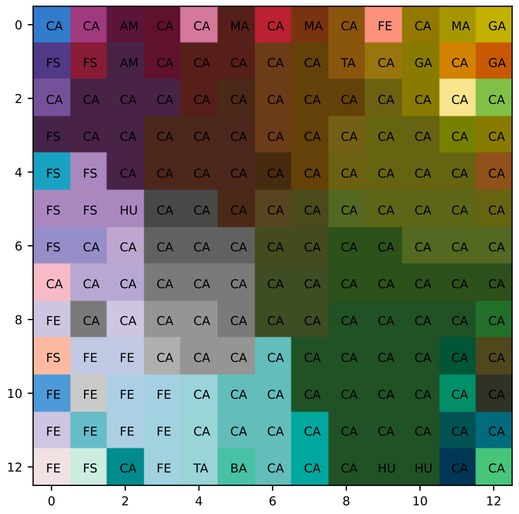

(**e**) Learnt SOM Cells

1.87. Sirionó; $|\mathcal{W}| = 14$

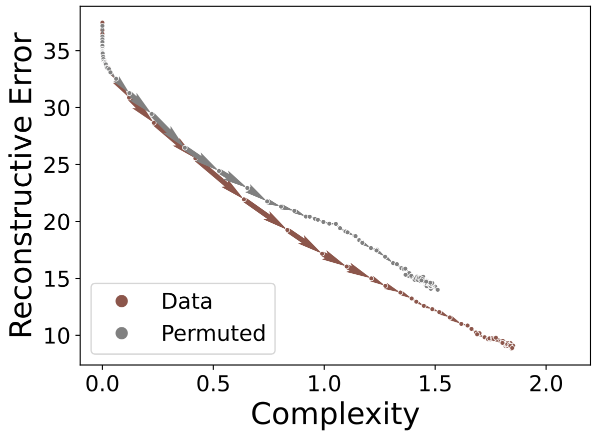

(**a**) Learning Space

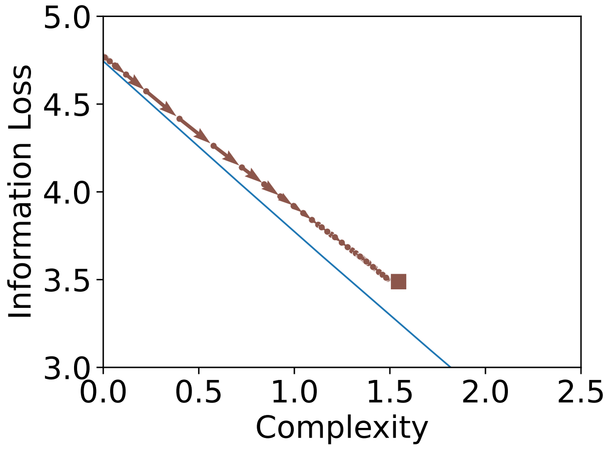

(**b**) Communicative Space

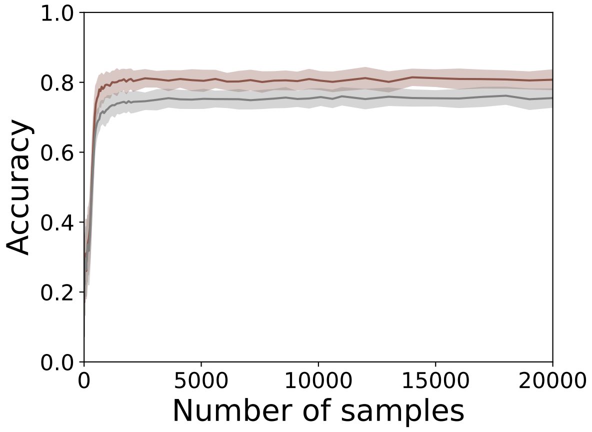

(**c**) Accuracy

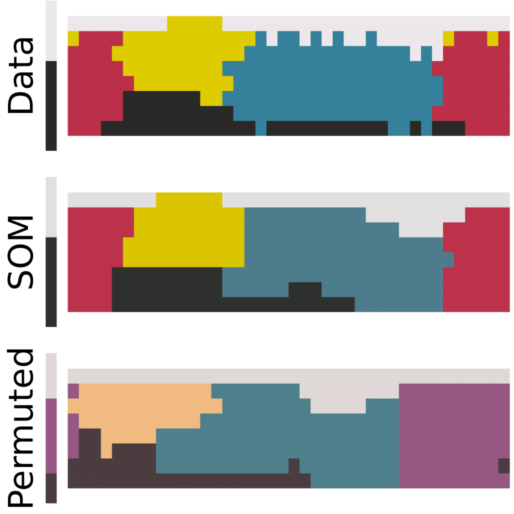

(**d**) Mode Maps

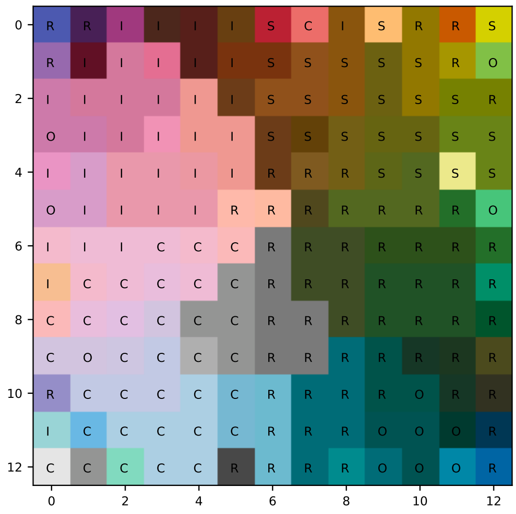

(**e**) Learnt SOM Cells

1.88. Slave; $|\mathcal{W}| = 48$

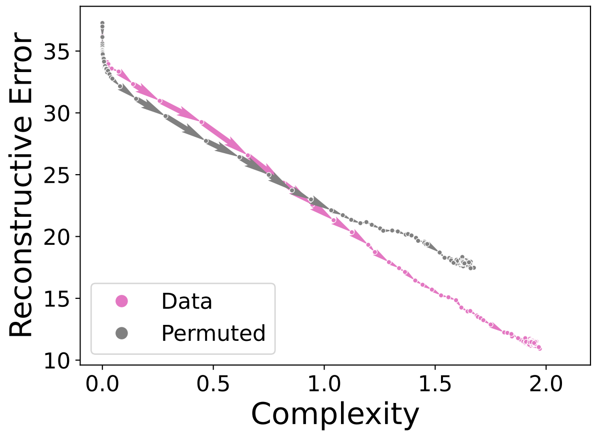

(**a**) Learning Space

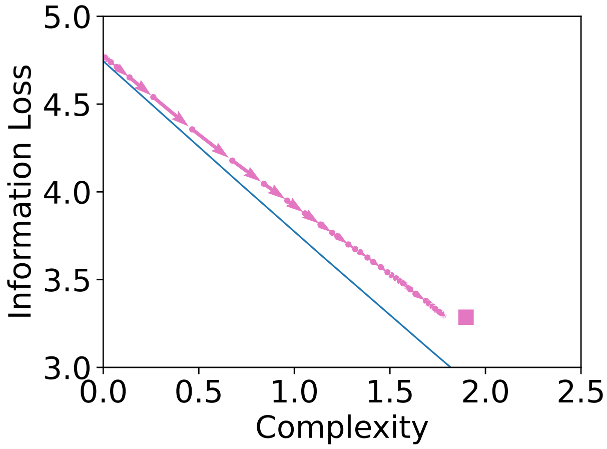

(**b**) Communicative Space

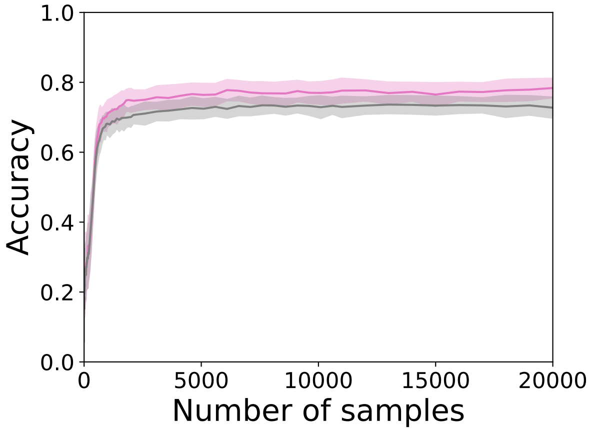

(**c**) Accuracy

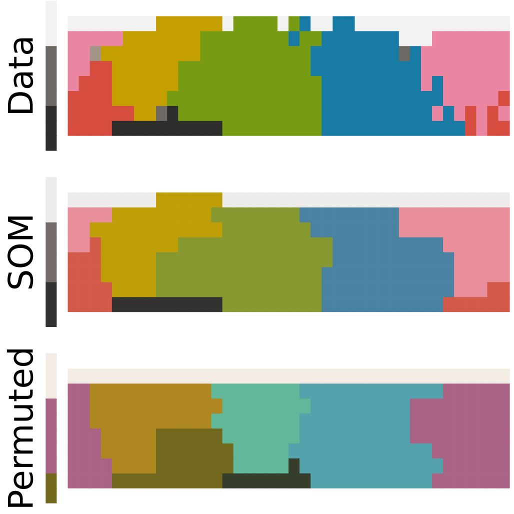

(**d**) Mode Maps

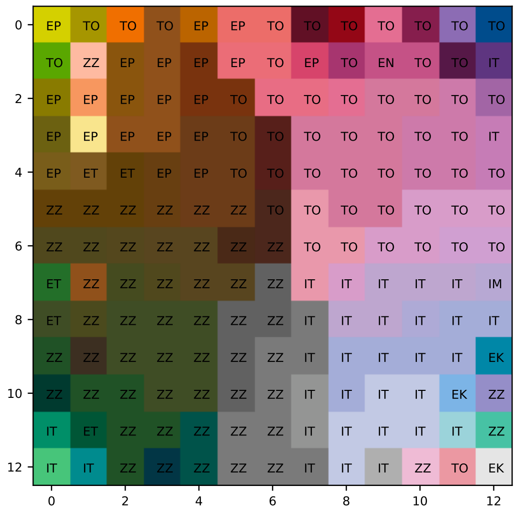

(**e**) Learnt SOM Cells

1.89. Sursurunga; $|\mathcal{W}| = 12$

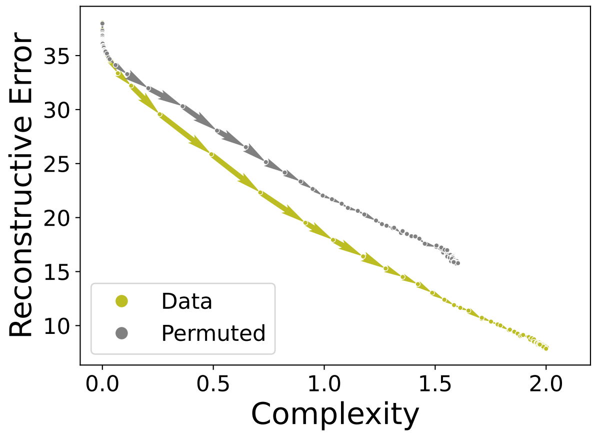

(**a**) Learning Space

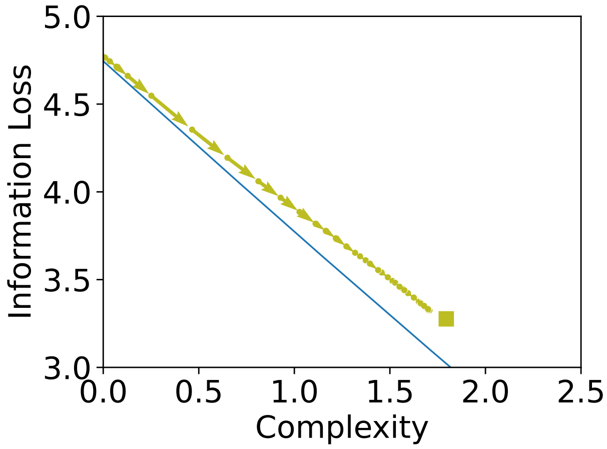

(**b**) Communicative Space

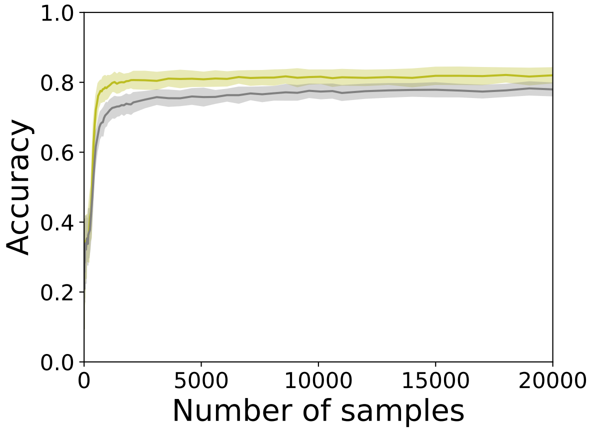

(**c**) Accuracy

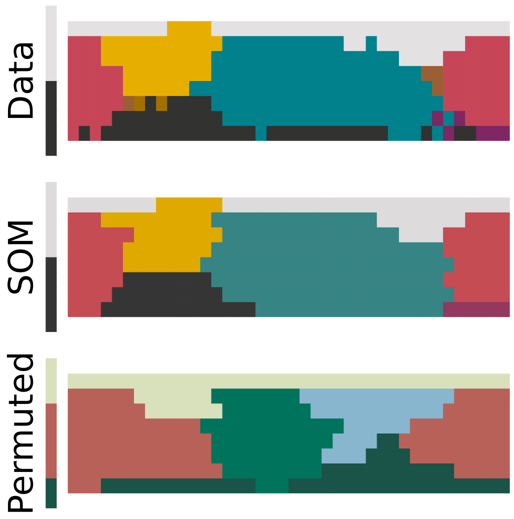

(**d**) Mode Maps

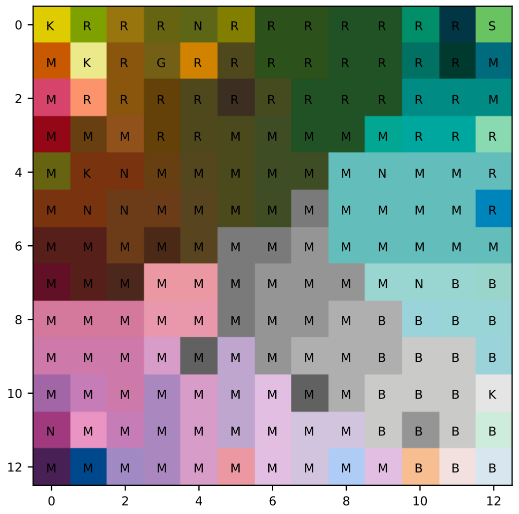

(**e**) Learnt SOM Cells

1.90. Tabla; $|\mathcal{W}| = 8$

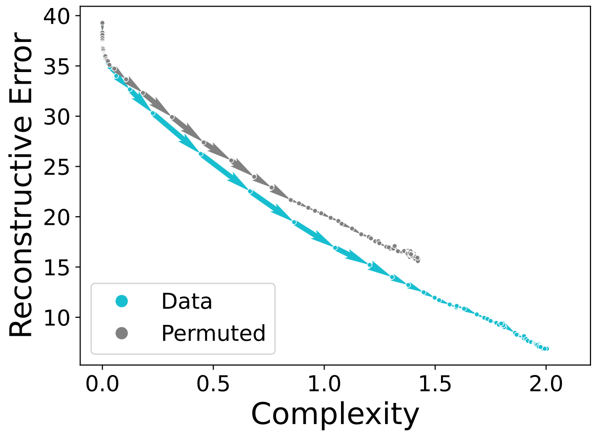

(**a**) Learning Space

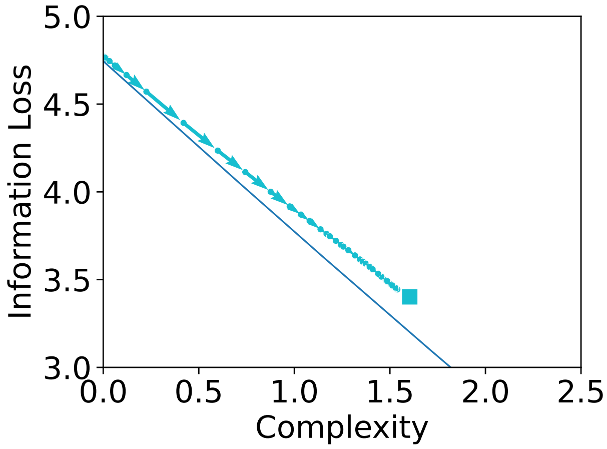

(**b**) Communicative Space

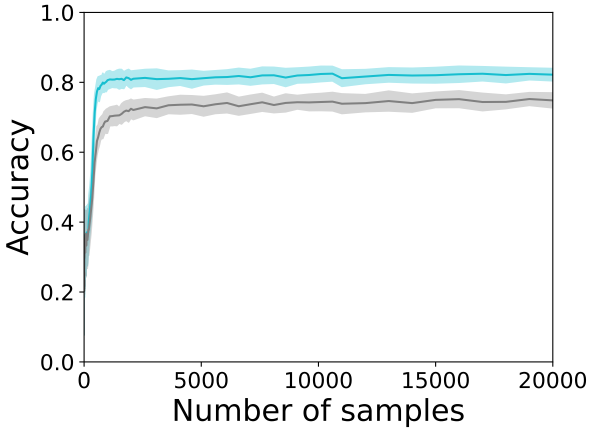

(**c**) Accuracy

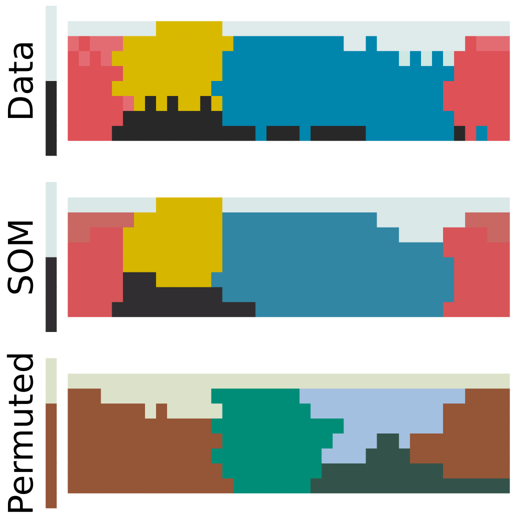

(**d**) Mode Maps

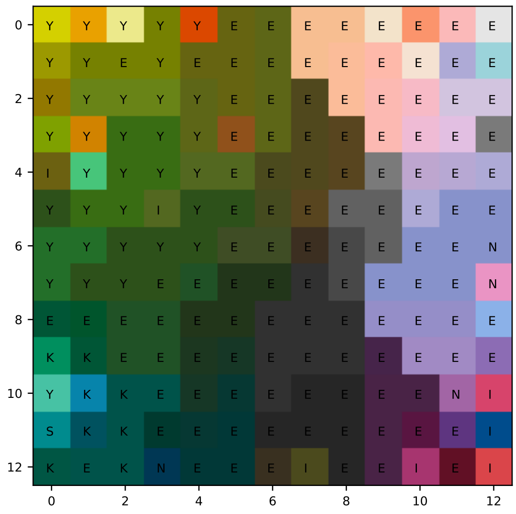

(**e**) Learnt SOM Cells

1.91. Tacana; $|\mathcal{W}| = 32$

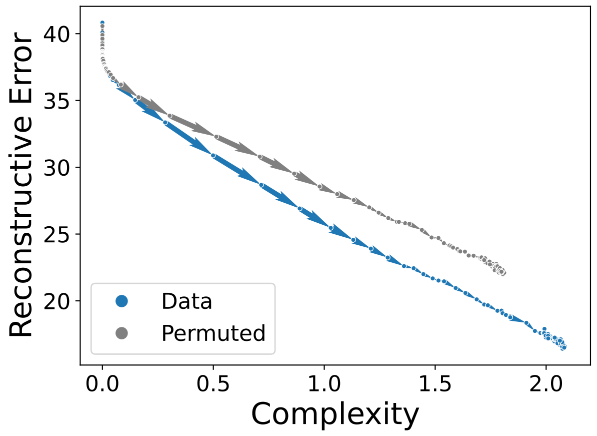

(**a**) Learning Space

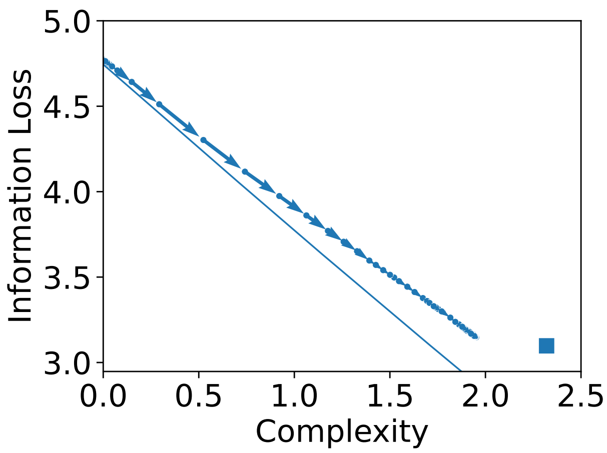

(**b**) Communicative Space

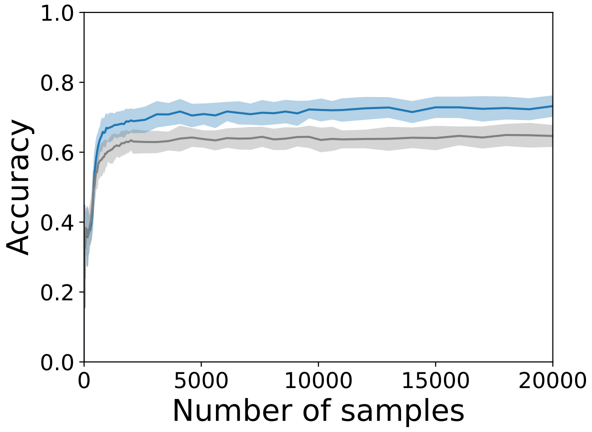

(**c**) Accuracy

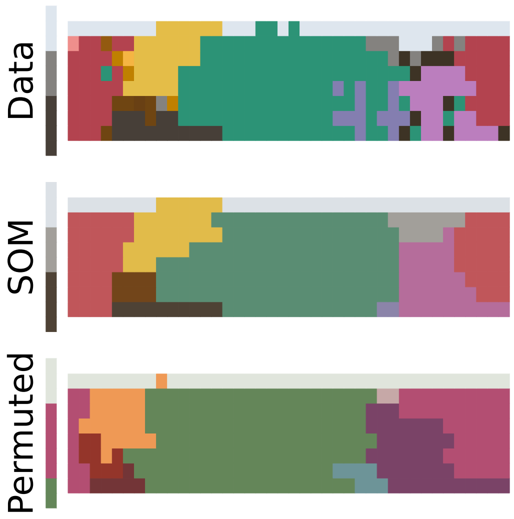

(**d**) Mode Maps

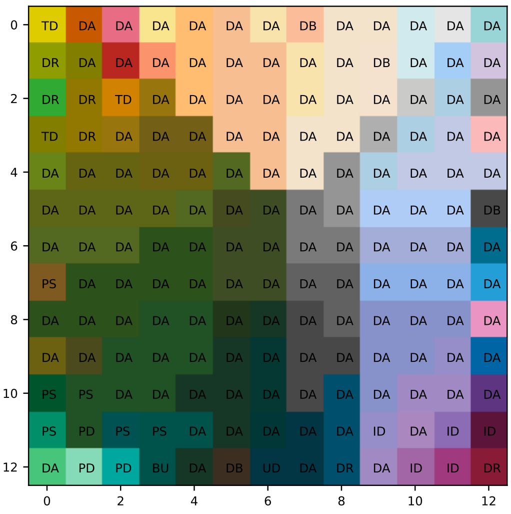

(**e**) Learnt SOM Cells

1.92. Central Tarahumara; $|\mathcal{W}| = 21$

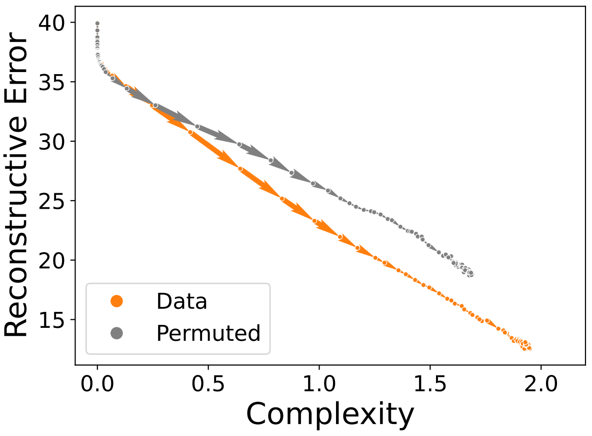

(**a**) Learning Space

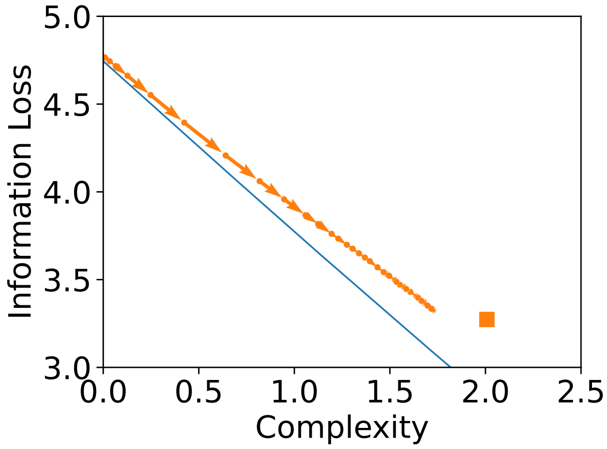

(**b**) Communicative Space

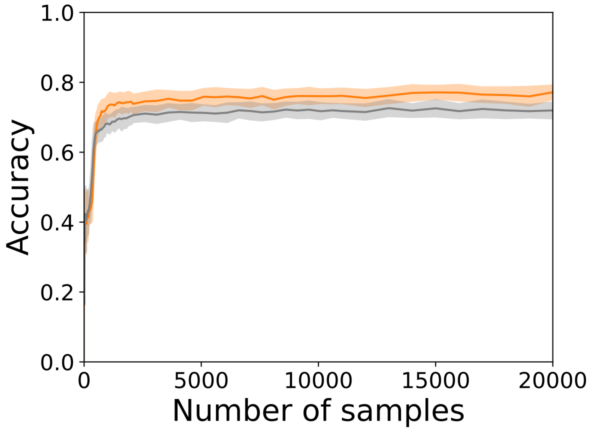

(**c**) Accuracy

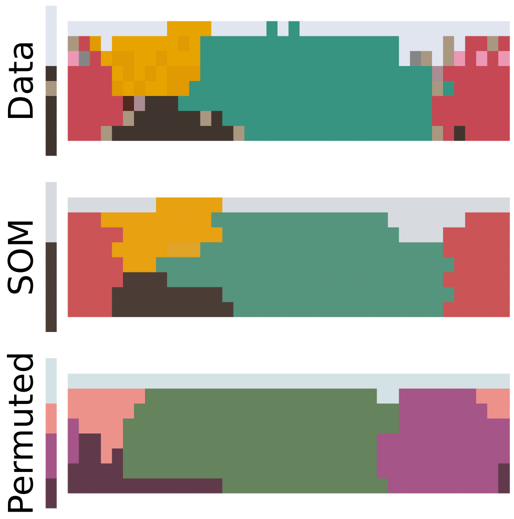

(**d**) Mode Maps

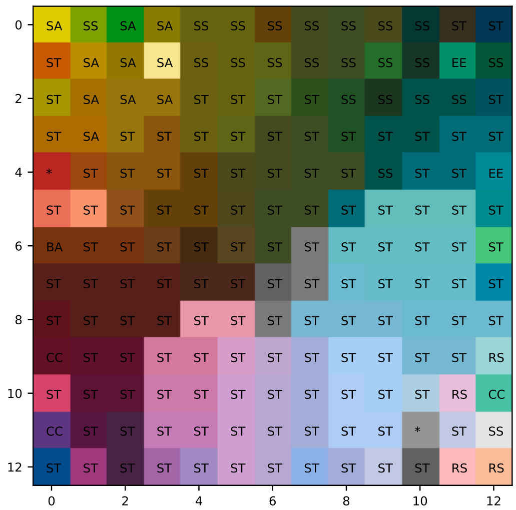

(**e**) Learnt SOM Cells

1.93. W. Tarahumara; $|\mathcal{W}| = 11$

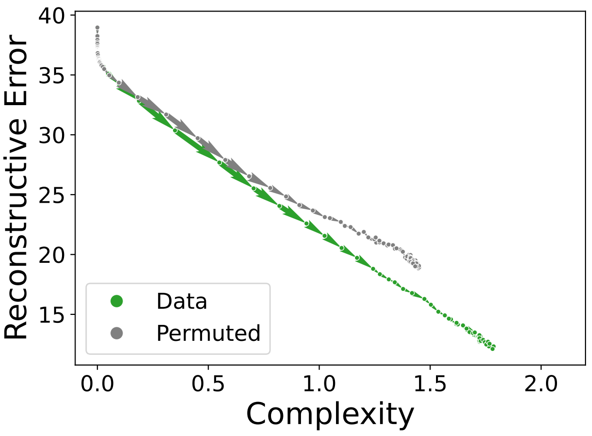

(**a**) Learning Space

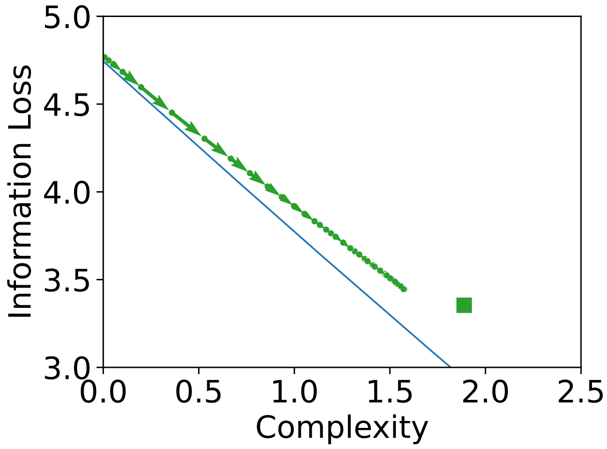

(**b**) Communicative Space

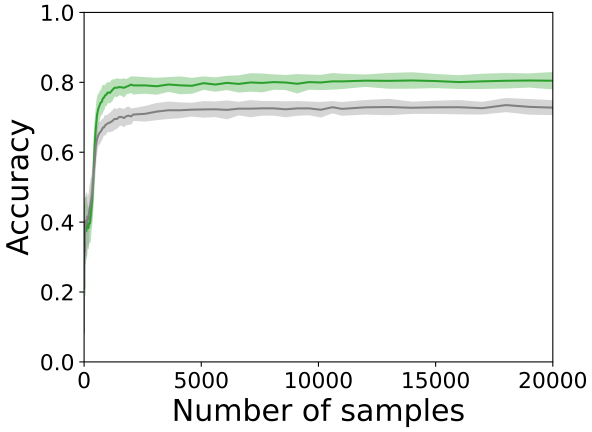

(**c**) Accuracy

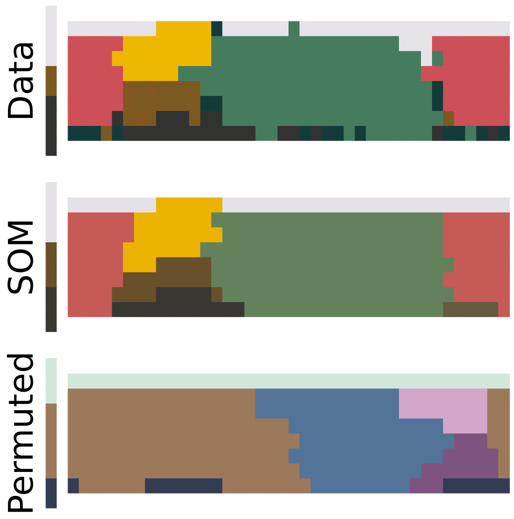

(**d**) Mode Maps

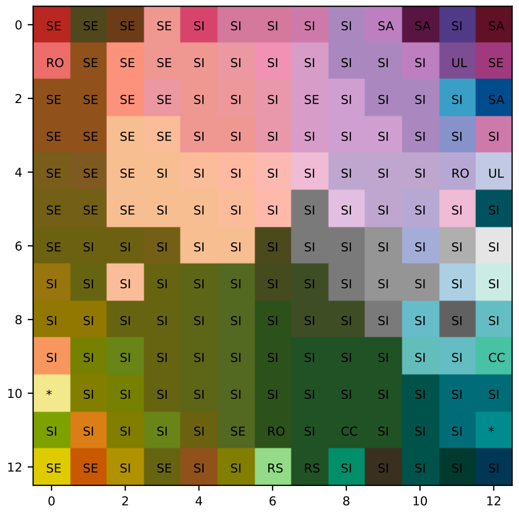

(**e**) Learnt SOM Cells

1.94. Tboli; $|\mathcal{W}| = 10$

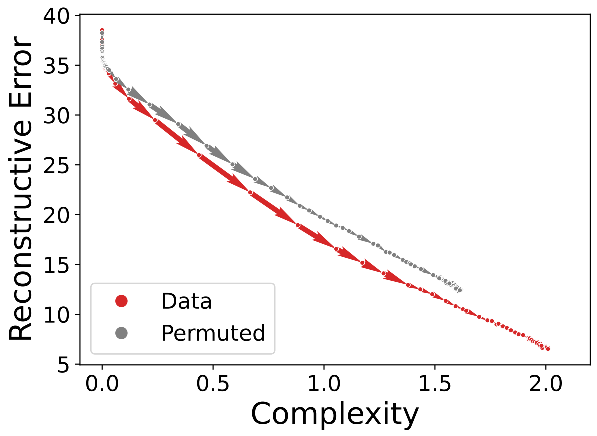

(**a**) Learning Space

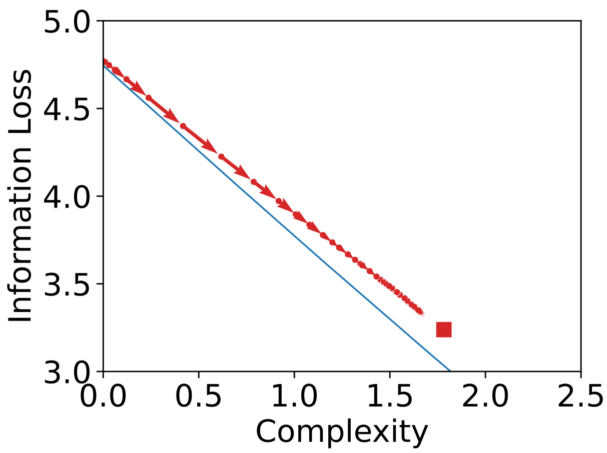

(**b**) Communicative Space

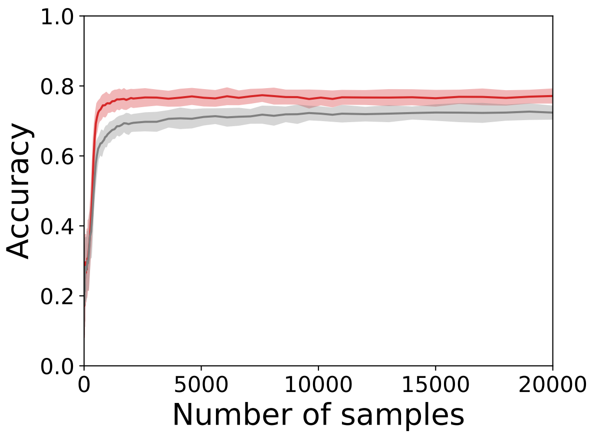

(**c**) Accuracy

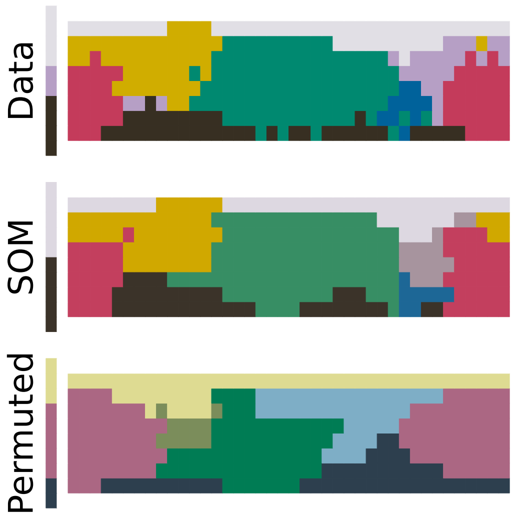

(**d**) Mode Maps

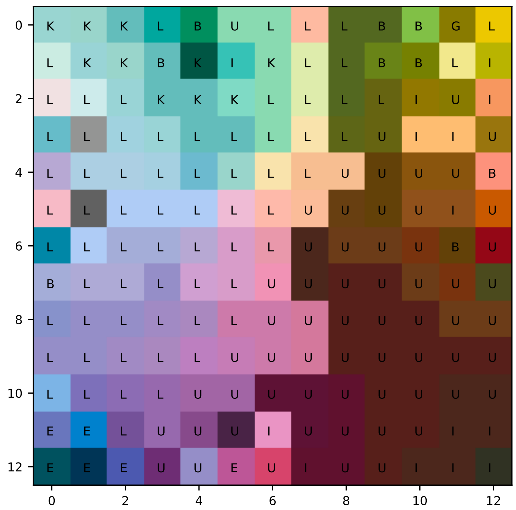

(**e**) Learnt SOM Cells

1.95. Teribe; $|\mathcal{W}| = 22$

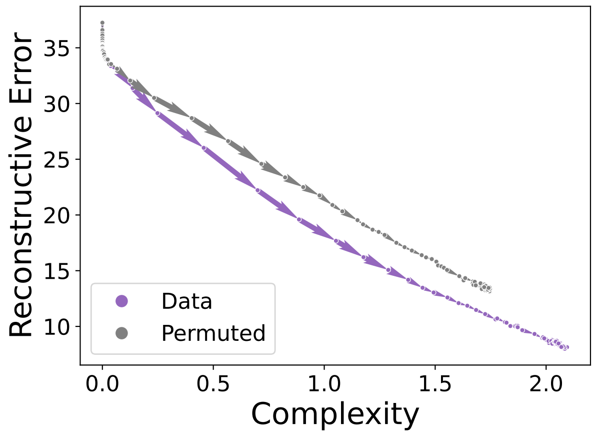

(**a**) Learning Space

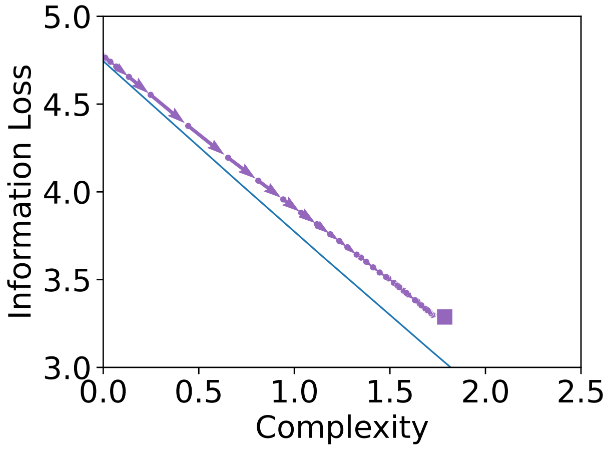

(**b**) Communicative Space

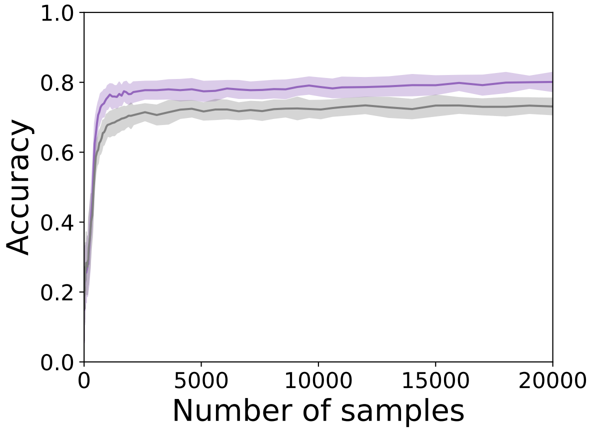

(**c**) Accuracy

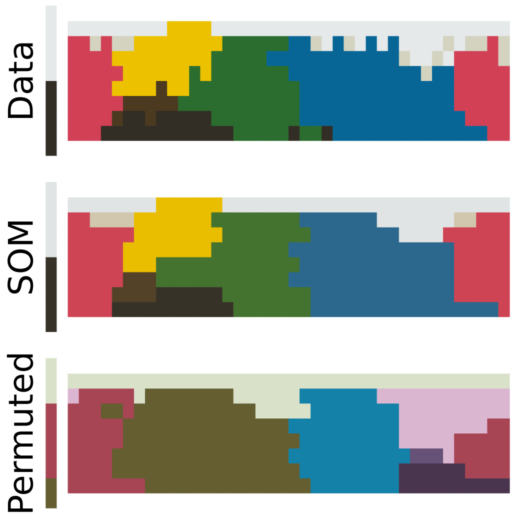

(**d**) Mode Maps

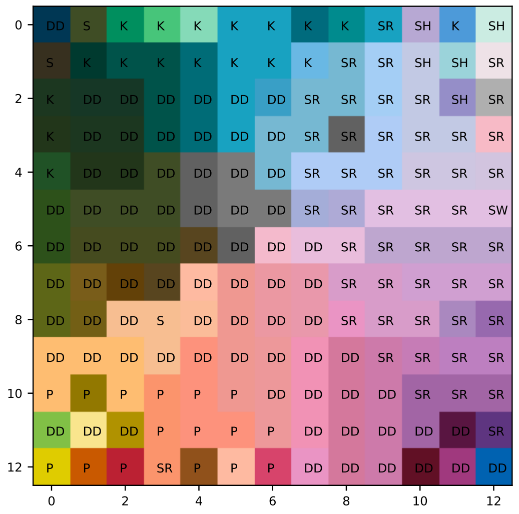

(**e**) Learnt SOM Cells

1.96. Ticuna; $|\mathcal{W}| = 11$

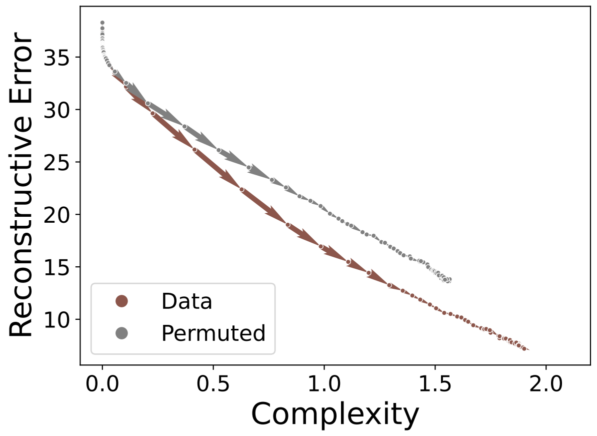

(**a**) Learning Space

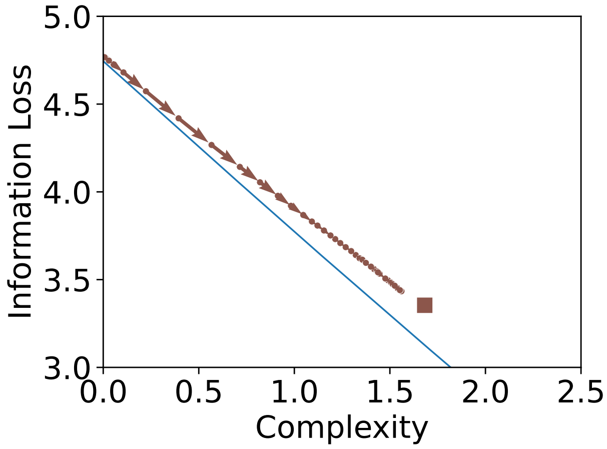

(**b**) Communicative Space

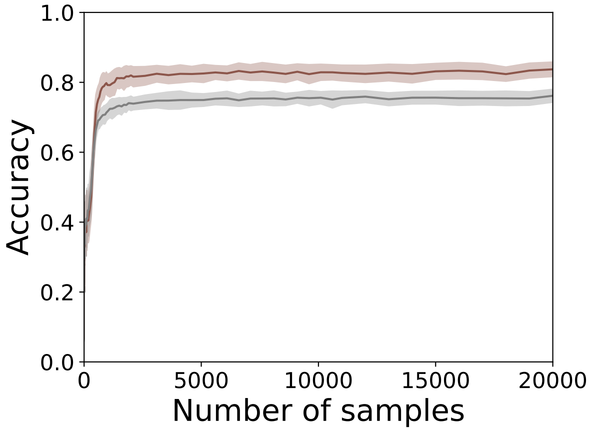

(**c**) Accuracy

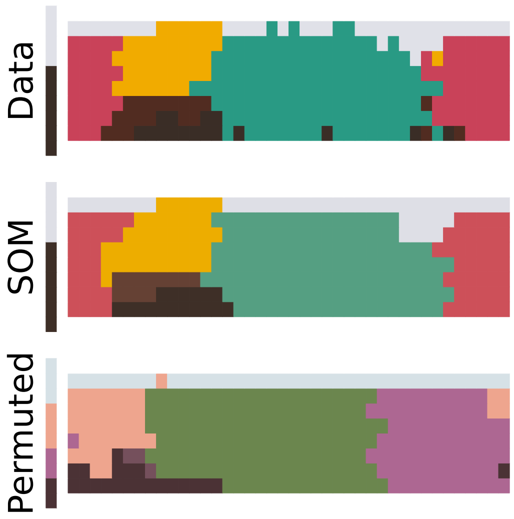

(**d**) Mode Maps

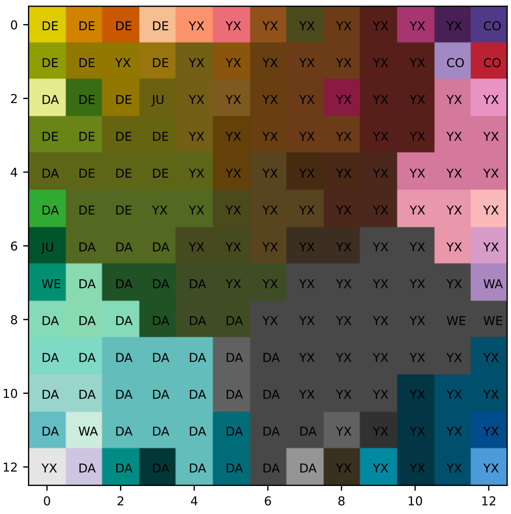

(**e**) Learnt SOM Cells

1.97. Tifal; $|\mathcal{W}| = 25$

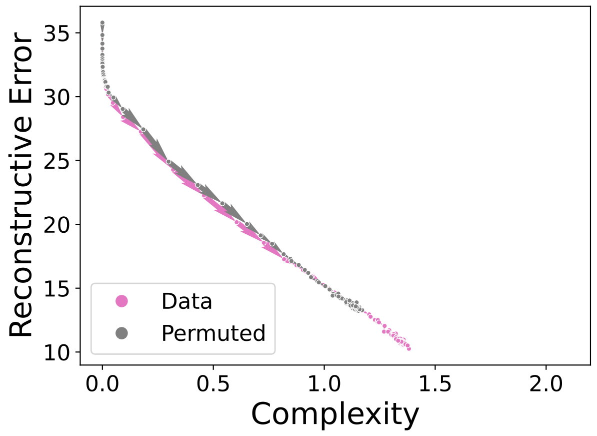

(**a**) Learning Space

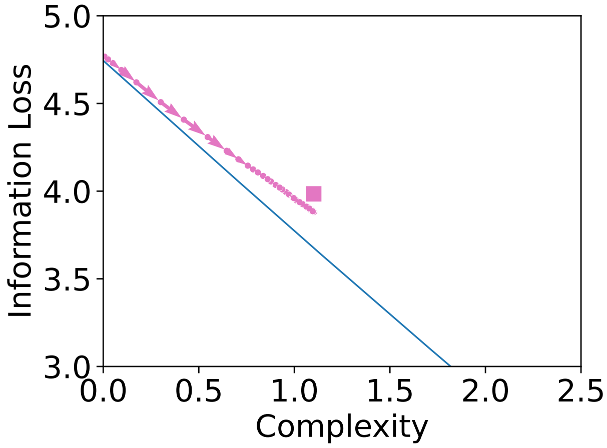

(**b**) Communicative Space

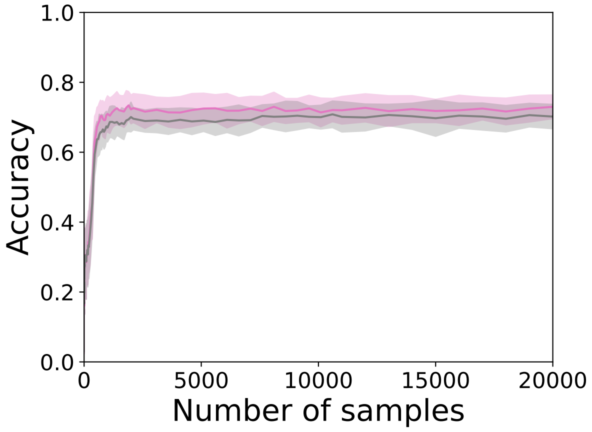

(**c**) Accuracy

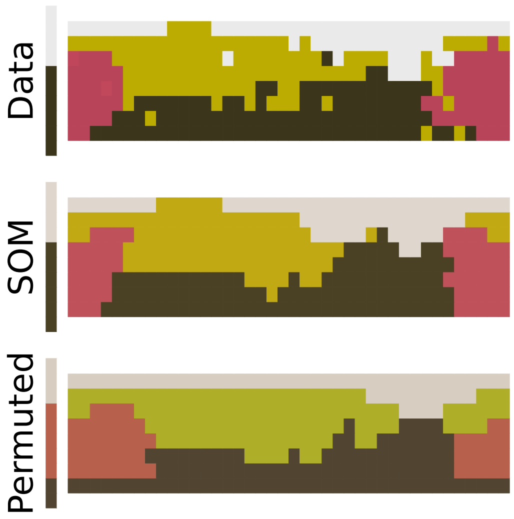

(**d**) Mode Maps

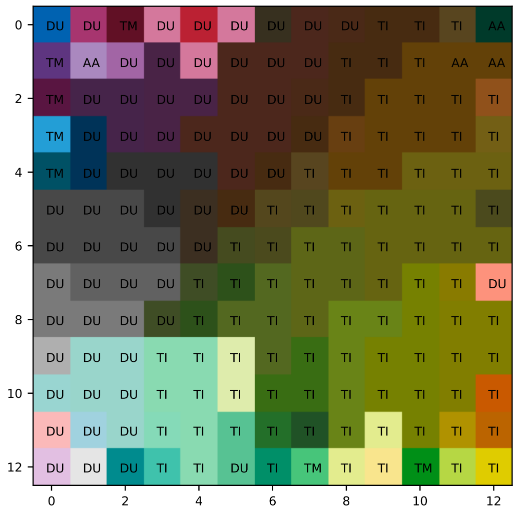

(**e**) Learnt SOM Cells

1.98. Tlapaneco; $|\mathcal{W}| = 14$

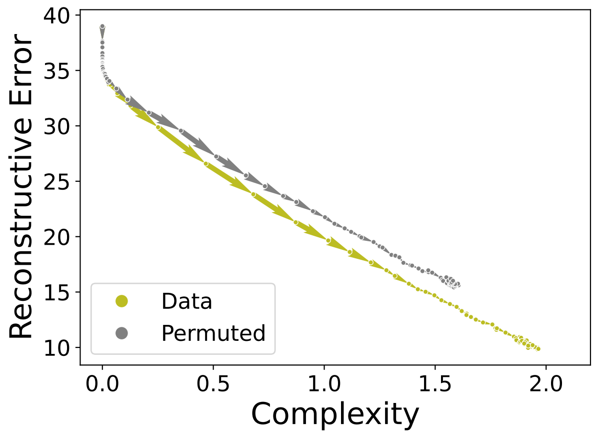

(**a**) Learning Space

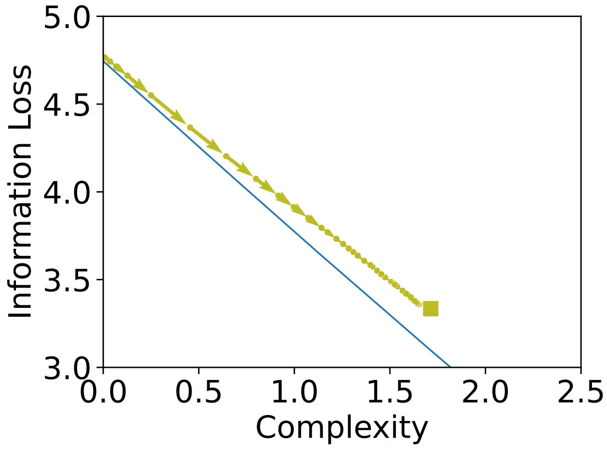

(**b**) Communicative Space

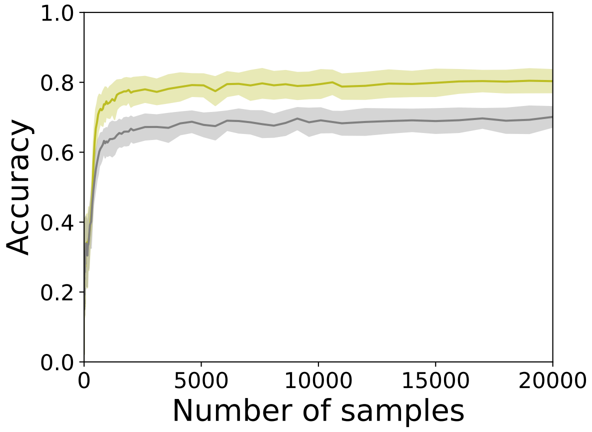

(**c**) Accuracy

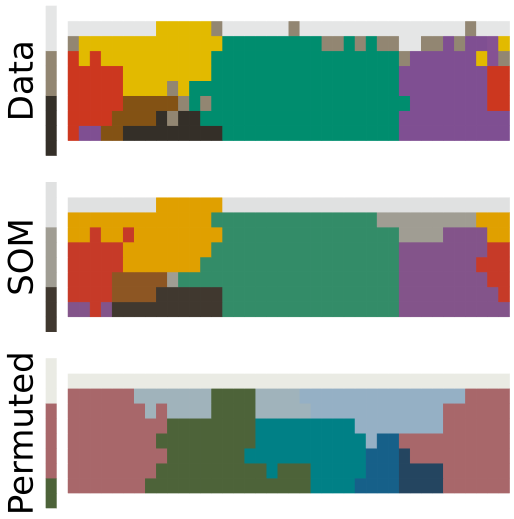

(**d**) Mode Maps

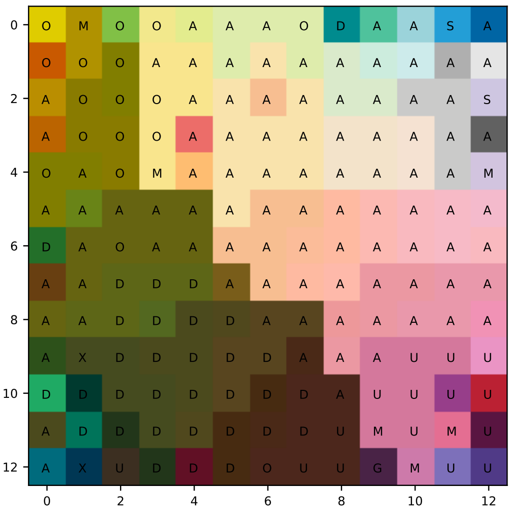

(**e**) Learnt SOM Cells

1.99. Tucano; $|\mathcal{W}| = 7$

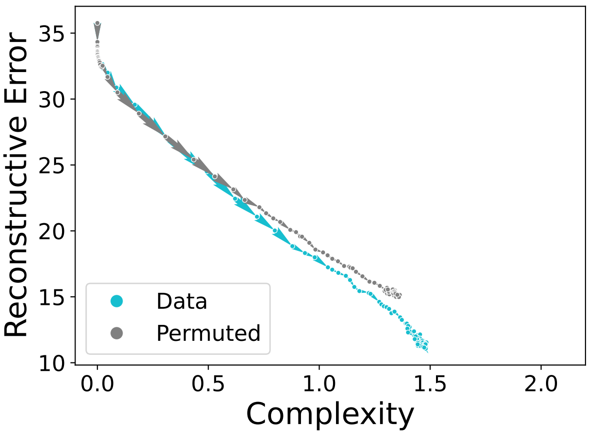

(**a**) Learning Space

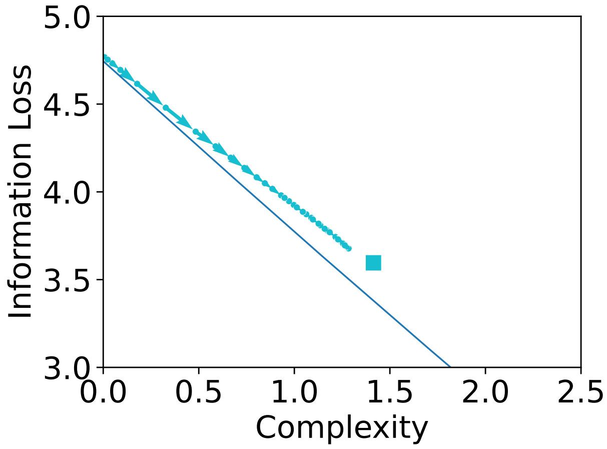

(**b**) Communicative Space

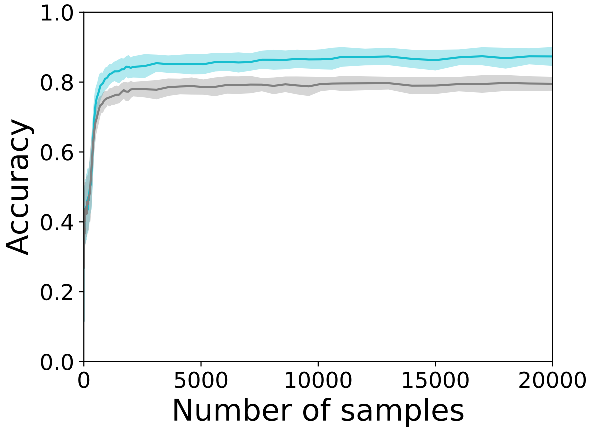

(**c**) Accuracy

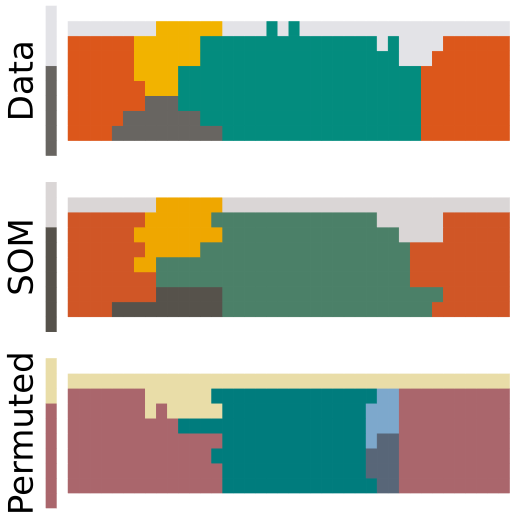

(**d**) Mode Maps

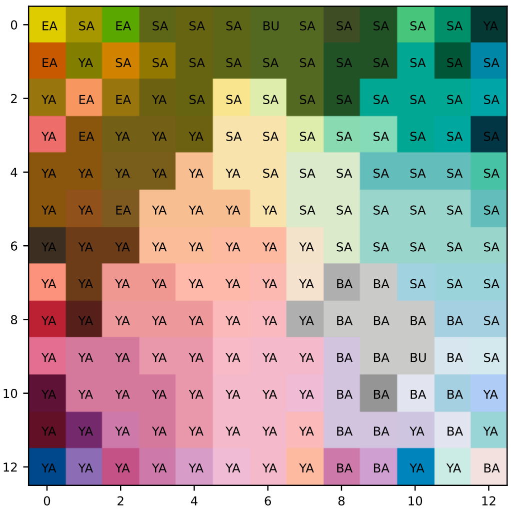

(**e**) Learnt SOM Cells

1.100. Vagla; $|\mathcal{W}| = 7$

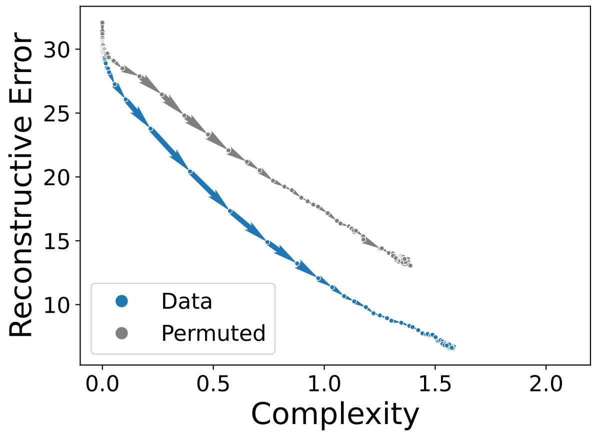

(**a**) Learning Space

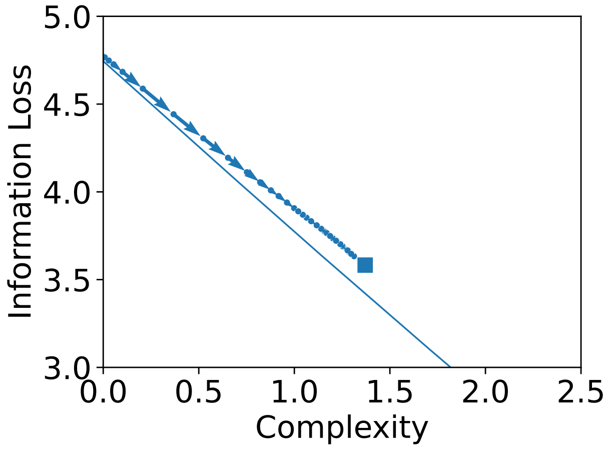

(**b**) Communicative Space

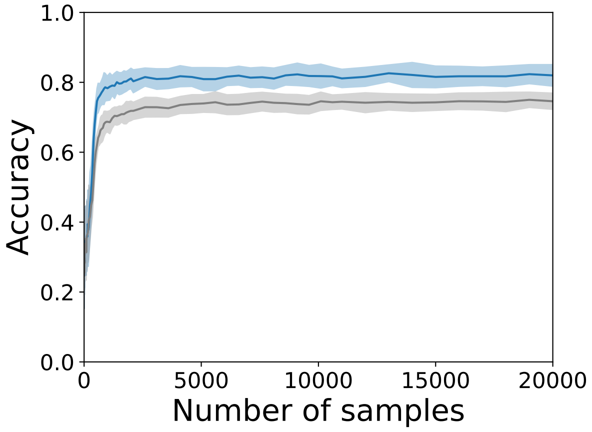

(**c**) Accuracy

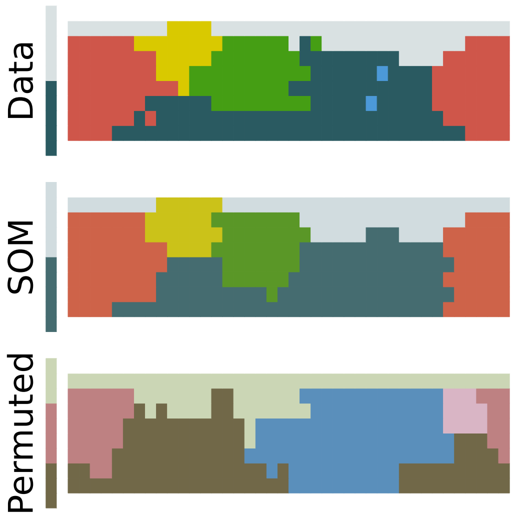

(**d**) Mode Maps

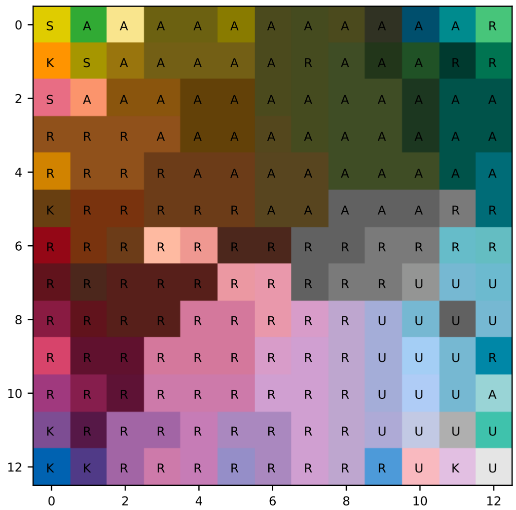

(**e**) Learnt SOM Cells

1.101. Vasavi; $|\mathcal{W}| = 11$

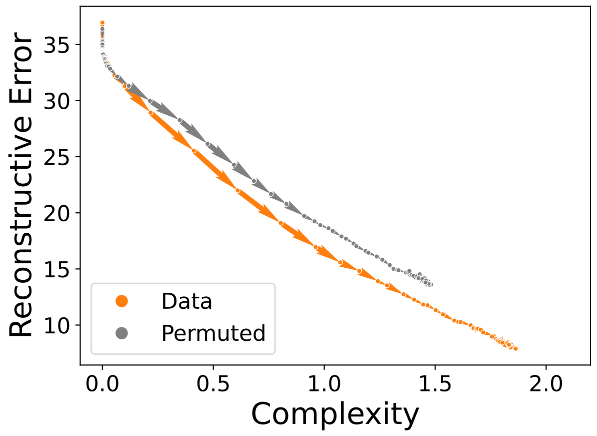

(**a**) Learning Space

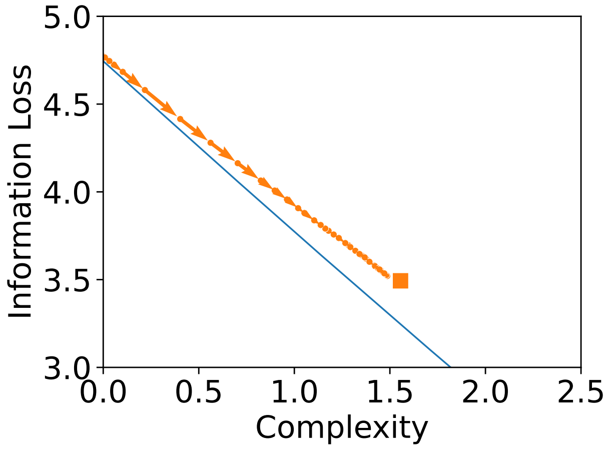

(**b**) Communicative Space

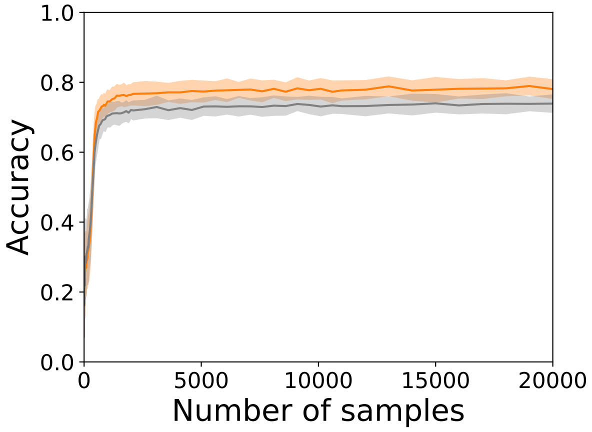

(**c**) Accuracy

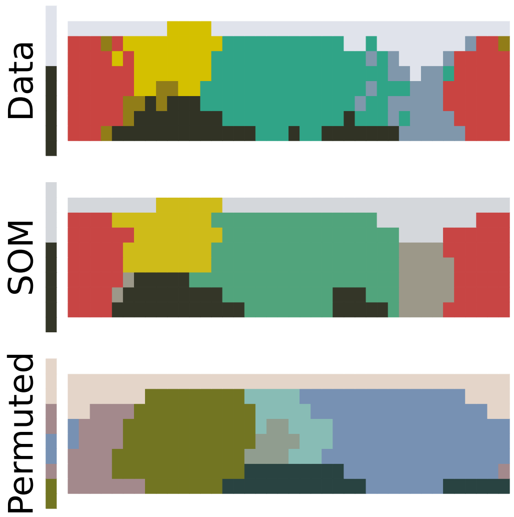

(**d**) Mode Maps

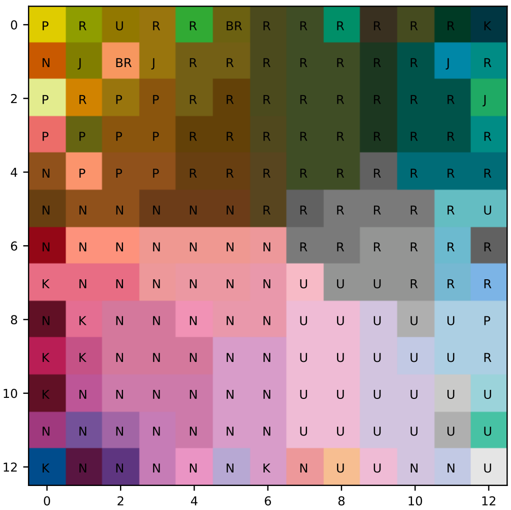

(**e**) Learnt SOM Cells

1.102. Waorani; $|\mathcal{W}| = 19$

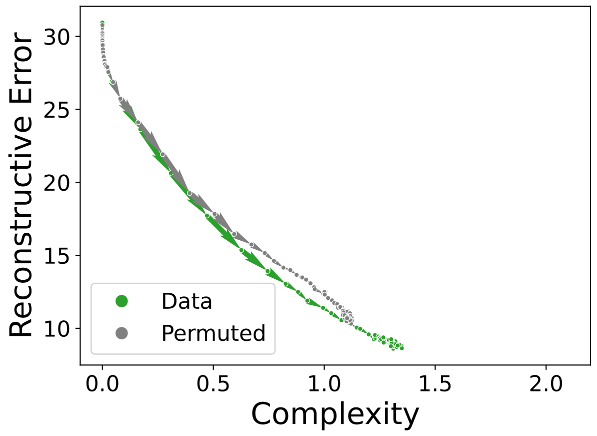

(**a**) Learning Space

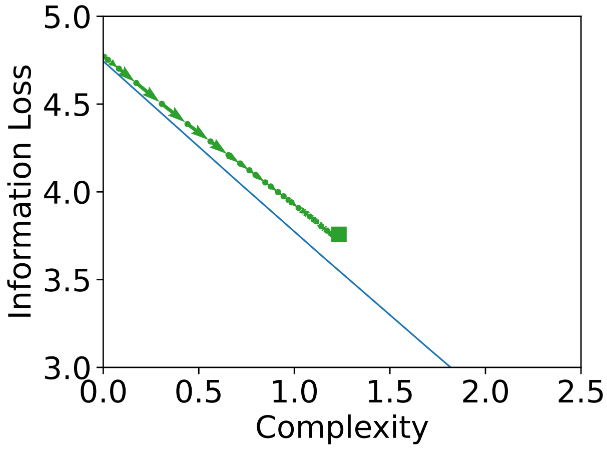

(**b**) Communicative Space

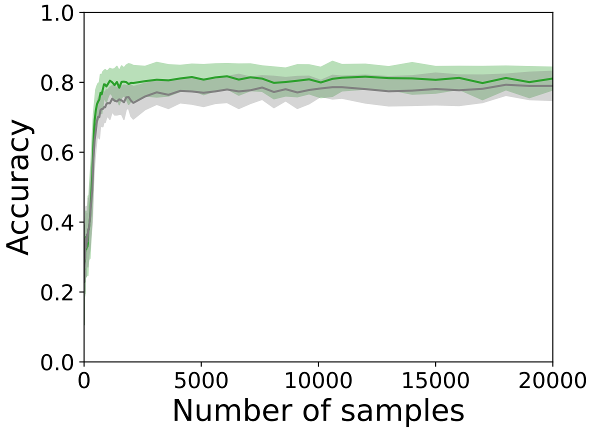

(**c**) Accuracy

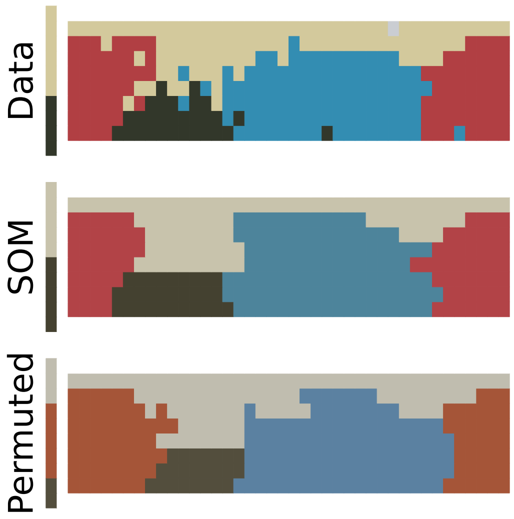

(**d**) Mode Maps

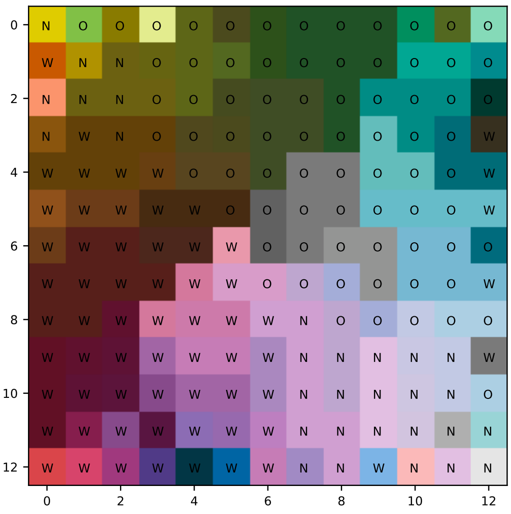

(**e**) Learnt SOM Cells

1.103. Walpiri; $|\mathcal{W}| = 63$

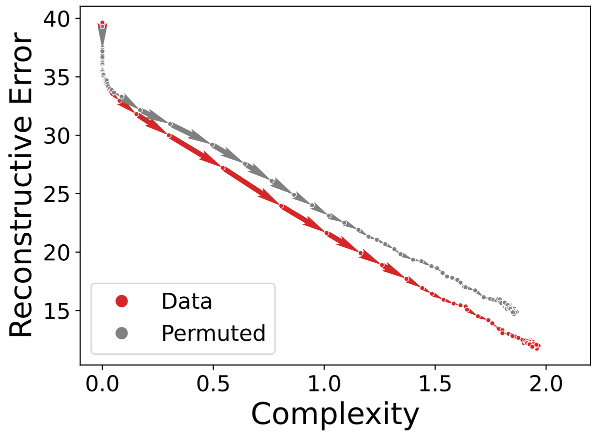

(**a**) Learning Space

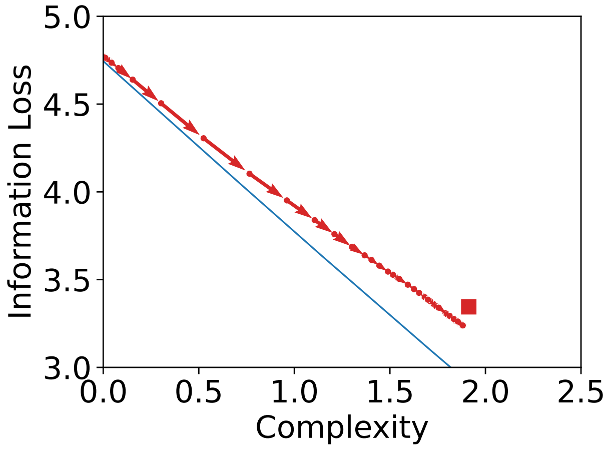

(**b**) Communicative Space

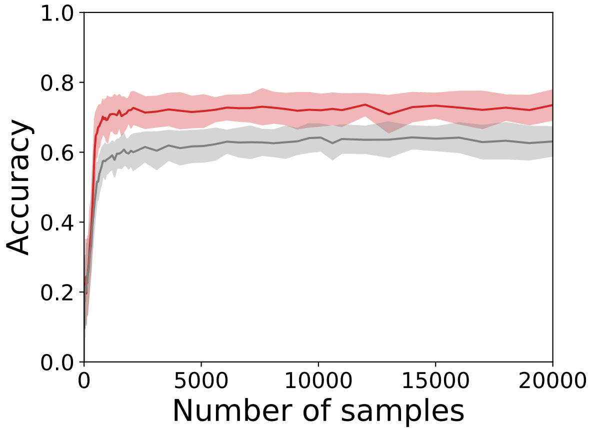

(**c**) Accuracy

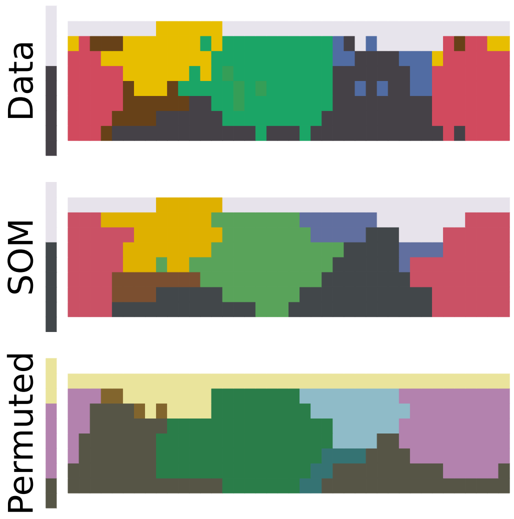

(**d**) Mode Maps

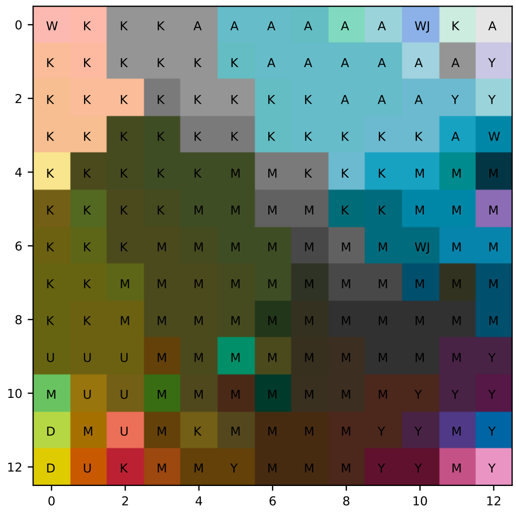

(**e**) Learnt SOM Cells

1.104. Wobe; $|\mathcal{W}| = 6$

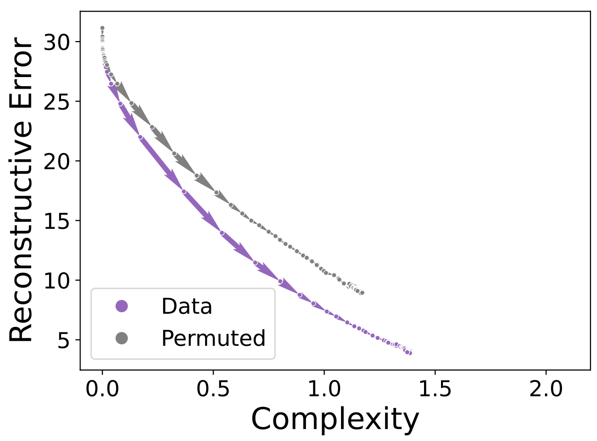

(**a**) Learning Space

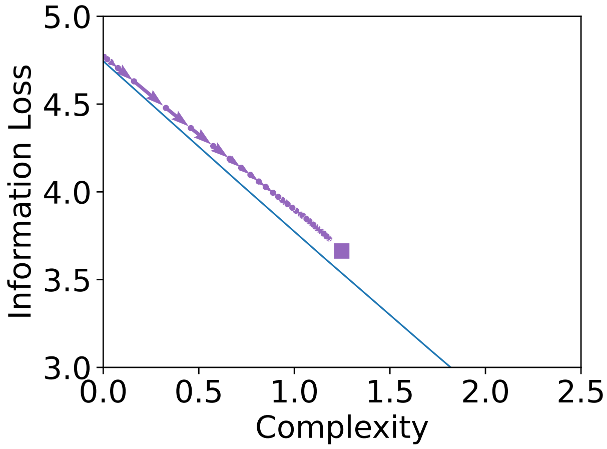

(**b**) Communicative Space

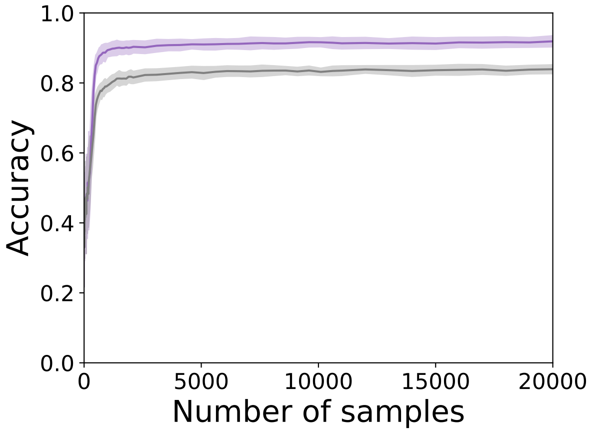

(**c**) Accuracy

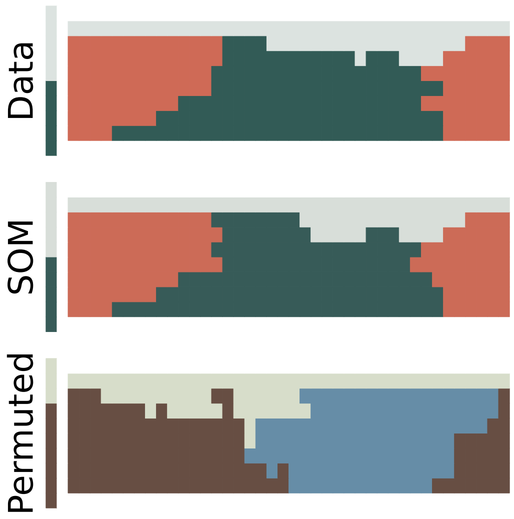

(**d**) Mode Maps

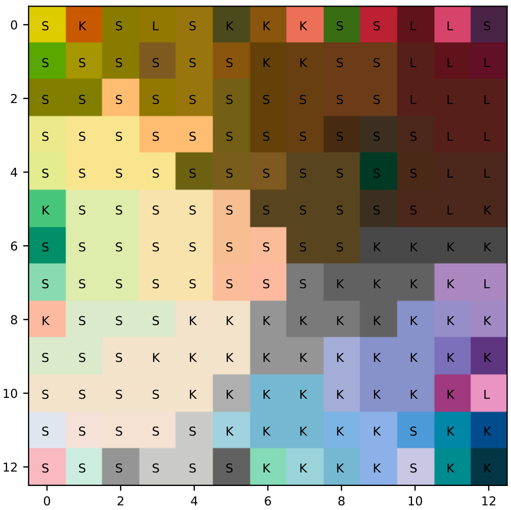

(**e**) Learnt SOM Cells

1.105. Yacouba; $|\mathcal{W}| = 4$

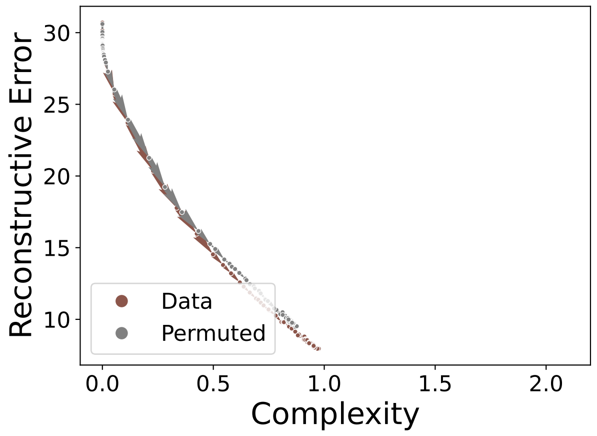

(**a**) Learning Space

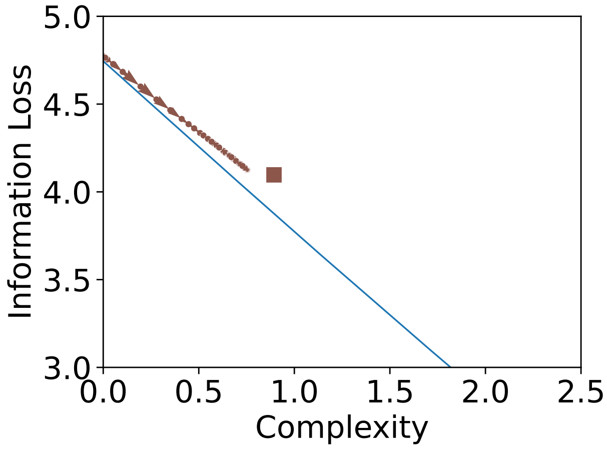

(**b**) Communicative Space

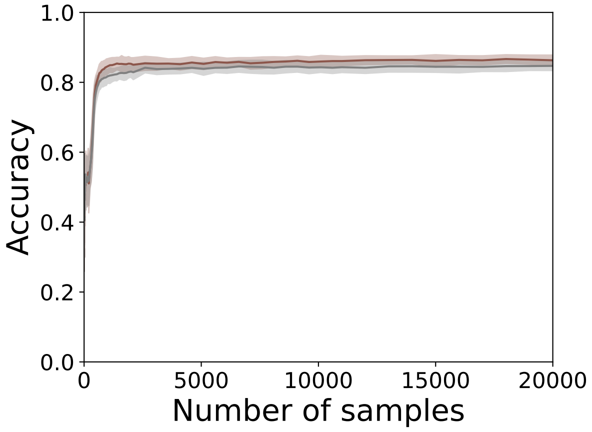

(**c**) Accuracy

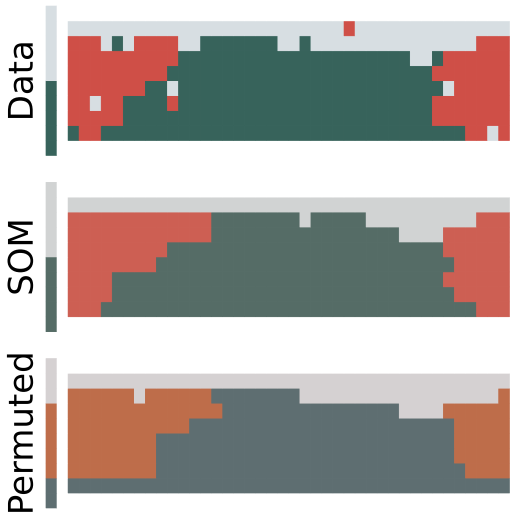

(**d**) Mode Maps

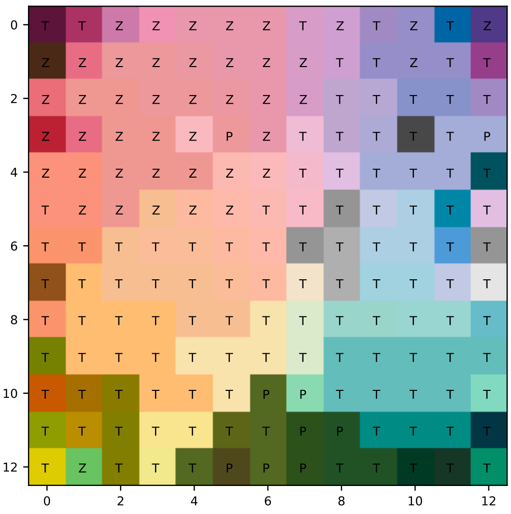

(**e**) Learnt SOM Cells

1.106. Yakan; $|\mathcal{W}| = 14$

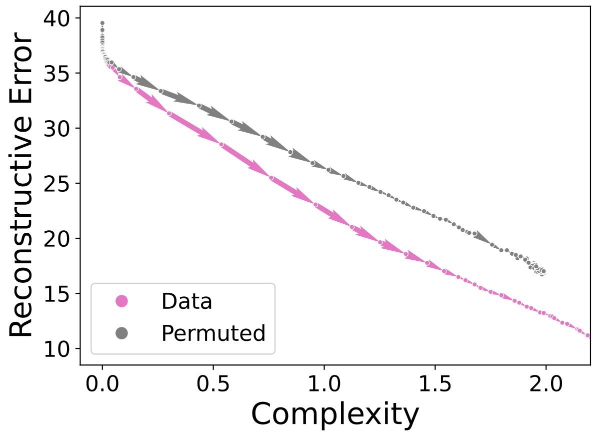

(**a**) Learning Space

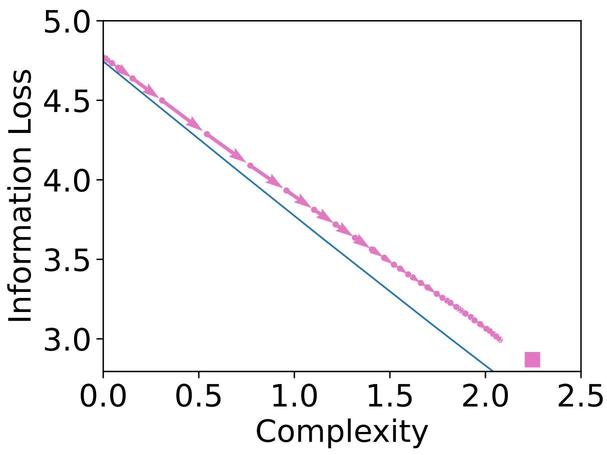

(**b**) Communicative Space

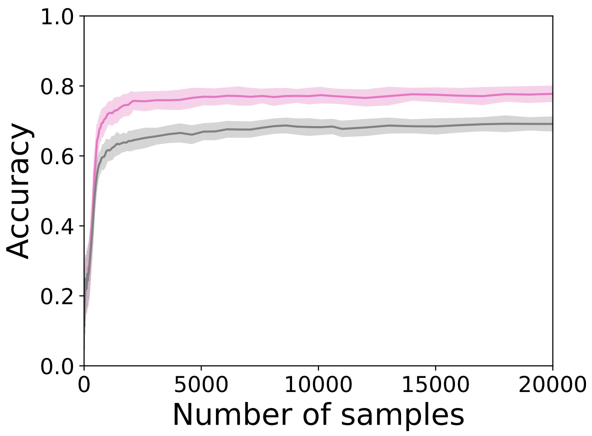

(**c**) Accuracy

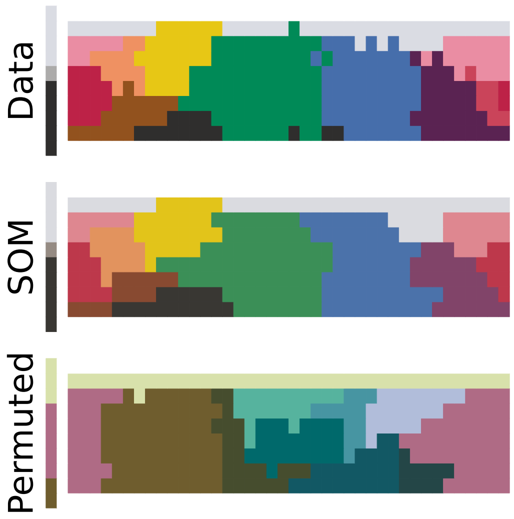

(**d**) Mode Maps

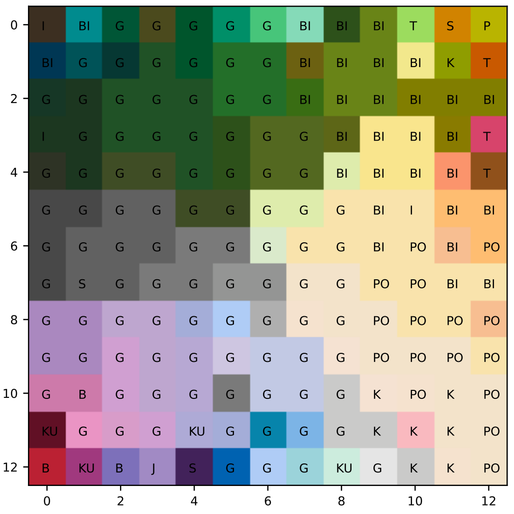

(**e**) Learnt SOM Cells

1.107. Yaminahua; $|\mathcal{W}| = 17$

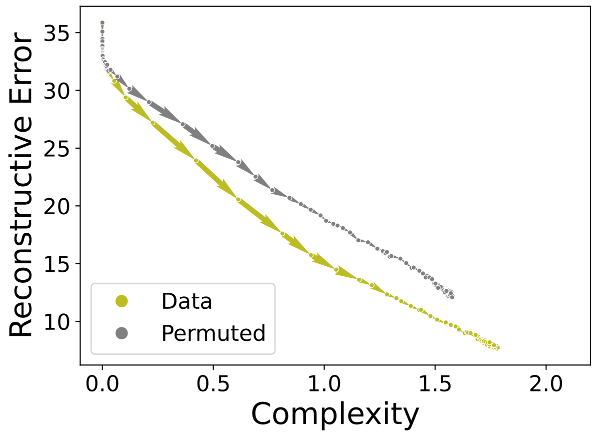

(**a**) Learning Space

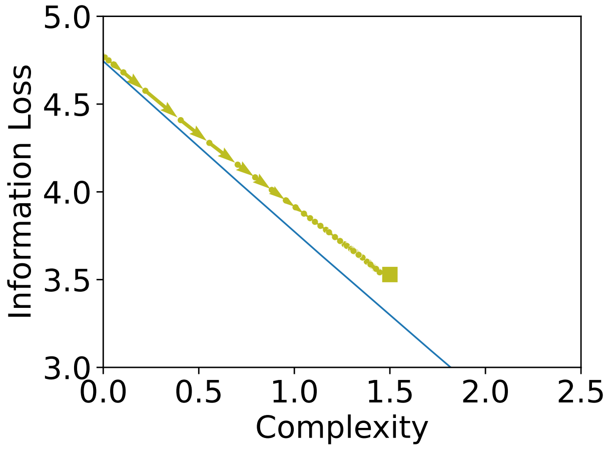

(**b**) Communicative Space

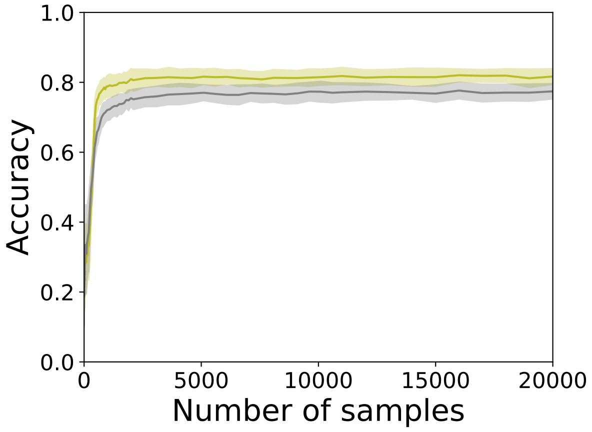

(**c**) Accuracy

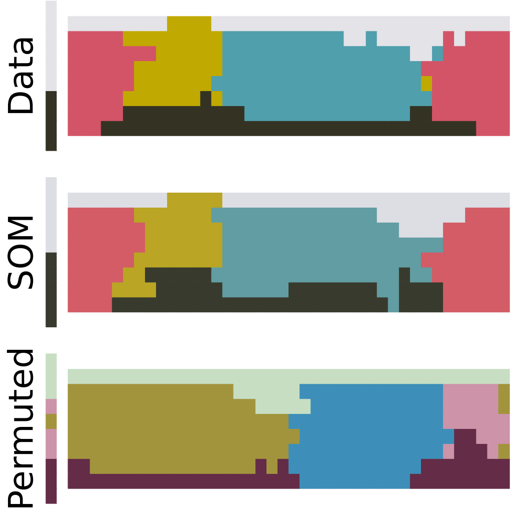

(**d**) Mode Maps

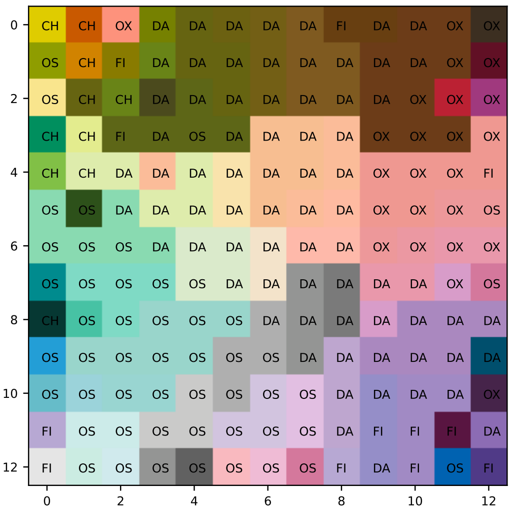

(**e**) Learnt SOM Cells

1.108. Yucuna; $|\mathcal{W}| = 9$

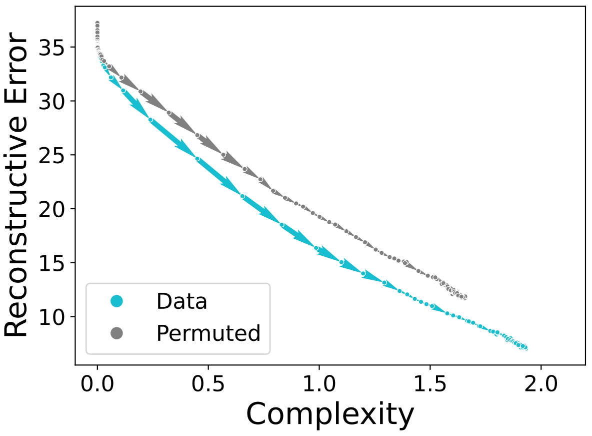

(**a**) Learning Space

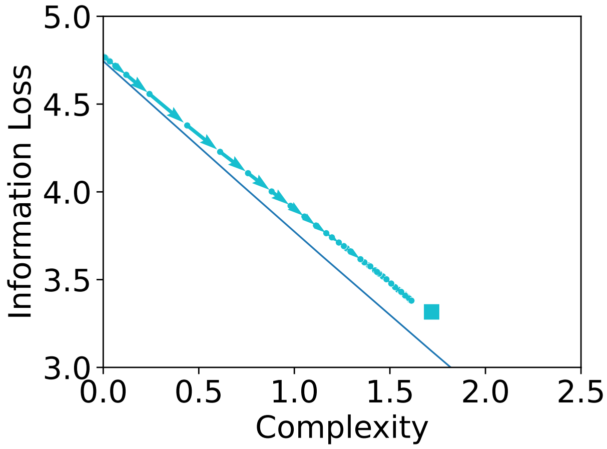

(**b**) Communicative Space

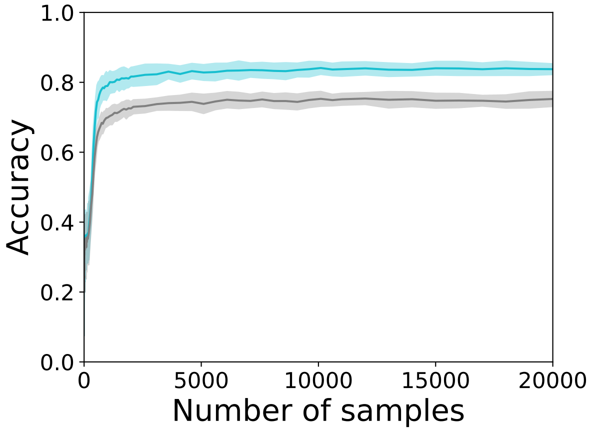

(**c**) Accuracy

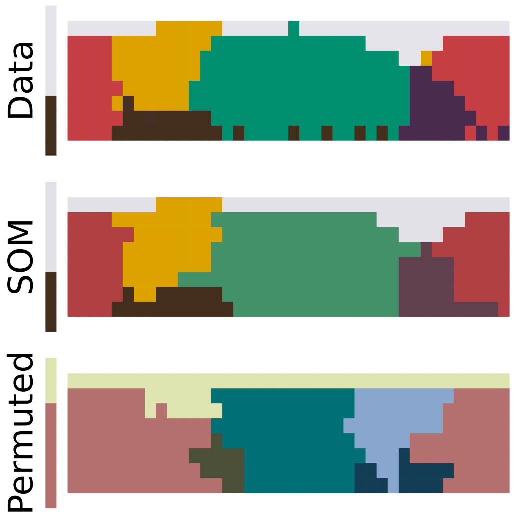

(**d**) Mode Maps

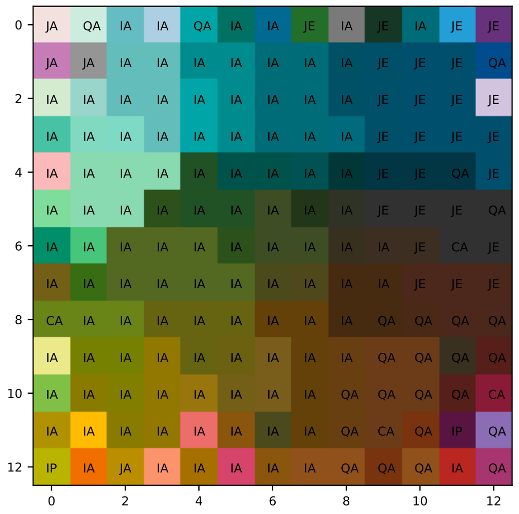

(**e**) Learnt SOM Cells

1.109. Yupik; $|\mathcal{W}| = 26$

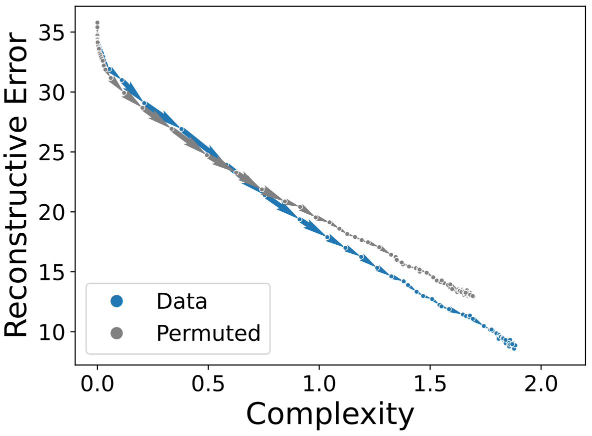

(**a**) Learning Space

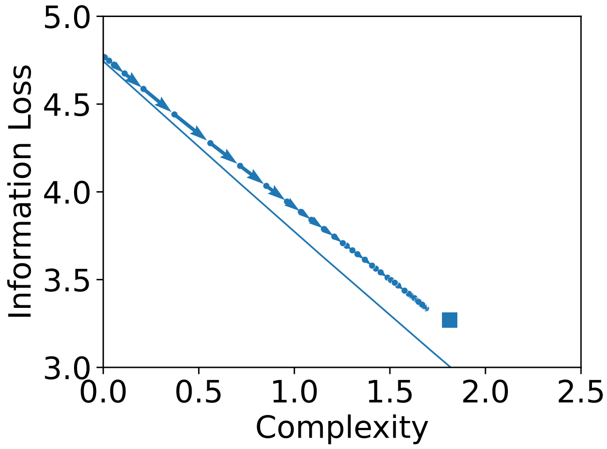

(**b**) Communicative Space

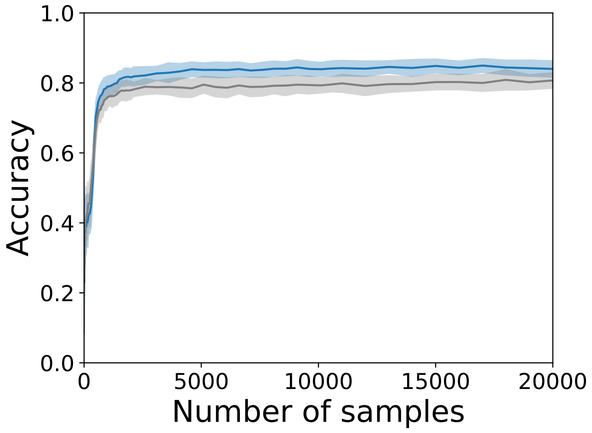

(**c**) Accuracy

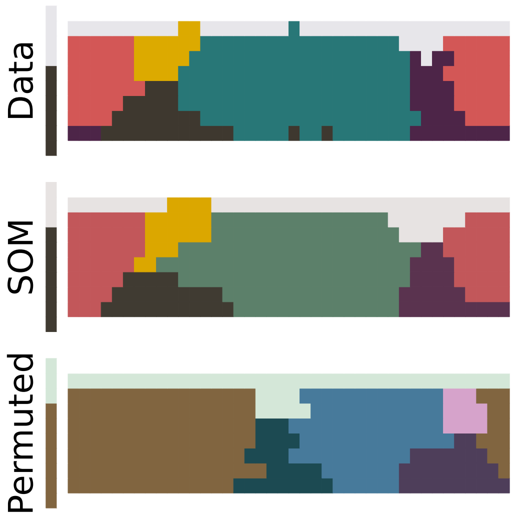

(**d**) Mode Maps

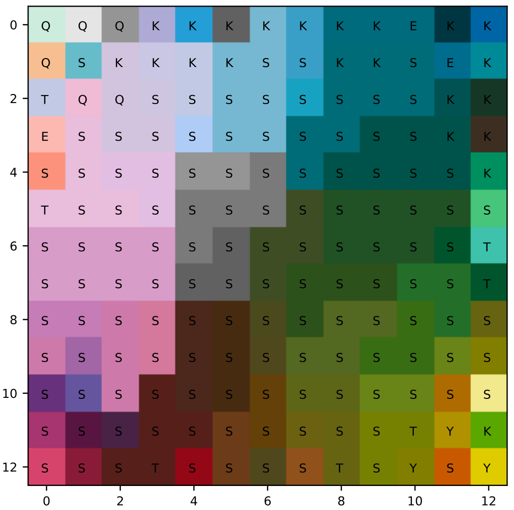

(**e**) Learnt SOM Cells

1.110. Zapoteco; $|\mathcal{W}| = 13$

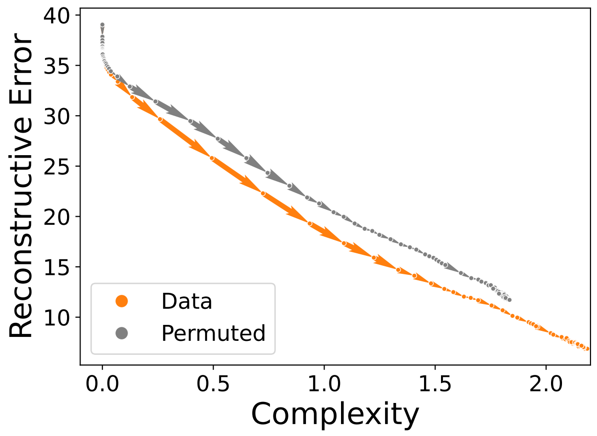

(**a**) Learning Space

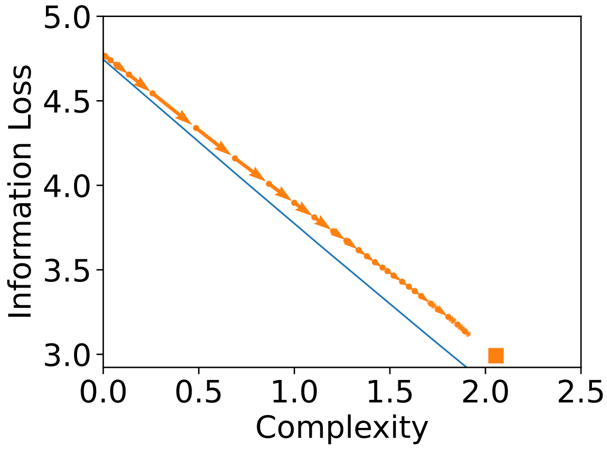

(**b**) Communicative Space

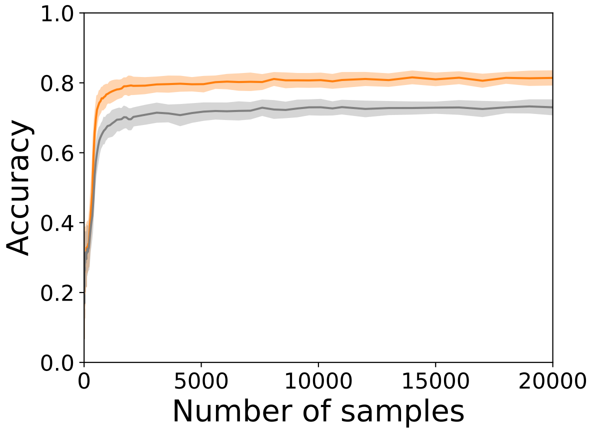

(**c**) Accuracy

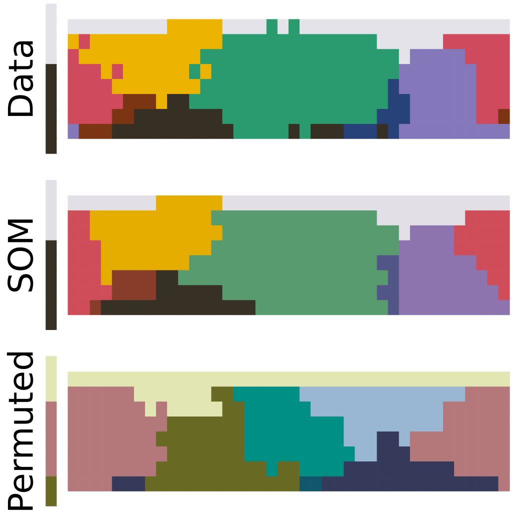

(**d**) Mode Maps

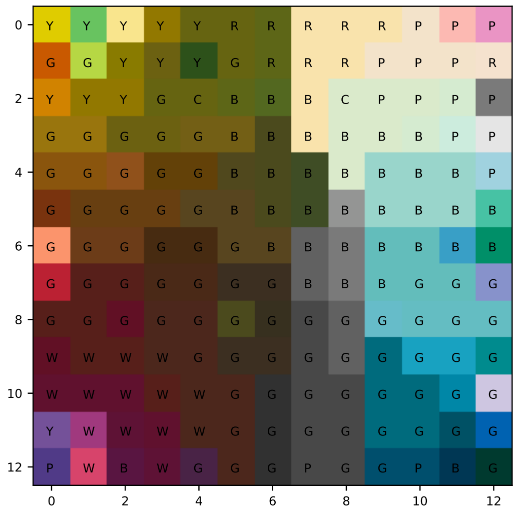

(**e**) Learnt SOM Cells

Reference

1. Cook, R.S.; Kay, P.; Regier, T. The world color survey database. In *Handbook of Categorization in Cognitive Science*; Elsevier: Amsterdam, The Netherlands, 2005; pp. 223–241.
